# *De Novo* and Rare Variants at Multiple Loci Support the Oligogenic Origins of Atrioventricular Septal Heart Defects

**DOI:** 10.1371/journal.pgen.1005963

**Published:** 2016-04-08

**Authors:** James R. Priest, Kazutoyo Osoegawa, Nebil Mohammed, Vivek Nanda, Ramendra Kundu, Kathleen Schultz, Edward J. Lammer, Santhosh Girirajan, Todd Scheetz, Daryl Waggott, Francois Haddad, Sushma Reddy, Daniel Bernstein, Trudy Burns, Jeffrey D. Steimle, Xinan H. Yang, Ivan P. Moskowitz, Matthew Hurles, Richard P. Lifton, Debbie Nickerson, Michael Bamshad, Evan E. Eichler, Seema Mital, Val Sheffield, Thomas Quertermous, Bruce D. Gelb, Michael Portman, Euan A. Ashley

**Affiliations:** 1 Division of Pediatric Cardiology, Stanford University School of Medicine, Stanford University, Stanford, California, United States of America; 2 Cardiovascular Institute, Stanford University School of Medicine, Stanford University, Stanford, California, United States of America; 3 Department of Pathology, Stanford University School of Medicine, Stanford University, Stanford, California, United States of America; 4 University of California San Francisco Benioff Children’s Hospital Oakland, University of California San Francisco, San Francisco, California, United States of America; 5 Department of Vascular Surgery, Stanford University School of Medicine, Stanford University, Stanford, California, United States of America; 6 Division of Cardiovascular Medicine, Stanford University School of Medicine, Stanford University, Stanford, California, United States of America; 7 Departments of Biochemistry, Molecular Biology, and Anthropology, Pennsylvania State University, University Park, Pennsylvania, United States of America; 8 College of Engineering, University of Iowa, Iowa City, Iowa, United States of America; 9 College of Public Health, University of Iowa, Iowa City, Iowa, United States of America; 10 Department of Pathology, University of Chicago, Chicago, Illinois, United States of America; 11 Wellcome Trust Sanger Institute, Hinxton, Cambridge, United Kingdom; 12 Department of Genetics, Yale University, New Haven, Connecticut, United States of America; 13 Howard Hughes Medical Institute, Chevy Chase, Maryland, United States of America; 14 Department of Genome Sciences, University of Washington, Seattle, Washington, United States of America; 15 Department of Pediatrics, University of Washington, Seattle, Washington, United States of America; 16 Department of Pediatrics, University of Toronto, Toronto, Ontario, Canada; 17 Division of Medical Genetics, University of Iowa Carver College of Medicine, Iowa City, Iowa, United States of America; 18 Mindich Child Health and Development Institute, Icahn School of Medicine at Mt. Sinai, New York, New York, United States of America; University of Alabama at Birmingham, UNITED STATES

## Abstract

Congenital heart disease (CHD) has a complex genetic etiology, and recent studies suggest that high penetrance *de novo* mutations may account for only a small fraction of disease. In a multi-institutional cohort surveyed by exome sequencing, combining analysis of 987 individuals (discovery cohort of 59 affected trios and 59 control trios, and a replication cohort of 100 affected singletons and 533 unaffected singletons) we observe variation at novel and known loci related to a specific cardiac malformation the atrioventricular septal defect (AVSD). In a primary analysis, by combining developmental coexpression networks with inheritance modeling, we identify a *de novo* mutation in the DNA binding domain of *NR1D2* (p.R175W). We show that p.R175W changes the transcriptional activity of Nr1d2 using an *in vitro* transactivation model in HUVEC cells. Finally, we demonstrate previously unrecognized cardiovascular malformations in the *Nr1d2*^*tm1-Dgen*^ knockout mouse. In secondary analyses we map genetic variation to protein-interaction networks suggesting a role for two collagen genes in AVSD, which we corroborate by burden testing in a second replication cohort of 100 AVSDs and 533 controls (*p* = 8.37e-08). Finally, we apply a rare-disease inheritance model to identify variation in genes previously associated with CHD (*ZFPM2*, *NSD1*, *NOTCH1*, *VCAN*, and *MYH6)*, cardiac malformations in mouse models (*ADAM17*, *CHRD*, *IFT140*, *PTPRJ*, *RYR1* and *ATE1)*, and hypomorphic alleles of genes causing syndromic CHD (*EHMT1*, *SRCAP*, *BBS2*, *NOTCH2*, and *KMT2D)* in 14 of 59 trios, greatly exceeding variation in control trios without CHD (*p* = 9.60e-06). In total, 32% of trios carried at least one putatively disease-associated variant across 19 loci,suggesting that inherited and *de novo* variation across a heterogeneous group of loci may contribute to disease risk.

## Author Summary

Congenital heart disease (CHD) is a leading cause of childhood morbidity in the developed world. There are few prevalent clinical risk factors and though it is possible that up to 90% of risk for CHD may be genetic, the number of genes clinically associated with disease is small. Rather than grouping disparate CHD phenotypes as other studies have done, we studied a single specific malformation- the atrioventricular septal defect (AVSD). Instead of recurrent variation in a handful of genes, we observed *de novo* and inherited variation in 19 genes associated with human disease, syndromic loci, and genes implicated in cardiac development by mouse knockout. The number of loci identified support the longstanding hypothesis of a complex oligogenic inheritance for a single malformation and suggest that analyses of CHD data to include inherited variation may uncover additional loci contributing risk for cardiac malformations.

## Introduction

Congenital heart disease (CHD) is the most common congenital malformation and the most common cause of mortality during the first year of life in the United States [1,2]. Most cases occur sporadically without a strong family history or identifiable genetic syndrome, and the primary heritable basis of most non-syndromic congenital heart disease has yet to be identified [3,4]. Studies of affected kindreds and syndromic disease have revealed high-penetrance mutations at a small number of key loci [5]. Exome sequencing and studies of structural variation of mixed cardiac phenotypes focusing on *de novo* events have identified novel disease loci in 4–10% of participants [6,7]. However the remaining majority of non-syndromic subjects in exome and CNV studies are without an identified genetic cause.

Atrioventricular septal defects (AVSD) are a rare cardiac malformation associated to date with a handful of canonical genes in cardiac development (*NKX2-5*, *GATA4*, *GATA6*, *CRELD1)* and may co-occur with certain rare syndromes. A recent study discovered causal variation in the nuclear receptor *NR2F2* in 4% of 125 subjects with AVSD, pinpointing a single additional disease-associated gene [7]. However the 4–10% discovery rate in studies of CHD highlight the observation that for any individual gene, highly penetrant *de novo* coding mutations may only account for a small portion of disease incidence, a phenomenon similar to the sporadic occurrence and complex genetics of neurodevelopmental disorders [8]. Therefore, expanding the scope of analysis in studies of CHD to include both inherited and *de novo* variation in multiple genes could increase the sensitivity of genetic studies of this heterogeneous group of oligogenic diseases [9–13].

**Fig 1:**
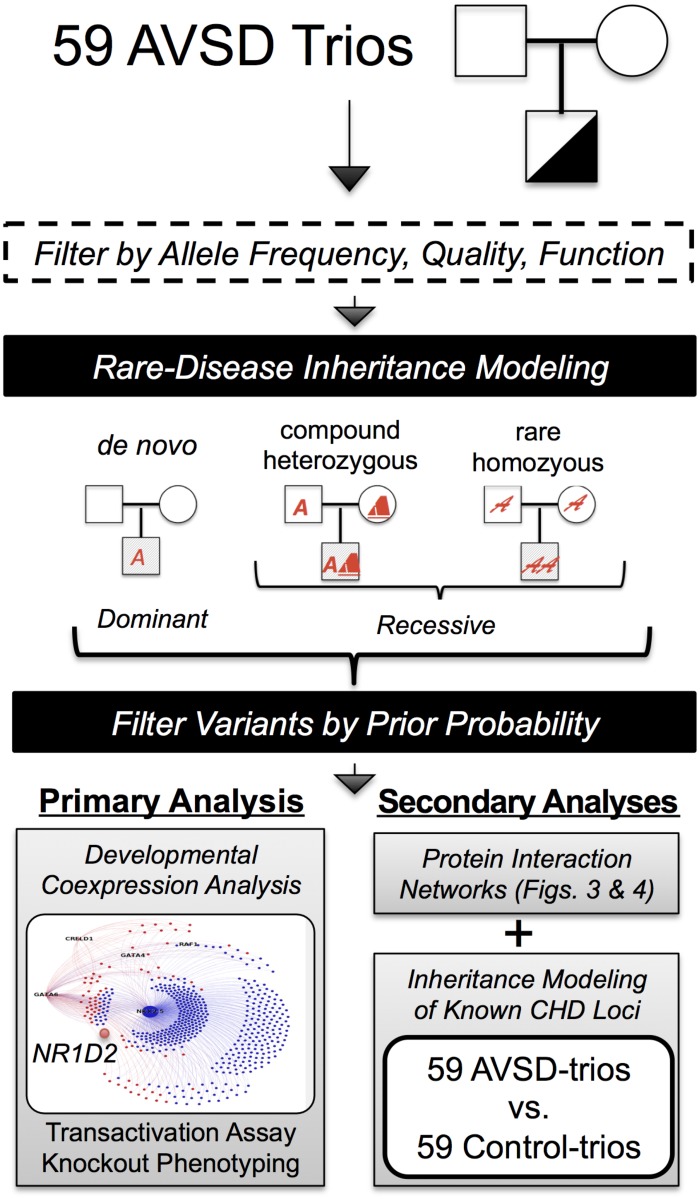
Analytical approach for disease gene discovery in a cohort of AVSD trios. Protein altering variants meeting allele frequency, quality, and read depth cutoffs from 59 trios were sorted with an inheritance model consistent with rare disease which combines *de novo*, compound heterozygous, and rare homozygous variants to collate variants into a list of genes [16]. Three analytical approaches were applied to the final dataset. Primary Analysis. *De novo* variants in the AVSD trios were cross-referenced with the genes in a module highly enriched for CHD and cardiac development obtained from unsupervised weighted-gene coexpression network analysis to identify a novel AVSD gene. Secondary Analyses. Variants from the AVSD trios were mapped onto a protein interaction network followed by burden testing in a replication cohort (details in Fig 3). In a final analysis employing inheritance modeling, *de novo*, compound heterozygous, and rare homozygous loci observed in the AVSD trios were compared with a predefined list of genes associated with human or mouse cardiac malformations. Statistical comparisons were performed in a control group of 59 control trios without CHD.

To this end we assembled a multi-institutional cohort combining a discovery cohort of 59 trios with non-syndromic AVSD and a replication cohort of 100 single affected individuals and performed a genetic survey by exome sequencing and array-CGH. In a primary analysis we identified a novel candidate gene for AVSDs using inheritance modeling and prior knowledge of early cardiac gene expression (Fig 1). In secondary analyses, we searched protein interaction networks to identify the contribution of additional loci to this rare cardiovascular malformation. Finally we explored the contribution of rare inherited variation in genes related to other types of human and mouse cardiac malformations to AVSD.

## Results

### The RTG Pipeline Yields a Highly Sensitive and Accurate Variant Call Set

We determined the sensitivity of our informatics approach using a recently described consensus standard dataset of exonic 24,734 variants from NA12878 [14]. Raw exome data from a well-characterized individual was analyzed with BWA/GATK 3.2 best practices and the RTG version 3.3.2 software pipeline (Real Time Genomics Inc., Hamilton, New Zealand). RTG displayed greater sensitivity detecting 84.5% consensus standard variants compared to 79.9% for BWA/GATK (S1 Table). Using the RTG pipeline we analyzed exome sequencing data on 159 individuals with AVSD but without a syndrome (a discovery cohort of 59 trios, and a replication cohort of 100 singletons derived largely from a published study of AVSDs (S2 Table), along with 710 controls without congenital heart disease (59 trios, 533 singletons). The affected patients were situs solitus with a simple AVSD. Patients with other cardiac defects, heterotaxy, anatomical malformations, or developmental delay were excluded. Across all individuals, called variants displayed a median Ti/Tv ratio of 3.10, and a median of 89.6% phased genotypes within trio probands, which suggested an overall highly sensitive and accurate variant call set (S3 Table). All *de novo* variants and insertions/deletions of interest were confirmed by Sanger sequencing.

### A Rare-Disease Inheritance Model and Developmental Coexpression Networks Identify *NR1D2*

Within the 59 AVSD trios and 59 control trios we analyzed variants with minor allele frequency of 0.03 or less in the 61,468 multiethnic individuals in the EXaC consortium [15]. With the remaining protein-altering variants, we applied a rare-disease inheritance model to select only variants displaying classical inheritance patterns associated with sporadic presentation of a rare disease (*de novo*, homozygous, compound heterozygous) (Fig 1) [16]; this filtering process yielded a list of 710 variants in 399 genes in the 59 AVSD trios (S4 Table).

To identify novel genes involved in cardiac development and disease among the 399 loci, we reanalyzed 72 digital gene expression datasets derived from 22 tissues during mouse embryonic development (www.mouseatlas.org) [17] (S1 Fig). The tissue types included the AV-canal along with 5 other cardiac tissues, and 16 other tissues from other organs or structures. After constructing co-expression modules using unsupervised weighted-gene coexpression network analysis, we observed that one of the co-expression modules expressed in mouse AV-canal tissue included four of six genes known to cause AVSDs (*GATA4*, *GATA6*, *NKX2-5*, and *CRELD1*, *p* = 6.57e-05, one-tail hypergeometric test) along with 69 of 756 genes related to other human or mouse cardiac malformations (S5 Table) (*p* = 7.81e-08, one-tail hypergeometric test). These observations suggested the discovery of a co-expression module highly enriched for genes related to heart development and cardiac malformations.

**Fig 2:**
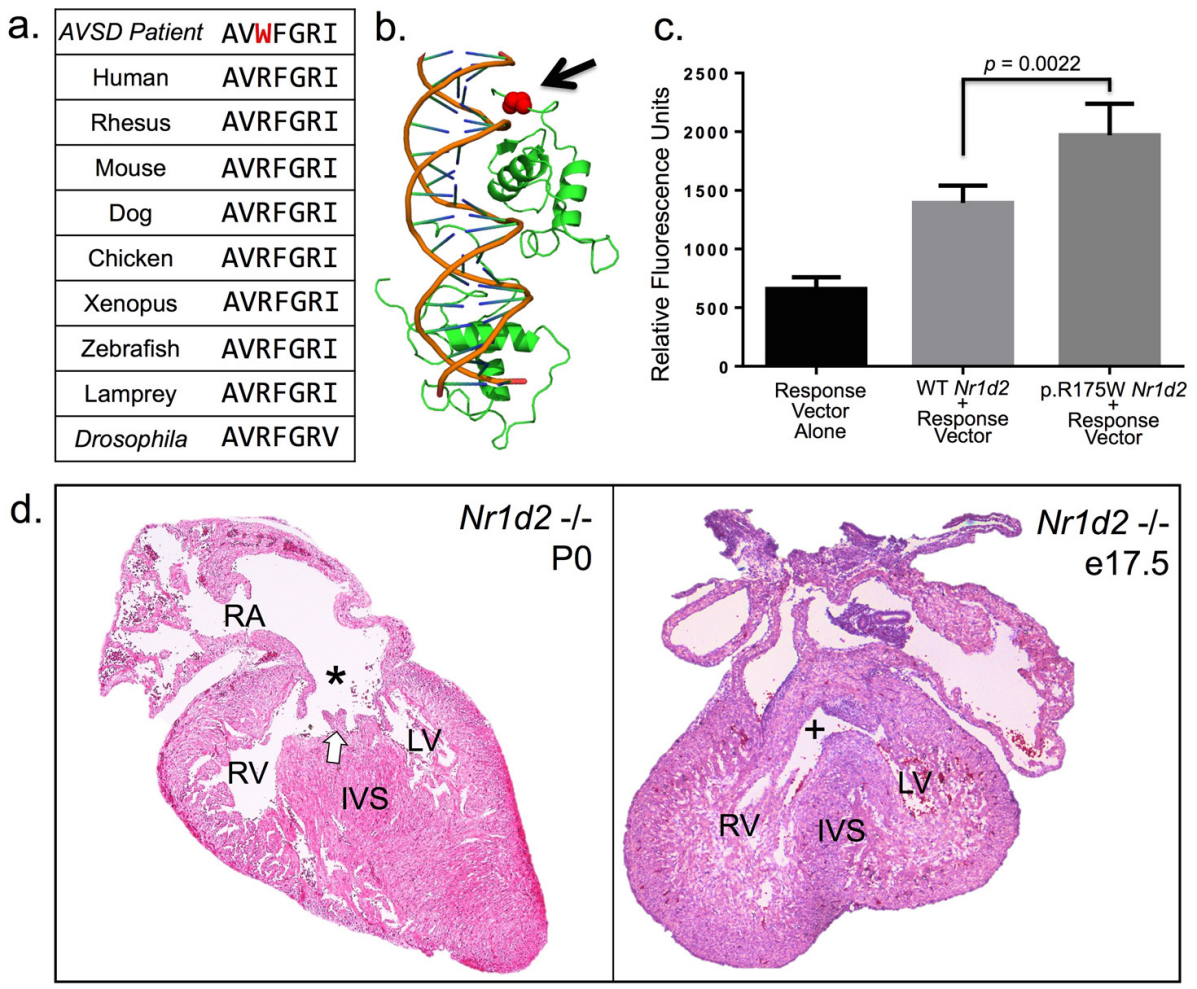
Mutation at a highly conserved arginine residue in the DNA binding domain of *NR1D2* is associated with AVSD. **(a)** Multiple alignment of amino acid residues in the C-terminal aspect of the DNA binding domain of NR1D2 show strong conservation through evolution to the *Drosophila* paralog Eip75B. **(b)** A 3d representation of the crystal structure of NR1D1 complexed to DNA. The NR1D1 and NR1D2 DNA binding domains display 96% sequence identity (S3 Fig), and the altered arginine residue (black arrow) contacts the minor groove of DNA in the C-terminal portion of the DNA binding domain (RCSB 1A6Y) [82]. **(c)** The p.R175W mutation shows increased transcriptional activity in a cell culture assay (*p = 0*.*0022*, two-tailed t-test). A wild-type or mutant *Nr1d2* construct was transfected in HUVEC cells along with a vector containing a synthetic response element driving a GFP reporter. Mean and standard deviation for each condition are displayed. **(d)** Two representative cardiac lesions in *Nr1d2*^*tm1-Dgen*^ *-/-* mouse hearts. An oblique coronal section of the heart of a spontaneously deceased P0 *Nr1d2*^*tm1-Dgen*^ *-/-* pup reveals a primum ASD and suggests a common AV-valve (white arrow) indicative of AVSD (star). A coronal section of an e17.5 *Nr1d2*^*tm1-Dgen*^ *-/-* heart shows an inlet VSD (plus sign) which is a type of VSD closely related to AVSDs in cardiac development [78,83]. Abbreviations: LV-left and RV-right ventricles, RA-right atrium, IVS- interventricular septum, VSD-ventricular septal defect, ASD-atrial septal defect.

Intersecting the genes in this unique coexpression module (S6 Table) with the 399 genes identified by the rare-disease inheritance model, two probands from the 59 AVSD trios displayed *de novo* mutations. One proband had a missense mutation in a non-conserved residue of *KCNJ3* and a second proband had a missense variant in *NR1D2*. The *KCNJ3* variant has been observed in low frequencies in European and African populations in the ExAC database and the available literature on *Kcnj3* knockout animals did not suggest occult cardiovascular malformations [18,19]. By contrast, the *NR1D2* variant causes an arginine to tryptophan mutation at position 175 (p.R175W) in a highly conserved DNA binding domain (Fig 2a and 2b). *NR1D2* is a transcriptional co-repressor and modulator without a described role in cardiac development [20]. *De novo* mutations in *NR1D2* or any gene in the cardiac malformation module were absent from 59 control trios without congenital heart disease. Among the 61,468 putatively healthy individuals cataloged in the ExAC database there was only a single non-synonymous mutation in the surrounding five protein residues surrounding the p.R175W allele. Overall the data were suggestive that this *de novo* allele might impact the function of *NR1D2*.

### The p.R175W Mutation Impacts Transcriptional Activity of Nr1d2 *in vitro*

To characterize the transcriptional activity of the p.R175W mutation we developed an *in vitro* transactivation assay performed in HUVEC cells, which employed a murine *Nr1d2* expression vector co-transfected with an *Nr1d2* response element (RE) consisting of 5 tandem repeats of a conserved binding site upstream of a minimal CMV promoter driving GFP. As *NR1D2* is thought to act as a transcriptional co-repressor, a positive change in transcriptional activity of the RE vector may represent a decrease in the transcriptional co-repressor activity of *Nr1d2*. In this *in vitro* assay, the p.R175W mutation displayed an increased transcriptional activity relative to the wild-type allele (Fig 2c), which suggested that the p.R175W mutation in a conserved region of the DNA binding domain might functionally impair the native co-repressor function of *NR1D2*.

### The *Nr1d2*^*tm1-Dgen*^ -/- Mouse Shows AVSDs and Related Cardiovascular Malformations

Though reports of a previous characterization of an *Nr1d2* knockout allele did not show cardiovascular malformations [20], a percentage of homozygous knockout animals have been reported to die within hours of birth consistent with the presence of hemodynamically significant cardiac malformations [21]. Two pairs of heterozygous founder animals were bred, yielding 17 pups (2 wild type, 7 *Nr1d2*^*tm1-Dgen*^ +/-, and *8 Nr1d2*^*tm1-Dgen*^ -/-) which did not deviate obviously from expected Mendelian allelic ratios. Upon careful histological examination of two spontaneously deceased *Nr1d2*^*tm1-Dgen*^ *-/-* pups at P0 we detected a previously undescribed AVSD phenocopy (Fig 2d). We performed additional matings of +/- and -/- animals, and sacrificed mothers to obtain embryos at e16.5 and e17.5. A single -/- animal at e16.5 displayed an AVSD and a single -/- animal at e17.5 displayed an inlet ventricular septal defect which is closely related to AVSDs (Fig 2d). In total, 4 out of 15 -/- hearts assayed displayed a cardiac defect. Thus by combining inheritance modeling with a gene-coexpression network enriched for genes causing CHD, we identified a variant in *NR1D2* which impacts transcriptional activity *in vitro*, and uncovered previously unrecognized cardiovascular malformations in an *Nr1d2* knockout allele. Together these data suggest a new role for the transcriptional repressor *NR1D2* in cardiac development and human disease.

### Protein Interaction Networks Identified in the AVSD-trios are Expressed in Cardiac Development

**Fig 3:**
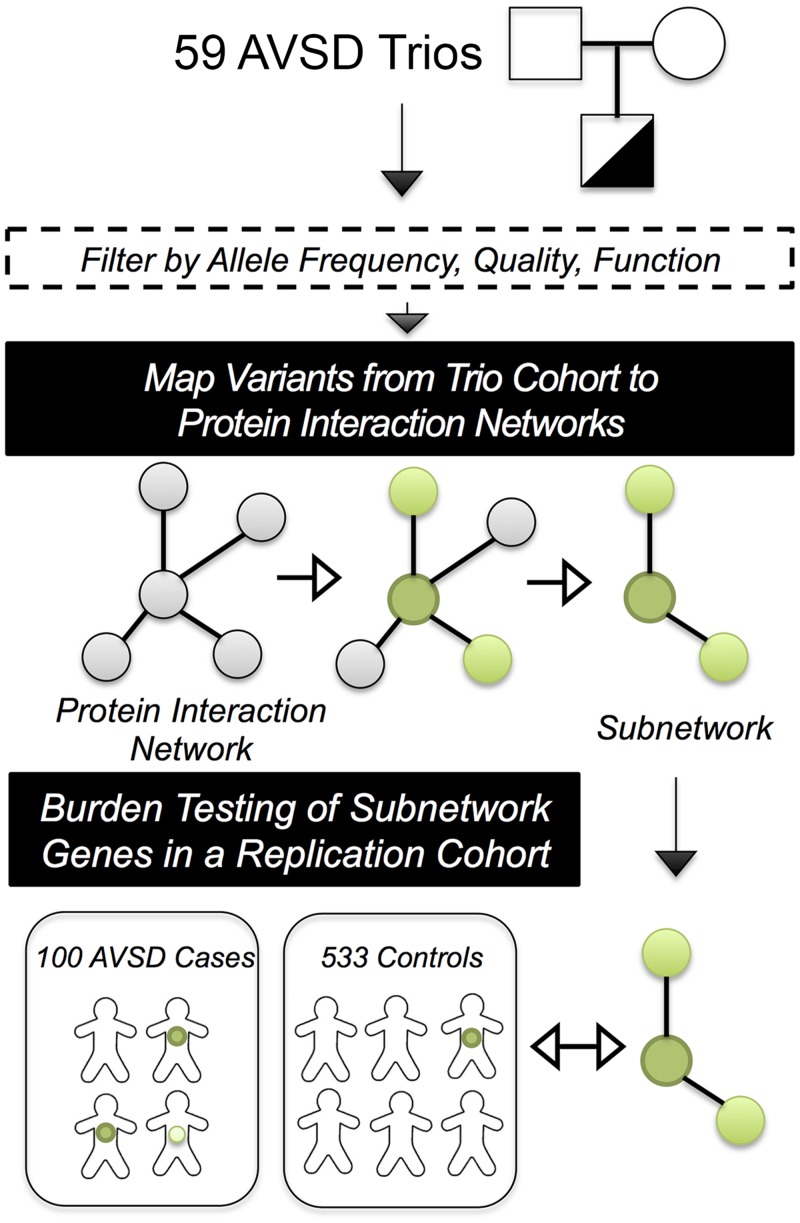
An illustration of discovery of protein interaction networks for AVSD and validation by burden testing in separate a replication cohort. Filtered SNPs and CNVs from the AVSD trio probands are mapped to a protein interaction network (represented by grey dots and black lines), and the network is pruned to yield subnetworks (green dots and black lines). The subnetworks represent variants in genes which have verified interactions at the protein level, often constituting a portion of a signaling pathway or enzymatic complex [22]. To validate the disease association of the individual subnetworks discovered in the trios, we performed burden testing for each subnetwork in a replication cohort of 100 AVSD cases originating largely from a separate study of AVSDs [10] along with 533 controls without congenital heart disease.

The relatively low discovery rate in rare-variant association studies of CHD suggests that alternative analytical approaches may be necessary to distinguish the contribution of novel loci to disease risk [6,7]. Protein interaction networks have successfully integrated known disease genes to discover the impact of novel loci in neurodevelopmental disorders and cancer, thus in a secondary analysis we employed an algorithm which searches protein-interaction data for over-representation of genetic variation within interacting proteins in the AVSD trios [22] (Fig 3).

**Fig 4:**
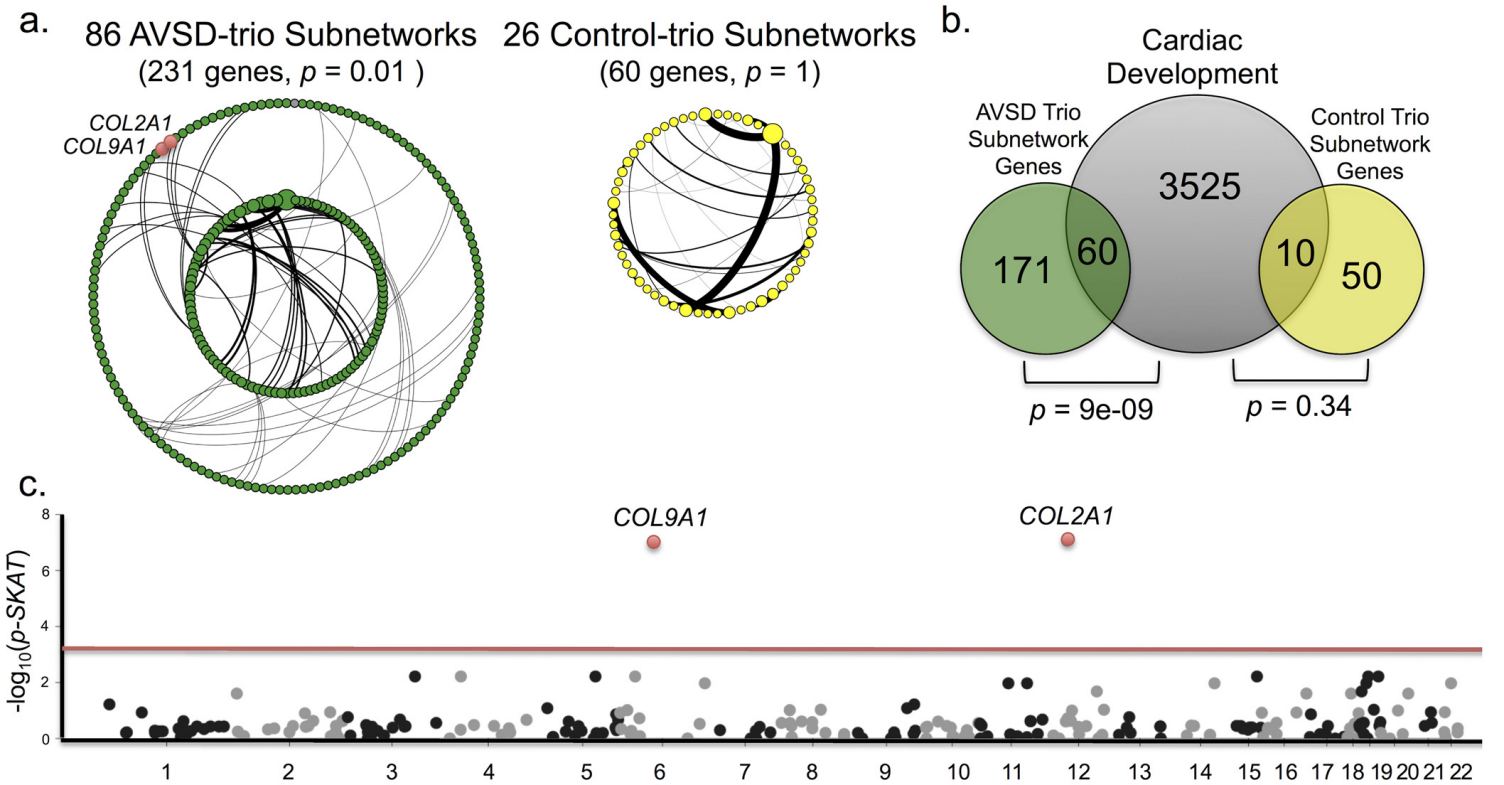
Protein interaction networks identify collagen genes putatively associated with atrioventricular septal defects. **(a)** A network diagram displaying protein-protein interactions between genes. A greater number of networks (n = 86) and genes (n = 231) are detected by protein-interaction analysis in AVSD-trio subnetworks (green nodes) relative to the networks (n = 26) and genes (n = 60) detected in the Control-trio subnetworks (yellow nodes). A median *p*-value from a protein interaction network permutation procedure is reported for each group of subnetworks, which shows an enrichment of true protein-protein interactions in the AVSD-trio subnetworks (*p* = 0.01) that is not observed in the control trio subnetworks (*p* = 1). The black lines indicate a protein-protein interaction between two nodes in the subnetwork and the weight of the line corresponds to “heat” value output by the Hotnet2 algorithm. The number of protein interactions for each observed gene corresponds to the size of the node. The two collagen genes in the AVSD-trio subnetworks (*COL2A1* and *COL9A1*) are labeled and the nodes highlighted in red. The networks from each group of trios are arranged in a circular layout. **(b)** A Venn diagram comparing genes/variants in the AVSD-trio subnetworks (green circle) and Control-trio subnetworks (yellow circle) to genes expressed during early mouse cardiac development (grey circle). The number of genes in each dataset and their overlap with other datasets is indicated and the *p*-value from a hypergeometric test is reported. A greater proportion of genes in the AVSD-trio subnetworks (60 of 231 genes, *p* = 9e-09) are found to be expressed in the early cardiac development dataset, compared to the control-trio subnetworks (10 of 60 genes, *p* = 0.34). **(c)** The subnetwork comprised of *COL2A1* and *COL9A1* are highlighted in a Manhattan plot of test statistics from the SKAT linear weight test for each of the 86 AVSD-trio subnetworks identifies an elevated burden of variation in a replication cohort of 100 AVSD subjects and 533 control subjects. The *p*-value is plotted for the chromosomal position of each gene within the subnetwork. A significance cutoff of 5.81e-04 was derived from the Bonferroni correction of 86 tests performed.

Including protein altering single nucleotide mutations and CNVs derived from the discovery cohort of 59 AVSD trios, the algorithm identified 86 enriched subnetworks of interacting proteins containing 2 to 7 genes. By comparison, applying the algorithm to 59 control-trios identified 26 subnetworks of interacting proteins containing only 2 to 4 genes (Fig 4a). Using a procedure where the protein interaction network is randomly permuted, the genes within the AVSD-trio subnetworks were found to be enriched for true protein-protein interactions (median *p* = 0.01, network permutation procedure), while true protein-protein interactions were not observed within the control trio subnetworks (median *p* = 1.0, network permutation procedure) [23] (Fig 4a).

To further characterize the protein interaction networks detected, we compared the subnetworks to gene expression in mouse cardiac development (S8 Table). Genes within the AVSD-trio subnetworks were strongly overrepresented during mouse heart development (*p* = 9e-09, one-tailed hypergeometric test) while genes within the control-trio subnetworks were not (*p* = 0.34, one-tailed hypergeometric test) (Fig 4b). Thus, mapping genetic variation in the AVSD trios to protein interaction networks identifies 86 enriched subnetworks with deleterious variation in 231 genes that are preferentially expressed during cardiac development, a phenomenon not seen in the control trios without CHD.

### Protein Interaction Networks Identified in the AVSD-trios are Validated by Burden Testing in a Replication Cohort

To validate the genetic associations suggested by the discovered AVSD-trio subnetworks, we assembled a separate replication cohort of 100 singleton individuals (S2 Table) and performed burden testing for each of the 86 protein networks (Fig 3). After Bonferroni correction for multiple hypothesis testing, a single subnetwork from the AVSD trios composed of a pair of interacting collagen genes (*COL2A1*, *COL9A1*) (Fig 4a and 4c) displayed an elevated burden of rare coding variation in 100 affected individuals with AVSD compared to 533 controls without congenital heart disease (*p* = 8.37e-08, SKAT linear weighted test) (Fig 4c). Interestingly, the two genes *COL2A1* and *COL9A1* have evolutionarily conserved roles in cardiac development in both zebrafish and mouse [24–26]. One mouse knockout allele of *Col2a1* displays cardiac valve abnormalities [27], and mutations in both genes are associated with Stickler syndrome where 46% of patients are affected with congenital dysfunction of the mitral valve [28]. Together the functional data on *COL2A1* and *COL9A1* accompanied by the identification of these genes with two separate methodologies in two separate cohorts of AVSD patients, support a potential association with other congenital structural malformations of cardiac valve tissue such as AVSDs.

### A Rare-Disease Inheritance Model Identifies Known CHD Loci in 23% of Trio Probands

Outside of novel genes identified by developmental coexpression and protein interaction analyses, we wished to examine the impact of genes known to play a role in cardiovascular development or CHD in our cohort. Interestingly, *de novo* single nucleotide mutations in genes previously associated with AVSD (*NKX2-5*, *GATA4*, *GATA6*, *EVC*, *CRELD1*, *NR2F2*) were absent and we did not detect genes with recurrent *de novo* mutations.

In an effort to categorize and catalog variation at known CHD loci within the 710 variants in 399 genes identified by the rare-disease inheritance model in the 59 AVSD-trios (S4 Table), we assembled a predefined group of 756 loci associated with any human or mouse cardiac malformation (S5 Table). Among the genes identified by the rare-disease inheritance model in the 59-AVSD probands (S4 Table), we observed inherited and *de novo* variation in 16 genes (Table 1 and S7 Table) [29]. Four of the affected probands displayed variation in more than one gene. In a set of 59 control trios without congenital heart disease we applied the identical variant calling pipeline and rare-disease inheritance model. Comparing the number of AVSD probands with inherited mutations in the identified 16 genes to unaffected controls, we observed 16 mutations in the AVSD-cases and only a single variant in controls (*p* = 9.60e-06, Fisher's exact test), and additional simulations confirmed an unusual distribution of mutations in the AVSD-cases compared to controls was unlikely to be a chance occurrence (*p* = 1.23e-06, Monte Carlo simulation). As an additional negative control we compared mutations in a list of 43 genes associated with congenital ocular malformations between the 59 AVSD cases and 59 controls; there was only a single inherited variant among the AVSD trios and none within the control trios (*p* = 1.0, Fisher’s exact test). Together these results suggest a preponderance of *de novo* and inherited variation in genes associated with human or mouse cardiac malformations detected in the AVSD trios which greatly exceeded similar variation in control trios.

**Table 1:** AVSD trio probands display *de novo* and inherited variation in genes related to human and mouse cardiac malformations.

Gene	Proband	Inheritance	Amino Acid Changes	Prior Evidence
*ADAM17*	3721	compound heterozygous	p.L97R & p.S90L	Mouse Phenotype [43,44]
*RYR1*	1131	compound heterozygous	p.E30V & p.D4500H	Mouse Phenotype [42]
*CHRD*	7952	compound heterozygous	p.A159V & p.T362I	Mouse Phenotype [47]
*PTPRJ*	3731	compound heterozygous	p.V372I & p.E173K	Mouse Phenotype [46]
*IFT140*	8522	compound heterozygous	p.E1065K & p.V108M	Mouse Phenotype [41]
*ATE1*	28	homozygous rare	p.L71F	Mouse Phenotype [45]
*NOTCH1*	2933	compound heterozygous	p.V2285I & p.R509H	Clinical Association
*NSD1*	155	compound heterozygous	p.A933P & p.R361S	Clinical Association
*ZFPM2*	2020	compound heterozygous	p.E30G & p.S388G	Clinical Association
*MYH6*	2023	compound heterozygous	p.A936V & p.D208N	Clinical Association
*VCAN*	54	compound heterozygous	p.V81I & p.E199G	Clinical Association
*MYH6*	54	homozygous rare	p.E1295Q	Clinical Association
*SRCAP*	7952	compound heterozygous	p.S377N & p.E1697G	Syndrome with CHD [49]
*KMT2D*	2002	compound heterozygous	p.R652H & p.R83Q	Syndrome with CHD [52]
*NOTCH2*	155	compound heterozygous	p.L2408H & p.D1327G	Syndrome with CHD [51]
*BBS2*	135	compound heterozygous	p.G688R & p.L62fs	Syndrome with CHD [50]
*BBS2*	3731	compound heterozygous	p.A504V & p.K231E	Syndrome with CHD [50]
*EHMT1*	2028	*deNovo*	p.R260Q	Syndrome with CHD [49]

Among 710 variants in 399 genes displaying *de novo*, homozygous, compound heterozygous inheritance in the AVSD-trio probands we observed 16 genes associated with human or mouse cardiac malformations. Additional variant annotations are found in S4 Table.

Within the discovery cohort of 59 trios we also assessed structural variation by array CGH and read-depth analysis from exome studies. One patient was observed to have a 3.7 Mb *de novo* deletion at 8p23.1 encompassing 43 genes including *GATA4* (Table 2a). An additional paternally inherited duplication at chr22:21,989,140–23,627,391 partially overlapping the congenital heart disease-associated 22q11.2 duplication syndrome region was also detected [30]. Additional CNVs with a previous association to CHD were identified in the singleton subjects (Table 2b) [31]. CNVs in these regions were absent from the trio and singleton controls. Thus, including inherited variants, *de novo* mutations, and structural variation, rare deleterious variants or CNVs in genes with strong prior evidence for association with congenital heart disease were observed in 14 of 59 or 23% of affected trios.

**Table 2:** Discovered CNVs associated with AVSDs and other cardiac malformations.

**a. Trios**				
**Coordinates (hg19)**	**Length**	**Type & Inheritance**	**Genes**	**Additional Information**
22:21989140–23627391	1,638,251	Duplication, paternally inherited	28 genes including *MAPK1*	Overlaps with 22q11.2 duplication region [30]
8:8175651–11853818	3,678,167	Deletion, *de novo*	43 genes including *GATA4*	Canonical CHD gene [29]
**b. Singletons**				
**Coordinates (hg19)**	**Length**	**Type**	**Genes**	**Additional Information**
Y:119064–2562294	2,443,230	Deletion	13 genes, pseudoautosomal region	Previously associated with conotruncal CHD [31]
1:145645983–145763756	117,773	Duplication	5 genes including *CD160 PDZK1*	1q21.1 risk locus [29]
4:5692835–5800635	107,800	Deletion	*EVC*, *EVC2*	Causal gene in CHD associated Ellis-van-Creveld syndrome
8:10755273–11929256	1,173,983	Deletion	14 genes including *GATA4*	Canonical CHD gene [29]

Reported CNVs were larger than 100,000 bp, and detected by at least two of three independent methodologies (arrayCGH, consensus read depth based analysis; Conifer & XHMM, or a TaqMan CNV assay) and overlapped known CHD loci and were absent from controls. Coordinates refer to the hg19 build of the human genome reference.

## Discussion

In this study combining both exome-sequencing and array-CGH for a single specific cardiac malformation, we observed *de novo* and inherited variation in 19 genes associated with human disease, syndromic loci, and genes implicated in cardiac development by mouse knockout. In the absence of recurrent *de novo* events in a moderately sized cohort of 159 affected individuals, we applied an array of analytical techniques to look for both *de novo* and inherited variation associated with AVSDs.

### Coexpression Networks Suggest a Novel CHD Locus

When combined with inheritance analysis, a gene-coexpression network derived from mouse development allowed us to identify a previously unrecognized role for the transcriptional repressor *NR1D2* in cardiac development and human disease. Our experimental studies suggested that the observed p.R175W mutation impacts the transcriptional activity of murine *Nr1d2*, and we observed previously unrecognized cardiac malformations in the *Nr1d2*^*tm1-Dgen*^ knockout mouse. Yet within a cohort of 159 affected individuals, there was only a single patient with a *de novo* mutation in *NR1D2*, which highlights the underlying genetic heterogeneity of CHD and the utility of applying orthogonal datasets to pinpoint causal variation.

The -/- animals for the *Nr1d2*^*tm1-Dgen*^ allele display incomplete penetrance; a majority of animals do not display cardiovascular malformations and develop normally to adulthood displaying phenotypes related to circadian rhythm and abnormal lipid metabolism [20]. *Nr1d2* may retain multiple roles in modulating transcription but is most clearly described as a transcriptional repressor, therefore a mutation in the DNA binding domain might impact *Nr1d2* to binding to target sequences resulting in a “de-repression” transcriptional targets of *Nr1d2*. A knockout allele such as *Nr1d2*^*tm1-Dgen*^ might similarly “de-repress” targets of *Nr1d2*. From the standpoint of transcriptional repression, increased transcription of the p.R175W mutant observed *in vitro* may represent a decrease in transcriptional repression relative to the wild-type protein, and as such could be entirely consistent with the phenotype of a mouse knockout allele. Interestingly, *NR1D2* is a well-characterized component of the molecular clock, and further studies would be necessary to investigate *NR1D2* as a link between the molecular clock and timing of cardiac development.

Chromatin remodeling factors have recently been implicated as primary and secondary causal factors in CHD [6,32], and both newly discovered factors *NR1D2* and *NR2F2* may play integrated roles in chromatin remodeling during cardiac development. The key histone deacetylase HDAC1 is directly activated by NR1D2 binding and indirectly activated by NR2F2 via PROX1 [33–35]. Additionally NR1D2 may function upstream of NR2F2, modulating the auto-regulatory activity of NR2F2 via HDAC1 and the glucocorticoid receptor GR complex [36,37]. Further experiments are necessary to delineate the tissue localization, timing, expression, and functional roles of these two transcription factors and their role in chromatin modulation and transcriptional regulation during cardiac development.

### Exploring Oligogenic Inheritance with Protein Interaction Networks

Within a complex cellular or tissue signaling pathway, capturing the genetic variation in one or more interacting proteins has yielded novel candidate genes in cancer and neurodevelopmental disorders [22,38]. Adapting a tool designed to search protein interaction networks in cancer, we identified a small number of variants in genes within the AVSD trios, two of which (*COL2A1* and *COL9A1*) were subsequently validated in burden testing of a separate replication cohort of 100 individuals at a statistically significant threshold. Independent experimental data implicates these genes in the development of the cardiac valve structures, and links these genes to a genetic syndrome that includes abnormalities of the cardiac valves among a host of other phenotypes. Though our observations are not firmly conclusive of a causal role for *COL2A1* and *COL9A1* in the pathogenesis of AVSDs, they are supportive of such a role, and we believe, consistent with the idea that network-based approaches may be fruitfully applied to gene discovery in CHD phenotypes.

### Absence of Recurrent Mutations in Canonical AVSD Genes

Surprisingly, with the exception of a deletion encompassing *GATA4* seen in one trio subject and one singleton subject (Table 2), we did not discover *de novo* coding mutations or gene dosage alterations within the 59 trios in canonical AVSD genes (*NKX2-5*, *EVC*, *CRELD1*, *GATA6)* or at the newly discovered CHD risk locus *NR2F2*. This finding is consistent with studies of CHD examining candidate genes [39] and exome sequencing where protein-altering variants in any single gene are reported in no more than 1–4% of patients [7]. The absence of recurrent *de novo* variants in a cohort of 59 patients with AVSD is in striking contrast with other cardiac conditions such as long QT syndrome where pathogenic coding variation in only 5 genes accounts for disease in 70% patients [40]. We hypothesize that the absence of *de novo* variation observed at canonical loci in a cohort of this size reflects the complexity of CHD genetics and highlights the utility of considering alternative inheritance patterns to detect disease-associated variation.

### Rare Inheritance Models Identify Genes Associated with CHD and Cardiac Development

Among a list of 756 genes with either a clinical or experimental association to cardiac malformations (S5 Table) we observed rare inherited variants in the AVSD trios that were not seen in control trios [29]. In genes clinically associated with CHD we observed compound heterozygous variants inherited *in trans* in *ZFPM2*, *NSD1*, *NOTCH1*, *VCAN*, and *MYH6* and rare homozygous variants in *MYH6*. Variation was observed in 9 additional genes including compound heterozygous variants in *ADAM17*, *CHRD*, *IFT140*, *PTPRJ*, and *RYR1* and rare homozygous variants in *ATE1*, and the presence of heart defects in mouse knockout models for these loci supports their association with human cardiac malformations.

In an independent forward mutagenesis screen *Ift140* causes AVSD among a variety of congenital malformations [41]. The calcium channel *RYR1* is associated with skeletal myopathies and malignant hyperthermia, but primum atrial septal defects in one mouse allele suggest a role in early cardiac development [42]. The metalloproteinase *ADAM17* may link *NOTCH1* signaling in cardiac valve development to the left-right patterning of the heart [43,44]. Individual knockout alleles of *CHRD*, *PTPRJ*, and *ATE1* each show defects in heart development recapitulating different human malformations [45–47]. Importantly, we observed rare inherited variation in genes with experimental or clinical evidence for a role in cardiac development and CHD within the AVSD trios, but rare inherited variation in these same genes was largely absent from the control-trios.

### Syndromic Alleles in Nonsyndromic Patients

Despite excluding syndromic features and developmental delay from our patients at the time of recruitment, we observed inherited and *de novo* variation in genes causing syndromic disease that include heart malformations. A *de novo* mutation was detected in an unknown protein domain of *EHMT1* the causal gene in Kleefstra syndrome, compound heterozygous variants inherited in trans were observed in *SRCAP* which was recently associated with Floating-Harbor syndrome, two individuals showed compound heterozygous mutations in *BBS2* which causes Bardet-Biedel syndrome, a fifth individual displayed compound heterozygous mutations in *NOTCH2* which causes Alagille syndrome, and a sixth individual displayed a compound heterozygous mutation in *KMT2D* the locus implicated in Kabuki syndrome. Each of these multi-organ syndromes is frequently accompanied by AVSD or another related form of congenital heart disease [48–52].

Within a single locus associated with a genetic syndrome, different alleles may vary in their expressivity. We hypothesize that these variants represent hypomorphic alleles of syndromic genes, where the patients affected present only one aspect of the phenotype associated with the syndrome, in this case a phenocopy of a syndromic associated cardiovascular malformation [53]. Indeed on secondary followup, none of the included probands with *EHMT1*, *KMT2D*, *SRCAP*, or *NOTCH2* variants displayed other characteristics of their associated syndromes, while the patients with *BBS2* mutations were not available for review (additional phenotypic information on patients carrying syndromic alleles is detailed in the S1 Text). Supporting the possibility of hypomorphic alleles, there was a striking absence of *de novo* or inherited variants in these syndromic genes within the control trios suggestive that the discovered variants may confer risk for AVSD.

### Limitations and Conclusions

These findings have limitations. Although we excluded patients with a family history of cardiac malformations, in an earlier era of surgical care the parents of the study participants would have been less likely to survive to reproductive age [54]. In our study the parents received only a questionnaire and did not receive screening echocardiogram, thus we cannot rule out that a parent in an included trio may have a *forme fruste* of an AVSD-related malformation such as a cleft mitral valve or ostium primum ASD. Additionally, there is emerging evidence that maternal risk factors (both genetic and environmental) which confer risk for CHD that were not considered in our study design [55–57].

Genetic studies of CHD are challenged by the fact that specific individual malformations are quite rare (AVSD is approximately 0.3 per 10,000 live births), and that any substantial group of patients with a single malformation will contain some population stratification. The unexplored role of non-coding gene regulatory variation in congenital heart disease is not surveyed by our exome-sequencing approach [58,59]. The power of SKAT tests are likely limited by a small cohort size, the heterogeneous genetic backgrounds of the case and control populations, the differences in exome sequence capture and sequencing chemistries employed, the absence of an inheritance model, and most importantly the underlying complex oligogenic architecture of cardiac malformations [12,60,61]. Finally there are no statistical models that account for ethnicity in models of rare-variant transmission, therefore the influence of population stratification or ethnicity upon our rare-inheritance model of disease is not known.

Overall our analysis suggests locus heterogeneity in the pathophysiology of a single cardiac developmental malformation. We observed recurrent variation within three genes (*GATA4*, *MYH6*, and *BBS2*) in only 6 of 59 trios. Including inherited, *de novo*, and discovered loci, 32% of trios displayed one or more putatively contributory mutations in the 19 genes identified. Supported by both experimental and clinical evidence, we suggest that inherited rare variants with a moderate effect size across multiple loci may impact the risk of congenital heart disease in addition to *de novo* variation. Taken together our catalog of 19 loci with experimental evidence for disease among 159 patients is consistent with the long-hypothesized oligogenic inheritance of congenital heart disease [12].

## Materials and Methods

### Ethics Statement, Case Description, and Patient Cohorts

The guidelines of the Declaration of Helsinki were followed and the study was approved by the institutional review board of Stanford University (IRB-23637, IRB-23572) along with each institution from which participants were recruited. Written informed consent was obtained from each participant. Trios or single patients with an AVSD and situs solitus were recruited. We excluded patients with other major congenital malformations, developmental delay, or other types of CHD (excluding patent ductus arteriosus or secundum atrial septal defect). All participants were assessed clinically by pediatric cardiologists or clinical geneticists to exclude other syndromes associated with AVSD or CHD. Participants were obtained from Seattle Children’s Hospital [62], the Pediatric Cardiac Genomics Consortium, the University of Iowa, and reanalyzed from a published study of AVSDs drawn from patients at the University of Toronto [7] (S2 Table).

### Control Cohorts

Exome sequences for 531 control subjects of Caucasian and African American descent without CHD were derived from the atherosclerosis risk in communities (ARIC) consortium warehoused at dbGAP, and from local recruitment efforts. An additional 59 control trios (healthy parents with a healthy child) were obtained from the Simons Foundation. Raw sequence data in the form of bam or fastq files was re-aligned and re-analyzed with the below-described bioinformatic pipelines.

### Exome Sequencing and CNV Detection

DNA was isolated by standard techniques from either whole blood, saliva samples, or immortalized lymphoblasts. Exome sequencing was performed for all complete trios and single affected individuals at two academic centers (University of Washington or Yale University) and two commercial sequencing providers with the SeqCap EZ Human Exome Library v2.0 (Roche NimbleGen, Madison, Wisconsin, USA), SureSelectXT Human All Exon V4 (Agilent Technologies Inc., Santa Clara, California, USA), or a proprietary capture library based on the Agilent SureSelectXT Human All Exon V5 platform (Personalis, Corp, Menlo Park, California, USA). Paired-end sequencing was performed on Illumina HiSeq 2500 machines with 75-, 100-, or 150-bp read lengths in all but two trios, which were sequenced with 33 bp paired end reads. For all included samples Median Ts/Tv was 3.10 while coverage depth was 43.8x (S3 Table). Exome sequencing on single individuals from the Toronto cohort was performed as described [7], and for subset of unrelated individuals raw fastq files were obtained and reanalyzed via the below described bioinformatics platform for the purposes of reanalysis.

AVSD trios and the singleton/replication patients not originating from Toronto were assayed for CNVs by array comparative genomic hybridization (CGH) by using a custom chip described previously or the SurePrint G3 Human CGH Microarray Kit, 8x60K (Agilent Technologies Inc., Santa Clara, California, USA) with described protocols [31,62]. When a CNV was detected in an affected participant, a dye-swap experiment was repeated to ensure reproducibility and the parents were then assayed if available to determine inheritance status. For detection of smaller variation, two exome CNV detection assays using read depth data were also employed for all participants [63,64]. When there was agreement in a structural variant call between at least two calling methods for any variant of interest, we employed an orthogonal CNV genotyping assay when DNA was available (for all of the AVSD trios and the singleton/replication patients not originating from Toronto). The CNV genotyping assay was carried out with 10 ng of DNA per manufacturer instructions against the RNaseP CNV reference assay (Life Technologies, Carlsbad, California, USA). The assay was run on a ViiA 7 Real Time PCR System (Life Technologies, Carlsbad, California, USA) for 40 cycles under standard reaction conditions, and CNV genotypes were called with the copycaller software [65].

### Genotyping Pipeline

Two genotyping pipelines were tested. A rapid and sensitive commercial software package, rtg-core version 3.3.2 was applied to the raw exome sequence data for mapping, pedigree-aware variant calling, and genotype filtration (Real Time Genomics Inc., Hamilton, New Zealand) [66] to the UC Santa Cruz human genome reference sequence (hg19) (S1 Script). A second pipeline based on the HugeSeq BWA/GATK HaplotypeCaller pipeline was also employed for purposes of comparison.

To determine the accuracy of our genotyping pipeline we re-genotyped available exome data from NA12878 for comparison to a recently described gold standard dataset of 24,734 variants from this individual [14]. This set of variants from the consensus standard dataset was limited to the exome capture region of the nextera kit all human exome v2 (Illumina Corp, San Diego, California, USA) and regions of suspicious variant quality [67] were excluded to yield a true positive dataset of 24,734 true positive variants from NA12878. The vcfeval tool from RTG was used for all comparisons of vcf files as it robustly handles the different possible textual representations of insertions, deletions, and substitutions that may be produced by different genotyping algorithms. Using raw fastq files generated by the Garvan Institute, comparing the unfiltered output of both pipelines the two algorithms both called 19,566 variants (79.9%) of the NA12878 true positive dataset (TP) in common. RTG called an additional 1,469 TP, compared to BWA/GATK which only called an additional 198 unique TP variants. Overall RTG displayed a greater unfiltered sensitivity at 84.5% compared to 79.9% for BWA/GATK; therefore we selected the RTG pipeline for further analysis of the cohort (S1 Table).

To classify variants we selected an AVR score of 0.5 to balance a sensitivity of 99.4% and positive predictive value of 90.3% for the purposes of variant discovery. Alignments for all variants of interest were manually inspected with the IGV software, and all *de novo* coding variants and any insertion/deletion of interest was confirmed by direct Sanger sequencing of PCR amplicons, or alternately clonal Sanger sequencing of 12 colonies from cloned PCR amplicons. For burden testing, pooled simultaneous variant calling for all included cases and controls was performed with the RTG population caller and variants filtered for a read depth of 8 and AVR score of 0.5. Analyses were limited to the regions of intersection of the bed files of the exome capture kits obtained from the respective manufacturers. Known artifactual variants arising from exome sequencing were removed at the time of variant filtration [67,68].

### Statistical Analysis

All statistical analyses employed the R language for statistical computing version 3.1 unless otherwise specified. For population analyses we selected 8,940 snps from the Affymetrix Genome-Wide Human SNP Array 6.0 (Affymetrix, Santa Clara, California, USA) common to the five exome capture protocols employed in the study. All included probands were re-genotyped for this limited set of variants, and combined with individual level data from 1032 diverse samples of known ethnicity from the 1000 genomes project. The MDS and kinship modules from the KING software were used to estimate ethnic background and five individuals displaying cryptic familial relationships from the Toronto cohort among the singletons were identified and excluded from further analysis [69]. A multinomial linear model was built for each population and the presence of admixture from the populations of the 1032 known samples, and used to infer ethnic background and the presence of admixture in the included probands. Self-reported ethnicity was available for 547 individuals, which was 97.6% concordant with predicted ethnicity.

Protein altering variants were sorted for minor allele frequency less than 0.03 and prioritized by inheritance patterns consistent with rare disease (*de novo*, rare homozygous, and compound heterozygous) using the trioTools module from the STMP package [16]. Protein-altering variants from the 59 trios were selected, haplotypes constructed, and variants phased with the PLINKseq software package [70]. Imputation was not performed thus all variants analyzed originated from the primary genotyping pipeline.

After sorting for inheritance consistent with rare disease (*de novo*, rare homozygous, and compound heterozygous), variants were collated and assembled into lists. A blacklist of genes with low prior likelihood of causality was excluded from further analysis, which included genes with a residual variation intolerance score greater than 90 and genes with copy number polymorphisms [68,71,72].

For statistical comparisons between the groups of trios, the number of individuals with rare inheritance (*de novo*, rare homozygous, or compound heterozygous) in these genes in the 59 trios and 59 controls was counted and compared with a Fisher’s Exact test. As the underlying distribution combining *de novo*, rare homozygous, and compound heterozygous inheritance into a unified “rare inheritance” model is not readily estimated from available genotyping data, and because variant detection (particularly *de novo* variation) may vary systematically with the technical aspects of sequencing (variant calling algorithm, read depth, exome capture platform, and sequencing chemistry) we estimated empiric *p*-values by Monte Carlo simulation. To simulate an underlying distribution we performed permutations drawing 16 genes randomly from a list of 18,495 protein-coding genes, the rare inheritance events for each random list counted within 59 cases and 59 controls by individual, and a Fisher’s exact test applied.

The *p*-value was estimated with the formula *(r+1)/(n+1)* where *n* is the number of simulated replicate samples and r is the number of test statistics exceeding the calculated test statistic from the observed data (*p* = 9.60e-06). This simulation procedure suggested an empiric *p*-value of 1.23e-06, a similar order of magnitude as the *p*-value calculated from the observed data.

Gene modules or subnetworks identified by protein interaction networks in the HotNet2 algorithm (see below), were subjected to burden testing with the SKAT linear weight test and a Bonferroni correction for multiple hypothesis testing employed [73,74]. The first four principal components from a principal components analysis of all variants were used as covariates for burden testing. Within the SKAT algorithm variant weighting was derived from a beta density function (p_i_, 1, 25) where p_i_ is the minor allele frequency. For variant weighting, minor allele frequencies were derived from the 61,428 individuals in the multiethnic EXaC dataset (version 0.2 http://exac.broadinstitute.org/), while for variants not observed or reported in the EXaC dataset minor allele frequencies were calculated from frequency observed among the genotyped individuals. The quantile-quantile plots for the SKAT linear weight test of 86 subnetworks (inclusive of combinations of 231 single genes—see below) suggest that the test-statistics derived are controlled for ethnicity or other systematic differences between cases and controls such as coverage (S2 Fig).

### Co-expression Network Analysis from Mouse Organ Development

Unique tag-count data from 74 SAGE libraries representing 22 tissues constructed as a part of the mouse atlas of gene expression project were downloaded from www.mouseatlas.org [17]. SAGE tags were mapped with the Burrows-Wheeler aligner to the 115,746 unique mouse RefSeq transcripts downloaded from UC Santa Cruz website (http://genome.ucsc.edu) and tag data converted to a digital gene expression format constituting a tag counts per transcript using custom Perl scripts. Tags matching pseudogenes were removed. Using the R environment for statistical computing, tag counts by transcript were normalized by library size with the EdgeR package and collapsed to gene by connectivity with the WGCNA package [75,76]. A standard WGCNA workflow for digital gene expression was applied to the normalized and collapsed data for coexpression module construction followed by correlation to the tissue of origin and annotation with the human orthologous gene name when available (S2 Script). To independently assess the predictive capacities of our unsupervised network building procedure, we observed that the developmental expression of 13 well characterized congenital heart disease genes are accurately localized by the network model to their appropriate cardiac tissue, suggesting excellent specificity for detecting developmental cardiac related gene expression (S9 Table). Comparison of variant and gene lists for under- and over-representation were performed with a one-tailed hypergeometric test. Gene expression networks were visualized with the Gephi software package [77]. Because the DMP is a key structure in development of the atrioventricular septum [78] and was not explicitly included in the original set of micro-dissected tissues from the mouse atlas of organ development (www.mouseatlas.org), we added the DMP gene expression data to the cardiac development gene expression set. Among genes expressed in the DMP we selected the most highly expressed 1000 genes across 6 datasets generated from the posterior second heart field including the dorsal mesenchymal protrusion. The microdissection of the posterior heart field was performed in the laboratory of Dr. Moskowitz at embryonic mouse tissue at E9.5, subject to reverse-transcription, amplification, and sequencing by The University of Chicago Genomics Core (GSE75077).

### Protein Interaction Networks with HotNet2

Variant data annotated with the STMP package [16] including SNVs and CNVs from the 59 AVSD trio probands and 59 control trio probands was converted and formatted with custom python scripts, and included all protein altering variants with a minor allele frequency in EXaC of 0.02 or less were included in the analysis using four included protein interaction networks. For each of three protein interaction networks (Multinet, IrefIndex9, and HINT [22]) four delta values were derived with the network permutation procedure and applied to identify subnetworks. The resulting subnetworks identified by the HotNet2 algorithm were manually inspected for validity, and the subnetworks from the MultiNet protein interaction networks were chosen for further analysis. For each set of subnetwork sizes ranging from 2 to 10, the HotNet2 algorithm derives a *p*-value from the hypergeometric distribution comparing the observed number of subnetworks of size *n* within in a dataset compared to an expected number of subnetworks. The expected number of subnetworks is derived from a computationally intensive network permutation procedure; in this case the MultiNet protein interaction network was subject to 100 permutations limiting the range of *p*-values to a minimum *p*-value to 0.01 and maximum *p*-value to 1. For the AVSD-trio subnetworks the median p-value was 0.01 across the nine subnetwork sizes and four derived delta values, in comparison to the control-trio subnetworks where the median p-value was 1.0; this suggested an enrichment in variants occurring in genes with true protein-protein interactions within the AVSD-trios (rather than randomly occurring simulated protein-protein interactions in the permuted networks). A single output run utilizing the MultiNet protein interaction network applying a delta value of 0.00126036397514 yielded 86 subnetworks containing 231 genes in the AVSD trios, and the 86 subnetworks were subject to burden testing in the singleton cohort (see above). Protein interaction data were processed with custom python and shell scripts for visualization using the Gephi software tool [77].

### Gene Lists

#### Rare inherited variants in the AVSD-trios

The variant calling and filtering process in the 59 AVSD-trios produced a list 710 variants in 399 genes (S4 Table).

#### The AVSD Coexpression Module

The coexpression module highly expressed in the AV-Canal contains 934 genes including four of six known genes causing AVSDs, including *NKX2-5*, *GATA4*, *GATA6*, and *CRELD1* (S6 Table).

#### Known CHD genes in human & mouse

To develop a list of 756 genes related to cardiovascular malformations in humans an mice we combined a list of 85 genes previously associated with human cardiac malformations [29] along with a list of 671 mouse genes known to cause cardiac malformations similar to human disease obtained from the mouse genome database (accessed 2/3/15; Mouse Phenotype IDs 10425,10435,3105,10412,6113,285,10422,281,279,10429,2925) (S5 Table).

#### Cardiac development

Genes from the coexpression modules with correlation to one of the six developing cardiac tissues (atrioventricular canal, branchial arches, heart tube, atria, ventricle, and outflow tracts) were combined with RNAseq data from a key developmental structure in atrioventricular septal morphogenesis, the dorsal mesenchymal protrusion. These data combined yielded an early cardiac development gene set of 3,595 loci (S8 Table) [78].

#### Ocular malformations

To serve as a list of control genes unrelated to cardiac malformations, we developed a list of genes related to ocular malformations (n = 43) derived from the mouse genome database (accessed 7/15/15; Mouse Phenotype IDs 000516, 0002092) and the literature [79] and excluded genes appearing in the cardiac malformation gene list (S10 Table).

### Mouse Experiments

Live breeding pairs of the B6;129P2-Nr1d2^tm1Dgen^/H mouse line (hereafter referred to as *Nr1d2*^*tm1-Dgen*^) were obtained from the European Mouse Mutant Archive (Munich, Germany). Animals were in housed and cared for in AAALAC accredited facilities under standard conditions with oversight and approval from the Stanford University APLAC committee (protocol APLAC-11334). Euthanasia was carried out under anesthesia with isofluorane following APLAC and AAALAC guidelines using carbon dioxide followed by cervical dislocation. Genotyping was performed by PCR of toe or tail clippings with gel electrophoresis using standard methodology with two primer pairs (CAAGTAACAAGCCTGGGACATAAAG and CTTCGTAGAGGGAGTAATATGACAC yield a 517 bp PCR product from the WT allele; CAAGTAACAAGCCTGGGACATAAAG and GACGAGTTCTTCTGAGGGGATCGATC yield a 757 bp product from the knockout allele). Two pairs of heterozygous founder animals were bred, yielding 17 pups (2 wild type, 7 *Nr1d2*^*tm1-Dgen*^ +/-, and *8 Nr1d2*^*tm1-Dgen*^ -/-) which did not deviate obviously from expected Mendelian allelic ratios. Spontaneous death within hours after birth occurred in a single +/- and two -/- animals. The thoracic and abdominal cavities of spontaneously deceased homozygous knockout animals were visually inspected to examine the great vessels and visceral situs which were normal, and hearts dissected out and subject to sectioning and H & E staining by standard techniques. Sectioning of tissue samples was performed following either embedding of frozen sections followed by dehydration and fixation in 10% neutral buffered formalin or alternately fixation in 3% paraformaldehyde followed by alcohol dehydration and paraffin embedding. We performed additional matings of +/- and -/- animals, and sacrificed mothers to obtain embryos at e16.5 and e17.5. Visualization was performed by brightfield microscopy on a Nikon 90i Eclipse upright with a DS Fi1 camera with a 20x objective. Both P0 *Nr1d2*^*tm1-Dgen*^ -/- animals displayed atrioventricular septal defects, a single -/- animal at e17.5 displayed an inlet ventricular septal defect, and single -/- animal at e16.5 displayed an AVSD. In total, 4 out of 15 -/- hearts assayed displayed a cardiac defect. Spontaneously deceased animals were analyzed, and additionally euthanasia of pregnant female mice was performed to obtain embryonic animals.

### Cell Culture Experiments

The crystal structure of the DNA binding domain of NR1D1 (RCSB 1A6Y) was visualized with the PyMOL Molecular Graphics System, Version 1.7.4 Schrödinger, LLC (New York, New York, USA). A construct containing wild-type murine *Nr1d2* cDNA construct under control of a CMV promoter in the pCS6 expression vector was obtained from Transomic Technologies Inc. (Huntsville, Alabama, USA), and subject to a site-directed mutagenesis yielding a codon switch at p.R175W which corresponds to the de novo mutation observed in the subject with AVSD (S4a Fig). An *Nr1d2* response element vector was constructed cloning 5 tandem repeats of an evolutionarily conserved NR1D2 binding site REV-DR2 [80,81] upstream of a minimal CMV promoter driving GFP expression using the pSF-MinCMV-daGFP vector (Sigma-Aldrich Inc, St. Louis, Missouri, USA) (S4b Fig). Site-directed mutagenesis and cloning were performed by a commercial provider GENEWIZ Inc. (South Plainfield, New Jersey, USA), and sequence verified in our own laboratory. The three vectors were subject to routine endotoxin free preparation. Commercially available primary HUVEC cells obtained from Cell Applications (San Diego, California, USA) were seeded at 80% confluency in black transparent-flat bottom 96 well plates (Greiner, North Carolina, USA) and transfected the following day with 100ng of each vector (response-element, wild-type or p.R175W) using Lipofectamine 3000 (Life Technologies, Grand Island, New York, USA). Twenty-four hours after transfection, GFP fluorescence was measured on a Tecan Infinite M1000-multimode plate reader (Tecan Group Ltd, Mannendorf, Switzerland). We performed three transfection conditions including response-element+Nr1d2-WT, response-element+Nr1d2-P403W and response-element alone in 24 technical replicates. Autofluoresence of untransfected wells were averaged, and subtracted from the response-element alone and the two experimental conditions. The highest and lowest value from each condition were excluded from analysis yielding 22 technical replicates per experimental condition, and 4 technical replicates for the response element alone. Statistical analysis and graphing of the transfection experiments was performed in Prism 6 Graphpad Software (La Jolla, California, USA).

## Supporting Information

S1 Text Supplementary results. Additional phenotypic descriptions of individuals with AVSDs accompanied by mutations in syndromic genes or large CNVs. For AVSD patients displaying large CNVs (Table 2) or mutations in syndromic alleles, we returned to the medical record or referring provider to look for evidence of other syndromic features or developmental delay not ascertained at the time or enrollment. These phenotypic results are discussed herein. As noted in the Discussion, the two patients with compound heterozygous mutations in *BBS2* were unavailable for follow up.
(PDF)

S1 Fig Coexpression modules are correlated with organ development. A heat map displaying correlation between the tissues included from the developmental SAGE expression data on the X-axis and the inferred module eigengene (represented by arbitrarily assigned colors) on the Y-axis. Red and green colors represent correlation and anti-correlation respectively as indicated on the right hand Figure legend. Modules related to atrioventricular canal development are indicated with a black box, the single module containing 934 genes including AVSD-genes and other genes related to cardiovascular malformations is indicated with the blue arrow and box (S6 Table).
(PDF)

S2 Fig A quantile-quantile plot of *p*-values derived from the SKAT linear weight test of 86 subnetworks in 100 AVSD cases compared to 533 controls. The 86 test-statistics produced by the SKAT linear weighted test for the AVSD-trio subnetworks in the replication cohort display a normal distribution, suggesting the comparisons are adequately controlled for ethnicity or other systematic differences between cases and controls such as read depth or exome-capture kits.
(PDF)

S3 Fig A multiple protein alignment of the DNA binding domains of NR1D1 and NR1D2 shows sequence identity surrounding a conserved arginine residue. The relevant portion of a protein sequence alignment of Q6NSM0 (NR1D2) and P20393 (NR1D1) from UniProt.org (accessed 4/7/15) using standard parameters is shown. The DNA binding domains of the two proteins are delineated by the black text and display 96% sequence identity. The crystal structure of the DNA binding domain of NR1D1 is displayed in Fig 3b. The region of multiple alignment in Fig 3a is highlighted in yellow, and the conserved arginine residue altered in the AVSD patient is highlighted in green.
(PDF)

S4 Fig Diagrams of nucleic acid reagents for the in vitro cell culture experiments for the discovered p.R175W NR1D2 experiment. **(a)** multiple alignment of DNA and protein sequences for wild type and mutant versions of the human and mouse of the NR1D2 protein. **(b)** Synthetic NR1D2 response element cloned into the XmaI site of pSF-MinCMV-daGFP, containing 5 tandem REV-DR2 response elements (highlighted in bold), separated by random DNA sequence generated by a python script.
(PDF)

S1 Script Example command line for read mapping and variant calling using the RTG software package. (PDF)

S2 Script Example R workflow for weighted gene network coexpression network construction and correlation to a tissue specific expression. (PDF)

S1 Table Raw sensitivity of RTG is greater than BWA/GATK before filtering. (PDF)

S2 Table Proband ethnicity and enrollment by site. (PDF)

S3 Table Sequencing and genotyping parameters of case and control subjects. (PDF)

S4 Table Complete list of 710 variants in 399 genes from 59 trios with AVSD displaying rare inheritance. (PDF)

S5 Table Genes associated with cardiac malformations in human disease or mouse knockout model. (PDF)

S6 Table A co-expression module of 934 genes includes *NKX2-5*, *CRELD1*, and *GATA4* is enriched for genes associated with cardiac malformations. (PDF)

S7 Table Rare variant inheritance patterns identify genes associated with cardiac malformations by prior clinical or experimental evidence. (PDF)

S8 Table 3595 genes expressed in cardiac development by weighted gene coexpression analysis. (PDF)

S9 Table 13 well known cardiac developmental genes are appropriately localized to developmental compartment by unsupervised weighted gene co- expression network analysis of SAGE expression data. (PDF)

S10 Table 3595 genes expressed in cardiac development by weighted gene coexpression analaysis. (PDF)
