# Supplementary material for: De Novo and Rare Variants at Multiple Loci Support the Oligogenic Origins of Atrioventricular Septal Heart Defects
Source: PLoS Genet. 2016 Apr 8;12(4):e1005963. doi: 10.1371/journal.pgen.1005963 (PMC4825975; doi:10.1371/journal.pgen.1005963)
Supplement: S1 Text — Additional phenotypic descriptions of individuals with AVSDs accompanied by mutations in syndromic genes or large CNVs. For AVSD patients displaying large CNVs (Table 2) or mutations in syndromic alleles, we returned to the medical record or referring provider to look for evidence of other syndromic features or developmental delay not ascertained at the time or enrollment. These phenotypic results are discussed herein. As noted in the Discussion, the two patients with compound heterozygous mutations in BBS2 were unavailable for follow up. (PDF) [file pgen.1005963.s001.pdf]

SUPPORTING INFORMATION

Supplementary Results

Phenotypic Analysis of Individuals Carrying Syndromic and Disease-Associated Alleles

The discovery of disease-associated alleles prompted careful scrutiny of patient phenotypes which we report in detail here. The patient carrying a compound heterozygous mutation in *SRCAP* compound was enrolled at 5 months of age and was without other abnormalities or malformations. The patient with a compound heterozygous mutation in *RYR1* was enrolled at age 17, and has a history of requiring speech and occupational therapy but is enrolled in school with peers and is without history suggestive of a skeletal myopathy. The individual with a compound heterozygous mutation in *ADAM17* was enrolled at age 13 and displays pectus excavatum along with clinodactyly, but is otherwise developmentally normal. The patient carrying compound heterozygous mutations in *BBS2* and *PTPRJ*, along with a deletion of *GATA4* was enrolled at age 16 months, and displayed slightly downslanted palpebral fissures bilaterally, low set ears, high arched palette, polydactyly, and two moderate sized café au lait spots on the back. The patient with the compound heterozygous mutation in *IFT140* was enrolled at age 9, and has mild developmental delay requiring speech therapy and is obese, but displays no other malformations. Though patients with other cardiac and non-cardiac malformations or neurodevelopmental phenotypes were excluded, ascertainment for neurodevelopment phenotypes was imperfect as some patients were enrolled during infancy.

Phenotypic and CNV analysis of Singleton Cohort

Within the singleton cohort, additional rare CNVs were detected in a handful of loci associated with congenital heart disease. One patient with a large deletion of *GATA4* had pulmonary stenosis and a left diaphragmatic hernia but was without developmental delay. A second patient with a deletion of syndromic gene *EVC2* also had a partial fusion of the aortic valve but was otherwise without syndromic features. Another displayed a duplication at the 1q21.1 locus inclusive of *CD160* and *PDZK1*. One patient had duplication at chrY:119,064-2,562,294 overlapping a pseudoautosomal region recently associated with conotruncal heart defects [31].
