# Supplementary figures and images for: De Novo and Rare Variants at Multiple Loci Support the Oligogenic Origins of Atrioventricular Septal Heart Defects
Source: PLoS Genet. 2016 Apr 8;12(4):e1005963. doi: 10.1371/journal.pgen.1005963 (PMC4825975; doi:10.1371/journal.pgen.1005963)

Figure S1. Coexpression Modules are Correlated with Organ Development.

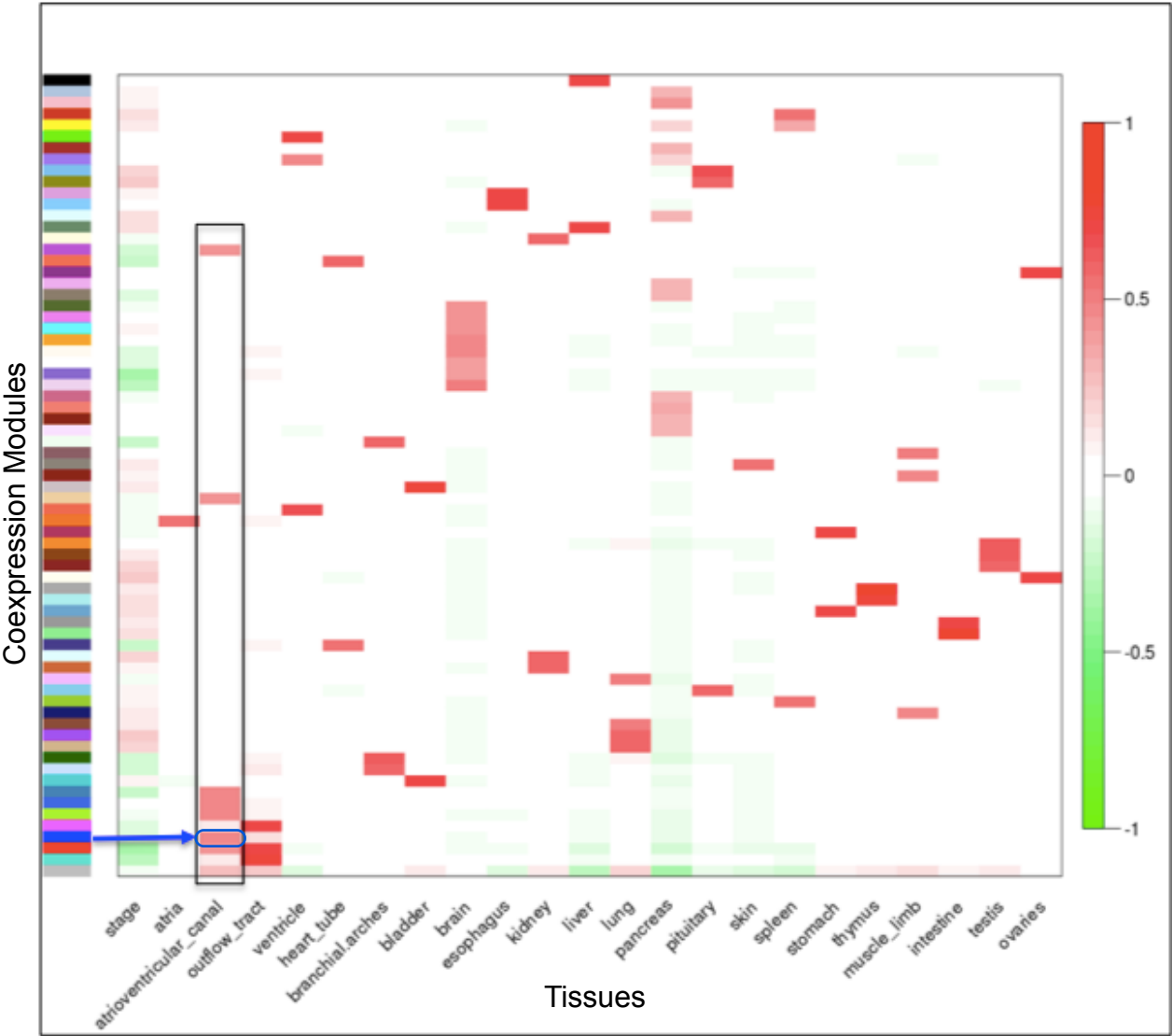

Supplement: S1 Fig — A heat map displaying correlation between the tissues included from the developmental SAGE expression data on the X-axis and the inferred module eigengene (represented by arbitrarily assigned colors) on the Y-axis. Red and green colors represent correlation and anti-correlation respectively as indicated on the right hand Figure legend. Modules related to atrioventricular canal development are indicated with a black box, the single module containing 934 genes including AVSD-genes and other genes related to cardiovascular malformations is indicated with the blue arrow and box (S6 Table). (PDF) [file pgen.1005963.s002.pdf]
