# Supplementary material for: De Novo and Rare Variants at Multiple Loci Support the Oligogenic Origins of Atrioventricular Septal Heart Defects
Source: PLoS Genet. 2016 Apr 8;12(4):e1005963. doi: 10.1371/journal.pgen.1005963 (PMC4825975; doi:10.1371/journal.pgen.1005963)

Figure S2. A Quantile-Quantile Plot of p-values derived from the SKAT linear weight test of 86 subnetworks in 100 AVSD cases compared to 533 controls.

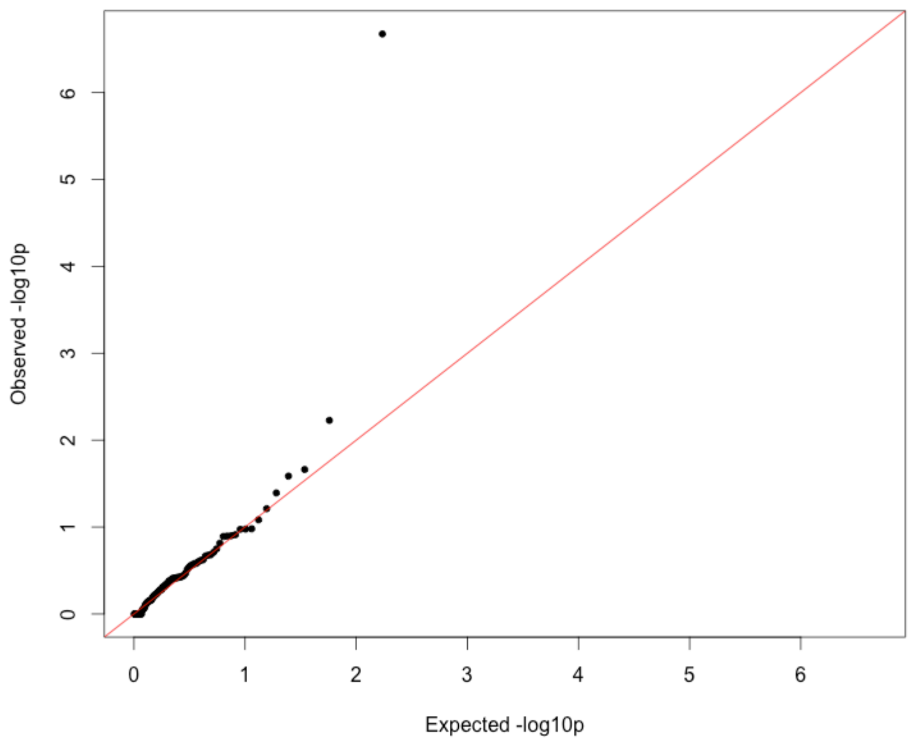

Supplement: S2 Fig — The 86 test-statistics produced by the SKAT linear weighted test for the AVSD-trio subnetworks in the replication cohort display a normal distribution, suggesting the comparisons are adequately controlled for ethnicity or other systematic differences between cases and controls such as read depth or exome-capture kits. (PDF) [file pgen.1005963.s003.pdf]
