# Supplementary material for: De Novo and Rare Variants at Multiple Loci Support the Oligogenic Origins of Atrioventricular Septal Heart Defects
Source: PLoS Genet. 2016 Apr 8;12(4):e1005963. doi: 10.1371/journal.pgen.1005963 (PMC4825975; doi:10.1371/journal.pgen.1005963)
Supplement: S3 Fig — The relevant portion of a protein sequence alignment of Q6NSM0 (NR1D2) and P20393 (NR1D1) from UniProt.org (accessed 4/7/15) using standard parameters is shown. The DNA binding domains of the two proteins are delineated by the black text and display 96% sequence identity. The crystal structure of the DNA binding domain of NR1D1 is displayed in Fig 3b. The region of multiple alignment in Fig 3a is highlighted in yellow, and the conserved arginine residue altered in the AVSD patient is highlighted in green. (PDF) [file pgen.1005963.s004.pdf]

Figure S3. A Multiple Protein Alignment of the DNA Binding Domains of NR1D1 and NR1D2 Shows Sequence Identity Surrounding a Conserved Arginine Residue.

```
NR1D2   90 HSGVTKFSGMVLLCKVCGDVASGFHYGVHACEGCKGFFRRSIQQNIQYKKCLKNENCSIM 149
NR1D1  119 TSNITKLNGMVLLCKVCGDVASGFHYGVHACEGCKGFFRRSIQQNIQYKRCLKNENCSIV 178
            * :**:.*****************************************:*********:

NR1D2  150 RMNRNRCQQCRFKKCLSVGMSRDAVRFGRIPKREKQRMLIEMQSAMKTMMNSQFSGHLQN 209
NR1D1  179 RINRNRCQQCRFKKCLSVGMSRDAVRFGRIPKREKQRMLAEMQSAMNLANNQ-LSSQCPL 237
           *:************************************* ******:   *. :*.:
```
