# Supplementary material for: De Novo and Rare Variants at Multiple Loci Support the Oligogenic Origins of Atrioventricular Septal Heart Defects
Source: PLoS Genet. 2016 Apr 8;12(4):e1005963. doi: 10.1371/journal.pgen.1005963 (PMC4825975; doi:10.1371/journal.pgen.1005963)
Supplement: S4 Fig — (a) multiple alignment of DNA and protein sequences for wild type and mutant versions of the human and mouse of the NR1D2 protein. (b) Synthetic NR1D2 response element cloned into the XmaI site of pSF-MinCMV-daGFP, containing 5 tandem REV-DR2 response elements (highlighted in bold), separated by random DNA sequence generated by a python script. (PDF) [file pgen.1005963.s005.pdf]

Figure S4. Diagrams of Nucleic Acid Reagents for the In Vitro Cell Culture Experiments for the Discovered R175W NR1D2 Experiment.

a.

**WT Human DNA**	GCT	GTT	CGG	TTT	GGT	CGT	ATT
**WT Human Protein**	A	V	R	F	G	R	I
**mutant Human DNA**	GCT	GTT	TGG	TTT	GGT	CGT	ATT
**mutant Human Protein**	A	V	**W**	F	G	R	I
**WT Mouse DNA**	GCT	GTT	CGA	TTT	GGC	CGA	ATT
**WT Mouse Protein**	A	V	R	F	G	R	I
**mutant Mouse DNA**	GCT	GTT	TGG	TTT	GGC	CGA	ATT
**mutant Mouse Protein**	A	V	**W**	F	G	R	I

b.

CCCGGGAGTGA**AGGTCA**TT**AGGTCA**TCATAAGTTT**AGGTCA**AG**AGGTCA**TAGTTTGCTC**AGGTC**
AGA**AGGTCA**GAGTAATGTA**AGGTCA**CT**AGGTCA**GTGAGTAATGTA**AGGTCA**AT**AGGTCA**ATTAA
CCCGGG
