# Supplementary material for: De Novo and Rare Variants at Multiple Loci Support the Oligogenic Origins of Atrioventricular Septal Heart Defects
Source: PLoS Genet. 2016 Apr 8;12(4):e1005963. doi: 10.1371/journal.pgen.1005963 (PMC4825975; doi:10.1371/journal.pgen.1005963)
Supplement: S1 Script — (PDF) [file pgen.1005963.s006.pdf]

Script S1. Example command line for read mapping and variant calling using the RTG software package

```
rtg-core-3.3.2/rtg map \
-l PCGC_1131_A.bam_1.fq -r PCGC_1131_A.bam_2.fq -o ./PCGC_1131_A \
-t ./hg19/SDF/ -T 48 -F fastq -q sanger --bam \
--sam-rg "@RG\tID:PCGC_1131_A\tSM:PCGC_1131_A\tPL:ILLUMINA" \
--bed-regions=./Nimblegen.v2.Tiled.44.1.bed

rtg-core-3.3.2/rtg family -o ./PCGC_1131_family \
-t ./hg19/SDF/ --father PCGC_1131_B --mother PCGC_1131_A \
--son PCGC_1131_P -m illumina \
--avr-model illumina-pe-1000g-phase2.avr \
../PCGC_1131_P/mated.bam ../PCGC_1131_P/unmated.bam \
../PCGC_1131_B/mated.bam ../PCGC_1131_B/unmated.bam \
../PCGC_1131_A/mated.bam ../PCGC_1131_A/unmated.bam \

rtg-core-3.3.2/rtg vcffilter \
-I /PCGC_1131_individual/snps.vcf.gz \
-o /PCGC_1131_individual/PCGC_1131_individual.vcf \
--include-bed=./Nimblegen.v2.Tiled.44.1.bed
```
