# Supplementary material for: De Novo and Rare Variants at Multiple Loci Support the Oligogenic Origins of Atrioventricular Septal Heart Defects
Source: PLoS Genet. 2016 Apr 8;12(4):e1005963. doi: 10.1371/journal.pgen.1005963 (PMC4825975; doi:10.1371/journal.pgen.1005963)
Supplement: S2 Script — (PDF) [file pgen.1005963.s007.pdf]

Script S2. Example R workflow for weighted gene network coexpression network construction and correlation to a tissue specific expression.

```
targets <-readTargets()
trimonlyraw <-readDGE(targets)
keep <- rowSums(cpm(trimonlyraw)>10) >= 2
trimonlyraw <- trimonlyraw[keep,]
trimonlyraw<- calcNormFactors(trimonlyraw)
dim(trimonlyraw)
[1] 70295    74
rowGroups = as.character(lapply(strsplit(as.character(rownames(trimonlyraw)), split=":"),
"[", 2))
trimonlyrawnorm = trimonlyraw$samples$norm.factors * trimonlyraw$counts
collapseobject = collapseRows(trimonlyrawnorm, rowGroups, rownames(trimonlyrawnorm),
method = "MaxMean", connectivityBasedCollapsing=TRUE, methodFunction=NULL,
connectivityPower=1,selectFewestMissing=TRUE, thresholdCombine=NA)
collapsetrim_raw=data.frame(collapseobject$group2row, collapseobject$datETcollapsed)
dim(collapsetrim_raw)
[1] 24266    76

datExpr0 = as.data.frame(t(collapsetrim_raw[, -c(1:2)]));
rownames(datExpr0) = trimonlyraw$samples$files;
gsg = goodSamplesGenes(datExpr0, verbose = 3);

if (sum(!gsg$goodGenes)>0)
printFlush(paste("Removing genes:", paste(names(datExpr0)[!gsg$goodGenes], collapse = ",
")));
if (sum(!gsg$goodSamples)>0)
printFlush(paste("Removing samples:", paste(rownames(datExpr0)[!gsg$goodSamples],
collapse = ", ")));
# Remove the offending genes and samples from the data:
datExpr0 = datExpr0[gsg$goodSamples, gsg$goodGenes]

datExpr_trim_keep_col <- datExpr0

> dim(datExpr_trim_keep_col)
[1]    74 24266

traitData = read.csv("traits2.csv");
samples = rownames(datExpr_trim_keep_col);
traitRows = match(samples, traitData$file);
datTraits_trim_keep_col = traitData[traitRows, -1];
rownames(datTraits_trim_keep_col) = traitData[traitRows, 1];

powers = c(c(1:10), seq(from = 12, to=20, by=2))
```

```
sft = pickSoftThreshold(datExpr_trim_keep_col, powerVector = powers, verbose = 5)

net_trim_collapsecon = blockwiseModules(datExpr_trim_keep_col, power = 7, TOMType =
"unsigned", minModuleSize = 30, reassignThreshold = 0, mergeCutHeight = 0.25,
numericLabels = TRUE, pamRespectsDendro = FALSE, saveTOMs = TRUE, saveTOMFileBase =
"trim_keep_col", verbose = 3, maxBlockSize = 33000)

save.image("trim_keep_col.RData")

mergedColors = labels2colors(net_trim_collapsecon$colors)

plotDendroAndColors(net_trim_collapsecon$dendrograms[[1]],
mergedColors[net_trim_collapsecon$blockGenes[[1]]], "Module colors", dendroLabels =
FALSE, hang = 0.03, addGuide = TRUE, guideHang = 0.05)

moduleLabels = net_trim_collapsecon$colors
moduleColors = labels2colors(net_trim_collapsecon$colors)
MEs = net_trim_collapsecon$MEs;
geneTree = net_trim_collapsecon$dendrograms[[1]];

moduleTraitCor = cor(MEs, datTraits_trim_keep_col, use = "p");
moduleTraitPvalue = corPvalueStudent(moduleTraitCor, nSamples);

textMatrix_cor = paste(signif(moduleTraitCor,2))
dim(textMatrix_cor) = dim(moduleTraitCor);
textMatrix_pvalue = paste(signif(moduleTraitPvalue,1))
dim(textMatrix_pvalue) = dim(moduleTraitCor);

textMatrix = paste(signif(moduleTraitCor,2)," ",signif(moduleTraitPvalue,1),sep = "");
dim(textMatrix) = dim(moduleTraitCor);
labeledHeatmap(Matrix = moduleTraitCor, xLabels = names(datTraits_trim_keep_col),
yLabels=names(MEs), ySymbols = names(MEs),colorLabels=FALSE, colors= greenWhiteRed(50),
textMatrix = textMatrix, setStdMargins = TRUE, cex.text = 0.4, zlim = c(-1,1),cex.lab.y =
0.4, main = paste("Module to Organ Developmental Relationships"))


nGenes = ncol(datTraits_trim_keep_col);
nSamples = nrow(datExpr_trim_keep_col);
# Recalculate MEs with color labels

MEs0 = moduleEigengenes(datExpr_trim_keep_col, moduleColors)$eigengenes
MEs = orderMEs(MEs0)
moduleTraitCor = cor(MEs, datTraits_trim_keep_col, use = "p");
moduleTraitPvalue = corPvalueStudent(moduleTraitCor, nSamples);

probes = names(datExpr_trim_keep_col)
```

```
annot = read.csv(file = "newhomologylist.csv");
probes2annot = match(probes, annot$mouse_geneid)


# Define variable avc_tkc containing the avc_tkc column of datTrait
avc_tkc = as.data.frame(datTraits_trim_keep_col$atrioventricular_canal);
names(avc_tkc) = "avc_tkc"
# names (colors) of the modules
modNames = substring(names(MEs), 3)
geneModuleMembership = as.data.frame(cor(datExpr_trim_keep_col, MEs, use = "p"));
MMPvalue = as.data.frame(corPvalueStudent(as.matrix(geneModuleMembership), nSamples));
names(geneModuleMembership) = paste("MM", modNames, sep="");
names(MMPvalue) = paste("p.MM", modNames, sep="");
geneTraitSignificance_avc_tkc = as.data.frame(cor(datExpr_trim_keep_col, avc_tkc, use =
"p"));
GSPvalue_avc_tkc =
as.data.frame(corPvalueStudent(as.matrix(geneTraitSignificance_avc_tkc), nSamples));
names(geneTraitSignificance_avc_tkc) = paste("GS.", names(avc_tkc), sep="");
names(GSPvalue_avc_tkc) = paste("p.GS.", names(avc_tkc), sep="");

probes = names(datExpr_coltrim)

geneInfo0 = data.frame(probes, humanid = annot$human_geneid[probes2annot],  moduleColor =
moduleColors, geneTraitSignificance_avc_tkc, GSPvalue_avc_tkc)
modOrder = order(-abs(cor(MEs, avc_tkc, use = "p")));

#output csv file with correlation information and pvalues
for (mod in 1:ncol(geneModuleMembership))
{
oldNames = names(geneInfo0)
geneInfo0 = data.frame(geneInfo0, geneModuleMembership[, modOrder[mod]],MMPvalue[,
modOrder[mod]]);
names(geneInfo0) = c(oldNames, paste("MM.", modNames[modOrder[mod]], sep=""),
paste("p.MM.", modNames[modOrder[mod]], sep=""))
}
geneOrder = order(geneInfo0$moduleColor, -abs(geneInfo0$GS.avc_tkc));
geneInfo_avc_tkc = geneInfo0[geneOrder, ];
write.csv(geneInfo_avc_tkc, file = "geneInfo_avc_tkc.csv");
```
