# Supplementary material for: De Novo and Rare Variants at Multiple Loci Support the Oligogenic Origins of Atrioventricular Septal Heart Defects
Source: PLoS Genet. 2016 Apr 8;12(4):e1005963. doi: 10.1371/journal.pgen.1005963 (PMC4825975; doi:10.1371/journal.pgen.1005963)
Supplement: S1 Table — (PDF) [file pgen.1005963.s008.pdf]

Table S1. Raw Sensitivity of RTG is Greater than BWA/GATK Before Filtering

	TP detected	False Negatives	Raw Sensitivity	False Negative Rate
**RTG**	20,901	3,833	*0.845*	*0.155*
**BWA/GATK**	19,771	4,963	*0.799*	*0.201*
