# Supplementary material for: De Novo and Rare Variants at Multiple Loci Support the Oligogenic Origins of Atrioventricular Septal Heart Defects
Source: PLoS Genet. 2016 Apr 8;12(4):e1005963. doi: 10.1371/journal.pgen.1005963 (PMC4825975; doi:10.1371/journal.pgen.1005963)
Supplement: S2 Table — (PDF) [file pgen.1005963.s009.pdf]

Table S2. Proband Ethnicity & Enrollment by Site

Site	Trios	Singletons
**University of Washington**		
African American	-	-
Asian	1	-
Caucasian	17	-
Native American (AMR)	-	1
**University of Iowa**		
African American	-	-
Asian	-	-
Caucasian	26	4
Native American (AMR)	-	-
**PCGC**		
African American	2	2
Asian	1	-
Caucasian	-	4
Native American (AMR)	12	-
**University of Toronto**		
African American	-	-
Asian	-	-
Caucasian	-	87
Native American (AMR)	-	2
**TOTAL**	**59**	**100**
