# Supplementary material for: De Novo and Rare Variants at Multiple Loci Support the Oligogenic Origins of Atrioventricular Septal Heart Defects
Source: PLoS Genet. 2016 Apr 8;12(4):e1005963. doi: 10.1371/journal.pgen.1005963 (PMC4825975; doi:10.1371/journal.pgen.1005963)
Supplement: S3 Table — (PDF) [file pgen.1005963.s010.pdf]

Table S3. Sequencing and Genotyping Parameters of Case and Control Subjects

	AVSD Trios	Control Trios	AVSD Singletons	Control Singletons
Number of Individuals	177	177	100	533
median Ti/Tv	2.40	2.17	3.10	3.12
median Het/Hom	1.84	2.10	1.47	1.54
median Read Depth	41.73	111.18	54.50	47.58
median % phased genotypes (probands)	89.9%	89.0%	n/a	n/a
