# Supplementary material for: De Novo and Rare Variants at Multiple Loci Support the Oligogenic Origins of Atrioventricular Septal Heart Defects
Source: PLoS Genet. 2016 Apr 8;12(4):e1005963. doi: 10.1371/journal.pgen.1005963 (PMC4825975; doi:10.1371/journal.pgen.1005963)
Supplement: S4 Table — (PDF) [file pgen.1005963.s011.pdf]

Table S4. Complete List of 710 variants in 399 genes from 59 Trios with AVSD Displaying Rare Inheritance

Proband	Gene	Inheritance	Residual Variation Intolerance Score	Exonic Function	Amino Acid Change	Minor Allele Frequency EXAC	dbSNP138	PolyPhen2 Score	PolyPhen2 Prediction	GERP Score	Chromosome	Position	Ref	Alt	Quality Score	Paternal Genotype	Maternal Genotype	Proband Genotype
17	*MARCH7*	compound heterozygous	86.299835	nonsynonymous SNV	uc010for.3:c.C599T:p.T200M	0.02	rs76774368	0	B	1.22	chr2	160604514	C	T	1935.4	0/0	0/1	0|1
17	*MARCH7*	compound heterozygous	86.299835	nonsynonymous SNV	uc010for.3:c.G853A:p.A285T	0.02	rs141659304	0	B	-3.38	chr2	160604768	G	A	710.2	0/1	0/0	1|0
2036	*ABCA3*	compound heterozygous	3.7096013	nonsynonymous SNV	uc010bsk.1:c.A3610G:p.S1204G	8.54E-04	rs35089233	0.836	P	4.89	chr16	2334358	T	C	1064.9	0/0	0/1	0|1
2036	*ABCA3*	compound heterozygous	3.7096013	nonsynonymous SNV	uc002cpy.1:c.G863A:p.R288K	5.96E-03	rs117603931	0	B	-6.84	chr16	2369592	C	T	622.6	0/1	0/0	1|0
60	*ABCA4*	compound heterozygous	86.305732	nonsynonymous SNV	uc001dqi.1:c.G505C:p.V169L	2.73E-03	rs41292677	0.993	D	5.08	chr1	94467548	C	G	588	0/1	0/0	1|0
60	*ABCA4*	compound heterozygous	86.305732	nonsynonymous SNV	uc001dqh.3:c.G1654A:p.V552I	2.62E-03	rs145525174	0.92	D	3.63	chr1	94528774	C	T	499	0/0	0/1	0|1
2812	*ABCA4*	compound heterozygous	86.305732	nonsynonymous SNV	uc001dqh.3:c.G6529A:p.D2177N	0.01	rs1800555	0.004	B	5.03	chr1	94463617	C	T	483.2	0/0	0/1	0|1
2812	*ABCA4*	compound heterozygous	86.305732	nonsynonymous SNV	uc001dqh.3:c.A466G:p.I156V	1.30E-03	rs62646863	0	B	-1.46	chr1	94568675	T	C	2879.7	0/1	0/0	1|0
313	*ABHD14A,ACY1*	homozygous rare	NA	nonsynonymous SNV	uc011bea.2:c.G182A:p.R61H	8.20E-03	rs41292350				chr3	52012320	G	A	935.1	0/1	0/1	1|1
155	*ABO*	homozygous rare	NA	nonframeshift substitution	ABO:uc004cda.1:exon4:c.188_189GC						chr9	136135237	AT	GC	3700.4	0/1	0/1	1|1
2020	*ACACB*	compound heterozygous	1.7751828	nonsynonymous SNV	uc001toc.3:c.A2503G:p.M835V	0.02	rs17848802	0.558	P	4.01	chr12	109634834	A	G	1346.1	0/0	0/1	0|1
2020	*ACACB*	compound heterozygous	1.7751828	nonsynonymous SNV	uc010sxm.2:c.G1984A:p.V662M	5.77E-04	rs138582469	0.995	D	5.16	chr12	109690904	G	A	3649.8	0/1	0/0	1|0
2812	*ACACB*	compound heterozygous	1.7751828	nonsynonymous SNV	uc001toc.3:c.A2503G:p.M835V	0.02	rs17848802	0.558	P	4.01	chr12	109634834	A	G	891.8	0/1	0/0	1|0
2812	*ACACB*	compound heterozygous	1.7751828	nonsynonymous SNV	uc001toc.3:c.A3845G:p.Y1282C	6.59E-04	rs149793040	1	D	4.1	chr12	109661672	A	G	2718.8	0/0	0/1	0|1
3761	*ACACB*	compound heterozygous	1.7751828	nonsynonymous SNV	uc001toc.3:c.A2503G:p.M835V	0.02	rs17848802	0.558	P	4.01	chr12	109634834	A	G	763.3	0/1	0/0	1|0
3761	*ACACB*	compound heterozygous	1.7751828	nonsynonymous SNV	uc010sxm.2:c.C1109T:p.A370V			0.036	B	4.32	chr12	109680330	C	T	1039.7	0/0	0/1	0|1
313	*ACTN2*	homozygous rare	2.4652041	frameshift deletion	uc001hyg.2:c.95delC:p.P32fs						chr1	236899903	CC	C	728.4	0/1	0/1	1|1
3721	*ADAM17*	compound heterozygous	82.778957	nonsynonymous SNV	uc010ewz.3:c.T290G:p.L97R			0.003	B	1.61	chr2	9630520	A	C	2144.5	0/0	0/1	0|1
3721	*ADAM17*	compound heterozygous	82.778957	nonsynonymous SNV	uc010ewz.3:c.C269T:p.S90L	0.01	rs55796712	0.003	B	1.66	chr2	9630541	G	A	2781.7	0/1	0/0	1|0
2035	*ADAM28*	compound heterozygous	86.854211	nonsynonymous SNV	uc010lua.3:c.G122A:p.C41Y	2.11E-04	rs145612420	1	D	5.4	chr8	24187586	G	A	1867.6	0/0	0/1	0|1
2035	*ADAM28*	compound heterozygous	86.854211	nonsynonymous SNV	uc010lua.3:c.T1166C:p.L389P	1.33E-03	rs147942437	0	B	-8.9	chr8	24208750	T	C	2267.9	0/1	0/0	1|0
2007	*ADAMTS15*	homozygous rare	84.754659	nonsynonymous SNV	uc010scd.2:c.G397C:p.E133Q	2.70E-03	rs185269810	0.988	D	3.08	chr11	130319265	G	C	977.5	0/1	0/1	1|1
2028	*ADCY1*	homozygous rare	4.2462845	nonsynonymous SNV	uc003tne.4:c.G2818A:p.A940T	0.01	rs45444695	0.026	B	4.87	chr7	45747949	G	A	6593.7	0/1	0/1	1|1
313	*AFAP1L2*	homozygous rare	87.296532	nonsynonymous SNV	uc001lbn.3:c.G46A:p.D16N	9.77E-03	rs41300231	0.243	P	4.63	chr10	116100461	C	T	949.1	0/1	0/1	1|1
3731	*AFM*	homozygous rare	79.464496	nonsynonymous SNV	uc003hhb.3:c.G1184A:p.R395H	0.02	rs41265665	0.013	B	2.04	chr4	74361142	G	A	5345.4	0/1	0/1	1|1
7952	*AFP*	compound heterozygous	71.567587	nonsynonymous SNV	uc003hgz.1:c.A559C:p.K187Q	0.01	rs35765619	0.977	D	3.81	chr4	74308089	A	C	2227.4	0/1	0/0	1|0
7952	*AFP*	compound heterozygous	71.567587	nonsynonymous SNV	uc011cbg.1:c.C80G:p.T27S	1.87E-04	rs139295532	0.235	P	5.09	chr4	74310754	C	G	3774.8	0/0	0/1	0|1
3781	*AGA*	compound heterozygous	86.712668	nonsynonymous SNV	uc003iuu.2:c.C313A:p.L105I	9.56E-03	rs76491548	0.282	P	5.58	chr4	178360811	G	T	2118.6	0/1	0/0	1|0
3781	*AGA*	compound heterozygous	86.712668	nonsynonymous SNV	uc003iuu.2:c.G34T:p.V12L	0.01	rs74626221	0	B	-9.86	chr4	178363496	C	A	383.4	0/0	0/1	0|1
152	*AHI1*	homozygous rare	78.066761	nonsynonymous SNV	uc003qgg.3:c.C838T:p.R280W	0.02	rs13312995				chr6	135751024	G	A	2032.4	0/1	0/1	1|1
23	*AIF1L*	homozygous rare	81.216089	nonsynonymous SNV	uc011mce.1:c.A388G:p.M130V	0.02	rs112844133	0.705	P	4.19	chr9	133995629	A	G	3259.5	0/1	0/1	1|1
3761	*AIF1L*	compound heterozygous	81.216089	nonsynonymous SNV	uc011mce.1:c.T185C:p.M62T	2.16E-03	rs146896674	0.85	P	4.57	chr9	133989973	T	C	2251.9	0/0	0/1	0|1
3761	*AIF1L*	compound heterozygous	81.216089	nonsynonymous SNV	uc011mce.1:c.A388G:p.M130V	0.02	rs112844133	0.705	P	4.19	chr9	133995629	A	G	926.1	0/1	0/0	1|0
2037	*AKAP4*	homozygous rare	84.613116	nonsynonymous SNV	uc004dou.1:c.G860A:p.G287D	5.04E-03	rs141513690	0.072	B	2.46	chrX	49958477	C	T	1123.9	0/1	0/1	1|1
2002	*AKAP6*	compound heterozygous	71.650153	nonsynonymous SNV	uc001wrq.3:c.A1672G:p.N558D	6.82E-03	rs35210906	0.463	P	-6.88	chr14	33015531	A	G	643.1	0/1	0/0	1|0
2002	*AKAP6*	compound heterozygous	71.650153	nonsynonymous SNV	uc001wrq.3:c.G4564A:p.V1522I	0.02	rs34711402	0.001	B	2.82	chr14	33291583	G	A	1124.9	0/0	0/1	0|1
152	*AKAP9*	compound heterozygous	68.447747	nonsynonymous SNV	uc003uld.4:c.C139T:p.H47Y	8.09E-03	rs35669569	0	B	0.0934	chr7	91603115	C	T	1165	0/0	0/1	0|1
152	*AKAP9*	compound heterozygous	68.447747	nonsynonymous SNV	uc003uli.3:c.G2467C:p.E823Q	1.63E-05		0.884	D	3.25	chr7	91643622	G	C	644.4	0/1	0/0	1|0
2013	*AKNAD1*	homozygous rare	88.110403	nonsynonymous SNV	uc001dwa.3:c.C311T:p.A104V	0.01	rs17621411	0.068	B	-1.16	chr1	109394976	G	A	3744.6	0/1	0/1	1|1
3781	*ALDH4A1*	compound heterozygous	67.521821	nonsynonymous SNV	uc021ohl.1:c.C1430A:p.T477N	0.02	rs61757683	0.911	D	4.47	chr1	19199448	G	T	792.8	0/1	0/0	1|0
3781	*ALDH4A1*	compound heterozygous	67.521821	nonsynonymous SNV	uc001bbb.3:c.T1261C:p.C421R	1.62E-03	rs149414160	0.959	D	4.53	chr1	19202886	A	G	476	0/0	0/1	0|1
17	*ALG8*	compound heterozygous	79.464496	nonsynonymous SNV	uc001oyz.1:c.T1316C:p.I439T	0.02	rs17825668	0.002	B	3.06	chr11	77815059	A	G	674.3	0/0	0/1	0|1
17	*ALG8*	compound heterozygous	79.464496	nonsynonymous SNV	uc001oyz.1:c.G803A:p.R268Q	0.01	rs61995925	0.998	D	5.22	chr11	77823791	C	T	242.7	0/1	0/0	1|0
151	*AMPD1*	compound heterozygous	59.170795	nonsynonymous SNV	uc001eff.2:c.A1881T:p.L627F	1.55E-04	rs200717164	0.997	D	3.32	chr1	115217379	T	A	312.1	0/0	0/1	0|1
151	*AMPD1*	compound heterozygous	59.170795	nonsynonymous SNV	uc001eff.2:c.A947T:p.K316I	0.02	rs34526199	0.993	D	5.58	chr1	115222237	T	A	526.9	0/1	0/0	1|0
146	*AMZ1*	compound heterozygous	69.839585	nonsynonymous SNV	uc003smr.1:c.T680C:p.V227A	1.97E-03	rs150799683	0.001	B	-4.8	chr7	2748787	T	C	642.1	0/1	0/0	1|0
146	*AMZ1*	compound heterozygous	69.839585	nonsynonymous SNV	uc011jwa.1:c.C615A:p.S205R	3.05E-03	rs148177744	0.002	B	-4.11	chr7	2752383	C	A	686.9	0/0	0/1	0|1
2022	*ANKRD11*	compound heterozygous	0.0943619	nonsynonymous SNV	uc002fmx.1:c.C7519A:p.Q2507K	4.88E-05		0.98	D	5.1	chr16	89341551	G	T	791.9	0/0	0/1	0|1
2022	*ANKRD11*	compound heterozygous	0.0943619	nonsynonymous SNV	uc002fnb.1:c.C3848T:p.T1283M	6.91E-04	rs144673419	0.33	P	5.16	chr16	89348973	G	A	375.2	0/1	0/0	1|0
749	*ANKRD12*	compound heterozygous	45.364473	nonsynonymous SNV	uc010dkx.1:c.C713G:p.T238S	0.02	rs7243088	0	B	4.5	chr18	9254857	C	G	789.6	0/0	0/1	0|1
749	*ANKRD12*	compound heterozygous	45.364473	nonframeshift deletion	uc010dkx.1:c.1668_1670del:p.556_557del						chr18	9255811	ATGA	A	928.1	0/1	0/0	1|0
313	*ANKRD31*	homozygous rare	NA	nonframeshift deletion	uc003kdo.2:c.752_754del:p.251_252del						chr5	74491718	ATCA	A	1471.7	0/1	0/1	1|1
1346	*AOC2*	homozygous rare	44.102383	nonsynonymous SNV	uc002ibt.3:c.G818A:p.R273Q	7.37E-03	rs35508987	0.042	B	-0.175	chr17	40997461	G	A	3650.7	0/1	0/1	1|1
3781	*APBA3*	compound heterozygous	89.266336	nonsynonymous SNV	uc002lyp.1:c.C236T:p.A79V	6.04E-03	rs11556574	0.005	B	-1.69	chr19	3760027	G	A	731.2	0/1	0/0	1|0
3781	*APBA3*	compound heterozygous	89.266336	nonsynonymous SNV	uc002lyp.1:c.G48A:p.M16I	2.12E-04	rs200523433	0.04	B	4.5	chr19	3760215	C	T	535.3	0/0	0/1	0|1
2036	*APOBEC3H*	homozygous rare	85.527247	nonframeshift substitution	uc021wps.1:c.45_53GCGCCT:APOBEC3H						chr22	39496328	CAAGCGCCG	GCGCCT	3590.6	0/1	0/1	1|1
123	*APOBEC3H*	homozygous rare	85.527247	nonframeshift substitution	uc021wps.1:c.45_53GCGCCT:APOBEC3H						chr22	39496328	CAAGCGCCG	GCGCCT	4561.5	0/1	0/1	1|1
3721	*AQP1*	compound heterozygous	57.15381	nonsynonymous SNV	uc003tbv.2:c.G494A:p.G165D	0.02	rs28362731	0.017	B	3.49	chr7	30961790	G	A	648	0/0	0/1	0|1
3721	*AQP1*	compound heterozygous	57.15381	nonsynonymous SNV	uc003tbv.2:c.A778G:p.I260V			0.002	B	1.48	chr7	30963212	A	G	654.1	0/1	0/0	1|0
2036	*ARHGAP11A*	compound heterozygous	37.738854	nonsynonymous SNV	uc001zgw.3:c.A763G:p.I255V	0.02	rs71462813	0	B	2.62	chr15	32917735	A	G	3429.4	0/0	0/1	0|1
2036	*ARHGAP11A*	compound heterozygous	37.738854	nonsynonymous SNV	uc001zgw.3:c.G1228A:p.V410I	6.43E-03	rs61733064	0.325	P	1.73	chr15	32925302	G	A	2751.5	0/1	0/0	1|0
2001	*ARHGEF10L*	compound heterozygous	13.452465	nonsynonymous SNV	uc001bar.3:c.G1600A:p.G534R			0	B	2.44	chr1	17982383	G	A	1046.1	0/1	0/0	1|0
2001	*ARHGEF10L*	compound heterozygous	13.452465	nonsynonymous SNV	uc001bas.3:c.C614A:p.A205E			0.006	B	4.4	chr1	18023577	C	A	676.3	0/0	0/1	0|1
1346	*ARMC2*	compound heterozygous	19.933947	nonsynonymous SNV	uc011eao.2:c.G338A:p.R113H	1.22E-04	rs373656754				chr6	109220981	G	A	1556.1	0/1	0/0	1|0
1346	*ARMC2*	compound heterozygous	19.933947	nonsynonymous SNV	uc011eao.2:c.C1142T:p.T381M	0.02	rs61741720				chr6	109274276	C	T	1870.6	0/0	0/1	0|1
3721	*ARSF*	homozygous rare	74.663836	nonsynonymous SNV	uc004cre.2:c.G1276A:p.G426S			0.999	D	2.95	chrX	3028179	G	A	932	0/1	0/1	1|1
155	*ASB2*	compound heterozygous	27.02878	nonsynonymous SNV	uc001ycb.2:c.G746A:p.R249Q	0.01	rs35858830	0.077	B	1.2	chr14	94401102	C	T	502.1	0/0	0/1	0|1
155	*ASB2*	compound heterozygous	27.02878	nonsynonymous SNV	uc001ycc.2:c.A550G:p.N184D	0.02	rs11555542	0.56	P	5.16	chr14	94417531	T	C	120.1	0/1	0/0	1|0
8522	*ASXL2*	compound heterozygous	NA	nonsynonymous SNV	uc002rgs.2:c.A3179G:p.K1060R	3.27E-04	rs201921368				chr2	25966027	T	C	3472.6	0/1	0/0	1|0
8522	*ASXL2*	compound heterozygous	NA	nonsynonymous SNV	uc002rgs.2:c.A553G:p.S185G	2.07E-03	rs147191545				chr2	25991689	T	C	3744.1	0/0	0/1	0|1
28	*ATE1*	homozygous rare	22.505308	nonsynonymous SNV	uc010qts.2:c.C211T:p.L71F	0.01	rs148095496	0.001	B	1.57	chr10	123670505	G	A	2325.6	0/1	0/1	1|1
54	*ATF7IP2*	homozygous rare	59.105921	nonframeshift deletion	uc002czw.3:c.183_185del:p.61_62del						chr16	10524659	CGAC	C	2466.4	0/1	0/1	1|1
2037	*ATP1B4*	homozygous rare	65.758434	nonsynonymous SNV	uc011mty.2:c.T908C:p.V303A	4.88E-05	rs200352903	0.012	B	4.9	chrX	119513452	T	C	2404.7	0/1	0/1	1|1
151	*AX747619*	homozygous rare	NA	frameshift substitution	AX747619:uc002alq.1:exon1:c.183_183delinsAC		rs371991942				chr15	63340990	C	GT	1926.9	0/1	0/1	1|1
3781	*BACH1*	homozygous rare	30.820948	nonsynonymous SNV	uc002ynj.3:c.G1412C:p.G471A	0.01	rs35766001	0.937	D	1.38	chr21	30699557	G	C	2997.4	0/1	0/1	1|1
2035	*BAIAP3*	compound heterozygous	1.4685067	nonsynonymous SNV	uc002clj.3:c.G68A:p.R23H	0.01	rs11642118	0.998	D	3.17	chr16	1388618	G	A	531.1	0/1	0/0	1|0
2035	*BAIAP3*	compound heterozygous	1.4685067	nonsynonymous SNV	uc010uvc.1:c.G983C:p.G328A	1.49E-03	rs34598300	0.001	B	4.43	chr16	1392745	G	C	616.1	0/0	0/1	0|1
62	*BARD1*	compound heterozygous	89.861996	nonsynonymous SNV	uc021vwc.1:c.C142T:p.R48C	7.88E-03	rs3738888	0.23	P	5.42	chr2	215595164	G	A	602.5	0/0	0/1	0|1
62	*BARD1*	compound heterozygous	89.861996	nonframeshift deletion	uc021vwf.1:c.784_804del:p.262_268del	0.02	rs28997575				chr2	215645502	GTGGTGAAGAACATTCAGGCAA	G	1405.9	0/1	0/0	1|0
135	*BBS2*	compound heterozygous	11.364709	nonsynonymous SNV	uc002ejd.2:c.G2062A:p.G688R		rs376063241	0.996	D	5.14	chr16	56518777	C	T	535.6	0/0	0/1	0|1
135	*BBS2*	compound heterozygous	11.364709	frameshift insertion	uc002ejd.2:c.185_186insC:p.L62fs						chr16	56548525	A	AG	614.6	0/1	0/0	1|0
3731	*BBS2*	compound heterozygous	11.364709	nonsynonymous SNV	uc002ejd.2:c.C1511T:p.A504V	6.12E-03	rs16957538	0	B	2.91	chr16	56533706	G	A	1455.9	0/0	0/1	0|1
3731	*BBS2*	compound heterozygous	11.364709	nonsynonymous SNV	uc002ejd.2:c.A691G:p.K231E	1.63E-05		0.168	P	4.29	chr16	56540058	T	C	2175.3	0/1	0/0	1|0
123	*BC040901*	homozygous rare	NA	nonframeshift substitution	BC040901:uc003mle.3:exon2:c.276_277CT						chr5	179287585	GC	CT	3548.8	0/1	0/1	1|1
749	*BC040901*	homozygous rare	NA	nonframeshift substitution	BC040901:uc003mle.3:exon2:c.276_277CT						chr5	179287585	GC	CT	3396.4	0/1	0/1	1|1
53	*BOD1L*	compound heterozygous	NA	nonsynonymous SNV	uc003gmz.1:c.A7531C:p.T2511P	9.80E-03	rs61995953	0	B	0.722	chr4	13600993	T	G	652.9	0/0	0/1	0|1
53	*BOD1L*	compound heterozygous	NA	nonsynonymous SNV	uc003gmz.1:c.C2002A:p.Q668K	7.32E-05	rs370551969	0.931	D	5.36	chr4	13606522	G	T	1378.6	0/1	0/0	1|0
10	*C10orf28*	compound heterozygous	NA	nonsynonymous SNV	uc001kow.4:c.C1636T:p.P546S	0.02	rs35122894	0.07	B	-1.37	chr10	99969507	C	T	657.4	0/1	0/0	1|0
10	*C10orf28*	compound heterozygous	NA	nonsynonymous SNV	uc009xvy.3:c.C260T:p.A87V	3.66E-04	rs143935716	0.98	D	5.42	chr10	99995191	C	T	181.4	0/0	0/1	0|1
24	*C12orf74*	homozygous rare	81.381222	nonsynonymous SNV	uc021rbt.1:c.T488A:p.L163Q	0.02	rs71456571	0	B	-3.2	chr12	93100895	T	A	1364.9	0/1	0/1	1|1
2036	*C14orf37*	compound heterozygous	77.311866	nonsynonymous SNV	uc001xdd.3:c.C1624G:p.Q542E	7.81E-03	rs45570237	0.121	B	-1.21	chr14	58598437	G	C	1455.3	0/1	0/0	1|0
2036	*C14orf37*	compound heterozygous	77.311866	nonsynonymous SNV	uc001xdd.3:c.G1005C:p.E335D	0.02	rs61741193	0.738	P	0.902	chr14	58605072	C	G	2199.9	0/1	0/0	1|0
2028	*C14orf49*	compound heterozygous	NA	nonsynonymous SNV	uc001yei.4:c.C2159T:p.P720L	6.51E-05	rs140338587	0.016	B	-1.83	chr14	95906036	G	A	1306.9	0/0	0/1	0|1
2028	*C14orf49*	compound heterozygous	NA	nonsynonymous SNV	uc001yei.4:c.G2100T:p.K700N	8.55E-03	rs61754461	0.999	D	2.3	chr14	95906095	C	A	502.5	0/1	0/0	1|0
3737	*C14orf49*	homozygous rare	NA	nonsynonymous SNV	uc001yei.4:c.G274T:p.A92S	3.04E-03	rs35481769	0	B	0.413	chr14	95934175	C	A	1624.9	0/1	0/1	1|1
313	*C15orf42*	compound heterozygous	NA	nonsynonymous SNV	uc002boe.3:c.T2240C:p.V747A	0.01	rs12905387	0.889	D	4.14	chr15	90144615	T	C	697.1	0/0	0/1	0|1
313	*C15orf42*	compound heterozygous	NA	nonsynonymous SNV	uc002boe.3:c.C5579A:p.S1860Y			0.981	D	2.08	chr15	90169269	C	A	922	0/1	0/0	1|0
2037	*C16orf79*	deNovo	89.82661	nonsynonymous SNV	uc010bsh.3:c.T748C:p.C250R			0.999	D	5.72	chr16	2259398	A	G	440.7	0/0	0/0	0/1
2034	*C1QTNF5*	compound heterozygous	NA	nonsynonymous SNV	uc010rzg.1:c.G1148T:p.C383F	8.22E-03	rs145881139	0.453	P	1.08	chr11	119213319	C	A	1348.4	0/1	0/0	1|0
2034	*C1QTNF5*	compound heterozygous	NA	nonsynonymous SNV	uc010rzg.1:c.G1119A:p.M373I	3.66E-04	rs139725672	0	B	-1.17	chr11	119213348	C	T	2113.9	0/0	0/1	0|1
3721	*C2orf42*	compound heterozygous	69.45624	nonsynonymous SNV	uc002sgh.3:c.T1214C:p.I405T	5.69E-04	rs141556112	0	B	4.67	chr2	70392698	A	G	2116.7	0/0	0/1	0|1
3721	*C2orf42*	compound heterozygous	69.45624	nonsynonymous SNV	uc002sgh.3:c.G880T:p.A294S	1.81E-03	rs141350086	0	B	2.76	chr2	70406718	C	A	3023.1	0/1	0/0	1|0

Proband	Gene	Inheritance	Residual Variation Intolerance Score	Exonic Function	Amino Acid Change	Minor Allele Frequency EXAC	dbSNP138	PolyPhen2 Score	PolyPhen2 Prediction	GERP Score	Chromosome	Position	Ref	Alt	Quality Score	Paternal Genotype	Maternal Genotype	Proband Genotype
135	*C6orf132*	compound heterozygous	NA	nonsynonymous SNV	uc003orw.2:c.C2480T:p.P827L						chr6	42073170	G	A	420.8	0/1	0/0	1|0
135	*C6orf132*	compound heterozygous	NA	nonsynonymous SNV	uc003orw.2:c.T778G:p.S260A	0.02	rs11758539				chr6	42074872	A	C	486.7	0/0	0/1	0|1
2008	*C6orf186*	homozygous rare	NA	nonsynonymous SNV	uc003pub.2:c.A143G:p.H48R	0.02	rs41288584	0.977	D	1.91	chr6	110620177	T	C	3341	0/1	0/1	1|1
1131	*C8orf46*	homozygous rare	38.281434	nonsynonymous SNV	uc003xwg.3:c.G235T:p.A79S	0.02	rs61736270	0.015	B	0.125	chr8	67417718	G	T	834	0/1	0/1	1|1
10	*C8orf58*	compound heterozygous	87.137297	nonsynonymous SNV	uc003xce.3:c.C661T:p.P221S	7.55E-03	rs147403979	0.479	P	0.876	chr8	22459488	C	T	481.8	0/1	0/0	1|0
10	*C8orf58*	compound heterozygous	87.137297	nonsynonymous SNV	uc003xce.3:c.C931T:p.R311W	0.02	rs117289616	0.003	B	0.395	chr8	22460101	C	T	701.4	0/0	0/1	0|1
2026	*C9orf131*	compound heterozygous	82.944091	nonsynonymous SNV	uc003zvw.3:c.G45A:p.M15I	0.02	rs61742471	0.08	B	-3.07	chr9	35042296	G	A	2025.5	0/0	0/1	0|1
2026	*C9orf131*	compound heterozygous	82.944091	nonsynonymous SNV	uc003zvv.3:c.T1553C:p.I518T	0.01	rs34368082	0.065	B	-3.09	chr9	35044398	T	C	1265.4	0/1	0/0	1|0
151	*C9orf47*	homozygous rare	NA	nonsynonymous SNV	uc004aqc.2:c.G461A:p.S154N	0.01	rs34421923	0.012	B	1.52	chr9	91606812	G	A	3114.4	0/1	0/1	1|1
2010	*CACNA1C*	deNovo	1.5746638	nonsynonymous SNV	uc001qka.1:c.G1660A:p.V554I						chr12	2714851	G	A	1338.3	0/0	0/0	1|1
60	*CACNA2D4*	homozygous rare	89.661477	nonsynonymous SNV	uc021qsy.1:c.C266T:p.P89L	7.28E-03	rs61741336				chr12	1906632	G	A	1304.6	0/1	0/1	1|1
4405	*CAPN2*	homozygous rare	85.828025	nonsynonymous SNV	uc001hoc.3:c.C34T:p.P12S	6.03E-03	rs114318533				chr1	223946945	C	T	1531.2	0/1	0/1	1|1
3765	*CAPS2*	homozygous rare	67.303609	nonsynonymous SNV	uc001sxi.4:c.G418A:p.D140N	0.01	rs117753158	0.005	B	0.356	chr12	75687039	C	T	7175.2	0/1	0/1	1|1
23	*CCDC159*	homozygous rare	53.509082	frameshift substitution	CCDC159:uc010xlw.1:exon2:c.66_68AGCA						chr19	11459667	GCG	AGCA	2777.1	0/1	0/1	1|1
3721	*CCDC165*	compound heterozygous	NA	nonsynonymous SNV	uc002kns.2:c.A5G:p.E2G	8.13E-06		0.995	D	5.47	chr18	8796234	A	G	1579.2	0/1	0/0	1|0
3721	*CCDC165*	compound heterozygous	NA	nonsynonymous SNV	uc002kns.2:c.C2676G:p.H892Q	8.72E-04	rs146312067	0.998	D	-8.4	chr18	8826164	C	G	999.8	0/0	0/1	0|1
151	*CCDC168*	compound heterozygous	NA	nonsynonymous SNV	uc001vpm.3:c.A10790G:p.N3597S						chr13	103392257	T	C	428.7	0/1	0/0	1|0
151	*CCDC168*	compound heterozygous	NA	nonframeshift deletion	uc001vpm.3:c.8872_8874del:p.2958_2958del						chr13	103394172	CTTC	C	356.6	0/0	0/1	0|1
7952	*CCDC48*	compound heterozygous	NA	nonsynonymous SNV	uc011bkt.2:c.G1381T:p.A461S	1.22E-04	rs377018804	0.006	B	0.177	chr3	128753104	G	T	1108.5	0/0	0/1	0|1
7952	*CCDC48*	compound heterozygous	NA	nonsynonymous SNV	uc011bkt.2:c.G1662C:p.E554D	0.01	rs75140996	0.004	B	0.404	chr3	128758556	G	C	413.9	0/1	0/0	1|0
289	*CCDC88B*	compound heterozygous	71.083982	nonsynonymous SNV	uc001nzz.1:c.G1328A:p.R443Q	8.35E-03	rs143386417	0.695	P	2.15	chr11	64112394	G	A	714.3	0/0	0/1	0|1
289	*CCDC88B*	compound heterozygous	71.083982	nonsynonymous SNV	uc001oab.1:c.C502T:p.R168C			0.999	D	2.82	chr11	64121552	C	T	379.9	0/1	0/0	1|0
3761	*CCDC88B*	compound heterozygous	71.083982	nonsynonymous SNV	uc001oaa.3:c.C284T:p.T95I	0.01	rs144037797	0.053	B	-7.49	chr11	64117106	C	T	630.2	0/0	0/1	0|1
3761	*CCDC88B*	compound heterozygous	71.083982	nonsynonymous SNV	uc001oac.3:c.G317C:p.R106P	3.90E-04	rs202018582				chr11	64124605	G	C	315	0/1	0/0	1|0
2013	*CCNL2*	compound heterozygous	53.980892	nonsynonymous SNV	uc001afi.2:c.C212G:p.T71S	0.02	rs17851391	0.016	B	1.39	chr1	1334475	G	C	314.9	0/0	0/1	0|1
2013	*CCNL2*	compound heterozygous	53.980892	nonsynonymous SNV	uc001afi.2:c.C168G:p.D56E	0.01	rs114112990	0.001	B	-1.82	chr1	1334519	G	C	948.2	0/1	0/0	1|0
123	*CCNT1*	compound heterozygous	52.093654	nonsynonymous SNV	uc009zkz.2:c.G860A:p.R287H	5.53E-04	rs61751602	0.991	D	4.82	chr12	49087282	C	T	397.4	0/1	0/0	1|0
123	*CCNT1*	compound heterozygous	52.093654	nonsynonymous SNV	uc009zkz.2:c.A817G:p.T273A	0.02	rs61751603	0.001	B	1.09	chr12	49087325	T	C	526.1	0/0	0/1	0|1
2013	*CD1B*	homozygous rare	72.59967	nonsynonymous SNV	uc001frw.3:c.G409A:p.G137R	0.01	rs35841099	0.001	B	-0.749	chr1	158299840	C	T	2832.5	0/1	0/1	1|1
3731	*CD1E*	homozygous rare	60.56853	nonsynonymous SNV	uc001fry.3:c.A305G:p.H102R	5.13E-03	rs2873587	0.005	B	0.583	chr1	158324413	A	G	1620.8	0/1	0/1	1|1
50	*CD2BP2*	compound heterozygous	58.740269	nonsynonymous SNV	uc002dxr.3:c.C785T:p.T262I	0.01	rs34391305	0.913	D	3.62	chr16	30364712	G	A	1687.2	0/0	0/1	0|1
50	*CD2BP2*	compound heterozygous	58.740269	nonsynonymous SNV	uc002dxr.3:c.A262T:p.T88S	4.53E-03	rs139111996	0.995	D	4.93	chr16	30365335	T	A	1092	0/1	0/0	1|0
2007	*CD300LD*	compound heterozygous	72.22812	nonsynonymous SNV	uc002jkz.2:c.A325G:p.T109A	2.28E-04	rs180816540	0.002	B	3.83	chr17	72584704	T	C	3519.3	0/1	0/0	1|0
2007	*CD300LD*	compound heterozygous	72.22812	nonsynonymous SNV	uc002jkz.2:c.T142A:p.Y48N	6.35E-04	rs142719069	0.897	D	-8.42	chr17	72584887	A	T	1536.5	0/0	0/1	0|1
2023	*CD7*	homozygous rare	81.481481	nonsynonymous SNV	uc002kel.1:c.G686A:p.R229H	0.02	rs113733263	0.973	D	-0.364	chr17	80273234	C	T	2932.8	0/1	0/1	1|1
14	*CDH5*	homozygous rare	7.5784383	nonframeshift substitution	CDH5:uc002eom.4:exon10:c.1550_1551CT						chr16	66432423	TC	CT	1907.7	0/1	0/1	1|1
2001	*CDK5RAP2*	homozygous rare	1.8695447	nonsynonymous SNV	uc004bke.3:c.G920A:p.G307E	0.02	rs34523498	0.981	D	-0.892	chr9	123205981	C	T	26859.6	0/1	0/1	1|1
50	*CDKL5*	homozygous rare	15.864591	nonsynonymous SNV	uc022btn.1:c.C1303T:p.R435C	1.79E-04	rs61753977	0.999	D	4.1	chrX	18622374	C	T	1188.3	0/1	0/1	1|1
155	*CELSR2*	compound heterozygous	2.1762208	nonsynonymous SNV	uc001dxa.4:c.A3800G:p.H1267R	7.39E-03	rs138543788	0.998	D	4.4	chr1	109801543	A	G	1520.5	0/1	0/0	1|0
155	*CELSR2*	compound heterozygous	2.1762208	nonframeshift substitution	CELSR2:uc001dxa.4:exon16:c.6044_6045AA						chr1	109810200	GG	AA	852	0/0	0/1	0|1
2027	*CHD6*	compound heterozygous	8.9112998	nonsynonymous SNV	uc002xka.1:c.C7165T:p.R2389C	0.01	rs61752057	0.999	D	4.81	chr20	40040870	G	A	943.6	0/0	0/1	0|1
2027	*CHD6*	compound heterozygous	8.9112998	nonsynonymous SNV	uc002xka.1:c.G4301A:p.R1434K	6.91E-04	rs140327030	0.245	P	4.59	chr20	40053863	C	T	3242.3	0/1	0/0	1|0
7952	*CHRD*	compound heterozygous	15.363293	nonsynonymous SNV	uc011brr.2:c.C476T:p.A159V	5.29E-04	rs150401834	0.251	P	5.28	chr3	184102470	C	T	765.7	0/0	0/1	0|1
7952	*CHRD*	compound heterozygous	15.363293	nonsynonymous SNV	uc011brr.2:c.C1085T:p.T362I	2.36E-04	rs138569844	0.992	D	3.88	chr3	184105726	C	T	479.8	0/1	0/0	1|0
60	*CLDN16*	homozygous rare	43.571597	frameshift substitution	uc003fsi.3:c.165_166C:CLDN16						chr3	190106073	GG	C	3897.6	0/1	0/1	1|1
3731	*CLEC6A*	homozygous rare	89.956358	nonsynonymous SNV	uc001qum.1:c.G223C:p.A75P	1.30E-03	rs150852294	0.086	B	2.37	chr12	8612294	G	C	4063.9	0/1	0/1	1|1
2023	*CLECL1*	homozygous rare	83.138712	frameshift insertion	uc001qwi.3:c.153_154insAACTT:p.L51fs						chr12	9885708	T	TAAGTT	6907.5	0/1	0/1	1|1
2037	*CNOT4*	homozygous rare	57.313046	nonsynonymous SNV	uc003vss.3:c.C20G:p.A7G	0.02	rs17480616	0.001	B	0.995	chr7	135123060	G	C	6067.2	0/1	0/1	1|1
10	*CNR2*	homozygous rare	60.474168	nonframeshift substitution	uc021oij.1:c.188_189GG:CNR2						chr1	24201919	TT	CC	975.2	0/1	0/1	1|1
151	*CNR2*	homozygous rare	60.474168	nonframeshift substitution	uc021oij.1:c.188_189GG:CNR2						chr1	24201919	TT	CC	1185.1	0/1	0/1	1|1
17	*COBL*	homozygous rare	81.552253	nonframeshift deletion	uc003tpo.4:c.64_66del:p.22_22del						chr7	51098572	CTTC	C	1680.9	0/1	0/1	1|1
2007	*COL4A5*	homozygous rare	6.3163482	nonsynonymous SNV	uc004eob.1:c.C1039G:p.P347A	1.10E-03	rs104886164	0.745693	NA	1.75	chrX	107846262	C	G	1113.7	0/1	0/1	1|1
2812	*COL5A1*	compound heterozygous	0.5248879	nonsynonymous SNV	uc004cfe.3:c.G574A:p.D192N	0.02	rs138579182	0.32044	NA	3.29	chr9	137593099	G	A	1520.3	0/0	0/1	0|1
2812	*COL5A1*	compound heterozygous	0.5248879	nonsynonymous SNV	uc004cfe.3:c.G4322A:p.G1441E			0.787282	NA	4.34	chr9	137710593	G	A	588.9	0/1	0/0	1|0
2034	*COL6A2*	homozygous rare	8.2212786	nonsynonymous SNV	uc002zhy.1:c.C1552T:p.P518S	8.83E-03	rs141166141	0.998	D	4.23	chr21	47542052	C	T	3854.2	0/1	0/1	1|1
2007	*COL6A3*	compound heterozygous	0.8610521	nonsynonymous SNV	uc002vwj.2:c.G1291A:p.A431T	1.04E-03	rs114596320	0.391154	NA	-3.39	chr2	238243350	C	T	912.4	0/0	0/1	0|1
2007	*COL6A3*	compound heterozygous	0.8610521	nonsynonymous SNV	uc002vwr.3:c.T257C:p.V86A	1.27E-03	rs116794756	0.697623	NA	5.15	chr2	238289977	A	G	939.8	0/1	0/0	1|0
2033	*COL6A5*	homozygous rare	NA	nonsynonymous SNV	uc010htj.1:c.T167C:p.M56T	0.02	rs113396273				chr3	130095179	T	C	5685.2	0/1	0/1	1|1
2033	*COL6A6*	compound heterozygous	15.422269	nonsynonymous SNV	uc010htl.3:c.T3410C:p.I1137T	6.88E-03	rs200274210				chr3	130293232	T	C	966.4	0/1	0/0	1|0
2033	*COL6A6*	compound heterozygous	15.422269	nonsynonymous SNV	uc010htl.3:c.A4964G:p.Y1655C	0.01	rs141836516				chr3	130346185	A	G	5311.2	0/0	0/1	0|1
2002	*CPN2*	homozygous rare	71.620665	nonframeshift substitution	uc021xix.1:c.1525_1526TG:CPN2						chr3	194061906	TG	CA	1464	0/1	0/1	1|1
24	*CSF1R*	homozygous rare	82.955886	nonframeshift substitution	CSF1R:uc011dcf.2:exon5:c.956_957TT						chr5	149456771	CT	AA	5039.4	0/1	0/1	1|1
53	*CSF1R*	homozygous rare	82.955886	nonframeshift substitution	CSF1R:uc011dcf.2:exon5:c.956_957TT						chr5	149456771	CT	AA	4468.8	0/1	0/1	1|1
135	*CSF1R*	homozygous rare	82.955886	nonframeshift substitution	CSF1R:uc011dcf.2:exon5:c.956_957TT						chr5	149456771	CT	AA	4209.8	0/1	0/1	1|1
289	*CSF1R*	homozygous rare	82.955886	nonframeshift substitution	CSF1R:uc011dcf.2:exon5:c.956_957TT						chr5	149456771	CT	AA	4761.3	0/1	0/1	1|1
32	*CSF2RB*	compound heterozygous	7.3248408	nonsynonymous SNV	uc003aqc.4:c.G952A:p.D318N	0.01	rs117805308	0.027	B	1.43	chr22	37326794	G	A	487.8	0/0	0/1	0|1
32	*CSF2RB*	compound heterozygous	7.3248408	nonsynonymous SNV	uc003aqc.4:c.C1825A:p.P609T	0.02	rs1801122	0.999	D	3.67	chr22	37333657	C	A	413.9	0/1	0/0	1|0
2023	*CTAGE4*	homozygous rare	NA	nonsynonymous SNV	uc010lpe.3:c.T2077C:p.F693L		rs1635873				chr7	143964267	A	G	2148.4	0/1	0/1	1|1
66	*CYP2A6*	homozygous rare	71.644256	nonsynonymous SNV	uc002opl.4:c.G86A:p.S29N	0.02	rs28399435	0.024	B	-2.06	chr19	41356246	C	T	2688.9	0/1	0/1	1|1
3781	*DAAM2*	compound heterozygous	56.269167	nonsynonymous SNV	uc003oow.3:c.G515A:p.R172H	1.86E-03	rs200589550				chr6	39835372	G	A	798.9	0/1	0/0	1|0
3781	*DAAM2*	compound heterozygous	56.269167	nonsynonymous SNV	uc003oow.3:c.C1745A:p.P582H	1.34E-03	rs150676991				chr6	39847153	C	A	530.9	0/0	0/1	0|1
2013	*DAPK2*	homozygous rare	60.474168	nonsynonymous SNV	uc002amr.3:c.G179A:p.R60Q	4.13E-03	rs145090475	0.002	B	-9.41	chr15	64275867	C	T	1754	0/1	0/1	1|1
2034	*DCLK3*	compound heterozygous	52.25289	nonsynonymous SNV	uc003cgi.2:c.G1899T:p.E633D	0.01	rs35704209	0.949	D	4.29	chr3	36756867	C	A	3331.1	0/0	0/1	0|1
2034	*DCLK3*	compound heterozygous	52.25289	nonsynonymous SNV	uc003cgi.2:c.G71A:p.R24Q	6.34E-03	rs56070233	0.996	D	4.87	chr3	36780080	C	T	3385.7	0/1	0/0	1|0
3731	*DHX32*	compound heterozygous	21.726822	nonsynonymous SNV	uc001lje.1:c.C1000A:p.L334I		rs138137798	0.98	D	2.48	chr10	127525360	G	T	2729	0/0	0/1	0|1
3731	*DHX32*	compound heterozygous	21.726822	nonsynonymous SNV	uc001ljf.1:c.G108C:p.E36D	4.47E-04	rs139351747	0.054	B	3.34	chr10	127569286	C	G	1036.7	0/1	0/0	1|0
2020	*DHX34*	compound heterozygous	8.0030668	nonsynonymous SNV	uc010elc.1:c.C380T:p.A127V	3.74E-04	rs140291013	0.427539	NA	2.57	chr19	47856667	C	T	552.6	0/1	0/0	1|0
2020	*DHX34*	compound heterozygous	8.0030668	nonsynonymous SNV	uc010elc.1:c.C904T:p.R302W	2.36E-04	rs61757592	0.716289	NA	2.62	chr19	47858494	C	T	546.7	0/0	0/1	0|1
4405	*DISP2*	homozygous rare	78.957301	nonsynonymous SNV	uc001zlk.1:c.G341A:p.R114Q	0.02	rs35070171	0.058	B	0.796	chr15	40656047	G	A	1192.2	0/1	0/1	1|1
24	*DLGAP1*	compound heterozygous	3.0549658	nonsynonymous SNV	uc002kme.2:c.G1541A:p.R514Q	0.02	rs35822832	0.023	B	5.58	chr18	3534224	C	T	292.6	0/0	0/1	0|1
24	*DLGAP1*	compound heterozygous	3.0549658	nonsynonymous SNV	uc002kmk.2:c.C695T:p.A232V			0.001	B	5.08	chr18	3879374	G	A	401.4	0/1	0/0	1|0
2028	*DNAH2*	compound heterozygous	1.0851616	nonsynonymous SNV	uc002giu.1:c.G8281A:p.V2761M	0.02	rs35295135	0.051	B	5.38	chr17	7701525	G	A	398.8	0/0	0/1	0|1
2028	*DNAH2*	compound heterozygous	1.0851616	nonsynonymous SNV	uc002giu.1:c.C12974G:p.P4325R	0.02	rs116930996	0.999	D	4.36	chr17	7736242	C	G	4471.9	0/1	0/0	1|0
7952	*DNAH2*	compound heterozygous	1.0851616	nonsynonymous SNV	uc002giu.1:c.A890G:p.K297R	6.11E-03	rs144934136	0.024	B	5.06	chr17	7637938	A	G	1719.8	0/0	0/1	0|1
7952	*DNAH2*	compound heterozygous	1.0851616	nonsynonymous SNV	uc010cnm.1:c.A2998C:p.I1000L	0.02	rs79350244	0.236	P	5.37	chr17	7734114	A	C	1285.4	0/1	0/0	1|0
2026	*DNAH3*	compound heterozygous	3.7331918	nonsynonymous SNV	uc010vbd.2:c.G2771A:p.R924Q	0.02	rs111539520	0.008	B	0.412	chr16	20974740	C	T	1850.3	0/1	0/0	1|0
2026	*DNAH3*	compound heterozygous	3.7331918	nonsynonymous SNV	uc010vbd.2:c.G574C:p.E192Q	1.03E-03	rs34223805	0.985	D	4.3	chr16	20981303	C	G	827.9	0/0	0/1	0|1
2036	*DNAH3*	compound heterozygous	3.7331918	nonsynonymous SNV	uc010vbe.2:c.G2724C:p.R908S	5.99E-03	rs117470111	0.996	D	2.16	chr16	21098323	C	G	1459.3	0/1	0/0	1|0
2036	*DNAH3*	compound heterozygous	3.7331918	nonsynonymous SNV	uc010vbe.2:c.C1145T:p.P382L	9.69E-03	rs72780871	0.391	P	5.22	chr16	21139071	G	A	2581.7	0/0	0/1	0|1
1131	*DNAH3*	compound heterozygous	3.7331918	nonsynonymous SNV	uc010vbe.2:c.C3590T:p.P1197L	0.02	rs144617499	1	D	5.28	chr16	21073933	G	A	1802.7	0/0	0/1	0|1
1131	*DNAH3*	compound heterozygous	3.7331918	nonsynonymous SNV	uc002die.2:c.G1743T:p.K581N	3.08E-03	rs146094160	0	B	-1.98	chr16	21123123	C	A	2416.9	0/1	0/0	1|0
1131	*DNAH8*	compound heterozygous	NA	nonsynonymous SNV	uc003ooe.2:c.G1969A:p.V657M	0.02	rs45529837	0.016	B	5.57	chr6	38757615	G	A	5584.5	0/1	0/0	1|0
1131	*DNAH8*	compound heterozygous	NA	nonsynonymous SNV	uc003ooe.2:c.T7688C:p.I2563T	4.34E-03	rs142328376	0.967	D	5.44	chr6	38854646	T	C	2158.5	0/0	0/1	0|1
8522	*DNAH9*	compound heterozygous	78.060863	nonsynonymous SNV	uc002gne.3:c.A1810G:p.M604V	0.02	rs61740059	0.998	D	4.72	chr17	11543610	A	G	951.7	0/0	0/1	0|1
8522	*DNAH9*	compound heterozygous	78.060863	nonsynonymous SNV	uc010coo.3:c.G3667A:p.V1223M	5.45E-04	rs150023546	0.697	P	4.6	chr17	11631210	G	A	4348.5	0/1	0/0	1|0
1346	*DNAJC13*	compound heterozygous	65.770229	nonsynonymous SNV	uc003eor.3:c.C4543T:p.P1515S	0.02	rs55825559	0.183	P	5.37	chr3	132221139	C	T	4378.1	0/1	0/0	1|0
1346	*DNAJC13*	compound heterozygous	65.770229	nonsynonymous SNV	uc003eor.3:c.T6509G:p.L2170W	2.34E-03	rs140537885	0.913	D	5.1	chr3	132247160	T	G	1603.3	0/0	0/1	0|1
69	*DNAJC17*	homozygous rare	75.294881	nonsynonymous SNV	uc001zms.2:c.A350C:p.E117A	0.02	rs79709714	0.006	B	3.26	chr15	41068771	T	G	1160.1	0/1	0/1	1|1
155	*DNASE1L3*	homozygous rare	72.59967	nonsynonymous SNV	uc003djo.1:c.G244C:p.G82R	8.71E-03	rs74350392	0.001	B	2.91	chr3	58191274	C	G	1593.5	0/1	0/1	1|1
146	*DPH1*	compound heterozygous	76.356452	nonsynonymous SNV	uc002ftw.3:c.G125A:p.R42Q	0.01	rs61757361	0.004	B	-8.99	chr17	1943818	G	A	1744.5	0/0	0/1	0|1
146	*DPH1*	compound heterozygous	76.356452	nonsynonymous SNV	uc002ftw.3:c.C140T:p.S47F			0	B	3.6	chr17	1943833	C	T	1744.5	0/1	0/0	1|0
3781	*DPH1*	compound heterozygous	76.356452	nonsynonymous SNV	uc010cjx.3:c.C65T:p.T22I	6.07E-03	rs116911386	0.001	B	3.73	chr17	1939892	C	T	988.5	0/1	0/0	1|0
3781	*DPH1*	compound heterozygous	76.356452	nonsynonymous SNV	uc002ftw.3:c.G125A:p.R42Q	0.01	rs61757361	0.004	B	-8.99	chr17	1943818	G	A	865.7	0/0	0/1	0|1
7952	*DPYSL5*	homozygous rare	22.505308	nonsynonymous SNV	uc002rhu.4:c.G1534A:p.A512T	0.02	rs41288789	0.013	B	4.8	chr2	27167617	G	A	1579.8	0/1	0/1	1|1

Proband	Gene	Inheritance	Residual Variation Intolerance Score	Exonic Function	Amino Acid Change	Minor Allele Frequency EXAC	dbSNP138	PolyPhen2 Score	PolyPhen2 Prediction	GERP Score	Chromosome	Position	Ref	Alt	Quality Score	Paternal Genotype	Maternal Genotype	Proband Genotype
23	*DQ583205*	homozygous rare	NA	nonsynonymous SNV	uc010vph.1:c.C752T:p.P251L	0.02	rs61754918				chr16	89300425	C	T	882.5	0/1	0/1	1|1
2022	*DYNC2H1*	compound heterozygous	29.564756	nonsynonymous SNV	uc001phn.1:c.G8881A:p.A2961T	1.79E-03	rs199568537				chr11	103090692	G	A	2453.3	0/0	0/1	0|1
2022	*DYNC2H1*	compound heterozygous	29.564756	nonsynonymous SNV	uc009yxe.1:c.G2704C:p.G902R	0.01	rs144717489				chr11	103349922	G	C	3145.1	0/1	0/0	1|0
749	*DYNC2H1*	compound heterozygous	29.564756	nonsynonymous SNV	uc001phn.1:c.C4728G:p.N1576K	0.01	rs72989738				chr11	103036743	C	G	1108.4	0/0	0/1	0|1
749	*DYNC2H1*	compound heterozygous	29.564756	nonsynonymous SNV	uc009yxe.1:c.G2704C:p.G902R	0.01	rs144717489				chr11	103349922	G	C	2137.8	0/0	0/1	0|1
3781	*E2F1*	compound heterozygous	72.381458	nonsynonymous SNV	uc002wzu.4:c.G598A:p.G200S	0.02	rs35385772	0	B	-7.32	chr20	32266134	C	T	332.4	0/0	0/1	0|1
3781	*E2F1*	compound heterozygous	72.381458	nonsynonymous SNV	uc002wzu.4:c.G304A:p.A102T	4.66E-03	rs145741678	0.28	P	3.3	chr20	32268180	C	T	1411.8	0/1	0/0	1|0
8522	*EHBP1*	compound heterozygous	47.222222	nonsynonymous SNV	uc002scb.3:c.T802C:p.F268L	1.71E-04	rs140051312	0.687	P	4.23	chr2	63091910	T	C	3630.6	0/1	0/0	1|0
8522	*EHBP1*	compound heterozygous	47.222222	nonsynonymous SNV	uc002sbz.3:c.C2749G:p.L917V	9.84E-04	rs150215775	0.006	B	5.57	chr2	63220680	C	G	3255.6	0/0	0/1	0|1
2028	*EHMT1*	deNovo	3.1316348	nonsynonymous SNV	uc004cob.1:c.G779A:p.R260Q			0.999	D	5.07	chr9	140637871	G	A	1671.7	0/0	0/0	1|1
313	*EIF5A*	homozygous rare	49.386648	nonsynonymous SNV	uc010vtu.1:c.G367A:p.V123I	0.02	rs78916808				chr17	7213352	G	A	5532.8	0/1	0/1	1|1
123	*ELOVL5*	compound heterozygous	59.760557	nonsynonymous SNV	uc003pbs.2:c.G746A:p.C249Y	2.28E-04	rs146646780	0	B	5.61	chr6	53133954	C	T	696.1	0/0	0/1	0|1
123	*ELOVL5*	compound heterozygous	59.760557	stopgain SNV	uc003pbs.2:c.G736T:p.G246X		rs41273878				chr6	53133964	C	A	1043.3	0/1	0/0	1|0
8522	*ENOSF1*	homozygous rare	82.254069	nonsynonymous SNV	uc002kku.4:c.C93A:p.D31E	0.01	rs34724061	0.036	B	0.557	chr18	706570	G	T	2893.8	0/1	0/1	1|1
2023	*EPB41L3*	homozygous rare	89.325313	nonsynonymous SNV	uc010dkr.2:c.G230A:p.R77Q	4.25E-03	rs117538203	0.996	D	5.56	chr18	5419723	C	T	4532.4	0/1	0/1	1|1
2028	*EPG5*	compound heterozygous	28.314461	nonsynonymous SNV	uc002lbn.2:c.G1722C:p.E574D			0.003	B	2.22	chr18	43467728	C	G	2713.8	0/1	0/0	1|0
2028	*EPG5*	compound heterozygous	28.314461	nonsynonymous SNV	uc002lbm.3:c.A1766G:p.Q589R	0.01	rs61734402	0.855	D	4.83	chr18	43523997	T	C	4263.5	0/0	0/1	0|1
152	*ERC1*	homozygous rare	10.539042	nonsynonymous SNV	uc001qjf.2:c.A148G:p.S50G	0.02	rs35037408	0	B	-0.365	chr12	1137217	A	G	1442.1	0/1	0/1	1|1
66	*ERCC2*	compound heterozygous	1.8872376	nonsynonymous SNV	uc002pbk.2:c.A470C:p.K157T	3.25E-05		0.961	D	3.33	chr19	45868148	T	G	1284.7	0/0	0/1	0|1
66	*ERCC2*	compound heterozygous	1.8872376	nonsynonymous SNV	uc002pbi.2:c.T52G:p.F18V	0.01	rs111562095				chr19	45865046	A	C	748.9	0/1	0/0	1|0
2025	*FAM55C*	homozygous rare	NA	nonsynonymous SNV	uc003dvn.3:c.T1269G:p.H423Q	0.02	rs35598292	0.165	P	-2.72	chr3	101540387	T	G	4893.7	0/1	0/1	1|1
23	*FAM58BP*	homozygous rare	NA	nonframeshift deletion	uc009wzi.1:c.13_36del:p.5_12del		rs141962771				chr1	200182703	GGAGGACGCCGGAGGAGGAGGCCGGA	G	5751.1	0/1	0/1	1|1
2020	*FAM70B*	compound heterozygous	84.123614	nonsynonymous SNV	uc001vuh.3:c.C347T:p.P116L	2.03E-03	rs114871504	0.918	D	3.29	chr13	114502316	C	T	2281.7	0/1	0/0	1|0
2020	*FAM70B*	compound heterozygous	84.123614	nonsynonymous SNV	uc001vuh.3:c.G547A:p.V183I	0.02	rs41284482	0.995	D	4.34	chr13	114504663	G	A	423.6	0/0	0/1	0|1
2020	*FBLN2*	compound heterozygous	6.9886766	nonsynonymous SNV	uc011auz.2:c.G1009A:p.A337T	3.12E-03	rs111389908				chr3	13612786	G	A	423.3	0/1	0/0	1|0
2020	*FBLN2*	compound heterozygous	6.9886766	nonsynonymous SNV	uc011auz.2:c.G2221A:p.V741M	4.65E-04	rs200141144				chr3	13661319	G	A	2112.4	0/0	0/1	0|1
2020	*FBN1*	compound heterozygous	0.6428403	nonsynonymous SNV	uc001zwx.2:c.C7660T:p.R2554W	1.63E-05	rs369294972	0.995	D	4.48	chr15	48713794	G	A	3850.3	0/0	0/1	0|1
2020	*FBN1*	compound heterozygous	0.6428403	nonsynonymous SNV	uc001zwx.2:c.T986C:p.I329T	2.28E-03	rs12324002	0.003	B	5.68	chr15	48818329	A	G	1595.2	0/1	0/0	1|0
2026	*FBXL18*	homozygous rare	12.485256	nonsynonymous SNV	uc003son.4:c.T1656G:p.N552K	0.01	rs33941092	0.047	B	2.46	chr7	5540244	A	C	1059.6	0/1	0/1	1|1
135	*FBXL6*	compound heterozygous	24.534088	nonsynonymous SNV	uc003zbz.3:c.T338C:p.L113P	5.96E-03	rs72695499	0.989	D	4.21	chr8	145580028	A	G	727.2	0/0	0/1	0|1
135	*FBXL6*	compound heterozygous	24.534088	nonsynonymous SNV	uc010mfx.3:c.T35C:p.L12P			0.852	D	4.43	chr8	145580669	A	G	588.8	0/1	0/0	1|0
2026	*FBXO38*	compound heterozygous	10.503657	nonsynonymous SNV	uc003lpf.1:c.T1002A:p.D334E			0.99	D	2.93	chr5	147790237	T	A	4208.4	0/1	0/0	1|0
2026	*FBXO38*	compound heterozygous	10.503657	nonsynonymous SNV	uc003lpf.1:c.C2341T:p.P781S	1.06E-03	rs116266000	0.001	B	0.934	chr5	147807198	C	T	628.7	0/0	0/1	0|1
2023	*FBXO40*	compound heterozygous	12.57372	nonsynonymous SNV	uc003eeg.2:c.G367A:p.E123K	3.42E-04	rs34751480	0.854	D	4.96	chr3	121340643	G	A	2562.6	0/0	0/1	0|1
2023	*FBXO40*	compound heterozygous	12.57372	nonsynonymous SNV	uc003eeg.2:c.G1540A:p.A514T	1.71E-04	rs139593536	0.88	D	4.6	chr3	121341816	G	A	1266.9	0/1	0/0	1|0
3761	*FCHSD1*	compound heterozygous	68.536211	nonsynonymous SNV	uc010jgg.3:c.A1031C:p.D344A	0.02	rs116772138				chr5	141021294	T	G	1534.7	0/1	0/0	1|0
3761	*FCHSD1*	compound heterozygous	68.536211	nonsynonymous SNV	uc003llk.3:c.C1032A:p.N344K	0.02	rs3749760				chr5	141026182	G	T	865.5	0/0	0/1	0|1
3761	*FCN2*	deNovo	82.295353	nonsynonymous SNV	uc004cfh.1:c.G666A:p.M222I			0.006	B	-3.05	chr9	137779099	G	A	547.6	0/0	0/0	1|1
7952	*FGFBP3*	compound heterozygous	NA	nonsynonymous SNV	uc021pwg.1:c.G676C:p.D226H	0.01	rs80269321	0.871	D	0.648	chr10	93668051	C	G	754.4	0/1	0/0	1|0
7952	*FGFBP3*	compound heterozygous	NA	nonsynonymous SNV	uc021pwg.1:c.A587G:p.K196R	0.01	rs11186740	0.024	B	1.25	chr10	93668140	T	C	1746.1	0/0	0/1	0|1
1346	*FMN1*	homozygous rare	88.635291	nonsynonymous SNV	uc001zhf.4:c.C2048T:p.P683L	0.02	rs117804335				chr15	33261185	G	A	1691.5	0/1	0/1	1|1
3737	*FMN1*	deNovo	88.635291	nonsynonymous SNV	uc001zhf.4:c.C1439T:p.P480L						chr15	33357211	G	A	2091.7	0/0	0/0	1|1
45	*FNIP2*	compound heterozygous	45.647558	nonsynonymous SNV	uc003iqe.4:c.T901C:p.S301P	0.02	rs148251675				chr4	159780252	T	C	979.9	0/0	0/1	0|1
45	*FNIP2*	compound heterozygous	45.647558	nonsynonymous SNV	uc003iqe.4:c.C1653A:p.S551R	0.02	rs62001914				chr4	159789441	C	A	1073.8	0/1	0/0	1|0
120	*FNIP2*	compound heterozygous	45.647558	nonsynonymous SNV	uc003iqe.4:c.G1426T:p.A476S	0.01	rs62001915				chr4	159782889	G	T	544.3	0/1	0/0	1|0
120	*FNIP2*	compound heterozygous	45.647558	nonsynonymous SNV	uc003iqe.4:c.C1653A:p.S551R	0.02	rs62001914				chr4	159789441	C	A	421.6	0/0	0/1	0|1
14	*FREM2*	homozygous rare	16.772824	nonframeshift substitution	uc001uww.3:c.716_717GC:FREM2						chr13	39424253	CT	GC	1177.2	0/1	0/1	1|1
28	*FSCN2*	compound heterozygous	NA	nonsynonymous SNV	uc010wuo.2:c.G829T:p.V277F		rs181420326				chr17	79502080	G	T	497.7	0/1	0/0	1|0
28	*FSCN2*	compound heterozygous	NA	nonsynonymous SNV	uc010wuo.2:c.G967A:p.A323T	0.01	rs186367879				chr17	79502218	G	A	977.1	0/0	0/1	0|1
2035	*G2E3*	homozygous rare	75.294881	nonsynonymous SNV	uc001wql.1:c.G442A:p.A148T	5.73E-03	rs45475495	0.002	B	3.13	chr14	31085525	G	A	3273.8	0/1	0/1	1|1
2033	*GAA*	homozygous rare	6.0981364	nonsynonymous SNV	uc002jxp.3:c.G271A:p.D91N	0.02	rs1800299	0.999	D	4.7	chr17	78078656	G	A	2339.1	0/1	0/1	1|1
2007	*GAB3*	homozygous rare	50.011795	nonsynonymous SNV	uc004fml.1:c.G416C:p.S139T	7.42E-03	rs148492317	0.123	B	3.4	chrX	153908497	C	G	2937.4	0/1	0/1	1|1
3731	*GAL3ST1*	compound heterozygous	85.208776	nonsynonymous SNV	uc003aih.1:c.G119C:p.G40A	1.63E-05	rs200175330	0.016	B	3.68	chr22	30953261	C	G	708.1	0/1	0/0	1|0
3731	*GAL3ST1*	compound heterozygous	85.208776	nonsynonymous SNV	uc003aih.1:c.G100A:p.V34M	0.02	rs55674628	0.065	B	3.67	chr22	30953280	C	T	879.1	0/0	0/1	0|1
3731	*GHITM*	homozygous rare	45.128568	nonsynonymous SNV	uc010qmb.1:c.C353T:p.P118L	5.49E-03	rs11550184	0.012	B	4.76	chr10	85908565	C	T	6801.1	0/1	0/1	1|1
2027	*GLIS3*	homozygous rare	52.860344	nonsynonymous SNV	uc003zhy.1:c.C870A:p.D290E	0.02	rs148199056	0	B	2.56	chr9	4117942	G	T	2111.4	0/1	0/1	1|1
2034	*GMPPB*	compound heterozygous	30.555556	nonsynonymous SNV	uc003cxk.1:c.C869T:p.T290M	4.07E-05	rs139668958	0.28	P	4.52	chr3	49759480	G	A	568.2	0/0	0/1	0|1
2034	*GMPPB*	compound heterozygous	30.555556	nonsynonymous SNV	uc003cxk.1:c.C376G:p.H126D	0.01	rs34345884	0.001	B	4.59	chr3	49760431	G	C	1311.3	0/1	0/0	1|0
749	*GOLGB1*	homozygous rare	37.768342	nonsynonymous SNV	uc011bjm.1:c.C2200T:p.L734F	8.65E-03	rs114420009	0.45	P	4.84	chr3	121416813	G	A	4347.4	0/1	0/1	1|1
3721	*GPATCH3*	homozygous rare	19.798301	nonsynonymous SNV	uc001bne.3:c.A1463G:p.D488G	8.13E-06		0.992	D	4.68	chr1	27217616	T	C	1723.3	0/1	0/1	1|1
54	*GPNMB*	compound heterozygous	89.289927	nonsynonymous SNV	uc011jyz.2:c.C294G:p.N98K	1.73E-03	rs17147995	0.926	D	4.37	chr7	23299648	C	G	350.3	0/0	0/1	0|1
54	*GPNMB*	compound heterozygous	89.289927	stopgain SNV	uc011jyz.2:c.G1366T:p.E456X	9.08E-03	rs11537976	0.403943	NA	0.185	chr7	23313823	G	T	3031.4	0/1	0/0	1|0
28	*GPR111*	homozygous rare	NA	nonframeshift substitution	uc010jzj.1:c.1278_1279TG:GPR111						chr6	47649573	CA	TG	1376.1	0/1	0/1	1|1
289	*GPR111*	homozygous rare	NA	nonframeshift substitution	uc010jzj.1:c.1278_1279TG:GPR111						chr6	47649573	CA	TG	1548	0/1	0/1	1|1
2020	*GPR123*	compound heterozygous	43.772116	nonsynonymous SNV	uc001llw.3:c.G592A:p.A198T	0.02	rs11101757				chr10	134886558	G	A	825.8	0/0	0/1	0|1
2020	*GPR123*	compound heterozygous	43.772116	frameshift insertion	uc001llw.3:c.1667_1668insG:p.L556fs	2.17E-03	rs145811832				chr10	134902448	T	TG	438.7	0/1	0/0	1|0
45	*GPR123*	compound heterozygous	43.772116	nonsynonymous SNV	uc001llw.3:c.G592A:p.A198T	0.02	rs11101757				chr10	134886558	G	A	1634	0/0	0/1	0|1
45	*GPR123*	compound heterozygous	43.772116	frameshift insertion	uc001llw.3:c.1667_1668insG:p.L556fs	2.17E-03	rs145811832				chr10	134902448	T	TG	868.7	0/1	0/0	1|0
146	*GPX1*	homozygous rare	68.98443	unknown	UNKNOWN		rs71077799				chr3	49395690	CCGC	C	1988.5	0/1	0/1	1|1
7760	*GRHL2*	homozygous rare	26.7339	nonsynonymous SNV	uc010mbu.3:c.G1243A:p.V415I	0.01	rs3779617	0.955	D	5.5	chr8	102631911	G	A	9472	0/1	0/1	1|1
749	*GRHL3*	homozygous rare	81.086341	nonsynonymous SNV	uc001biz.3:c.T200C:p.V67A	0.02	rs34637004	0.01	B	4.02	chr1	24663184	T	C	5388.6	0/1	0/1	1|1
62	*GRIN3A*	homozygous rare	67.527719	nonframeshift substitution	uc004bbp.2:c.2502_2503CA:GRIN3A						chr9	104385711	CA	TG	1226.3	0/1	0/1	1|1
2033	*GTPBP5*	homozygous rare	81.068648	nonsynonymous SNV	uc011aaf.2:c.C536T:p.A179V	0.02	rs35693261	0.003	B	-1.97	chr20	60775922	C	T	3301.5	0/1	0/1	1|1
155	*GTSE1*	compound heterozygous	88.511441	nonsynonymous SNV	uc011aqz.2:c.C85T:p.R29W	6.51E-05	rs199691143	0.715	P	4.77	chr22	46704622	C	T	1419	0/1	0/0	1|0
155	*GTSE1*	compound heterozygous	88.511441	nonsynonymous SNV	uc003bhl.1:c.T17C:p.L6P	1.71E-04	rs142788611	0	B	-7.61	chr22	46712019	T	C	1017.5	0/0	0/1	0|1
7760	*GZF1*	homozygous rare	18.901864	nonsynonymous SNV	uc002wsz.3:c.A569G:p.N190S	9.86E-03	rs3810574	0.741	P	3.93	chr20	23345589	A	G	2187.6	0/1	0/1	1|1
135	*HABP2*	compound heterozygous	48.88535	nonsynonymous SNV	uc001lai.4:c.G95T:p.S32I	3.83E-03	rs61867369	0	B	1.07	chr10	115327273	G	T	251.8	0/1	0/0	1|0
135	*HABP2*	compound heterozygous	48.88535	nonsynonymous SNV	uc001lai.4:c.G1601A:p.G534E	0.02	rs7080536	0.998	D	5.58	chr10	115348046	G	A	706.4	0/0	0/1	0|1
10	*HAS3*	homozygous rare	19.863175	nonsynonymous SNV	uc002ewk.3:c.G518A:p.R173H	0.02	rs2232229	0	B	5.25	chr16	69143816	G	A	855	0/1	0/1	1|1
2027	*HK3*	compound heterozygous	19.550602	nonsynonymous SNV	uc003mez.3:c.G1295A:p.R432H	0.01	rs145827614	0	B	4.95	chr5	176308303	C	T	751.2	0/1	0/0	1|0
2027	*HK3*	compound heterozygous	19.550602	nonsynonymous SNV	uc003mfa.3:c.G737A:p.G246D	0.02	rs61749653	1	D	4.12	chr5	176316559	C	T	752.7	0/0	0/1	0|1
2026	*HOOK2*	compound heterozygous	72.753008	nonsynonymous SNV	uc002muz.2:c.G1963A:p.E655K	5.45E-03	rs189241336	0.989	D	5.11	chr19	12874383	C	T	1787.4	0/0	0/1	0|1
2026	*HOOK2*	compound heterozygous	72.753008	nonsynonymous SNV	uc002muy.2:c.A587G:p.D196G	7.68E-04	rs200086187	0.969	D	4.56	chr19	12882224	T	C	1654.9	0/1	0/0	1|0
45	*HS3ST6*	homozygous rare	NA	nonframeshift substitution	HS3ST6:uc002cnf.3:exon2:c.480_481TA						chr16	1962046	CG	TA	3352.8	0/1	0/1	1|1
66	*HS3ST6*	homozygous rare	NA	nonframeshift substitution	HS3ST6:uc002cnf.3:exon2:c.480_481TA						chr16	1962046	CG	TA	1746.2	0/1	0/1	1|1
66	*IDO2*	homozygous rare	NA	nonsynonymous SNV	uc010lwy.1:c.T754A:p.S252T	0.02	rs35212142				chr8	39862893	T	A	851.2	0/1	0/1	1|1
8522	*IFT140*	compound heterozygous	22.823779	nonsynonymous SNV	uc002clz.3:c.G3193A:p.E1065K	4.07E-05	rs142106374	0.615491	NA	3.85	chr16	1560980	C	T	732.4	0/0	0/1	0|1
8522	*IFT140*	compound heterozygous	22.823779	nonsynonymous SNV	uc002cmb.3:c.G322A:p.V108M	6.86E-03	rs146128830	0.03	B	-1.38	chr16	1652418	C	T	1146.8	0/1	0/0	1|0
2001	*INPP5F*	compound heterozygous	12.57372	nonsynonymous SNV	uc001leo.3:c.G702A:p.M234I	6.23E-03	rs34239637	0.001	B	-1.1	chr10	121556259	G	A	3882.6	0/1	0/0	1|0
2001	*INPP5F*	compound heterozygous	12.57372	nonsynonymous SNV	uc001leo.3:c.T872C:p.M291T	1.77E-03	rs145230512	0.756	P	5.51	chr10	121556976	T	C	10564.9	0/0	0/1	0|1
749	*INPP5F*	compound heterozygous	12.57372	nonsynonymous SNV	uc001leo.3:c.G702A:p.M234I	6.23E-03	rs34239637	0.001	B	-1.1	chr10	121556259	G	A	891.8	0/1	0/0	1|0
749	*INPP5F*	compound heterozygous	12.57372	nonsynonymous SNV	uc001lep.3:c.G1320C:p.E440D	4.90E-03	rs61757087	0.101	B	4.38	chr10	121587043	G	C	1206.1	0/0	0/1	0|1
2022	*IP6K3*	homozygous rare	85.763152	nonframeshift substitution	uc003ofb.2:c.933_934CA:IP6K3						chr6	33690796	CA	TG	5820.3	0/1	0/1	1|1
2033	*IP6K3*	homozygous rare	85.763152	nonframeshift substitution	uc003ofb.2:c.933_934CA:IP6K3						chr6	33690796	CA	TG	5691.4	0/1	0/1	1|1
23	*IP6K3*	homozygous rare	85.763152	nonframeshift substitution	uc003ofb.2:c.933_934CA:IP6K3						chr6	33690796	CA	TG	1294.1	0/1	0/1	1|1
62	*IP6K3*	homozygous rare	85.763152	nonframeshift substitution	uc003ofb.2:c.933_934CA:IP6K3						chr6	33690796	CA	TG	1585.2	0/1	0/1	1|1
120	*ITSN2*	homozygous rare	21.420146	nonsynonymous SNV	uc002rff.2:c.C3928T:p.R1310W	0.02	rs41281481	1	D	4.42	chr2	24435599	G	A	805.4	0/1	0/1	1|1
135	*JMJD1C*	compound heterozygous	19.810097	nonsynonymous SNV	uc009xpk.1:c.C2633T:p.P878L	0.02	rs41274064	0.008	B	4.09	chr10	64952835	G	A	709.4	0/0	0/1	0|1
135	*JMJD1C*	compound heterozygous	19.810097	nonsynonymous SNV	uc009xpk.1:c.G1459T:p.V487F			0.45	P	5.39	chr10	64967081	C	A	1071.9	0/1	0/0	1|0
28	*JPH1*	compound heterozygous	60.645199	nonsynonymous SNV	uc003yae.3:c.G1870C:p.D624H	0.01	rs16938828	0.028	B	2.12	chr8	75156799	C	G	1064.3	0/1	0/0	1|0
28	*JPH1*	compound heterozygous	60.645199	nonsynonymous SNV	uc003yae.3:c.C1520T:p.T507M	1.85E-03	rs16938829	0.021	B	-3.2	chr8	75157149	G	A	673	0/0	0/1	0|1
3731	*KCNJ14*	deNovo	48.543289	nonsynonymous SNV	uc002pjf.1:c.A844C:p.S282R			1	D	5.05	chr19	48967567	A	C	306.1	0/0	0/0	0/1
2001	*KCNJ3*	deNovo	54.948101	nonsynonymous SNV	uc002tyv.1:c.A322G:p.T108A	3.25E-05		0.001	B	2.35	chr2	155555609	A	G	6034.7	0/0	0/0	0/1
7952	*KCNQ2*	homozygous rare	15.864591	nonsynonymous SNV	uc002yfb.1:c.G1264A:p.V422M	1.27E-03	rs146492238	0	B	2.33	chr20	62046433	C	T	1482.7	0/1	0/1	1|1

Proband	Gene	Inheritance	Residual Variation Intolerance Score	Exonic Function	Amino Acid Change	Minor Allele Frequency EXAC	dbSNP138	PolyPhen2 Score	PolyPhen2 Prediction	GERP Score	Chromosome	Position	Ref	Alt	Quality Score	Paternal Genotype	Maternal Genotype	Proband Genotype
4405	*KIAA0100*	compound heterozygous	73.425336	nonsynonymous SNV	uc002hbu.3:c.G2852A:p.R951H	3.80E-03	rs61732688	0.998	D	5.55	chr17	26961753	C	T	1356	0/0	0/1	0|1
4405	*KIAA0100*	compound heterozygous	73.425336	nonsynonymous SNV	uc010crr.2:c.G321C:p.K107N			0.001	B	-4.36	chr17	26968923	C	G	916.8	0/1	0/0	1|0
2034	*KIAA0556*	homozygous rare	88.133994	nonsynonymous SNV	uc002dow.3:c.G4276A:p.E1426K	0.02	rs117316062	0.98	D	4.42	chr16	27784497	G	A	10634.5	0/1	0/1	1|1
32	*KIAA0564*	compound heterozygous	10.509554	nonsynonymous SNV	uc001uyj.3:c.G4690A:p.V1564M	4.57E-03	rs73464952	0.875	D	5.73	chr13	42189142	C	T	479.8	0/0	0/1	0|1
32	*KIAA0564*	compound heterozygous	10.509554	nonsynonymous SNV	uc001uyj.3:c.G2693A:p.R898K	8.67E-03	rs41288291	0.727	P	5.25	chr13	42301395	C	T	1848.6	0/1	0/0	1|0
3721	*KIAA0564*	compound heterozygous	10.509554	nonsynonymous SNV	uc001uyj.3:c.A2795G:p.K932R	3.25E-05		0.349	P	5.27	chr13	42295675	T	C	2222.4	0/1	0/0	1|0
3721	*KIAA0564*	compound heterozygous	10.509554	nonsynonymous SNV	uc001uyj.3:c.T2783C:p.V928A	3.33E-04	rs138978193	0.305	P	5.27	chr13	42295687	A	G	2278.6	0/0	0/1	0|1
155	*KIAA0664*	compound heterozygous	1.3328615	nonsynonymous SNV	uc002fux.1:c.G2233A:p.G745S	5.72E-05	rs370270067				chr17	2598568	C	T	1668.4	0/0	0/1	0|1
155	*KIAA0664*	compound heterozygous	1.3328615	nonsynonymous SNV	uc002fux.1:c.G1567A:p.A523T	6.92E-03	rs145919520				chr17	2601266	C	T	461.9	0/1	0/0	1|0
146	*KIAA0754*	compound heterozygous	NA	nonsynonymous SNV	uc009vvt.1:c.A636C:p.L212F	9.01E-03	rs74607331				chr1	39876573	A	C	2048.8	0/0	0/1	0|1
146	*KIAA0754*	compound heterozygous	NA	nonsynonymous SNV	uc009vvt.1:c.A4082C:p.E1361A	4.09E-03	rs78342613				chr1	39880019	A	C	414.7	0/1	0/0	1|0
2013	*KIAA0889*	compound heterozygous	NA	nonsynonymous SNV	uc002xgd.1:c.C1625T:p.P542L	3.82E-03	rs145513467				chr20	35441225	G	A	1140.1	0/1	0/0	1|0
2013	*KIAA0889*	compound heterozygous	NA	nonsynonymous SNV	uc002xgd.1:c.G1441C:p.V481L	2.44E-05					chr20	35443690	C	G	703.5	0/0	0/1	0|1
2022	*KIAA1797*	compound heterozygous	NA	nonsynonymous SNV	uc003zoh.1:c.C644T:p.T215I	1.28E-03	rs147376982	0.254	P	0.566	chr9	20881888	C	T	2203.9	0/0	0/1	0|1
2022	*KIAA1797*	compound heterozygous	NA	nonsynonymous SNV	uc003zoh.1:c.G3355A:p.A1119T	7.97E-03	rs150147497	0	B	4.24	chr9	20990164	G	A	1268.9	0/1	0/0	1|0
3721	*KIAA1797*	compound heterozygous	NA	nonsynonymous SNV	uc003zoh.1:c.A122C:p.D41A	8.14E-06		1	D	5	chr9	20823008	A	C	2080.7	0/0	0/1	0|1
3721	*KIAA1797*	compound heterozygous	NA	nonsynonymous SNV	uc003zoh.1:c.A554T:p.D185V			0.008	B	4.7	chr9	20874735	A	T	2572.6	0/1	0/0	1|0
2008	*KIF13A*	compound heterozygous	46.496815	nonsynonymous SNV	uc003nce.2:c.C516G:p.D172E	6.66E-03	rs41267712				chr6	17764896	G	C	2247.4	0/1	0/0	1|0
2008	*KIF13A*	compound heterozygous	46.496815	nonsynonymous SNV	uc003nce.2:c.A1G:p.M1V	0.02	rs17689215				chr6	17773790	T	C	3984.1	0/0	0/1	0|1
2013	*KIF20A*	compound heterozygous	12.532437	nonsynonymous SNV	uc003lcj.3:c.C131T:p.S44F	2.86E-03	rs150704301	0.995	D	4.05	chr5	137515500	C	T	1234.7	0/1	0/0	1|0
2013	*KIF20A*	compound heterozygous	12.532437	nonsynonymous SNV	uc003lcj.3:c.C2516T:p.P839L	0.01	rs3172747	0.303	P	5.48	chr5	137522945	C	T	4046.7	0/0	0/1	0|1
45	*KLHL38*	homozygous rare	80.372729	nonframeshift substitution	KLHL38:uc003yqs.1:exon1:c.293_294GC						chr8	124664873	AT	GC	2252	0/1	0/1	1|1
123	*KLHL38*	homozygous rare	80.372729	nonframeshift substitution	KLHL38:uc003yqs.1:exon1:c.293_294GC						chr8	124664873	AT	GC	1309.5	0/1	0/1	1|1
2026	*KLK3*	homozygous rare	67.356688	nonsynonymous SNV	uc002pts.1:c.G304A:p.D102N	0.02	rs61752561	0	B	-2.85	chr19	51361382	G	A	5170.2	0/1	0/1	1|1
17	*KLK3*	homozygous rare	67.356688	nonsynonymous SNV	uc002pts.1:c.G304A:p.D102N	0.02	rs61752561	0	B	-2.85	chr19	51361382	G	A	2478.1	0/1	0/1	1|1
2010	*KRT28*	compound heterozygous	87.585515	nonsynonymous SNV	uc002hvh.1:c.A1370G:p.K457R	2.44E-05		0.769	P	5.66	chr17	38948704	T	C	5084.1	0/1	0/0	1|0
2010	*KRT28*	compound heterozygous	87.585515	nonsynonymous SNV	uc002hvh.1:c.G565C:p.V189L	3.42E-03	rs139807956	0.01	B	3.26	chr17	38954612	C	G	3384.3	0/0	0/1	0|1
152	*KRT6B*	homozygous rare	32.248172	nonsynonymous SNV	uc001sak.3:c.G1495A:p.G499S	0.02	rs61746355	0.174644	NA	-0.274	chr12	52841174	C	T	3452.3	0/1	0/1	1|1
3731	*KRTAP17-1*	homozygous rare	67.716443	nonsynonymous SNV	uc002hwj.3:c.G140A:p.G47D	1.34E-03	rs74252500	0.414147	NA	3.36	chr17	39471763	C	T	735.9	0/1	0/1	1|1
2007	*KRTAP19-6*	homozygous rare	80.455296	frameshift substitution	KRTAP19-6:uc002yok.1:exon1:c.153_171TTATGGATTCTCTGGATT						chr21	31913982	GAATCCAGAGAATCCATAT	AATCCAGAGAATCCATAA	5664.2	0/1	0/1	1|1
17	*KRTAP19-6*	homozygous rare	80.455296	frameshift substitution	KRTAP19-6:uc002yok.1:exon1:c.153_171TTATGGATTCTCTGGATT						chr21	31913982	GAATCCAGAGAATCCATAT	AATCCAGAGAATCCATAA	6149.8	0/1	0/1	1|1
54	*KRTAP19-6*	homozygous rare	80.455296	frameshift substitution	KRTAP19-6:uc002yok.1:exon1:c.153_171TTATGGATTCTCTGGATT						chr21	31913982	GAATCCAGAGAATCCATAT	AATCCAGAGAATCCATAA	5995	0/1	0/1	1|1
2001	*LAIR2*	homozygous rare	75.12385	stopgain SNV	uc002qgc.3:c.C226T:p.R76X	0.02	rs61737751	0.534453	NA	0.692	chr19	55019261	C	T	15219.2	0/1	0/1	1|1
3731	*LAMA1*	homozygous rare	86.217268	nonsynonymous SNV	uc010wzj.2:c.C5959A:p.L1987M	4.71E-03	rs60009920	0.001	B	3.1	chr18	6961680	G	T	2946.2	0/1	0/1	1|1
155	*LAMA2*	compound heterozygous	79.641425	nonsynonymous SNV	uc003qbn.3:c.G922A:p.E308K	1.15E-03	rs146462599	0.620829	NA	5.57	chr6	129470136	G	A	794.2	0/1	0/0	1|0
155	*LAMA2*	compound heterozygous	79.641425	nonsynonymous SNV	uc003qbn.3:c.G4750A:p.G1584S	0.02	rs117781224	0.348762	NA	3.7	chr6	129687396	G	A	980.7	0/1	0/0	1|0
2812	*LAMB1*	compound heterozygous	8.5456476	nonsynonymous SNV	uc003vev.2:c.G2941A:p.D981N	0.01	rs61751041	0	B	2.75	chr7	107594185	C	T	1171.2	0/1	0/0	1|0
2812	*LAMB1*	compound heterozygous	8.5456476	nonsynonymous SNV	uc003vev.2:c.C2455G:p.R819G	0.01	rs80095409	0.999	D	4.96	chr7	107600211	G	C	382.3	0/0	0/1	0|1
53	*LARS*	homozygous rare	24.675631	nonframeshift substitution	uc003lnw.1:c.147_148GG:LARS						chr5	145508636	TA	CC	1338.3	0/1	0/1	1|1
146	*LEPRE1*	compound heterozygous	74.681529	nonsynonymous SNV	uc001chx.4:c.G2248A:p.G750R	5.05E-03	rs116577636	0.276323	NA	-1.8	chr1	43212750	C	T	1216.5	0/0	0/1	0|1
146	*LEPRE1*	compound heterozygous	74.681529				rs41269515				chr1	43217876	C	T	351.7	0/1	0/0	1|0
28	*LETM1*	homozygous rare	81.086341	nonsynonymous SNV	uc003gdv.3:c.G1496A:p.R499H	0.02	rs28681468	0.005	B	-1.51	chr4	1824020	C	T	919.2	0/1	0/1	1|1
123	*LGI4*	homozygous rare	53.851144	nonframeshift substitution	uc002nxx.2:c.1395_1396AT:LGI4						chr19	35616315	GC	AT	2156.4	0/1	0/1	1|1
2033	*LMTK2*	compound heterozygous	1.0497759	nonsynonymous SNV	uc003upd.2:c.G2584A:p.A862T	3.74E-03	rs34005293	0	B	-6.27	chr7	97822361	G	A	1495.8	0/1	0/0	1|0
2033	*LMTK2*	compound heterozygous	1.0497759	nonsynonymous SNV	uc003upd.2:c.C4022G:p.A1341G	5.07E-03	rs56343792	0	B	1.01	chr7	97823799	C	G	845.7	0/0	0/1	0|1
7760	*LOC285033*	homozygous rare	NA	nonsynonymous SNV	uc002svp.1:c.G298C:p.V100L	1.17E-03	rs116858801				chr2	96906359	G	C	1278	0/1	0/1	1|1
2027	*LOC286238*	homozygous rare	NA	nonframeshift substitution	LOC286238:uc010mql.1:exon2:c.149_150AG						chr9	91262493	AG	CT	5610.8	0/1	0/1	1|1
66	*LOC646508*	homozygous rare	NA	nonsynonymous SNV	uc021uze.1:c.G341A:p.S114N	0.01	rs201240639				chr19	53786079	G	A	2457.4	0/1	0/1	1|1
66	*LOC646508*	homozygous rare	NA	nonframeshift substitution	LOC646508:uc021uze.1:exon3:c.343_344AG						chr19	53786081	GA	AG	2457.4	0/1	0/1	1|1
146	*LOC646508*	homozygous rare	NA	nonsynonymous SNV	uc021uze.1:c.G341A:p.S114N	0.01	rs201240639				chr19	53786079	G	A	1522.9	0/1	0/1	1|1
146	*LOC646508*	homozygous rare	NA	nonframeshift substitution	LOC646508:uc021uze.1:exon3:c.343_344AG						chr19	53786081	GA	AG	1522.9	0/1	0/1	1|1
45	*LRRC16B*	compound heterozygous	16.153574	nonsynonymous SNV	uc001wlk.2:c.G287A:p.R96H	0.02	rs117092113	0.515007	NA	4.81	chr14	24533474	G	A	580.9	0/1	0/0	1|0
45	*LRRC16B*	compound heterozygous	16.153574	nonsynonymous SNV	uc001wlk.2:c.G391A:p.V131M	7.34E-05		0.304735	NA	-5.33	chr14	24534189	G	A	2317.5	0/0	0/1	0|1
50	*LRRC3C*	homozygous rare	NA	nonsynonymous SNV	uc021twv.1:c.C514G:p.L172V	0.02	rs72832968				chr17	38100673	C	G	2685.8	0/1	0/1	1|1
17	*LRRK1*	compound heterozygous	2.9723992	nonsynonymous SNV	uc002bwr.3:c.C1246A:p.L416M	8.54E-03	rs55739947	0.724424	NA	1.16	chr15	101551007	C	A	316.4	0/1	0/0	1|0
17	*LRRK1*	compound heterozygous	2.9723992	nonframeshift substitution	LRRK1:uc002bwr.3:exon33:c.5813_5814AA						chr15	101606889	GC	AA	379.1	0/0	0/1	0|1
69	*LRRK1*	homozygous rare	2.9723992	nonframeshift substitution	LRRK1:uc002bwr.3:exon33:c.5813_5814AA						chr15	101606889	GC	AA	2232	0/1	0/1	1|1
3781	*LRRK1*	homozygous rare	2.9723992	nonframeshift substitution	LRRK1:uc002bwr.3:exon33:c.5813_5814AA						chr15	101606889	GC	AA	5947.2	0/1	0/1	1|1
151	*LSG1*	homozygous rare	77.311866	nonframeshift substitution	LSG1:uc003fui.3:exon8:c.798_799TG						chr3	194373832	TG	CA	3953.8	0/1	0/1	1|1
2033	*LYSMD3*	homozygous rare	58.527955	nonsynonymous SNV	uc003kjr.3:c.C449A:p.S150Y	0.01	rs62375061	0.636	P	-0.634	chr5	89815108	G	T	7048.9	0/1	0/1	1|1
2020	*LYST*	compound heterozygous	0.4953999	nonsynonymous SNV	uc001hxi.2:c.T585G:p.N195K	2.19E-03	rs34702903	0.676	P	1.72	chr1	235894366	A	C	6068	0/0	0/1	0|1
2020	*LYST*	compound heterozygous	0.4953999	nonsynonymous SNV	uc001hxj.2:c.A3217G:p.I1073V	1.79E-04	rs151130915	0	B	-3.57	chr1	235969219	T	C	2396.3	0/1	0/0	1|0
2812	*MACF1*	compound heterozygous	0.2064166	nonsynonymous SNV	uc001cde.2:c.T873G:p.F291L	2.47E-03	rs138819868	0.760955	NA	4.31	chr1	39951304	T	G	1165	0/0	0/1	0|1
2812	*MACF1*	compound heterozygous	0.2064166	nonsynonymous SNV	uc021olw.1:c.T2006C:p.M669T	5.43E-03	rs41270805	0	B	-0.0377	chr1	39798946	T	C	1037.3	0/1	0/0	1|0
3737	*MACF1*	compound heterozygous	0.2064166	nonsynonymous SNV	uc001cde.2:c.T873G:p.F291L	2.47E-03	rs138819868	0.760955	NA	4.31	chr1	39951304	T	G	924.4	0/1	0/0	1|0
3737	*MACF1*	compound heterozygous	0.2064166	nonsynonymous SNV	uc021olw.1:c.A5228C:p.K1743T	0.02	rs17570718	0.028	B	-1.81	chr1	39802168	A	C	630.3	0/0	0/1	0|1
2035	*MAGI3*	homozygous rare	9.7546591	nonsynonymous SNV	uc001edk.3:c.G3953A:p.G1318D	0.02	rs61742849				chr1	114226143	G	A	4192.8	0/1	0/1	1|1
2036	*MAN2C1*	homozygous rare	46.207832	nonsynonymous SNV	uc010umi.1:c.C1274T:p.P425L	0.02	rs78610801	0.002	B	3.37	chr15	75651981	G	A	3148.2	0/1	0/1	1|1
749	*MAP1B*	compound heterozygous	1.3505544	nonsynonymous SNV	uc010iyy.1:c.G2008A:p.A670T	0.01	rs35473133	0.613493	NA	4.33	chr5	71491568	G	A	710.1	0/0	0/1	0|1
749	*MAP1B*	compound heterozygous	1.3505544	nonsynonymous SNV	uc010iyy.1:c.C5024T:p.S1675L	5.69E-05	rs368052791	0.635343	NA	5.22	chr5	71494584	C	T	1933.5	0/1	0/0	1|0
2008	*MAST4*	compound heterozygous	37.703468	nonsynonymous SNV	uc003juw.3:c.G4600A:p.V1534I	0.02	rs17221458				chr5	66460390	G	A	2251.1	0/0	0/1	0|1
2008	*MAST4*	compound heterozygous	37.703468	nonsynonymous SNV	uc003juw.3:c.C5573T:p.P1858L	0.02	rs55882716				chr5	66461363	C	T	915.9	0/1	0/0	1|0
2026	*MATN3*	homozygous rare	79.464496	nonsynonymous SNV	uc002rdl.3:c.G754A:p.E252K	0.02	rs52826764				chr2	20205541	C	T	2000.9	0/1	0/1	1|1
2010	*MCC*	compound heterozygous	4.0398679	nonsynonymous SNV	uc003kqj.4:c.G2188C:p.E730Q	1.63E-05		0.956	D	5.28	chr5	112364752	C	G	2169	0/0	0/1	0|1
2010	*MCC*	compound heterozygous	4.0398679	nonsynonymous SNV	uc003kqj.4:c.C1457T:p.P486L	0.01	rs35269015	0.011	B	3.53	chr5	112403779	G	A	850.6	0/1	0/0	1|0
3721	*MCTP2*	homozygous rare	39.30762	nonsynonymous SNV	uc002btj.3:c.G1028A:p.R343Q	2.62E-03	rs77311454	0.791	P	5.42	chr15	94899388	G	A	4983.1	0/1	0/1	1|1
2025	*MERTK*	homozygous rare	88.888889	nonsynonymous SNV	uc002thk.1:c.A60T:p.R20S	0.02	rs35898499	0.053	B	2.51	chr2	112656372	A	T	564.3	0/1	0/1	1|1
289	*MEX3C*	homozygous rare	30.555556	nonframeshift deletion	uc002lfc.4:c.529_537del:p.177_179del		rs78074704				chr18	48723153	CGGCCGCCGC	C	2212.4	0/1	0/1	1|1
3731	*MIRLET7BHG*	compound heterozygous	NA	nonsynonymous SNV	uc003bgr.4:c.G5C:p.W2S	9.68E-03	rs61995687				chr22	46493811	G	C	434.5	0/1	0/0	1|0
3731	*MIRLET7BHG*	compound heterozygous	NA	nonsynonymous SNV	uc003bgu.2:c.C399G:p.N133K	0.01	rs12159905				chr22	46505806	C	G	443.5	0/0	0/1	0|1
2002	*MLL2*	compound heterozygous	0.0648738	nonsynonymous SNV	uc001rta.4:c.G1955A:p.R652H	3.26E-05					chr12	49445511	C	T	222.5	0/1	0/0	1|0
2002	*MLL2*	compound heterozygous	0.0648738	nonsynonymous SNV	uc001rta.4:c.G248A:p.R83Q	0.02	rs55865069				chr12	49448463	C	T	656.4	0/0	0/1	0|1
3781	*MLN*	homozygous rare	74.952819	nonsynonymous SNV	uc003off.1:c.C74G:p.A25G	2.47E-03	rs146896135	0	B	3.76	chr6	33768867	G	C	1343.5	0/1	0/1	1|1
2034	*MMP19*	homozygous rare	44.031611	nonsynonymous SNV	uc001sib.3:c.C733T:p.P245S	7.73E-03	rs1056784	0.002	B	3.15	chr12	56233313	G	A	2642.9	0/1	0/1	1|1
69	*MMP19*	homozygous rare	44.031611	nonsynonymous SNV	uc001sia.3:c.G509A:p.R170Q	3.68E-03	rs150724096	0.705	P	3.41	chr12	56230980	C	T	1200.3	0/1	0/1	1|1
50	*MOXD1*	homozygous rare	12.455768	nonsynonymous SNV	uc003qde.3:c.G1258C:p.E420Q	0.02	rs36075540	0.001	B	5.13	chr6	132636820	C	G	2315.5	0/1	0/1	1|1
2037	*MTSS1*	homozygous rare	11.936778	frameshift deletion	uc003yrh.2:c.313_314del:p.105_105del		rs141972884				chr8	125568182	AGG	A	819.5	0/1	0/1	1|1
2812	*MX1*	deNovo	27.052371	stopgain SNV	uc010goq.3:c.C505T:p.R169X			0.735348	NA	4.82	chr21	42811689	C	T	764.3	0/0	0/0	0/1
2010	*MYH13*	compound heterozygous	16.743336	nonsynonymous SNV	uc002gmk.1:c.G4840C:p.D1614H	0.01	rs35069886				chr17	10212964	C	G	1412.8	0/1	0/0	1|0
2010	*MYH13*	compound heterozygous	16.743336	nonsynonymous SNV	uc002gmk.1:c.G2539A:p.A847T	5.36E-03	rs186137259				chr17	10231335	C	T	5153.7	0/0	0/1	0|1
2036	*MYH13*	compound heterozygous	16.743336	nonsynonymous SNV	uc002gmk.1:c.G4840C:p.D1614H	0.01	rs35069886				chr17	10212964	C	G	1093.6	0/0	0/1	0|1
2036	*MYH13*	compound heterozygous	16.743336	nonsynonymous SNV	uc002gmk.1:c.G2539A:p.A847T	5.36E-03	rs186137259				chr17	10231335	C	T	6345.4	0/1	0/0	1|0
24	*MYH4*	homozygous rare	63.623496	nonsynonymous SNV	uc002gmn.3:c.A986G:p.Q329R	0.02	rs35984286	0	B	5.32	chr17	10366204	T	C	951.8	0/1	0/1	1|1
2023	*MYH6*	compound heterozygous	0.6605331	nonsynonymous SNV	uc001wjv.3:c.C2807T:p.A936V	4.55E-04	rs199838024	0.937	D	4.7	chr14	23862996	G	A	1709.8	0/1	0/0	1|0
2023	*MYH6*	compound heterozygous	0.6605331	nonsynonymous SNV	uc001wjv.3:c.G622A:p.D208N	5.49E-03	rs142027794	0	B	3.17	chr14	23873940	C	T	2503.7	0/0	0/1	0|1
45	*MYH6*	homozygous rare	0.6605331	nonsynonymous SNV	uc001wjv.3:c.G3883C:p.E1295Q	3.21E-03	rs34935550	0.738	P	4.58	chr14	23858697	C	G	2365.8	0/1	0/1	1|1
155	*MYO5C*	homozygous rare	72.387356	nonsynonymous SNV	uc010ugc.2:c.C403T:p.R135C	0.02	rs55686434	0.997	D	3.81	chr15	52567851	G	A	3619.6	0/1	0/1	1|1
3781	*MYO5C*	compound heterozygous	72.387356	nonsynonymous SNV	uc010ugc.2:c.A608G:p.Y203C	3.02E-04	rs200785430	0.999	D	5.63	chr15	52564808	T	C	2828	0/1	0/0	1|0
3781	*MYO5C*	compound heterozygous	72.387356	nonsynonymous SNV	uc010ugc.2:c.C403T:p.R135C	0.02	rs55686434	0.997	D	3.81	chr15	52567851	G	A	1616.9	0/0	0/1	0|1
2035	*MYO9A*	compound heterozygous	14.785327	nonsynonymous SNV	uc002atk.3:c.C1885T:p.R629C	0.02	rs74475742	0.999	D	3.16	chr15	72172798	G	A	2629.2	0/0	0/1	0|1
2035	*MYO9A*	compound heterozygous	14.785327	nonsynonymous SNV	uc002atk.3:c.A1064T:p.N355I	0.02	rs80283650	0.442	P	2.99	chr15	72189952	T	A	4483.4	0/1	0/0	1|0
23	*MYO9A*	compound heterozygous	14.785327	nonsynonymous SNV	uc002atk.3:c.A1064T:p.N355I	0.02	rs80283650	0.442	P	2.99	chr15	72189952	T	A	569.2	0/1	0/0	1|0
23	*MYO9A*	compound heterozygous	14.785327	nonsynonymous SNV	uc002atk.3:c.T256C:p.S86P	0.02	rs55738821	0	B	-2.54	chr15	72190760	A	G	364.1	0/0	0/1	0|1
2037	*MYO9B*	compound heterozygous	10.928285	nonsynonymous SNV	uc002nfl.1:c.G10A:p.G4S	0.02	rs117099942				chr19	17311226	G	A	1094.8	0/1	0/0	1|0

Proband	Gene	Inheritance	Residual Variation Intolerance Score	Exonic Function	Amino Acid Change	Minor Allele Frequency EXAC	dbSNP138	PolyPhen2 Score	PolyPhen2 Prediction	GERP Score	Chromosome	Position	Ref	Alt	Quality Score	Paternal Genotype	Maternal Genotype	Proband Genotype
2037	*MYO9B*	compound heterozygous	10.928285	nonsynonymous SNV	uc010eak.3:c.T6155C:p.V2052A	0.02	rs117297085				chr19	17322800	T	C	476.4	0/0	0/1	0|1
3731	*MYOT*	homozygous rare	32.059448	nonsynonymous SNV	uc003lbv.3:c.A149G:p.Q50R	7.73E-03	rs34717730	0.578	P	5.67	chr5	137206489	A	G	1654.2	0/1	0/1	1|1
146	*NALCN*	homozygous rare	2.9841944	nonsynonymous SNV	uc001vox.1:c.C2305T:p.H769Y	0.02	rs76776920	0.984	D	5.09	chr13	101763029	G	A	1525.9	0/1	0/1	1|1
1131	*NAPA*	compound heterozygous	51.65723	nonsynonymous SNV	uc002pha.2:c.G25A:p.E9K	9.43E-04	rs141536777	0.98	D	4.99	chr19	48018173	C	T	1077.4	0/1	0/0	1|0
1131	*NAPA*	compound heterozygous	51.65723	nonsynonymous SNV	uc010elf.2:c.C134T:p.S45F						chr19	47996327	G	A	1984.9	0/0	0/1	0|1
2812	*NCAPD2*	homozygous rare	48.950224	nonsynonymous SNV	uc001qoo.2:c.G67T:p.V23L	0.02	rs61753197	0.16	P	4.29	chr12	6604331	G	T	4827.6	0/1	0/1	1|1
50	*NCOR1*	compound heterozygous	1.4979948	nonsynonymous SNV	uc010coy.3:c.A942C:p.L314F	8.90E-03	rs61753150	0.834	P	0.65	chr17	15973774	T	G	575.9	0/1	0/0	1|0
50	*NCOR1*	compound heterozygous	1.4979948	frameshift deletion	NCOR1:uc010coy.3:wholegene,	0.02	rs147790941				chr17	15983843	CAT	C	835.4	0/0	0/1	0|1
7952	*NDE1*	compound heterozygous	11.765747	nonsynonymous SNV	uc002ddu.1:c.C383T:p.T128I	8.73E-03	rs113493697	0.042	B	5.07	chr16	15785049	C	T	319.1	0/1	0/0	1|0
7952	*NDE1*	compound heterozygous	11.765747	nonsynonymous SNV	uc002ddu.1:c.G556T:p.A186S	4.07E-05	rs147233260	0.855	D	4.75	chr16	15788063	G	T	1350.2	0/0	0/1	0|1
3731	*NDUFA7*	compound heterozygous	81.888417	nonsynonymous SNV	uc002mjm.2:c.A158G:p.Y53C	1.63E-05		0.999	D	5.39	chr19	8381473	T	C	1087.5	0/0	0/1	0|1
3731	*NDUFA7*	compound heterozygous	81.888417	nonsynonymous SNV	uc002mjm.2:c.A152G:p.N51S	5.47E-04	rs146105751	0	B	3.31	chr19	8381479	T	C	1087.5	0/1	0/0	1|0
2035	*NDUFAF1*	compound heterozygous	82.165605	nonsynonymous SNV	uc001znx.3:c.G526A:p.E176K	0.02	rs35227875	0.088	B	3.34	chr15	41688732	C	T	1882.1	0/1	0/0	1|0
2035	*NDUFAF1*	compound heterozygous	82.165605	nonsynonymous SNV	uc001znx.3:c.T249A:p.D83E	3.17E-04	rs146540015	0.1	B	-1.31	chr15	41689009	A	T	1537.1	0/0	0/1	0|1
17	*NDUFS7*	compound heterozygous	39.95046	nonsynonymous SNV	uc002lse.4:c.C613G:p.R205G	2.49E-05		0.387	P	2.19	chr19	1395458	C	G	535.5	0/1	0/0	1|0
17	*NDUFS7*	compound heterozygous	39.95046	nonsynonymous SNV	uc002lsg.2:c.C77T:p.A26V	1.51E-04					chr19	1388765	C	T	184.1	0/0	0/1	0|1
3731	*NEK10*	homozygous rare	89.189667	nonsynonymous SNV	uc003cdt.2:c.A1202G:p.N401S	8.69E-03	rs141057699				chr3	27338698	T	C	2392.5	0/1	0/1	1|1
2001	*NEK9*	compound heterozygous	25.825666	nonsynonymous SNV	uc001xrj.3:c.A430G:p.T144A	1.87E-04	rs150013785	0.001	B	2.41	chr14	75553765	T	C	7328.9	0/0	0/1	0|1
2001	*NEK9*	compound heterozygous	25.825666	nonsynonymous SNV	uc001xrk.3:c.C752T:p.P251L	0.01	rs112467144	0.549	P	4.12	chr14	75558163	G	A	1240.2	0/1	0/0	1|0
151	*NIPA2*	homozygous rare	51.916726	frameshift deletion	uc001yva.3:c.1025_1400del:p.342_342del		rs368460716				chr15	23006218	TTCTT	T	1174.5	0/1	0/1	1|1
2027	*NLRP7*	compound heterozygous	73.11866	nonsynonymous SNV	uc002qig.4:c.A1532G:p.K511R	0.01	rs61743949	0.071	B	-4.69	chr19	55450655	T	C	1443.1	0/1	0/0	1|0
2027	*NLRP7*	compound heterozygous	73.11866	nonsynonymous SNV	uc002qig.4:c.A574C:p.M192L	1.69E-03	rs104895529	0.938	D	-0.676	chr19	55451613	T	G	1847.9	0/0	0/1	0|1
2033	*NOTCH1*	compound heterozygous	0.3302666	nonsynonymous SNV	uc004chz.3:c.G6853A:p.V2285I	0.02	rs61751489	0	B	1.38	chr9	139391338	C	T	621.8	0/1	0/0	1|0
2033	*NOTCH1*	compound heterozygous	0.3302666	nonsynonymous SNV	uc004cia.1:c.G1526A:p.R509H	0.01	rs61751543	0.874	D	3.49	chr9	139401233	C	T	541.2	0/0	0/1	0|1
155	*NOTCH2*	compound heterozygous	2.1467327	nonsynonymous SNV	uc001eik.3:c.T7223A:p.L2408H	1.77E-03	rs35586704	0.688	P	5	chr1	120458122	A	T	1173.5	0/0	0/1	0|1
155	*NOTCH2*	compound heterozygous	2.1467327	nonsynonymous SNV	uc001eik.3:c.A3980G:p.D1327G	0.01	rs61752484	0.067	B	5.47	chr1	120469147	T	C	1652.1	0/1	0/0	1|0
749	*NPAT*	homozygous rare	83.581033	nonsynonymous SNV	uc010rvv.2:c.G367A:p.D123N	3.90E-03	rs79119325	0.98	D	5.63	chr11	108032614	C	T	1504.2	0/1	0/1	1|1
2026	*NPHP4*	compound heterozygous	81.788158	nonsynonymous SNV	uc001alq.2:c.C2542T:p.R848W	0.01	rs17472401				chr1	5940243	G	A	2536.9	0/1	0/0	1|0
2026	*NPHP4*	compound heterozygous	81.788158	nonsynonymous SNV	uc001alq.2:c.G1852A:p.E618K	0.01	rs571655				chr1	5965455	C	T	1266.6	0/0	0/1	0|1
152	*NPHP4*	compound heterozygous	81.788158	nonsynonymous SNV	uc001alq.2:c.G2219A:p.R740H	0.02	rs34248917				chr1	5951013	C	T	185.7	0/0	0/1	0|1
152	*NPHP4*	compound heterozygous	81.788158	nonsynonymous SNV	uc001alq.2:c.G1852A:p.E618K	0.01	rs571655				chr1	5965455	C	T	610	0/1	0/0	1|0
14	*NPVF*	compound heterozygous	72.009908	frameshift substitution	NPVF:uc003sxo.3:exon3:c.547_553AG						chr7	25264779	TGAATAG	CT	1189.5	0/1	0/0	1|0
14	*NPVF*	compound heterozygous	72.009908	nonsynonymous SNV	uc003sxo.3:c.C168G:p.S56R	7.32E-04	rs147644964	0.991	D	2.23	chr7	25266616	G	C	258.9	0/0	0/1	0|1
2035	*NR1D2*	deNovo	60.56853	nonsynonymous SNV	uc003ccs.2:c.C523T:p.R175W			1	D	3.45	chr3	24003473	C	T	1055.4	0/0	0/0	0/1
155	*NSD1*	compound heterozygous	3.2377919	nonsynonymous SNV	uc003mfs.1:c.G2797C:p.A933P	0.02	rs28932179	0.004	B	-8.89	chr5	176638506	G	C	1166.6	0/0	0/1	0|1
155	*NSD1*	compound heterozygous	3.2377919	nonsynonymous SNV	uc021yip.1:c.A1083T:p.R361S	1.63E-05	rs377546578	0.405	P	3.13	chr5	176721980	A	T	236.5	0/1	0/0	1|0
2036	*NTRK1*	compound heterozygous	48.908941	nonsynonymous SNV	uc001fqh.1:c.G482A:p.R161H	3.01E-04	rs150271893	0.869	D	-0.246	chr1	156837949	G	A	1766.5	0/1	0/0	1|0
2036	*NTRK1*	compound heterozygous	48.908941	nonsynonymous SNV	uc001fqi.1:c.G1456A:p.E486K	5.20E-04	rs144901788	0.98	D	4.29	chr1	156845431	G	A	2537	0/0	0/1	0|1
2002	*NUP210*	compound heterozygous	1.7162067	nonsynonymous SNV	uc003bxv.1:c.G3655A:p.V1219I	3.25E-05	rs201994997	0	B	0.887	chr3	13378316	C	T	1511.2	0/1	0/0	1|0
2002	*NUP210*	compound heterozygous	1.7162067	nonsynonymous SNV	uc003bxv.1:c.G3154A:p.G1052S	2.74E-03	rs151008831	0.98	D	4.3	chr3	13383322	C	T	5000.9	0/0	0/1	0|1
28	*ORC1*	compound heterozygous	82.932295	nonsynonymous SNV	uc001ctt.3:c.C1115T:p.A372V	0.01	rs3087476	0.004	B	2.94	chr1	52854961	G	A	1283.7	0/1	0/0	1|0
28	*ORC1*	compound heterozygous	82.932295	nonsynonymous SNV	uc001ctt.3:c.G57C:p.R19S	4.15E-03	rs3087473	0.656	P	3.2	chr1	52867839	C	G	1684.2	0/0	0/1	0|1
2001	*OSBPL1_A*	compound heterozygous	NA	nonsynonymous SNV	uc002kvd.3:c.C825G:p.D275E	8.13E-06		0	B	-6.2	chr18	21750345	G	C	12219	0/0	0/1	0|1
2001	*OSBPL1_A*	compound heterozygous	NA	nonsynonymous SNV	uc002kve.3:c.T495A:p.N165K	4.03E-03	rs34907319	0.001	B	1.98	chr18	21913036	A	T	8862.2	0/1	0/0	1|0
2034	*OSBPL5*	compound heterozygous	20.960132	nonsynonymous SNV	uc001lxj.2:c.G401A:p.R134Q	0.02	rs35733103	0.725	P	-0.354	chr11	3114190	C	T	1085.8	0/0	0/1	0|1
2034	*OSBPL5*	compound heterozygous	20.960132	nonsynonymous SNV	uc009ydx.3:c.C55T:p.P19S	4.48E-05	rs377237273				chr11	3150396	G	A	1347.1	0/0	0/1	0|1
2033	*OTOF*	compound heterozygous	45.576787	nonsynonymous SNV	uc002rhi.3:c.T1681G:p.C561G	0.02	rs41288773	0.001	B	-1.8	chr2	26695500	A	C	765.4	0/0	0/1	0|1
2033	*OTOF*	compound heterozygous	45.576787	nonsynonymous SNV	uc002rhi.3:c.G311A:p.R104H	8.97E-04	rs80356592	0.939	D	4.09	chr2	26700309	C	T	1310.9	0/1	0/0	1|0
45	*OTOF*	homozygous rare	45.576787	nonsynonymous SNV	uc002rhk.3:c.G1723A:p.V575M	0.01	rs55676840	0.07	B	3.78	chr2	26703734	C	T	1384.1	0/1	0/1	1|1
151	*P2RX5*	homozygous rare	83.474876	frameshift deletion	uc002fwi.3:c.329delC:p.T110fs						chr17	3594280	GG	G	884.6	0/1	0/1	1|1
17	*PALLD*	compound heterozygous	63.670677	nonsynonymous SNV	uc003iru.3:c.A625C:p.N209H			0.374	P	5.2	chr4	169433280	A	C	773.8	0/1	0/0	1|0
17	*PALLD*	compound heterozygous	63.670677	nonsynonymous SNV	uc003iru.3:c.G764A:p.R255H	1.56E-03	rs146018183	0	B	0.176	chr4	169433419	G	A	289	0/0	0/1	0|1
24	*PALLD*	homozygous rare	63.670677	nonframeshift substitution	uc003iru.3:c.671_672CA:PALLD						chr4	169433326	TG	CA	1921.4	0/1	0/1	1|1
3737	*PCDH18*	compound heterozygous	5.3078556	nonsynonymous SNV	uc003ihe.4:c.G3154A:p.G1052R			0.99	D	4.3	chr4	138442437	C	T	937.6	0/0	0/1	0|1
3737	*PCDH18*	compound heterozygous	5.3078556	nonsynonymous SNV	uc003ihe.4:c.T2807C:p.L936P			0.956	D	4.06	chr4	138442784	A	G	1391.7	0/1	0/0	1|0
151	*PCDHB4*	homozygous rare	87.715263	nonframeshift substitution	PCDHB4:uc003lip.1:exon1:c.763_764TT						chr5	140502343	CC	TT	2331.9	0/1	0/1	1|1
3721	*PCDHGA1*	homozygous rare	62.190375	nonsynonymous SNV	uc003lji.2:c.C2335T:p.L779F	0.01	rs11575968	0.556785	NA	2.59	chr5	140712586	C	T	3919.3	0/1	0/1	1|1
2034	*PCDHGB5*	compound heterozygous	NA	nonsynonymous SNV	uc003lkf.2:c.C60G:p.F20L						chr5	140777754	C	G	328.5	0/0	0/1	0|1
2034	*PCDHGB5*	compound heterozygous	NA	nonsynonymous SNV	uc003lkf.2:c.G886A:p.E296K	0.01	rs145783835				chr5	140778580	G	A	463.8	0/1	0/0	1|0
2035	*PDCD11*	compound heterozygous	36.175985	nonsynonymous SNV	uc001kwy.1:c.G1307A:p.R436Q	9.76E-05	rs146207561	0.952	D	2.8	chr10	105173844	G	A	2619.4	0/0	0/1	0|1
2035	*PDCD11*	compound heterozygous	36.175985	nonsynonymous SNV	uc001kwy.1:c.T2696C:p.V899A	8.76E-03	rs61751511	0.968	D	5.69	chr10	105183348	T	C	2423.5	0/1	0/0	1|0
7952	*PEX11G*	homozygous rare	73.271998	nonsynonymous SNV	uc002mgk.1:c.C646T:p.L216F	0.02	rs11668511	0.997	D	4.77	chr19	7542168	G	A	1662.4	0/1	0/1	1|1
7760	*PEX14*	compound heterozygous	22.646851	nonsynonymous SNV	uc001arn.3:c.A26T:p.Q9L	1.47E-04	rs201979629	0.521065	NA	2.95	chr1	10535049	A	T	784.1	0/1	0/0	1|0
7760	*PEX14*	compound heterozygous	22.646851	nonsynonymous SNV	uc009vmw.3:c.T16G:p.S6A	2.33E-03	rs77261230	0.993	D	5.7	chr1	10659333	T	G	1039.4	0/0	0/1	0|1
3721	*PHF21B*	homozygous rare	89.242746	nonsynonymous SNV	uc003bfn.3:c.G418A:p.A140T	0.01	rs114948338	0	B	-3.2	chr22	45312306	C	T	784.8	0/1	0/1	1|1
2025	*PIEZO2*	compound heterozygous	NA	nonsynonymous SNV	uc002kos.2:c.C4128G:p.H1376Q	0.02	rs79261438				chr18	10750150	G	C	1548.9	0/1	0/0	1|0
2025	*PIEZO2*	compound heterozygous	NA	nonsynonymous SNV	uc002kos.2:c.G86T:p.G29V	4.32E-05					chr18	11066200	C	A	3429.6	0/0	0/1	0|1
1346	*PIEZO2*	compound heterozygous	NA	nonsynonymous SNV	uc002kos.2:c.A4948G:p.K1650E		rs140862542				chr18	10715782	T	C	1913.6	0/0	0/1	0|1
2001	*PIGQ*	homozygous rare	59.212078	nonframeshift substitution	uc010uuj.2:c.432_433TG:PIGQ						chr16	633353	TG	CA	3082.3	0/1	0/1	1|1
2002	*PIGQ*	homozygous rare	59.212078	frameshift substitution	PIGQ:uc002chp.3:exon1:c.157_165CCAGCCCC						chr16	632225	TCAGCCGCT	CCAGCCCC	1731.6	0/1	0/1	1|1
289	*PIGQ*	homozygous rare	59.212078	nonframeshift substitution	uc010uuj.2:c.432_433TG:PIGQ						chr16	633353	TG	CA	2397.6	0/1	0/1	1|1
289	*PIGQ*	homozygous rare	59.212078	frameshift substitution	PIGQ:uc002chp.3:exon1:c.157_165CCAGCCCC						chr16	632225	TCAGCCGCT	CCAGCCCC	1452.6	0/1	0/1	1|1
3737	*PIGQ*	homozygous rare	59.212078	nonframeshift substitution	uc010uuj.2:c.432_433TG:PIGQ						chr16	633353	TG	CA	1269.8	0/1	0/1	1|1
3781	*PIGQ*	homozygous rare	59.212078	nonframeshift substitution	uc010uuj.2:c.432_433TG:PIGQ						chr16	633353	TG	CA	1117.5	0/1	0/1	1|1
2026	*PIGZ*	homozygous rare	71.620665	nonframeshift substitution	PIGZ:uc003fxh.3:exon3:c.795_796GA						chr3	196674972	CT	TC	2624.8	0/1	0/1	1|1
3737	*PIGZ*	homozygous rare	71.620665	nonframeshift substitution	PIGZ:uc003fxh.3:exon3:c.795_796GA						chr3	196674972	CT	TC	2982.3	0/1	0/1	1|1
2036	*PIK3C2B*	compound heterozygous	77.925218	nonsynonymous SNV	uc001hax.1:c.G1729T:p.A577S	5.27E-03	rs61762602	0.002	B	2.7	chr1	204426183	C	A	2915.3	0/0	0/1	0|1
2036	*PIK3C2B*	compound heterozygous	77.925218	nonsynonymous SNV	uc001hax.1:c.C818A:p.P273H	5.97E-03	rs61740701	0.099	B	5	chr1	204438113	G	T	1467.8	0/1	0/0	1|0
3731	*PIK3C2G*	compound heterozygous	83.78745	nonsynonymous SNV	uc010sib.2:c.T1009G:p.C337G	8.59E-04	rs181944955				chr12	18446924	T	G	2393.9	0/0	0/1	0|1
3731	*PIK3C2G*	compound heterozygous	83.78745	stoploss SNV	uc001rdt.3:c.G4337C:p.X1446S	0.01	rs61757718				chr12	18800961	G	C	2930.5	0/1	0/0	1|0
4405	*PLA1A*	compound heterozygous	89.242746	nonsynonymous SNV	uc011bjc.2:c.G166A:p.V56I	1.55E-04	rs374066099	0.153	P	3.53	chr3	119334879	G	A	3568.5	0/1	0/0	1|0
4405	*PLA1A*	compound heterozygous	89.242746	nonsynonymous SNV	uc011bjc.2:c.C666A:p.N222K	0.01	rs56163046	0.002	B	0.297	chr3	119347611	C	A	1766.9	0/0	0/1	0|1
45	*PLCH2*	compound heterozygous	NA	nonsynonymous SNV	uc001aji.1:c.G344A:p.S115N	3.78E-03	rs41315664				chr1	2411245	G	A	406.7	0/1	0/0	1|0
45	*PLCH2*	compound heterozygous	NA	nonframeshift insertion	uc009vle.1:c.1605_1606insGTGGGGGCC:p.E535delinsEVGA	0.02	rs142848828				chr1	2430086	G	GGTGGGGGCC	2001.1	0/1	0/0	1|0
123	*POLN*	homozygous rare	84.18259	nonsynonymous SNV	uc010ich.1:c.A277G:p.S93G	0.02	rs34574483	0.01	B	-2.08	chr4	2172456	T	C	2812.8	0/1	0/1	1|1
1346	*PPP1R9A*	compound heterozygous	55.832744	nonsynonymous SNV	uc003unr.3:c.G446A:p.S149N	8.05E-03	rs61749905				chr7	94903477	G	A	2095.7	0/0	0/1	0|1
1346	*PPP1R9A*	compound heterozygous	55.832744	nonsynonymous SNV	uc003unr.3:c.G715A:p.A239T	1.30E-04	rs149491448	0.778	P	3.43	chr7	94915584	G	A	1272.9	0/1	0/0	1|0
45	*PREX2*	homozygous rare	15.416372	nonframeshift substitution	uc011lez.1:c.1623_1624GC:PREX2						chr8	68993013	AT	GC	1397.5	0/1	0/1	1|1
152	*PRKAG1*	compound heterozygous	66.820005	nonsynonymous SNV	uc001rsy.3:c.C266G:p.T89S	0.02	rs1126930	0.217	P	5.81	chr12	49399132	G	C	1607.2	0/0	0/1	0|1
152	*PRKAG1*	compound heterozygous	66.820005	nonsynonymous SNV	uc001rsy.3:c.T245A:p.F82Y			0.641996	NA	5.82	chr12	49399250	A	T	1567.9	0/1	0/0	1|0
123	*PRKDC*	compound heterozygous	NA	nonsynonymous SNV	uc003xqi.3:c.G10208A:p.G3403E	0.01	rs8178225				chr8	48711857	C	T	1137.6	0/1	0/0	1|0
123	*PRKDC*	compound heterozygous	NA	nonsynonymous SNV	uc003xqi.3:c.C2083T:p.P695S	7.82E-03	rs8178046				chr8	48841708	G	A	1235.9	0/0	0/1	0|1
135	*PRKDC*	compound heterozygous	NA	nonsynonymous SNV	uc003xqi.3:c.A7450G:p.R2484G						chr8	48752578	T	C	462.4	0/0	0/1	0|1
135	*PRKDC*	compound heterozygous	NA	nonsynonymous SNV	uc003xqi.3:c.C2083T:p.P695S	7.82E-03	rs8178046				chr8	48841708	G	A	1440.6	0/1	0/0	1|0
2036	*PRPH*	compound heterozygous	55.449398	nonsynonymous SNV	uc001rtu.3:c.G26A:p.R9Q	0.01	rs57451017	0.753	P	3	chr12	49689009	G	A	475.2	0/0	0/1	0|1
2036	*PRPH*	compound heterozygous	55.449398	nonsynonymous SNV	uc001rtu.3:c.G829A:p.A277T	0.02	rs62636520	0	B	5.6	chr12	49690798	G	A	564.1	0/1	0/0	1|0
3765	*PRR12*	compound heterozygous	NA	nonsynonymous SNV	uc002poo.4:c.C1118T:p.A373V	4.70E-04	rs376580810				chr19	50098710	C	T	327.2	0/0	0/1	0|1
3765	*PRR12*	compound heterozygous	NA	nonsynonymous SNV	uc002poo.4:c.G1130T:p.G377V	4.98E-04	rs201908223				chr19	50098722	G	T	445.4	0/1	0/0	1|0
2023	*PSRC1*	compound heterozygous	85.727766	stopgain SNV	uc001dxf.3:c.A745T:p.K249X	0.01	rs116389032	0.688871	NA	1.05	chr1	109823457	T	A	940.9	0/1	0/0	1|0
2023	*PSRC1*	compound heterozygous	85.727766	nonsynonymous SNV	uc001dxb.3:c.C256T:p.P86S	8.57E-03	rs76057315	0.996	D	5.82	chr1	109823537	G	A	1310.6	0/0	0/1	0|1
2008	*PTPN14*	compound heterozygous	32.27766	nonsynonymous SNV	uc021piy.1:c.C1682T:p.P561L	1.14E-04	rs146466456	0	B	1.12	chr1	214556808	G	A	595.7	0/0	0/1	0|1
2008	*PTPN14*	compound heterozygous	32.27766	nonsynonymous SNV	uc021piy.1:c.C763T:p.R255W	1.26E-03	rs138557012	0.999	D	3.72	chr1	214557727	G	A	664.2	0/1	0/0	1|0
23	*PTPRC*	compound heterozygous	68.571597	nonsynonymous SNV	uc009wze.1:c.G169C:p.D57H	0.01	rs41269905	0.992	D	2.71	chr1	198668761	G	C	1105.4	0/0	0/1	0|1
23	*PTPRC*	compound heterozygous	68.571597	nonsynonymous SNV	uc001gut.1:c.A397C:p.I133L	0.02	rs2230606	0	B	-9.34	chr1	198676063	A	C	657.4	0/0	0/1	0|1

Proband	Gene	Inheritance	Residual Variation Intolerance Score	Exonic Function	Amino Acid Change	Minor Allele Frequency EXAC	dbSNP138	PolyPhen2 Score	PolyPhen2 Prediction	GERP Score	Chromosome	Position	Ref	Alt	Quality Score	Paternal Genotype	Maternal Genotype	Proband Genotype
151	*PTPRC*	compound heterozygous	68.571597	nonsynonymous SNV	uc009wze.1:c.G169C:p.D57H	0.01	rs41269905	0.992	D	2.71	chr1	198668761	G	C	1741	0/0	0/1	0|1
151	*PTPRC*	compound heterozygous	68.571597				rs116151234				chr1	198661414	T	G	434.7	0/1	0/0	1|0
8522	*PTPRG*	compound heterozygous	2.4298184	nonsynonymous SNV	uc003dlb.3:c.C623T:p.P208L	1.63E-04	rs61757808	0.07	B	4.47	chr3	62118283	C	T	3852.9	0/0	0/1	0|1
8522	*PTPRG*	compound heterozygous	2.4298184	nonsynonymous SNV	uc003dlb.3:c.A644G:p.D215G	7.32E-05	rs140916981	0.984	D	5.45	chr3	62118304	A	G	4102.6	0/1	0/0	1|0
3731	*PTPRJ*	compound heterozygous	86.417787	nonsynonymous SNV	uc001ngo.4:c.G1114A:p.V372I	8.80E-03	rs2229703	0.014	B	-10.8	chr11	48149352	G	A	632.1	0/0	0/1	0|1
3731	*PTPRJ*	compound heterozygous	86.417787	nonsynonymous SNV	uc010rhr.1:c.G517A:p.E173K	4.07E-04	rs181029182	0.881	D	0.0462	chr11	48161067	G	A	1410	0/1	0/0	1|0
3721	*PTPRN2*	compound heterozygous	89.195565	nonsynonymous SNV	uc003wnp.3:c.T1112A:p.L371H	1.31E-03	rs7456452	0.03	B	-7.81	chr7	157929357	A	T	1400.3	0/1	0/0	1|0
3721	*PTPRN2*	compound heterozygous	89.195565	nonsynonymous SNV	uc003wnp.3:c.G1099A:p.D367N	9.56E-03	rs55645575	0.023	B	0.387	chr7	157929370	C	T	1192.3	0/0	0/1	0|1
114	*PTPRU*	deNovo	2.2823779	nonsynonymous SNV	uc001brw.3:c.C2722T:p.R908W	4.07E-05	rs150833997	1	D	2.49	chr1	29631340	C	T	147.7	0/0	0/0	0/1
3731	*PTPRZ1*	homozygous rare	1.3505544	nonframeshift substitution	PTPRZ1:uc003vjy.3:exon12:c.4290_4298CAGAGA						chr7	121653390	TGACAGAGG	CAGAGA	2434.2	0/1	0/1	1|1
749	*RAG2*	homozygous rare	55.608634	nonsynonymous SNV	uc021qge.1:c.G22A:p.V8I	3.13E-03	rs150762709	0.024	B	4.48	chr11	36615697	C	T	2265.6	0/1	0/1	1|1
146	*RALGDS*	homozygous rare	4.6414249	nonsynonymous SNV	uc004ccv.1:c.C2017T:p.P673S	0.02	rs641999				chr9	135976826	G	A	3732.9	0/1	0/1	1|1
2035	*RBM10*	homozygous rare	15.764331	nonsynonymous SNV	uc004dhg.3:c.G1657C:p.A553P	0.02	rs79693964	0.121	B	4.3	chrX	47041666	G	C	1681.6	0/1	0/1	1|1
7952	*RCBTB1*	compound heterozygous	18.783911	nonsynonymous SNV	uc001vde.1:c.A421G:p.M141V	2.03E-04	rs147650879	0.048	B	3.72	chr13	50134077	T	C	1768.7	0/0	0/1	0|1
7952	*RCBTB1*	compound heterozygous	18.783911	nonsynonymous SNV	uc001vde.1:c.A207C:p.E69D	5.40E-03	rs138308390	0.004	B	4.49	chr13	50140824	T	G	3367.4	0/1	0/0	1|0
3721	*RECQL*	compound heterozygous	8.8995046	nonsynonymous SNV	uc001rey.3:c.G1483C:p.D495H	3.88E-03	rs6499	0.003	B	5.09	chr12	21624546	C	G	1381.8	0/0	0/1	0|1
3721	*RECQL*	compound heterozygous	8.8995046	nonsynonymous SNV	uc001rey.3:c.G1361A:p.R454H	1.63E-05		1	D	4.75	chr12	21626571	C	T	1970	0/1	0/0	1|0
2001	*RELN*	compound heterozygous	1.4626091	nonsynonymous SNV	uc010liz.3:c.A1888C:p.S630R	0.02	rs115734214	0.949	D	5.27	chr7	103292112	T	G	1998.2	0/1	0/0	1|0
2001	*RELN*	compound heterozygous	1.4626091	nonsynonymous SNV	uc010liz.3:c.C1799T:p.S600F	0.01	rs78008536	0.975	D	5.45	chr7	103292201	G	A	2944.8	0/0	0/1	0|1
2037	*RHBG*	homozygous rare	NA	nonsynonymous SNV	uc010pho.2:c.C243G:p.F81L	0.01	rs150963900				chr1	156347147	C	G	4179.3	0/1	0/1	1|1
23	*RHBG*	homozygous rare	NA	frameshift deletion	uc009wrz.3:c.1169delC:p.S390fs		rs11303415				chr1	156354347	TC	T	1516	0/1	0/1	1|1
50	*RTKN2*	homozygous rare	48.779193	nonframeshift deletion	uc009xpf.1:c.321_326del:p.107_109del		rs59044276				chr10	63976913	AGCTTCA	A	6522.1	0/1	0/1	1|1
114	*RTKN2*	homozygous rare	48.779193	nonframeshift deletion	uc009xpf.1:c.321_326del:p.107_109del		rs59044276				chr10	63976913	AGCTTCA	A	2430.8	0/1	0/1	1|1
1131	*RTL1*	compound heterozygous	73.649446	nonsynonymous SNV	uc010txj.1:c.G3323A:p.R1108Q	0.02	rs117476938				chr14	101347803	C	T	245.8	0/0	0/1	0|1
1131	*RTL1*	compound heterozygous	73.649446	nonsynonymous SNV	uc010txj.1:c.C1672A:p.P558T	0.01	rs41286560				chr14	101349454	G	T	1245.4	0/1	0/0	1|0
1131	*RYR1*	compound heterozygous	0.0058976	nonsynonymous SNV	uc002oit.3:c.A89T:p.E30V	2.37E-04	rs145771708	0.158	P	4.41	chr19	38931428	A	T	602.1	0/1	0/0	1|0
1131	*RYR1*	compound heterozygous	0.0058976	nonsynonymous SNV	uc002oiu.3:c.G13498C:p.D4500H	3.31E-03	rs150396398	0.456933	NA	3.76	chr19	39057626	G	C	1248.1	0/0	0/1	0|1
2025	*SAMD3*	compound heterozygous	29.429111	nonsynonymous SNV	uc003qby.3:c.G541A:p.D181N	2.22E-03	rs150968705	0.99	D	5.31	chr6	130505611	C	T	2037.7	0/0	0/1	0|1
2025	*SAMD3*	compound heterozygous	29.429111	nonsynonymous SNV	uc003qby.3:c.G303T:p.R101S	5.69E-04	rs138130162	0	B	-2.45	chr6	130530720	C	A	1812.8	0/1	0/0	1|0
4405	*SCARF2*	compound heterozygous	NA	nonsynonymous SNV	uc002zsj.2:c.C1982T:p.P661L	0.02	rs9680797	0.563882	NA	2.78	chr22	20780296	G	A	424.7	0/0	0/1	0|1
4405	*SCARF2*	compound heterozygous	NA	nonsynonymous SNV	uc002zsj.2:c.C1661T:p.S554L	2.83E-03	rs150853613	0.564209	NA	4.36	chr22	20781732	G	A	1787.6	0/1	0/0	1|0
4405	*SCML4*	compound heterozygous	50.224109	nonsynonymous SNV	uc003pry.4:c.C283T:p.R95W	0.02	rs56215636				chr6	108029180	G	A	661.2	0/0	0/1	0|1
4405	*SCML4*	compound heterozygous	50.224109	nonsynonymous SNV	uc003prz.4:c.G142A:p.A48T	1.90E-03	rs143639662	0	B	0.998	chr6	108068064	C	T	680.3	0/1	0/0	1|0
2001	*SCN9A*	compound heterozygous	73.625855	nonsynonymous SNV	uc010fpl.3:c.T4612C:p.W1538R	2.07E-03	rs202084411				chr2	167060594	A	G	17600.7	0/0	0/1	0|1
2001	*SCN9A*	compound heterozygous	73.625855	nonsynonymous SNV	uc010fpl.3:c.G3329A:p.R1110Q	0.02	rs74401238				chr2	167108385	C	T	10215.2	0/1	0/0	1|0
135	*SCRN3*	homozygous rare	53.851144	frameshift deletion	uc002uis.3:c.477_489del:p.159_163del						chr2	175292598	ATTTATCAGTCAAA	A	2873.8	0/1	0/1	1|1
3731	*SCUBE1*	homozygous rare	3.9278132	nonsynonymous SNV	uc003bdt.2:c.C674T:p.T225M	9.38E-03	rs73420094	0	B	0.643	chr22	43654278	G	A	1903.7	0/1	0/1	1|1
2002	*SEL1L2*	compound heterozygous	77.229299	nonsynonymous SNV	uc010gcf.3:c.C1162T:p.P388S	1.80E-04	rs201283948	0.999	D	2.84	chr20	13850792	G	A	3324.8	0/0	0/1	0|1
2002	*SEL1L2*	compound heterozygous	77.229299	nonsynonymous SNV	uc010gcf.3:c.A577G:p.I193V	0.02	rs141957868	0.588	P	5.24	chr20	13869131	T	C	2788.4	0/1	0/0	1|0
2035	*SERINC2*	compound heterozygous	66.165369	nonsynonymous SNV	uc001bst.3:c.G259A:p.D87N	5.64E-03	rs112561697	0.024	B	3.95	chr1	31897587	G	A	638.4	0/1	0/0	1|0
2035	*SERINC2*	compound heterozygous	66.165369	nonsynonymous SNV	uc001bst.3:c.G563C:p.R188P	4.07E-05		0.817	P	-4.65	chr1	31898713	G	C	1073.6	0/0	0/1	0|1
2812	*SEZ6L2*	homozygous rare	6.5758434	nonsynonymous SNV	uc002dus.4:c.G1210A:p.D404N	0.02	rs113753753	0.988	D	5.35	chr16	29891206	C	T	2555.3	0/1	0/1	1|1
2026	*SGK223*	homozygous rare	54.128332	nonframeshift substitution	SGK223:uc003wsh.4:exon2:c.1700_1701TA						chr8	8234218	CG	TA	715.8	0/1	0/1	1|1
2010	*SIDT1*	deNovo	9.7782496	nonsynonymous SNV	uc011bii.2:c.G818A:p.R273Q	3.25E-05	rs147948601	0.001	B	2.24	chr3	113346530	G	A	1272.2	0/0	0/0	1|1
23	*SLC22A31*	homozygous rare	NA	nonframeshift substitution	SLC22A31:uc021tmr.1:exon4:c.233_234AT						chr16	89265100	GC	AT	2242.8	0/1	0/1	1|1
2035	*SLC26A7*	compound heterozygous	62.096013	nonsynonymous SNV	uc003yez.3:c.A643G:p.I215V	0.02	rs16912250	0.004	B	4.49	chr8	92346523	A	G	2861.5	0/1	0/0	1|0
2035	*SLC26A7*	compound heterozygous	62.096013	nonsynonymous SNV	uc003yez.3:c.A1454T:p.E485V	3.22E-03	rs140569478	0.01	B	5.33	chr8	92375732	A	T	4386.5	0/0	0/1	0|1
2023	*SLC27A4*	homozygous rare	7.1950932	nonsynonymous SNV	uc004but.3:c.A1052G:p.N351S	0.02	rs111417655	0	B	-1.94	chr9	131115368	A	G	2414.4	0/1	0/1	1|1
2034	*SLC6A17*	homozygous rare	18.901864	nonframeshift substitution	SLC6A17:uc009wfq.3:exon2:c.168_169CA						chr1	110709719	TG	CA	4217.5	0/1	0/1	1|1
2035	*SLC6A17*	homozygous rare	18.901864	nonframeshift substitution	SLC6A17:uc009wfq.3:exon2:c.168_169CA						chr1	110709719	TG	CA	3111.2	0/1	0/1	1|1
28	*SLC6A17*	homozygous rare	18.901864	nonframeshift substitution	SLC6A17:uc009wfq.3:exon2:c.168_169CA						chr1	110709719	TG	CA	934.3	0/1	0/1	1|1
10	*SLCO5A1*	compound heterozygous	80.337344	nonsynonymous SNV	uc003xyk.3:c.G1124A:p.R375Q	4.23E-03	rs149362749	1	D	4.94	chr8	70667793	C	T	1006.9	0/1	0/0	1|0
10	*SLCO5A1*	compound heterozygous	80.337344	nonsynonymous SNV	uc003xyk.3:c.G685A:p.A229T	8.78E-04	rs111622961	0.003	B	4.12	chr8	70744224	C	T	1497.5	0/0	0/1	0|1
152	*SMARCAD1*	compound heterozygous	70.582685	nonsynonymous SNV	uc011cdw.2:c.T37C:p.W13R		rs200569328	0.998	D	5.35	chr4	95185903	T	C	1380.6	0/1	0/0	1|0
152	*SMARCAD1*	compound heterozygous	70.582685	nonsynonymous SNV	uc011cdw.2:c.C637T:p.H213Y	8.95E-04	rs151125076	0.958	D	5.07	chr4	95197608	C	T	568.2	0/0	0/1	0|1
54	*SNRPC*	homozygous rare	51.037981	nonframeshift substitution	SNRPC:uc021yyw.1:exon1:c.41_41delinsATCT						chr6	34725658	G	ATCT	6487.5	0/1	0/1	1|1
69	*SON*	compound heterozygous	1.9933947	nonsynonymous SNV	uc002ysg.3:c.T517G:p.L173V	4.07E-05		0.813	P	4.04	chr21	34925081	T	G	520.9	0/1	0/0	1|0
69	*SON*	compound heterozygous	1.9933947	nonsynonymous SNV	uc002ysg.3:c.G1063A:p.A355T	1.65E-05	rs140276173	0	B	2.16	chr21	34925627	G	A	878.5	0/0	0/1	0|1
2020	*SPG11*	compound heterozygous	22.788393	nonsynonymous SNV	uc010bdw.3:c.A1646G:p.K549R	3.82E-03	rs76116949	0.968	D	3.3	chr15	44855395	T	C	3123.1	0/1	0/0	1|0
2020	*SPG11*	compound heterozygous	22.788393	nonsynonymous SNV	uc001ztx.3:c.T1698G:p.D566E	0.01	rs79708848	0.208	P	1.99	chr15	44925740	A	C	3568.1	0/0	0/1	0|1
2033	*SPG11*	compound heterozygous	22.788393	nonsynonymous SNV	uc001ztx.3:c.G2083A:p.A695T	0.01	rs78183930	0.674	P	5.57	chr15	44918690	C	T	4150.6	0/1	0/0	1|0
2033	*SPG11*	compound heterozygous	22.788393	nonsynonymous SNV	uc001ztx.3:c.G808A:p.V270I	6.05E-03	rs80338868	0.728	P	5.25	chr15	44949354	C	T	2702.9	0/0	0/1	0|1
8522	*SPPL2B*	deNovo	NA	nonsynonymous SNV	uc002lvr.3:c.G916A:p.V306I	2.44E-05					chr19	2340975	G	A	330	0/0	0/0	1|1
24	*SPTY2D1*	homozygous rare	74.581269	nonframeshift deletion	uc001moy.3:c.320_322del:p.107_108del	0.02	rs66514853				chr11	18637498	CTCT	C	4187.2	0/1	0/1	1|1
2002	*SRA1*	compound heterozygous	82.661005	nonframeshift substitution	uc003lfz.3:c.106_106delinsCGAC:SRA1		rs368142622				chr5	139931629	C	GTCG	3713.5	0/0	0/1	0|1
2002	*SRA1*	compound heterozygous	82.661005	nonsynonymous SNV	uc003lga.3:c.C94G:p.Q32E	6.93E-03	rs35610885	0.956	D	4.47	chr5	139936825	G	C	979.9	0/1	0/0	1|0
2028	*SRA1*	homozygous rare	82.661005	nonframeshift substitution	uc003lfz.3:c.106_106delinsCGAC:SRA1		rs368142622				chr5	139931629	C	GTCG	8008.3	0/1	0/1	1|1
50	*SRA1*	homozygous rare	82.661005	nonframeshift substitution	uc003lfz.3:c.106_106delinsCGAC:SRA1		rs368142622				chr5	139931629	C	GTCG	1911.8	0/1	0/1	1|1
313	*SRA1*	homozygous rare	82.661005	nonframeshift substitution	uc003lfz.3:c.106_106delinsCGAC:SRA1		rs368142622				chr5	139931629	C	GTCG	1747.5	0/1	0/1	1|1
7952	*SRCAP*	compound heterozygous	0.1533381	nonsynonymous SNV	uc002dzg.1:c.G1130A:p.S377N	8.05E-04	rs139339184	0.508889	NA	3.72	chr16	30723222	G	A	1361.6	0/0	0/1	0|1
7952	*SRCAP*	compound heterozygous	0.1533381	nonsynonymous SNV	uc002dzg.1:c.A5090G:p.E1697G	4.02E-03	rs117480926	0.562115	NA	5.35	chr16	30740333	A	G	1082.9	0/1	0/0	1|0
2020	*SSFA2*	compound heterozygous	68.595188	nonsynonymous SNV	uc002uoh.3:c.C107G:p.A36G	0.01	rs114985669	0.98	D	0.817	chr2	182756900	C	G	902.4	0/0	0/1	0|1
2020	*SSFA2*	compound heterozygous	68.595188	nonsynonymous SNV	uc002uol.3:c.A749G:p.E250G	1.33E-03	rs147357576	0.001	B	2.83	chr2	182766988	A	G	1947.5	0/1	0/0	1|0
3737	*SSH1*	homozygous rare	28.308563	nonsynonymous SNV	uc010sxg.2:c.C59T:p.T20M	0.01	rs117096112				chr12	109221123	G	A	4797	0/1	0/1	1|1
155	*SSH2*	homozygous rare	45.682944	nonsynonymous SNV	uc002heo.1:c.G4192A:p.G1398S	0.02	rs61737980	0.986	D	3.75	chr17	27957939	C	T	1370.3	0/1	0/1	1|1
2036	*STARD8*	homozygous rare	86.358811	nonsynonymous SNV	uc004dxa.3:c.C1742T:p.T581M	1.15E-04		0.92	D	3.98	chrX	67940198	C	T	892.9	0/1	0/1	1|1
14	*STK33*	homozygous rare	48.690729	nonframeshift substitution	uc001mgl.3:c.747_748GA:STK33						chr11	8435077	CA	TC	3444.9	0/1	0/1	1|1
120	*STK33*	homozygous rare	48.690729	nonframeshift substitution	uc001mgl.3:c.747_748GA:STK33						chr11	8435077	CA	TC	2755.7	0/1	0/1	1|1
2023	*STRN*	compound heterozygous	38.983251	nonsynonymous SNV	uc010ezx.3:c.C1205T:p.T402I	0.01	rs77353404	0.11	B	5.57	chr2	37105041	G	A	2922.3	0/0	0/1	0|1
2023	*STRN*	compound heterozygous	38.983251	nonsynonymous SNV	uc010ezx.3:c.C1027T:p.P343S	0.02	rs80243841	0.01	B	5.48	chr2	37111123	G	A	2236.5	0/1	0/0	1|0
313	*SULT2B1*	compound heterozygous	83.362821	nonsynonymous SNV	uc002pjm.3:c.T275C:p.I92T			0	B	2.97	chr19	49090591	T	C	509.3	0/1	0/0	1|0
313	*SULT2B1*	compound heterozygous	83.362821	nonsynonymous SNV	uc002pjm.3:c.C989T:p.P330L	0.01	rs17842463	0.518073	NA	-2.29	chr19	49102599	C	T	369.1	0/0	0/1	0|1
3731	*SUSD3*	homozygous rare	78.461901	nonsynonymous SNV	uc004atb.3:c.G531T:p.Q177H	3.18E-03	rs41313323	0.995	D	1.57	chr9	95841858	G	T	1001	0/1	0/1	1|1
2022	*SYNM*	compound heterozygous	NA	nonsynonymous SNV	uc002buo.3:c.C1199T:p.S400L	1.06E-04	rs376775614				chr15	99669767	C	T	936.6	0/1	0/0	1|0
2022	*SYNM*	compound heterozygous	NA	nonsynonymous SNV	uc002buo.3:c.A2924G:p.Q975R	0.02	rs5030695				chr15	99671492	A	G	1392.7	0/0	0/1	0|1
3731	*SYNM*	compound heterozygous	NA	nonsynonymous SNV	uc002buo.3:c.G1142A:p.R381H	4.08E-05					chr15	99669710	G	A	658.4	0/1	0/0	1|0
3731	*SYNM*	compound heterozygous	NA	nonsynonymous SNV	uc002bup.3:c.G3900T:p.L1300F	1.63E-05	rs184717724				chr15	99672468	G	T	1004.9	0/0	0/1	0|1
2023	*SYNRG*	compound heterozygous	14.820712	nonsynonymous SNV	uc002hof.3:c.A1894G:p.S632G	2.68E-04	rs142492240	0.028	B	5.68	chr17	35902380	T	C	2314	0/1	0/0	1|0
2023	*SYNRG*	compound heterozygous	14.820712	nonsynonymous SNV	uc002hof.3:c.C1790T:p.T597I	8.13E-06		0	B	3.71	chr17	35902484	G	A	2878.4	0/0	0/1	0|1
54	*TANC1*	homozygous rare	5.2665723	nonframeshift substitution	uc010fon.3:c.1248_1249TG:TANC1						chr2	160086653	CA	TG	1496.8	0/1	0/1	1|1
2020	*TARBP1*	compound heterozygous	36.364709	nonsynonymous SNV	uc001hwd.3:c.A1796G:p.K599R	6.51E-05	rs375951146	0	B	5.24	chr1	234586239	T	C	2615.6	0/0	0/1	0|1
2020	*TARBP1*	compound heterozygous	36.364709	nonsynonymous SNV	uc001hwd.3:c.A1538G:p.D513G	8.21E-03	rs35562024	0.992	D	2.36	chr1	234595070	T	C	1392	0/1	0/0	1|0
3721	*TCR-alpha*	homozygous rare	NA	nonsynonymous SNV	uc001wdb.2:c.T17C:p.L6S	0.01	rs61746511				chr14	22573636	T	C	3146.2	0/1	0/1	1|1
4405	*TCTN3*	compound heterozygous	69.45624	nonsynonymous SNV	uc010qoi.2:c.G733A:p.A245T			0.001	B	3.23	chr10	97443305	C	T	2232.2	0/0	0/1	0|1
4405	*TCTN3*	compound heterozygous	69.45624	nonsynonymous SNV	uc001klb.4:c.G925T:p.A309S	0.02	rs55859130				chr10	97445357	C	A	1746.8	0/1	0/0	1|0
155	*TDO2*	homozygous rare	53.727294	nonsynonymous SNV	uc003ipf.1:c.A685C:p.N229H	0.02	rs116105292	0.688	P	5.4	chr4	156832715	A	C	1150.4	0/1	0/1	1|1
146	*TDRD9*	compound heterozygous	39.278132	nonsynonymous SNV	uc001yom.4:c.G3514A:p.V1172I	0.01	rs76761167				chr14	104500301	G	A	466.5	0/0	0/1	0|1
146	*TDRD9*	compound heterozygous	39.278132	nonsynonymous SNV	uc001yom.4:c.G3826T:p.V1276F	1.29E-03	rs182776644				chr14	104506642	G	T	404.9	0/1	0/0	1|0
289	*TECTA*	compound heterozygous	1.032083	nonsynonymous SNV	uc010rzo.2:c.C2061G:p.N687K	1.66E-03	rs139165033	0.995	D	3.93	chr11	120998747	C	G	1669.3	0/0	0/1	0|1
289	*TECTA*	compound heterozygous	1.032083	nonsynonymous SNV	uc010rzo.2:c.G3316C:p.G1106R			0.871	D	4.88	chr11	121008504	G	C	895.3	0/1	0/0	1|0
2007	*TGM5*	homozygous rare	88.092711	nonsynonymous SNV	uc001zrd.2:c.C326T:p.T109M	9.78E-03	rs113463533	0.001	B	-3.59	chr15	43552360	G	A	2201.9	0/1	0/1	1|1
24	*THAP7-AS1*	homozygous rare	NA	frameshift deletion	uc002ztt.1:c.157_160del:p.53_54del		rs59698086				chr22	21356894	GACAG	G	2124.8	0/1	0/1	1|1
45	*THSD1*	homozygous rare	60.680585	nonsynonymous SNV	uc001vgo.3:c.G871A:p.E291K	0.02	rs41292808	0.968	D	1.96	chr13	52971517	C	T	2200.8	0/1	0/1	1|1
10	*THSD7B*	homozygous rare	NA	nonsynonymous SNV	uc002tvb.3:c.G635A:p.R212H	0.02	rs61741426				chr2	137852550	G	A	1535.9	0/1	0/1	1|1

Proband	Gene	Inheritance	Residual Variation Intolerance Score	Exonic Function	Amino Acid Change	Minor Allele Frequency EXAC	dbSNP138	PolyPhen2 Score	PolyPhen2 Prediction	GERP Score	Chromosome	Position	Ref	Alt	Quality Score	Paternal Genotype	Maternal Genotype	Proband Genotype
289	*THSD7B*	compound heterozygous	NA	nonsynonymous SNV	uc002tvb.3:c.G635A:p.R212H	0.02	rs61741426				chr2	137852550	G	A	375.7	0/0	0/1	0|1
289	*THSD7B*	compound heterozygous	NA	nonsynonymous SNV	uc002tvb.3:c.C1400T:p.T467M	3.76E-04	rs61741154				chr2	137988713	C	T	389.5	0/1	0/0	1|0
152	*TIAM1*	homozygous rare	9.8431234	nonframeshift substitution	uc011adk.1:c.739_740AT:TIAM1						chr21	32638549	CC	AT	2434	0/1	0/1	1|1
749	*TIAM1*	compound heterozygous	9.8431234	nonsynonymous SNV	uc011adl.1:c.A4226G:p.K1409R	8.42E-03	rs111536576	0.178	P	0.538	chr21	32493056	T	C	1193	0/1	0/0	1|0
749	*TIAM1*	compound heterozygous	9.8431234	nonsynonymous SNV	uc011adk.1:c.G92A:p.R31H	8.38E-03	rs141720377	0.999	D	3.67	chr21	32639197	C	T	1146	0/0	0/1	0|1
3781	*TIMP1*	homozygous rare	58.262562	nonsynonymous SNV	uc011mlr.1:c.C32T:p.P11L	0.01	rs1043428				chrX	47444663	C	T	1592.9	0/1	0/1	1|1
7952	*TJAP1*	homozygous rare	69.509318	nonsynonymous SNV	uc010jyp.2:c.G100A:p.D34N	6.42E-03	rs146174516	0.941	D	4.47	chr6	43469358	G	A	1403.2	0/1	0/1	1|1
3721	*TMEM184A*	homozygous rare	45.570889	nonframeshift substitution	uc003skt.4:c.1105_1105delinsGGCG:TMEM184A						chr7	1586662	T	CGCC	3157.2	0/1	0/1	1|1
69	*TMIE*	homozygous rare	41.247936	nonframeshift deletion	uc010hjk.1:c.391_393del:p.131_131del						chr3	46751097	GAAG	G	1574.5	0/1	0/1	1|1
1346	*TMPRSS13*	homozygous rare	84.642604	nonsynonymous SNV	uc001pru.2:c.G1304T:p.R435L	0.02	rs80264810				chr11	117779305	C	A	641.6	0/1	0/1	1|1
151	*TPMT*	homozygous rare	87.402689	nonsynonymous SNV	uc003ncm.3:c.G460A:p.A154T	0.02	rs1800460	0.848	P	-0.936	chr6	18139228	C	T	2051.1	0/1	0/1	1|1
7760	*TRAF4*	deNovo	26.533381	stopgain SNV	uc002hcs.3:c.C622T:p.Q208X			0.735351	NA	5.13	chr17	27075439	C	T	377.1	0/0	0/0	0/1
66	*TRGV3*	homozygous rare	NA	nonsynonymous SNV	uc003tgr.2:c.A332C:p.Y111S	0.01	rs150804886				chr7	38398135	T	G	1692.9	0/1	0/1	1|1
2028	*TRMT11*	homozygous rare	40.44586	nonsynonymous SNV	uc003qam.3:c.A916G:p.I306V	0.02	rs116913944	0.026	B	3.95	chr6	126332554	A	G	2469.7	0/1	0/1	1|1
23	*TRPM3*	compound heterozygous	6.982779	nonsynonymous SNV	uc004ahv.3:c.G4012A:p.G1338R	3.46E-03	rs148192709	0.988	D	5.25	chr9	73151387	C	T	303.5	0/1	0/0	1|0
23	*TRPM3*	compound heterozygous	6.982779	nonsynonymous SNV	uc004ahv.3:c.C2105G:p.S702C	1.63E-05	rs138882109	0.961	D	5.24	chr9	73218319	G	C	425.5	0/0	0/1	0|1
2020	*TSC2*	compound heterozygous	0.8728474	nonsynonymous SNV	uc002cop.3:c.G500A:p.R167Q	0.01	rs1800725	0.999	D	4.66	chr16	2110795	G	A	1670.9	0/1	0/0	1|0
2020	*TSC2*	compound heterozygous	0.8728474	nonsynonymous SNV	uc002coq.3:c.C1047G:p.F349L			0.966	D	2.88	chr16	2136253	C	G	941.9	0/0	0/1	0|1
62	*TSC2*	compound heterozygous	0.8728474	nonsynonymous SNV	uc002cop.3:c.G500A:p.R167Q	0.01	rs1800725	0.999	D	4.66	chr16	2110795	G	A	763.6	0/1	0/0	1|0
62	*TSC2*	compound heterozygous	0.8728474	nonsynonymous SNV	uc002coq.3:c.A149G:p.H50R	2.17E-04	rs184611539				chr16	2133636	A	G	529.7	0/0	0/1	0|1
28	*TSC22D1*	compound heterozygous	5.3432413	nonsynonymous SNV	uc001uzo.2:c.A1667G:p.Y556C	0.01	rs41288339				chr13	45008147	T	C	1662.3	0/0	0/1	0|1
28	*TSC22D1*	compound heterozygous	5.3432413	nonframeshift deletion	uc001uzn.4:c.1343_1345del:p.448_449del	0.01	rs201688574				chr13	45148865	TCCA	T	1810.7	0/1	0/0	1|0
3765	*TSHR*	deNovo	54.098844	nonsynonymous SNV	uc001xvb.1:c.G100A:p.E34K	1.87E-04	rs45499704	0.023	B	2.24	chr14	81422124	G	A	301.9	0/0	0/0	1|1
749	*TSHZ1*	compound heterozygous	5.0070771	nonsynonymous SNV	uc021uln.1:c.G1402A:p.A468T	0.02	rs33930274	0	B	-9.83	chr18	72998899	G	A	781	0/0	0/1	0|1
749	*TSHZ1*	compound heterozygous	5.0070771	nonsynonymous SNV	uc021uln.1:c.G2591A:p.R864Q	1.29E-03	rs150180967	0.977	D	4.83	chr18	73000088	G	A	736.3	0/1	0/0	1|0
2033	*TTC25*	homozygous rare	NA	nonsynonymous SNV	uc002hyj.4:c.C10T:p.P4S	0.02	rs34516580				chr17	40086986	C	T	3814.8	0/1	0/1	1|1
53	*TTC40*	compound heterozygous	NA	nonsynonymous SNV	uc021qbc.1:c.G5520A:p.M1840I	0.01	rs144154094	0.410823	NA	0.0344	chr10	134671148	C	T	1843.6	0/1	0/0	1|0
53	*TTC40*	compound heterozygous	NA	nonsynonymous SNV	uc021qbc.1:c.T5260C:p.S1754P	7.48E-04					chr10	134672690	A	G	563.8	0/0	0/1	0|1
313	*TTLL6*	homozygous rare	44.031611	frameshift deletion	uc021tzm.1:c.154_170del:p.52_57del	0.02					chr17	46882286	GCTTTCCAAAGTCGGGCA	G	11555.6	0/1	0/1	1|1
151	*TXNDC16*	homozygous rare	88.110403	nonsynonymous SNV	uc001wzs.3:c.A1691G:p.D564G	0.02	rs61744423	0.831	P	5	chr14	52923820	T	C	776.7	0/1	0/1	1|1
2035	*UBR1*	compound heterozygous	16.814107	nonsynonymous SNV	uc001zqq.3:c.A4834G:p.R1612G	5.45E-03	rs78948790	0.951	D	3.26	chr15	43250212	T	C	2869.9	0/1	0/0	1|0
2035	*UBR1*	compound heterozygous	16.814107	nonsynonymous SNV	uc001zqq.3:c.A2695G:p.I899V	0.02	rs35069201	0	B	-3	chr15	43317071	T	C	5287.2	0/0	0/1	0|1
313	*UBTFL1*	homozygous rare	NA	nonsynonymous SNV	uc010rub.2:c.C346T:p.P116S						chr11	89819463	C	T	1361.4	0/1	0/1	1|1
45	*UGT2B7*	homozygous rare	40.563812	nonframeshift substitution	uc003heg.4:c.801_802TC:UGT2B7						chr4	69964337	AT	TC	5163.8	0/1	0/1	1|1
69	*UGT2B7*	homozygous rare	40.563812	nonframeshift substitution	uc003heg.4:c.801_802TC:UGT2B7						chr4	69964337	AT	TC	1963.7	0/1	0/1	1|1
313	*UGT2B7*	homozygous rare	40.563812	nonframeshift substitution	uc003heg.4:c.801_802TC:UGT2B7						chr4	69964337	AT	TC	2844.3	0/1	0/1	1|1
2001	*UIMC1*	compound heterozygous	59.105921	nonsynonymous SNV	uc003mfd.2:c.T580C:p.Y194H	2.14E-03	rs115224789	0.99	D	5.56	chr5	176335682	A	G	6584.8	0/0	0/1	0|1
2001	*UIMC1*	compound heterozygous	59.105921	nonsynonymous SNV	uc021yil.1:c.T1058C:p.M353T	5.20E-04	rs143282828	0.009	B	-0.432	chr5	176395698	A	G	11345.9	0/1	0/0	1|0
3737	*VAV2*	compound heterozygous	2.5536683	nonsynonymous SNV	uc004cer.3:c.G2396A:p.R799Q	2.85E-04	rs138753594	0.997	D	4.63	chr9	136633640	C	T	360.1	0/1	0/0	1|0
3737	*VAV2*	compound heterozygous	2.5536683	nonsynonymous SNV	uc004cer.3:c.C2337G:p.I779M	0.01	rs61751477	0.031	B	0.847	chr9	136633699	G	C	584.9	0/0	0/1	0|1
54	*VCAN*	compound heterozygous	19.957537	nonsynonymous SNV	uc003kil.3:c.G241A:p.V81I	8.13E-06		0.877	D	3.44	chr5	82833071	G	A	830.1	0/1	0/0	1|0
54	*VCAN*	compound heterozygous	19.957537	nonsynonymous SNV	uc003kil.3:c.A596G:p.E199G	3.00E-03	rs61749614	0.01	B	1.67	chr5	82833426	A	G	399.5	0/0	0/1	0|1
7952	*VPS13A*	compound heterozygous	2.034678	nonsynonymous SNV	uc004akp.4:c.T2693C:p.V898A	0.02	rs78048112	0.006	B	2.43	chr9	79891006	T	C	5918.5	0/1	0/0	1|0
7952	*VPS13A*	compound heterozygous	2.034678	nonsynonymous SNV	uc004aks.3:c.T7340C:p.I2447T	1.55E-03	rs141138349	0.307	P	4.87	chr9	79968362	T	C	3403.8	0/0	0/1	0|1
3781	*VPS13B*	compound heterozygous	17.504128	nonsynonymous SNV	uc003yiv.3:c.G6337A:p.D2113N			0.931	D	5.39	chr8	100711968	G	A	2137.5	0/0	0/1	0|1
3781	*VPS13B*	compound heterozygous	17.504128	nonsynonymous SNV	uc003yiv.3:c.C8645T:p.P2882L	9.43E-04	rs145890213	0.121	B	4.45	chr8	100831065	C	T	1702.2	0/1	0/0	1|0
2812	*VPS39*	deNovo	11.276244	nonsynonymous SNV	uc001zpc.3:c.G574A:p.G192R			0.992	D	5.32	chr15	42476859	C	T	1134	0/0	0/0	0/1
2034	*VTN*	compound heterozygous	21.650153	nonsynonymous SNV	uc002hbc.3:c.A831C:p.K277N	2.44E-05	rs201356175	0.001	B	-9.54	chr17	26695690	T	G	2457.8	0/0	0/1	0|1
2034	*VTN*	compound heterozygous	21.650153	nonsynonymous SNV	uc002hbc.3:c.A377T:p.E126V	4.82E-03	rs112887300	0	B	1.03	chr17	26696680	T	A	599.4	0/1	0/0	1|0
1131	*WNK2*	compound heterozygous	NA	nonsynonymous SNV	uc004atk.3:c.C2975T:p.S992L		rs41296061				chr9	96051100	C	T	430.6	0/1	0/0	1|0
1131	*WNK2*	compound heterozygous	NA	nonsynonymous SNV	uc004atl.1:c.G118T:p.A40S	1.63E-05					chr9	96051369	G	T	355.6	0/0	0/1	0|1
17	*WWC3*	homozygous rare	85.851616	nonsynonymous SNV	uc004csx.4:c.G151A:p.E51K	5.15E-03	rs61748714	0.956	D	4.96	chrX	10035461	G	A	1042.1	0/1	0/1	1|1
3721	*XPO4*	compound heterozygous	12.974758	nonsynonymous SNV	uc001unq.4:c.A2695T:p.I899L	0.01	rs117470697	0	B	-1.86	chr13	21370317	T	A	2858.7	0/0	0/1	0|1
3721	*XPO4*	compound heterozygous	12.974758	nonsynonymous SNV	uc001unq.4:c.A446G:p.N149S	0.02	rs17320607	0.024	B	3.41	chr13	21429776	T	C	2963	0/1	0/0	1|0
2023	*ZFHX3*	compound heterozygous	0.1179523	nonsynonymous SNV	uc002fcl.3:c.G247T:p.A83S	0.01	rs2213978	0.733599	NA	4.77	chr16	72984595	C	A	1927	0/0	0/1	0|1
2023	*ZFHX3*	compound heterozygous	0.1179523	nonsynonymous SNV	uc002fck.3:c.C1667G:p.S556C	2.11E-04	rs147016640	0.312	P	4.89	chr16	72992378	G	C	1759.6	0/0	0/1	0|1
24	*ZFHX4*	compound heterozygous	2.435716	nonsynonymous SNV	uc003yaw.1:c.G196C:p.V66L	0.02	rs56261025				chr8	77616519	G	C	767.1	0/1	0/0	1|0
24	*ZFHX4*	compound heterozygous	2.435716	nonsynonymous SNV	uc003yaw.1:c.C7271T:p.P2424L	5.78E-03	rs61729528				chr8	77766563	C	T	1122.6	0/0	0/1	0|1
2020	*ZFPM2*	compound heterozygous	80.372729	nonsynonymous SNV	uc003ymd.3:c.A89G:p.E30G	2.68E-03	rs121908601				chr8	106431420	A	G	2960.2	0/0	0/1	0|1
2020	*ZFPM2*	compound heterozygous	80.372729	nonsynonymous SNV	uc011lhs.2:c.A1162G:p.S388G	0.01	rs28374544				chr8	106814279	A	G	1679.3	0/1	0/0	1|0
7952	*ZIC4*	compound heterozygous	50.224109	nonsynonymous SNV	uc003ewc.2:c.C296G:p.A99G	6.33E-03	rs62001026	0	B	0.0283	chr3	147113821	G	C	1229.6	0/1	0/0	1|0
7952	*ZIC4*	compound heterozygous	50.224109	nonsynonymous SNV	uc003ewc.2:c.G85C:p.G29R	6.90E-03	rs34676558	0.654	P	4.63	chr3	147114032	C	G	490.3	0/0	0/1	0|1
10	*ZKSCAN3*	homozygous rare	80.101439	nonframeshift substitution	uc003nlf.4:c.154_155GC:ZKSCAN3						chr6	28331127	AA	GC	1188.9	0/1	0/1	1|1
151	*ZNF154*	compound heterozygous	87.986553	stopgain SNV	uc010euf.3:c.C574T:p.R192X	8.78E-03	rs74939505	0.295211	NA	-2.03	chr19	58213743	G	A	994.9	0/0	0/1	0|1
151	*ZNF154*	compound heterozygous	87.986553	nonsynonymous SNV	uc010euf.3:c.G491A:p.S164N	6.60E-03	rs61738876	0.001	B	-0.452	chr19	58213826	C	T	1154.2	0/1	0/0	1|0
155	*ZNF212*	homozygous rare	71.597075	nonsynonymous SNV	uc003wfp.3:c.C877T:p.H293Y	0.02	rs34185245	0.777	P	-3.1	chr7	148950895	C	T	1422.8	0/1	0/1	1|1
8522	*ZNF236*	compound heterozygous	3.3498467	nonsynonymous SNV	uc002lmi.3:c.A1927G:p.S643G	0.02	rs3794873	0.028	B	5.3	chr18	74616374	A	G	4347.6	0/0	0/1	0|1
8522	*ZNF236*	compound heterozygous	3.3498467	nonsynonymous SNV	uc002lmi.3:c.A2345G:p.D782G	8.15E-04	rs200474825	0.002	B	1.95	chr18	74620329	A	G	316.8	0/1	0/0	1|0
2027	*ZNF251*	homozygous rare	21.650153	nonsynonymous SNV	uc003zdv.4:c.A605G:p.N202S	0.02	rs61732715				chr8	145948440	T	C	3399.3	0/1	0/1	1|1
3731	*ZNF318*	compound heterozygous	2.6185421	nonsynonymous SNV	uc003oux.3:c.C6728T:p.S2243F	1.50E-03	rs76571554	0.16	P	3.24	chr6	43305008	G	A	1767.3	0/0	0/1	0|1
3731	*ZNF318*	compound heterozygous	2.6185421	nonsynonymous SNV	uc003oux.3:c.A3883C:p.I1295L	1.38E-04	rs140429950	0.363	P	-0.228	chr6	43307853	T	G	3808.2	0/1	0/0	1|0
10	*ZNF407*	homozygous rare	9.3831092	nonsynonymous SNV	uc002llw.2:c.G6007A:p.E2003K	5.30E-03	rs150419019				chr18	72775684	G	A	990.8	0/1	0/1	1|1
17	*ZNF423*	compound heterozygous	1.3623496	nonsynonymous SNV	uc010vgn.2:c.G2986A:p.A996T	0.01	rs147898137	0	B	0.46	chr16	49669726	C	T	411.7	0/1	0/0	1|0
17	*ZNF423*	compound heterozygous	1.3623496	nonsynonymous SNV	uc010vgn.2:c.A1535G:p.N512S	0.02	rs34214571	0	B	4.32	chr16	49671177	T	C	742.1	0/0	0/1	0|1
2035	*ZNF518A*	compound heterozygous	NA	nonsynonymous SNV	uc001klp.3:c.G1477C:p.G493R	2.93E-03	rs41291602				chr10	97917556	G	C	843.4	0/1	0/0	1|0
2035	*ZNF518A*	compound heterozygous	NA	nonsynonymous SNV	uc001klp.3:c.A2435G:p.H812R	4.39E-03	rs149606048				chr10	97918514	A	G	1093.7	0/0	0/1	0|1
2023	*ZNF527*	homozygous rare	38.983251	nonframeshift substitution	uc002ogf.3:c.806_808TGTGCA:ZNF527						chr19	37879853	CAT	TGTGCA	6399.1	0/1	0/1	1|1
3721	*ZNF528*	homozygous rare	66.236141	nonsynonymous SNV	uc002pzh.3:c.G1328C:p.G443A	0.01	rs61978639	0.969	D	1.84	chr19	52919433	G	C	1892	0/1	0/1	1|1
3731	*ZNF572*	compound heterozygous	86.753951	nonsynonymous SNV	uc003yrr.3:c.A950C:p.K317T	0.02	rs10104558	0.96	D	4.39	chr8	125989460	A	C	1546.1	0/1	0/0	1|0
3731	*ZNF572*	compound heterozygous	86.753951	nonsynonymous SNV	uc003yrr.3:c.G1498A:p.V500I	0.01	rs7825375	0.031	B	3.45	chr8	125990008	G	A	1725.2	0/0	0/1	0|1
2023	*ZNF735*	homozygous rare	NA	nonframeshift substitution	ZNF735:uc011kdn.2:exon4:c.669_670TA						chr7	63680098	CC	TA	2714.9	0/1	0/1	1|1
2026	*ZNF735*	homozygous rare	NA	nonframeshift substitution	ZNF735:uc011kdn.2:exon4:c.669_670TA						chr7	63680098	CC	TA	2978.6	0/1	0/1	1|1
123	*ZNF735*	homozygous rare	NA	nonframeshift substitution	ZNF735:uc011kdn.2:exon4:c.669_670TA						chr7	63680098	CC	TA	1580.8	0/1	0/1	1|1
2001	*ZNF772*	homozygous rare	71.408351	nonframeshift insertion	uc002qot.3:c.12_13insGGC:p.A4delinsAA		rs34678661				chr19	57988666	A	AGCC	4617.8	0/1	0/1	1|1
2002	*ZNF772*	homozygous rare	71.408351	nonframeshift insertion	uc002qot.3:c.12_13insGGC:p.A4delinsAA		rs34678661				chr19	57988666	A	AGCC	2365.4	0/1	0/1	1|1
146	*ZNF772*	homozygous rare	71.408351	nonframeshift insertion	uc002qot.3:c.12_13insGGC:p.A4delinsAA		rs34678661				chr19	57988666	A	AGCC	2660	0/1	0/1	1|1
2008	*ZSCAN22*	compound heterozygous	30.820948	nonsynonymous SNV	uc002qsc.2:c.G1117T:p.V373L	8.21E-04	rs148862613	0.579	P	1.56	chr19	58850333	G	T	1003	0/0	0/1	0|1
2008	*ZSCAN22*	compound heterozygous	30.820948	nonsynonymous SNV	uc002qsc.2:c.G1141A:p.E381K	8.67E-03	rs138944686	0.762	P	0.286	chr19	58850357	G	A	917.6	0/1	0/0	1|0
