# Supplementary material for: De Novo and Rare Variants at Multiple Loci Support the Oligogenic Origins of Atrioventricular Septal Heart Defects
Source: PLoS Genet. 2016 Apr 8;12(4):e1005963. doi: 10.1371/journal.pgen.1005963 (PMC4825975; doi:10.1371/journal.pgen.1005963)
Supplement: S5 Table — (PDF) [file pgen.1005963.s012.pdf]

Table S5. 756 Genes Associated with Cardiac Malformations in Human Disease or Mouse Knockout Model

Known CHD Genes (Fahed et. al.)	Human Paralog of Genes with Mouse CHD Phenotype
ACP6	ABI1
ACTC	ABL1
ACVR1	ABRA
ACVR2B	ACE
ADAMTS2	ACKR3
ALDH1A2	ACTC1
ANKRD1	ACVR2A
BCL9	ACVRL1
CD160	ADAM10
CEP70	ADAM12
CFC1	ADAM15
CITED2	ADAM17
CNOT6	ADAM19
CRELD1	ADAMTS12
CSTB	ADAMTS9
CYFIP1	ADM
EDIL3	ADRBK1
EHMT1	AGER
ELN	AGTR1
FAIM	AGTR2
FAM62C	AHR
FDFT1	AIP
FKBP6	AKAP6
FLT4	ANGPT1
FMO5	ANGPT2
FOG2	ANGPTL4
FOXH1	ANGPTL6
FOXL2	ANKRD17
FSTL3	ANXA1
GATA4	ANXA2
GATA6	APC
GDF1	APH1A
GFPT2	APLNR
GJA1	APOE
GJA5	ARAP3
GJA8	ARHGEF11
GNA11	ARHGEF12
HAND2	ARHGEF15
HRAS	ARHGEF4
IRX4	ARID3B
JAG1	ARNT
LEFTY2	ARSB
MED13L	ATE1
MID1	ATP2A2
MIER2	AXIN1
MYH11	B4GALT1
MYH6	B9D1
MYH7	BAZ1B
NBPF11	BECN1
NEIL2	BICC1
NIPA1	BIRC2
NIPA2	BIRC3
NKX2-5	BIRC6
NKX2-6	BMP1
NODAL	BMP10
NOTCH1	BMP2
NPHP3	BMP4
NSD1	BMPR1A
PDGFRA	BRAF
PDZK1	BRCA1
PIK3CB	BTC
PPM1K	BVES
PRKAB2	C1GALT1
PTBP1	CACNB2
RAF1	CALR
S1PR4	CAPN2
SMAD6	CAPNS1
SOX7	CASP3
SSBP2	CASP7
TAB2	CASP8
TBX1	CASQ2
TBX20	CAV3
TBX5	CBS
TDGF1	CCDC88A
TFAP2B	CCL13
TMEM167A	CCM2
TMEM40	CCM2L
TNFSF11	CCND1
TUBGCP5	CCND2
VCAN	CCND3
VEGFA	CCNE1
WDR18	CCNE2
ZFPM2	CCNF
ZIC3	CD151
ZNF879	CD44
	CD47

CDH1
CDH13
CDH2
CDH5
CDK2
CDK4
CDKN1A
CFC1B
CFL1
CFLAR
CHD7
CHEK1
CHM
CHMP5
CHRD
CLEC4E
CLIC4
CLUAP1
COL18A1
COL2A1
COMMD3-BMI1
CRB2
CREBBP
CRHR2
CRKL
CSK
CSNK2A1
CSRP3
CTBP1
CTGF
CTNNB1
CTSS
CUL4B
CUL7
CXADR
CXCR3
CXCR4
CYP26A1
CYP51A1
CYR61
DAND5
DCBLD2
DCHS1
DDX17
DDX5
DES
DHRS3
DICER1
DISP1
DLC1
DLL1
DLL4
DNM1L
DNMT3B
DOCK1
DOT1L
DUSP6
DVL1
DVL2
DVL3
E2F7
E2F8
ECE1
ECE2
ECSCR
EDN1
EDNRA
EFNA1
EFNB2
EGFL7
EGFR
EGLN1
EGLN3
EGR1
ELAVL1
ENG
ENPP2
EP300
EPAS1
EPHA2
EPHB4
EPHX2
EPN1
EPN2
EPO
EPOR
ERBB2
ERBB3
ERF

ERG
ESAM
ETS1
ETS2
ETV2
ETV6
F2
F2R
F2RL1
F3
F7
FAT4
FBLN1
FBLN5
FBN1
FBXW7
FES
FGF10
FGF16
FGF19
FGF2
FGF8
FGF9
FGFR1
FGFR2
FGFRL1
FGR
FKBP1A
FKBP1B
FLI1
FLNA
FLRT3
FLT1
FLVCR1
FN1
FOSL1
FOXA2
FOXC1
FOXC2
FOXD3
FOXF1
FOXG1
FOXJ1
FOXM1
FOXO1
FOXP1
FOXP4
FRS2
FSTL1
FURIN
FXN
FZD1
FZD2
FZD4
FZD5
FZD7
GAB1
GAB2
GAS1
GATA1
GATA2
GATA5
GATAD2A
GBX2
GJC1
GNA13
GNAQ
GPC3
GPR124
GPR126
GPR4
GRB2
GSC
H2AFX
HAND1
HAS2
HBEGF
HCCS
HCK
HCN4
HDAC2
HDAC5
HDAC7
HEXIM1
HEY1
HEY2
HEYL
HGS
HHEX

HIF1A
HIF3A
HIRA
HIST1H1C
HIST1H1D
HIST1H1E
HK2
HOXA1
HOXA13
HOXA3
HOXB4
HPGD
HPRT1
HPSE
HSD17B7
HSP90B1
HSPB11
HSPB8
HTR1B
HTR2B
HUS1
ICAM1
ID1
ID3
IDH2
IDUA
IFNGR1
IFT172
IFT27
IFT57
IFT88
IGF1R
IGF2
IGF2R
IHH
IKBKAP
IL1A
IL1B
IL6
IL6ST
ILK
INSR
INTU
IRX3
IRX5
ISL1
ITGA5
ITGA6
ITGAV
ITGB1
ITGB3
ITGB4
ITGB5
ITGB8
ITPA
JMJD6
JUN
KAT2A
KAT6A
KAT7
KCNH2
KDM6A
KDM8
KDR
KIDINS220
KIF3A
KIF3B
KIFAP3
KIT
KLF15
KLF5
KLF6
KRAS
KRIT1
KRT1
LATS2
LBX1
LDB1
LEF1
LIG3
LIMS1
LIMS2
LIN28A
LMNA
LMO2
LRP5
LUZP1
LY6E
MAML1

MAML3
MAP2K5
MAP3K3
MAP3K7
MAPK1
MAPK11
MAPK12
MAPK14
MAPK3
MAPK7
MB
MCAM
MDM4
MECOM
MED1
MED12
MED23
MED24
MEF2C
MEGF8
MEIS1
MEN1
MEOX2
MESP1
METAP2
MGAT1
MGP
MIB1
MIXL1
MKL1
MKL2
MLST8
MMP13
MMP14
MMP2
MMP9
MRE11A
MUS81
MYB
MYBPC3
MYC
MYCN
MYH10
MYH14
MYH9
MYL2
MYL7
MYLK3
MYOCD
MYOZ2
NCOA6
NCOR2
NCSTN
NDST1
NEK8
NFATC1
NFATC3
NFATC4
NGFR
NOG
NOS1
NOS2
NOS3
NOTCH3
NOTCH4
NOV
NPM1
NPRL3
NR2F2
NRARP
NRP1
NRP2
NTF3
NTRK3
NUMB
NXN
OFD1
OSR1
OVOL2
PAK4
PALB2
PAM
PARVA
PATZ1
PAX1
PAX3
PAXIP1
PBRM1
PBX1

PCGF2
PCSK6
PDCD10
PDGFA
PDGFC
PDGFRB
PDPK1
PDPN
PDS5B
PFKM
PFN1
PGF
PHC1
PIFO
PIK3CA
PITX2
PKD1
PKD2
PKP2
PLAT
PLCE1
PLCG1
PLD1
PLG
PLVAP
PLXND1
PNN
PNPLA2
PNPLA6
POFUT1
POPDC2
POR
POSTN
PPAP2B
PPARG
PPARGC1A
PPP3R1
PRDM1
PRDM6
PRKAR1A
PRKCI
PROC
PROS1
PRRX1
PRRX2
PSEN1
PSEN2
PTCH1
PTEN
PTGER4
PTGS1
PTGS2
PTH
PTHLH
PTK2
PTK2B
PTPN11
PTPN12
PTPRB
PTPRJ
PXN
RAC1
RAC2
RAD23B
RAMP2
RAP1A
RAPGEF2
RARA
RARB
RARG
RASA1
RASA3
RASIP1
RB1
RBL1
RBL2
RBP4
RBPJ
RDH10
RECK
REV3L
RFX3
RHEB
RHOB
RHOJ
RIPK3
RIPPLY3
ROBO1
ROBO2

ROBO4
ROR1
RUNX1
RUNX2
RXRA
RXRB
RYR1
S1PR1
S1PR2
S1PR3
SALL1
SALL4
SCXA
SDC4
SELL
SEMA3C
SEMA3D
SEMA5A
SERPINE1
SERPINF1
SETD2
SHC1
SHH
SHOC2
SHOX2
SIRT1
SIX1
SLC20A1
SLC34A2
SLC6A4
SLC8A1
SLCO2A1
SMAD1
SMAD2
SMAD3
SMAD4
SMAD5
SMAD7
SMAD9
SMARCA4
SMN1
SMO
SMYD1
SNAI1
SOS1
SOX11
SOX12
SOX4
SOX9
SPEG
SPEN
SPHK1
SPHK2
SPP1
SPTBN1
SRF
SRSF10
STK11
STK4
SUFU
TAB1
TAL1
TAX1BP1
TBX18
TBX2
TBX3
TBX4
TBX6
TCF21
TCTN2
TEAD1
TEAD2
TEK
TFAM
TFAP2A
TGFB1
TGFB2
TGFB3
TGFBR1
TGFBR2
TGFBR3
TGIF1
TGIF2
TH
THBD
THBS1
THBS2
TIE1
TIMP3

TLL1
TLR2
TMEM100
TMEM38A
TMEM38B
TMOD1
TNFRSF1A
TNNI3
TNNT2
TP53
TRA2B
TSC1
TSC2
TWIST2
TXNRD2
UBP1
UBR1
UBR2
UBR4
UBR5
UNC5B
USP8
VANGL2
VASH1
VASH2
VAV2
VAV3
VCAM1
VCL
VDR
VEZF1
VHL
VIM
VPS52
WASF2
WDPCP
WDR83
WHSC1
WNT11
WNT3A
WNT7A
WNT7B
WRN
WT1
XBP1
XIAP
YAP1
YWHAE
ZFP36L1
ZFPM1
ZMIZ1
