# Supplementary material for: De Novo and Rare Variants at Multiple Loci Support the Oligogenic Origins of Atrioventricular Septal Heart Defects
Source: PLoS Genet. 2016 Apr 8;12(4):e1005963. doi: 10.1371/journal.pgen.1005963 (PMC4825975; doi:10.1371/journal.pgen.1005963)
Supplement: S6 Table — (PDF) [file pgen.1005963.s013.pdf]

Table S6. A Co-Expression Module of 934 Genes Includes *NKX2-5, CRELD1,* and *GATA4* is Enriched for Genes Associated with Cardiac Malformations

AADACL3
AAR2
ABI3
ACKR2
ACOT4
ACP6
ACTN4
ACTR1B
ACVR1
ACVR2B
ACYP2
ADAM10
ADAMTSL4
ADRBK2
AFAP1
AFAP1L1
AGAP3
AGBL1
AGMO
AGO4
AGPAT3
AGPAT6
AIDA
AIPL1
AJUBA
AK4
AKR1B1
AKT1S1
ALDH1L2
ALG5
ALKBH4
AMOTL2
ANK1
ANK3
ANKAR
ANKFY1
ANKH
ANKHD1-EIF4EBP3
ANKRD1
ANKRD13A
ANKRD17
ANKRD28
ANKRD32
ANKRD39

ANKRD40
ANKZF1
ANO6
ANP32A
ANXA5
ANXA6
APBA1
APBB1
APEH
APH1A
APLP2
ARCN1
ARHGAP10
ARHGAP29
ARHGEF26
ARID1A
ARIH1
ARIH2
ARL4D
ARMC1
ARMC2
ARMCX2
ASCC2
ASCC3
ATF1
ATF3
ATF7IP
ATG12
ATG5
ATL2
ATOH8
ATP1A2
ATP1B1
ATP1B2
ATP5G2
ATP6AP1
ATP6V0E1
ATP7A
ATP7B
B3GALT6
B3GNT7
BACH2
BAD
BAG6
BAHD1
BAMBI
BCHE

BCORL1
BFAR
BHLHE41
BLOC1S2
BLOC1S6
BMP5
BMPER
BRD4
BRD7
BRD9
BTBD11
BTRC
BUB1
BUB1B
BVES
C10orf118
C11orf84
C12orf75
C14orf2
C16orf45
C17orf49
C18orf25
C19orf44
C1orf198
C3orf20
C3orf80
C6orf222
C6orf62
C9orf84
CALR3
CALU
CAMK2G
CANT1
CAPG
CASP2
CBFB
CBX1
CC2D1A
CCAR1
CCDC146
CCDC166
CCDC28A
CCDC40
CCDC88A
CCDC88B
CCDC91
CCDC92

CCL27
CCNY
CD151
CD1D
CD320
CD68
CDAN1
CDC14B
CDC42EP5
CDCA2
CDH20
CDK16
CDKN1B
CDKN2AIP
CDYL2
CELF1
CENPE
CEP55
CEP78
CERK
CHD2
CHMP6
CHPF
CHPF2
CHST10
CHTF8
CKAP5
CLN8
CLPTM1L
CNOT11
CNOT7
CNPPD1
COL4A6
COL9A3
COLGALT2
COMMD10
COPA
COPB2
COPS5
COQ10B
CORIN
COX15
COX19
COX5B
CREB3
CREB3L2
CRELD1

CSNK1D
CSNK1G1
CTDSP2
CTHRC1
CTNNBL1
CTNND1
CTTN
CTTNBP2NL
CXCR4
CXXC5
CYHR1
DAG1
DBF4
DBI
DCAF12
DCAF12L2
DDN
DDOST
DDR1
DDX20
DDX3Y
DENND2C
DGKZ
DHRS13
DICER1
DLGAP4
DLST
DNMT3A
DOC2A
DOCK1
DOCK11
DONSON
DPH2
DPPA4
DPY30
DPYSL2
DZANK1
E2F3
EARS2
ECI2
EDIL3
EEF1A2
EEPD1
EFCAB14
EFNB1
EGLN1
EIF2S3

EIF5B
ELAC1
ELL2
ELOVL5
ELOVL6
EMC1
EMC4
EMILIN1
EMILIN2
ENPP1
ENY2
EPHA7
EPHB3
ERO1L
ESPL1
ESR2
ESYT1
ESYT2
ETS1
ETV2
EXOSC2
FAM122B
FAM161B
FAM168A
FAM168B
FAM173A
FAM173B
FAM181A
FAM198B
FAM205A
FAM20B
FAM222B
FAM26E
FAM53C
FAM73A
FARSA
FARSB
FAT4
FBLN2
FBN2
FBXO18
FBXO30
FBXO4
FBXO40
FBXO42
FBXO43
FBXW11

FBXW2
FCHSD2
FEM1A
FGFR1OP2
FGL2
FIP1L1
FITM1
FKBP10
FKBP1A
FKBP2
FLNB
FN1
FNDC3B
FOPNL
FOXB2
FOXK2
FOXN3
FSTL4
FTCD
FUNDC2
FURIN
FYN
G3BP2
GAB1
GALNS
GATA4
GATA5
GATA6
GDAP2
GDF6
GDI2
GFOD1
GFPT2
GFRA3
GGPS1
GHITM
GID8
GIGYF1
GIPC1
GLCCI1
GLIPR1L2
GLRX2
GMIP
GMPPB
GNB3
GOLGA4
GOLIM4

GPANK1
GPATCH8
GPD1L
GPR108
GPR182
GRAP2
GRIK4
GRIP2
GSK3B
GTF2E2
GXYLT1
HAUS8
HCFC2
HCN4
HDAC10
HEATR5A
HEATR5B
HEG1
HELZ2
HESX1
HEY1
HHAT
HIC2
HIVEP3
HLA-DMA
HMG20A
HMG20B
HMGB3
HMGCR
HMGXB4
HNRNPA2B1
HNRNPM
HNRNPUL1
HOXD8
HS1BP3
HS6ST2
HSDL2
HSPA2
HSPG2
HTR2B
ICK
ID1
IFNGR2
IGF1
IGF1R
IGF2BP3
IGFBP5

IGSF3
IGSF9
IMPA2
IMPAD1
IMPG1
INO80
INO80B
INO80C
INPPL1
INTS10
IPO4
IRF2
IRF2BP2
IRF2BPL
IRF3
IRX4
ITM2C
ITPR1
JAKMIP1
JPH3
KARS
KCNG1
KCNJ13
KCNJ3
KCNJ4
KCTD6
KDM2A
KDM6B
KDR
KIAA0930
KIAA1107
KIAA1239
KIAA1919
KIAA2026
KIF26B
KITLG
KLC2
KLHL31
KRT9
KRTAP13-1
LACC1
LACTB
LAMB2
LAPTM4B
LARP4B
LASP1
LATS1

LCA5
LDLRAD2
LDLRAD3
LENEP
LGALSL
LIMD2
LMBRD2
LMF2
LNX1
LRIF1
LRIG2
LRRN4
LUC7L
LUC7L2
LXN
LZTS2
MAFG
MAGT1
MAN1A1
MAP10
MAP2K1
MAP3K1
MAP3K2
MAP7D1
MAPK8IP1
MAPK9
MAPRE3
MARCH6
MARCH8
MARCKS
MARCKSL1
MAZ
MCMBP
MDM2
ME2
MECOM
MEGF9
MEN1
MEOX1
MESP1
MFSD10
MGAT4B
MIPEP
MKL2
MMP27
MON2
MPDZ

MPV17
MRAP
MROH1
MRPL1
MRPL10
MRPL12
MRPL16
MRPL51
MRPS11
MRPS23
MSL2
MT3
MTERFD3
MTHFD1L
MTMR12
MTMR3
MTMR9
MYCBPAP
MYH10
MYLIP
NCAM1
NCK2
NCL
NCOR1
NDC1
NDUFA3
NDUFAF7
NDUFB3
NDUFS5
NEDD4
NEIL3
NELFA
NFIA
NFKBIL1
NHSL1
NINJ1
NINL
NKX2-5
NLK
NLRP5
NOA1
NOG
NOX1
NOXRED1
NPEPPS
NPFF
NPLOC4

NR0B2
NR1D1
NR1D2
NR3C2
NRP1
NUDT17
NUP160
NUP88
OGDH
OPLAH
OSBPL2
OSBPL6
OSBPL8
OSGIN1
OSTM1
OXCT1
PABPC1
PABPC4
PANK4
PAOX
PAPPA
PAPSS2
PARG
PARM1
PAX2
PAXBP1
PCBP4
PCDHGA8
PCNX
PCNXL4
PDCD6IP
PDHX
PDK4
PDS5B
PEBP1
PEX1
PEX11B
PFN1
PGF
PHF8
PIGU
PIP5K1A
PITPNB
PLCH1
PLCZ1
PLEK
PLEK2

PLEKHA3
PLEKHA4
PLEKHA8
PLEKHB2
PLEKHG2
PLEKHM3
PLEKHO1
PLK4
PLXNC1
PM20D2
PMM1
PMP22
PNMT
POLR2D
POU2F1
PPAT
PPID
PPM1L
PPP1R11
PPP1R17
PPP1R36
PPP1R3A
PPP1R9A
PPP2R3A
PPP4R2
PRDM6
PREB
PRIMPOL
PRKAR1A
PRKAR2A
PRKCB
PRPF4B
PRPSAP1
PRR11
PRR12
PRR18
PRRC2B
PRRX2
PSMA6
PSME4
PTMS
PTOV1
PTP4A3
PTPN23
PTPRB
PVRL1
PWWP2A

PXDN
PXMP2
PYROXD1
QKI
R3HCC1
R3HCC1L
RAB11FIP3
RAB2A
RAB2B
RAB31
RAB3IP
RAB43
RAB4A
RAB8A
RAD23A
RAF1
RAI14
RAP1GDS1
RAPGEF1
RASIP1
RASSF1
RASSF3
RB1CC1
RBBP7
RBM20
RBM28
RBM4
RBM44
RBM8A
RBMS1
RCHY1
RCN3
REPS1
RFT1
RGL1
RGS12
RGS6
RHBDF1
RHOB
RHOBTB2
RIMBP2
RMND5B
RNF138
RNF14
RNF141
RNF25
RPA2

RPS12
RPS15
RPS25
RPS6KA3
RPS9
RRAS
RRAS2
RSBN1L
RWDD1
RXRB
RYR3
SAR1A
SBNO2
SCAF1
SCAF11
SCAMP3
SCO2
SCPEP1
SDCCAG3
SEC14L1
SEPT1
SERAC1
SETD3
SETD5
SETD8
SF3A1
SFXN4
SGSM2
SH2D2A
SH3TC2
SHMT2
SHOC2
SIGLEC15
SIK3
SIPA1L1
SIX5
SKIL
SLAIN2
SLC12A2
SLC13A4
SLC1A3
SLC1A6
SLC24A3
SLC24A4
SLC25A25
SLC25A3
SLC25A34

SLC25A37
SLC30A9
SLC35B4
SLC35E1
SLC36A4
SLC37A3
SLC38A10
SLC39A1
SLC39A10
SLC39A7
SLC52A2
SLC5A11
SLC7A7
SLFN14
SLTM
SMAD6
SMARCA5
SMARCD2
SMIM19
SNRPA
SOAT1
SOCS3
SOCS5
SOX13
SOX9
SPATA24
SPATA5
SPDYA
SPHKAP
SPOCD1
SPPL2A
SPRYD3
SRA1
SREBF2
ST3GAL5
STAP2
STAT3
STAT5A
STIM2
STK24
STK3
STRBP
STRN4
STUB1
SUPT3H
SZT2
TACC3

TANC2
TAOK1
TBCCD1
TBX18
TCEANC2
TCFL5
TCHP
TCTA
TEAD1
TECRL
TESK1
TEX261
TFEB
TGFB2
TGFBR1
TGFBRAP1
THAP11
THAP3
THAP7
THBS1
THBS4
THOC7
THUMPD1
THYN1
TIGD4
TK1
TLDC1
TLE3
TM2D2
TM4SF19
TM6SF1
TM9SF1
TM9SF3
TMA7
TMBIM1
TMBIM4
TMC2
TMCO1
TMED1
TMED2
TMED7
TMEM101
TMEM108
TMEM115
TMEM123
TMEM127
TMEM129

TMEM138
TMEM147
TMEM150A
TMEM161B
TMEM163
TMEM165
TMEM167A
TMEM168
TMEM17
TMEM170A
TMEM170B
TMEM178B
TMEM186
TMEM19
TMEM194B
TMEM2
TMEM200B
TMEM206
TMEM215
TMEM234
TMEM242
TMEM258
TMEM261
TMEM30A
TMEM38A
TMEM38B
TMEM55A
TMEM63B
TMEM64
TMEM65
TMEM66
TMEM71
TMEM86A
TMEM87A
TMEM88
TMEM98
TMEM9B
TMF1
TMLHE
TMOD1
TMPO
TMTC3
TMX3
TMX4
TNFSF11
TNIK
TNIP2

TNKS
TNNI3
TNNI3K
TOM1L1
TP53
TP53BP2
TP53INP2
TPCN1
TPX2
TRAK2
TREML2
TRIB2
TRIM5
TRIM55
TRIM62
TRIM63
TRIM68
TRIM8
TRIP10
TRMT2A
TSC22D1
TSC22D4
TSPAN12
TSPAN13
TSPAN9
TTC23
TTI1
TTK
TULP1
TWIST1
TWISTNB
U2AF2
U2SURP
UBE2D1
UBE2J2
UBE2R2
UBE4A
UBFD1
UBL3
UBN2
UBOX5
UBR4
UBXN2A
UGP2
UNC119
UPF3A
UPK3B

URB1
UTP20
VASH1
VCL
VCPKMT
VOPP1
WASH1
WBP1L
WDR13
WDR41
WDR82
WDR91
WDSUB1
WHSC1L1
WIPF2
WNK1
WTIP
WWC2
XIRP1
XK
XKR4
YEATS4
ZBED3
ZBTB24
ZBTB40
ZBTB45
ZBTB6
ZC4H2
ZCWPW2
ZEB1
ZFP36L1
ZFP36L2
ZFX
ZFYVE20
ZMAT5
ZMYM4
ZNF22
ZNF260
ZNF275
ZNF563
ZNF579
ZNF768
ZNF799
ZSCAN22
