# Supplementary material for: De Novo and Rare Variants at Multiple Loci Support the Oligogenic Origins of Atrioventricular Septal Heart Defects
Source: PLoS Genet. 2016 Apr 8;12(4):e1005963. doi: 10.1371/journal.pgen.1005963 (PMC4825975; doi:10.1371/journal.pgen.1005963)
Supplement: S7 Table — (PDF) [file pgen.1005963.s014.pdf]

Table S7. Rare Variant Inheritance Patterns Identify Genes Associated with Cardiac Malformations by Prior Clinical or Experimental Evidence

Prior Evidence	Proband	Gene	Inheritance	Residual Variation Intolerance Score	Exonic Function	Amino Acid Change	Minor Allele Frequency EXAC	dbSNP138	PolyPhen2 Score	PolyPhen2 Prediction	GERP Score	Chromosome	Position	Ref	Alt	Quality Score	Paternal Genotype	Maternal Genotype	Proband Genotype
Mouse KO Phenotype	3721	*ADAM17*	compound heterozygous	82.78	nonsynonymous SNV	uc010ewz.3:c.T290G:p.L97R	.	.	0.003	B	1.61	chr2	9630520	A	C	2144.5	0/0	0/1	0|1
Mouse KO Phenotype	3721	*ADAM17*	compound heterozygous	82.78	nonsynonymous SNV	uc010ewz.3:c.C269T:p.S90L	0.01	rs55796712	0.003	B	1.66	chr2	9630541	G	A	2781.7	0/1	0/0	1|0
Mouse KO Phenotype	1131	*RYR1*	compound heterozygous	0.01	nonsynonymous SNV	uc002oit.3:c.A89T:p.E30V	2.37E-04	rs145771708	0.158	P	4.41	chr19	38931428	A	T	602.1	0/1	0/0	1|0
Mouse KO Phenotype	1131	*RYR1*	compound heterozygous	0.01	nonsynonymous SNV	uc002oiu.3:c.G13498C:p.D4500H	3.31E-03	rs150396398	0.456933	.	3.76	chr19	39057626	G	C	1248.1	0/0	0/1	0|1
Mouse KO Phenotype	7952	*CHRD*	compound heterozygous	15.36	nonsynonymous SNV	uc011brr.2:c.C476T:p.A159V	5.29E-04	rs150401834	0.251	P	5.28	chr3	184102470	C	T	765.7	0/0	0/1	0|1
Mouse KO Phenotype	7952	*CHRD*	compound heterozygous	15.36	nonsynonymous SNV	uc011brr.2:c.C1085T:p.T362I	2.36E-04	rs138569844	0.992	D	3.88	chr3	184105726	C	T	479.8	0/1	0/0	1|0
Mouse KO Phenotype	3731	*PTPRJ*	compound heterozygous	86.42	nonsynonymous SNV	uc001ngo.4:c.G1114A:p.V372I	8.80E-03	rs2229703	0.014	B	-10.8	chr11	48149352	G	A	632.1	0/0	0/1	0|1
Mouse KO Phenotype	3731	*PTPRJ*	compound heterozygous	86.42	nonsynonymous SNV	uc010rhr.1:c.G517A:p.E173K	4.07E-04	rs181029182	0.881	D	0.0462	chr11	48161067	G	A	1410	0/1	0/0	1|0
Mouse KO Phenotype	8522	*IFT140*	compound heterozygous	22.82	nonsynonymous SNV	uc002clz.3:c.G3193A:p.E1065K	4.07E-05	rs142106374	0.615491	.	3.85	chr16	1560980	C	T	732.4	0/0	0/1	0|1
Mouse KO Phenotype	8522	*IFT140*	compound heterozygous	22.82	nonsynonymous SNV	uc002cmb.3:c.G322A:p.V108M	6.86E-03	rs146128830	0.03	B	-1.38	chr16	1652418	C	T	1146.8	0/1	0/0	1|0
Mouse KO Phenotype	28	*ATE1*	homozygous rare	22.51	nonsynonymous SNV	uc010qts.2:c.C211T:p.L71F	0.01	rs148095496	0.001	B	1.57	chr10	123670505	G	A	2325.6	0/1	0/1	1|1
Clinical Association with CHD	2933	*NOTCH1*	compound heterozygous	0.33	nonsynonymous SNV	uc004chz.3:c.G6853A:p.V2285I	0.02	rs61751489	0	B	1.38	chr9	139391338	C	T	621.8	0/1	0/0	1|0
Clinical Association with CHD	2033	*NOTCH1*	compound heterozygous	0.33	nonsynonymous SNV	uc004cia.1:c.G1526A:p.R509H	0.01	rs61751543	0.874	D	3.49	chr9	139401233	C	T	541.2	0/0	0/1	0|1
Clinical Association with CHD	155	*NSD1*	compound heterozygous	3.24	nonsynonymous SNV	uc003mfs.1:c.G2797C:p.A933P	0.02	rs28932179	0.004	B	-8.89	chr5	176638506	G	C	1166.6	0/0	0/1	0|1
Clinical Association with CHD	155	*NSD1*	compound heterozygous	3.24	nonsynonymous SNV	uc021yip.1:c.A1083T:p.R361S	1.63E-05	rs377546578	0.405	P	3.13	chr5	176721980	A	T	236.5	0/1	0/0	1|0
Clinical Association with CHD	2020	*ZFPM2*	compound heterozygous	80.37	nonsynonymous SNV	uc003ymd.3:c.A89G:p.E30G	2.68E-03	rs121908601	.	.	.	chr8	106431420	A	G	2960.2	0/0	0/1	0|1
Clinical Association with CHD	2020	*ZFPM2*	compound heterozygous	80.37	nonsynonymous SNV	uc011lhs.2:c.A1162G:p.S388G	0.01	rs28374544	.	.	.	chr8	106814279	A	G	1679.3	0/1	0/0	1|0
Clinical Association with CHD	2023	*MYH6*	compound heterozygous	0.66	nonsynonymous SNV	uc001wjv.3:c.C2807T:p.A936V	4.55E-04	rs199838024	0.937	D	4.7	chr14	23862996	G	A	1709.8	0/1	0/0	1|0
Clinical Association with CHD	2023	*MYH6*	compound heterozygous	0.66	nonsynonymous SNV	uc001wjv.3:c.G622A:p.D208N	5.49E-03	rs142027794	0	B	3.17	chr14	23873940	C	T	2503.7	0/0	0/1	0|1
Clinical Association with CHD	54	*VCAN*	compound heterozygous	19.96	nonsynonymous SNV	uc003kil.3:c.G241A:p.V81I	8.13E-06	.	0.877	D	3.44	chr5	82833071	G	A	830.1	0/1	0/0	1|0
Clinical Association with CHD	54	*VCAN*	compound heterozygous	19.96	nonsynonymous SNV	uc003kil.3:c.A596G:p.E199G	3.00E-03	rs61749614	0.01	B	1.67	chr5	82833426	A	G	399.5	0/0	0/1	0|1
Clinical Association with CHD	54	*MYH6*	homozygous rare	0.66	nonsynonymous SNV	uc001wjv.3:c.G3883C:p.E1295Q	3.21E-03	rs34935550	0.738	P	4.58	chr14	23858697	C	G	2365.8	0/1	0/1	1|1
CHD Associated Syndrome	7952	*SRCAP*	compound heterozygous	0.15	nonsynonymous SNV	uc002dzg.1:c.G1130A:p.S377N	8.05E-04	rs139339184	0.508889	.	3.72	chr16	30723222	G	A	1361.6	0/0	0/1	0|1
CHD Associated Syndrome	7952	*SRCAP*	compound heterozygous	0.15	nonsynonymous SNV	uc002dzg.1:c.A5090G:p.E1697G	4.02E-03	rs117480926	0.562115	.	5.35	chr16	30740333	A	G	1082.9	0/1	0/0	1|0
CHD Associated Syndrome	2002	*MLL2*	compound heterozygous	0.06	nonsynonymous SNV	uc001rta.4:c.G1955A:p.R652H	3.26E-05	.	.	.	.	chr12	49445511	C	T	222.5	0/1	0/0	1|0
CHD Associated Syndrome	2002	*MLL2*	compound heterozygous	0.06	nonsynonymous SNV	uc001rta.4:c.G248A:p.R83Q	0.02	rs55865069	.	.	.	chr12	49448463	C	T	656.4	0/0	0/1	0|1
CHD Associated Syndrome	155	*NOTCH2*	compound heterozygous	2.15	nonsynonymous SNV	uc001eik.3:c.T7223A:p.L2408H	1.77E-03	rs35586704	0.688	P	5	chr1	120458122	A	T	1173.5	0/0	0/1	0|1
CHD Associated Syndrome	155	*NOTCH2*	compound heterozygous	2.15	nonsynonymous SNV	uc001eik.3:c.A3980G:p.D1327G	0.01	rs61752484	0.067	B	5.47	chr1	120469147	T	C	1652.1	0/1	0/0	1|0
CHD Associated Syndrome	135	*BBS2*	compound heterozygous	11.36	nonsynonymous SNV	uc002ejd.2:c.G2062A:p.G688R	.	rs376063241	0.996	D	5.14	chr16	56518777	C	T	535.6	0/0	0/1	0|1
CHD Associated Syndrome	135	*BBS2*	compound heterozygous	11.36	frameshift insertion	uc002ejd.2:c.185_186insC:p.L62fs	.	.	.	.	.	chr16	56548525	A	AG	614.6	0/1	0/0	1|0
CHD Associated Syndrome	3731	*BBS2*	compound heterozygous	11.36	nonsynonymous SNV	uc002ejd.2:c.C1511T:p.A504V	6.12E-03	rs16957538	0	B	2.91	chr16	56533706	G	A	1455.9	0/0	0/1	0|1
CHD Associated Syndrome	3731	*BBS2*	compound heterozygous	11.36	nonsynonymous SNV	uc002ejd.2:c.A691G:p.K231E	1.63E-05	.	0.168	P	4.29	chr16	56540058	T	C	2175.3	0/1	0/0	1|0
CHD Associated Syndrome	2028	*EHMT1*	*deNovo*	3.13	nonsynonymous SNV	uc004cob.1:c.G779A:p.R260Q	.	.	0.999	D	5.07	chr9	140637871	G	A	1671.7	0/0	0/0	0/1
