# Supplementary material for: De Novo and Rare Variants at Multiple Loci Support the Oligogenic Origins of Atrioventricular Septal Heart Defects
Source: PLoS Genet. 2016 Apr 8;12(4):e1005963. doi: 10.1371/journal.pgen.1005963 (PMC4825975; doi:10.1371/journal.pgen.1005963)
Supplement: S8 Table — (PDF) [file pgen.1005963.s015.pdf]

Table S8. 3595 Genes Expressed in Cardiac Development by Weighted Gene Coexpression Analysis

Gene	Atrial Expression	p-value Atrial	AV-Canal Expression	p-value AV-Canal	Branchial Arches Expression	p-value Branchial Arches	Heart Tube Expression	p-value Heart Tube	Outflow Tract Expression	p-value Outflow Tract	Ventricle Expression	p-value Ventricle	DMP Expression
AAED1	.	.	.	.	.	.	.	.	0.6302173	1.78E-09	.	.	.
AAMP	.	.	0.3092181	0.0073457	.	.	.	.	.	.	.	.	yes
AANAT	.	.	0.5338249	9.69E-07	.	.	.	.	.	.	.	.	.
AAR2	.	.	0.4731521	2.07E-05	.	.	.	.	.	.	.	.	.
AARS	.	.	.	.	.	.	.	.	.	.	.	.	yes
ABCE1	.	.	.	.	.	.	.	.	.	.	.	.	yes
ABCF1	.	.	.	.	.	.	.	.	.	.	.	.	yes
ABHD17A	.	.	.	.	.	.	.	.	.	.	0.6119169	6.94E-09	.
ABI3	.	.	.	.	.	.	0.5534728	3.15E-07	.	.	.	.	.
ABI3BP	.	.	.	.	.	.	.	.	.	.	0.4766818	1.76E-05	.
ABL1	.	.	.	.	.	.	.	.	.	.	.	.	yes
ABRA	.	.	.	.	.	.	0.5904727	3.07E-08	0.5336702	9.77E-07	.	.	.
ABRACL	.	.	0.4014572	0.0003925	.	.	.	.	0.4494966	5.88E-05	.	.	.
ABT1	.	.	.	.	.	.	.	.	0.5969099	1.99E-08	.	.	.
ABTB1	.	.	0.3982195	0.0004417	.	.	.	.	.	.	.	.	.
ABTB2	.	.	0.5006013	5.57E-06	.	.	.	.	0.6444781	5.79E-10	.	.	.
ACADVL	.	.	.	.	.	.	.	.	0.4410344	8.39E-05	.	.	.
ACIN1	.	.	.	.	.	.	.	.	.	.	.	.	yes
ACLY	.	.	.	.	.	.	.	.	.	.	.	.	yes
ACMSD	.	.	.	.	.	.	.	.	0.6355215	1.18E-09	.	.	.
ACO2	.	.	.	.	.	.	0.425502	0.0001573	.	.	.	.	yes
ACOT1	.	.	.	.	0.4937607	7.81E-06	.	.	.	.	.	.	.
ACOT11	.	.	.	.	.	.	.	.	.	.	0.6640416	1.12E-10	.
ACOT13	.	.	0.3599381	0.0016334	.	.	.	.	.	.	0.5649999	1.57E-07	.
ACOT2	.	.	0.410779	0.0002776	.	.	.	.	.	.	.	.	.
ACP6	.	.	0.4397643	8.84E-05	.	.	.	.	.	.	.	.	.
ACSF2	.	.	.	.	.	.	.	.	0.4997799	5.80E-06	.	.	.
ACSL4	.	.	.	.	.	.	.	.	.	.	0.5967692	2.01E-08	.
ACSM4	.	.	.	.	.	.	.	.	0.7666312	1.70E-15	.	.	.
ACTA2	.	.	.	.	.	.	.	.	.	.	.	.	yes
ACTB	.	.	.	.	.	.	.	.	.	.	.	.	yes
ACTC1	.	.	0.475112	1.89E-05	.	.	0.3883608	0.0006281	0.4286284	0.0001389	.	.	yes
ACTN1	.	.	.	.	.	.	.	.	0.5975862	1.90E-08	.	.	yes
ACTN2	.	.	.	.	.	.	.	.	0.7882325	7.76E-17	.	.	.
ACTN4	.	.	.	.	.	.	.	.	.	.	.	.	yes
ACTR3	.	.	.	.	.	.	.	.	0.5227694	1.77E-06	.	.	.
ACTR3B	.	.	.	.	.	.	.	.	.	.	0.5174221	2.35E-06	.
ACVR1B	.	.	.	.	.	.	.	.	0.7463459	2.31E-14	.	.	.
ADAM10	.	.	0.5658738	1.49E-07	.	.	.	.	.	.	.	.	.
ADAM12	.	.	.	.	.	.	.	.	0.7188474	5.50E-13	.	.	.
ADAM19	.	.	0.479859	1.52E-05	.	.	.	.	.	.	.	.	.
ADAMTS15	.	.	0.4205725	0.0001908	.	.	.	.	.	.	.	.	.
ADAMTS6	.	.	.	.	.	.	.	.	0.4438517	7.46E-05	.	.	.
ADAT2	.	.	.	.	.	.	.	.	0.4738768	2.00E-05	.	.	.
ADAT3	.	.	0.4255588	0.0001569	.	.	.	.	.	.	.	.	.
ADCK1	.	.	.	.	.	.	.	.	.	.	0.626262	2.41E-09	.
ADCY5	.	.	0.5362721	8.46E-07	.	.	.	.	0.4234557	0.0001705	.	.	.
ADCY6	.	.	0.5866557	3.96E-08	.	.	.	.	.	.	.	.	.
ADCYAP1	.	.	.	.	0.4647733	3.02E-05	.	.	.	.	.	.	.
ADD1	.	.	.	.	0.3192112	0.005569	.	.	.	.	.	.	yes
ADD3	.	.	0.4225748	0.0001765	.	.	.	.	.	.	.	.	.
ADH5	.	.	.	.	.	.	.	.	0.3741527	0.0010237	.	.	yes
ADM	.	.	0.4316783	0.000123	.	.	.	.	.	.	.	.	.
ADNP	.	.	0.3584604	0.0017126	.	.	.	.	0.3974849	0.0004536	.	.	yes
ADO	.	.	.	.	.	.	.	.	.	.	0.4853346	1.17E-05	.
ADORA1	.	.	.	.	.	.	.	.	.	.	0.5526026	3.31E-07	.
ADORA2A	.	.	.	.	.	.	.	.	0.693368	7.55E-12	.	.	.
ADPRH	.	.	.	.	.	.	.	.	0.7923381	4.14E-17	.	.	.
ADPRHL1	.	.	.	.	.	.	.	.	.	.	0.8028327	7.81E-18	.
ADRA2C	.	.	.	.	.	.	.	.	0.5841845	4.66E-08	.	.	.
ADRBK2	.	.	0.4930925	8.07E-06	.	.	.	.	.	.	.	.	.
ADRM1	.	.	.	.	.	.	0.6490773	3.98E-10	.	.	.	.	.
ADSS	.	.	0.3332788	0.0037125	.	.	.	.	0.4602187	3.70E-05	.	.	.
AES	.	.	.	.	.	.	.	.	0.6081333	9.10E-09	.	.	.
AFAP1L1	.	.	0.3512183	0.0021532	.	.	0.3843082	0.0007236	0.6165272	4.97E-09	.	.	.
AFF4	.	.	.	.	.	.	.	.	.	.	.	.	yes
AFM	.	.	.	.	.	.	.	.	.	.	0.4739096	2.00E-05	.
AGAP2	.	.	.	.	.	.	.	.	.	.	0.6700949	6.60E-11	.
AGBL1	.	.	0.4342765	0.0001107	.	.	.	.	.	.	.	.	.
AGFG1	.	.	0.5506693	3.71E-07	.	.	.	.	.	.	.	.	.
AGMO	.	.	0.3756498	0.0009733	.	.	.	.	.	.	.	.	.
AGO1	.	.	.	.	.	.	.	.	.	.	0.5961385	2.10E-08	.
AGPAT1	.	.	.	.	.	.	.	.	0.6016443	1.43E-08	.	.	.
AGPAT3	.	.	0.4373013	9.79E-05	.	.	.	.	.	.	.	.	.

Gene	Atrial Expression	p-value Atrial	AV-Canal Expression	p-value AV-Canal	Branchial Arches Expression	p-value Branchial Arches	Heart Tube Expression	p-value Heart Tube	Outflow Tract Expression	p-value Outflow Tract	Ventricle Expression	p-value Ventricle	DMP Expression
AGRN	.	.	.	.	.	.	.	.	.	.	.	.	yes
AHCTF1	.	.	.	.	.	.	.	.	.	.	0.6843733	1.79E-11	yes
AHCYL1	.	.	.	.	.	.	.	.	.	.	.	.	yes
AHCYL2	.	.	.	.	.	.	.	.	0.5040844	4.68E-06	.	.	.
AHSA1	.	.	.	.	.	.	.	.	.	.	.	.	yes
AIDA	.	.	0.4530962	5.04E-05	.	.	.	.	0.4949593	7.36E-06	.	.	.
AIF1L	0.3117667	0.0068508	.	.	0.505474	4.36E-06	.	.	0.4553152	4.58E-05	.	.	.
AIFM1	.	.	0.6313241	1.64E-09	.	.	.	.	.	.	.	.	.
AIPL1	.	.	0.4036106	0.0003627	.	.	.	.	.	.	.	.	.
AJUBA	.	.	0.4581842	4.04E-05	.	.	.	.	.	.	.	.	.
AK1	.	.	.	.	.	.	0.4969367	6.68E-06	.	.	.	.	.
AK2	.	.	0.6530644	2.86E-10	.	.	.	.	.	.	.	.	.
AK8	.	.	.	.	.	.	.	.	.	.	0.6453356	5.40E-10	.
AKAP10	.	.	.	.	0.5287669	1.28E-06	.	.	.	.	.	.	.
AKAP11	.	.	0.4481631	6.22E-05	.	.	.	.	.	.	.	.	.
AKAP12	.	.	.	.	.	.	.	.	0.4808723	1.45E-05	.	.	.
AKAP13	.	.	0.3923311	0.0005458	.	.	.	.	.	.	.	.	.
AKAP6	.	.	.	.	.	.	.	.	.	.	0.7167847	6.87E-13	.
AKAP8	.	.	.	.	.	.	.	.	.	.	.	.	yes
AKIP1	.	.	.	.	.	.	.	.	0.6856495	1.58E-11	.	.	.
AKIRIN2	.	.	0.3917777	0.0005566	.	.	.	.	0.6654619	9.93E-11	.	.	.
AKNA	.	.	.	.	.	.	.	.	0.5296528	1.22E-06	.	.	.
AKR1A1	.	.	0.3690443	0.0012138	.	.	.	.	.	.	.	.	yes
AKR1B1	.	.	0.4186898	0.0002052	.	.	.	.	.	.	.	.	.
AKR1E2	.	.	.	.	.	.	.	.	0.474366	1.96E-05	.	.	.
AKT1	.	.	.	.	.	.	.	.	.	.	.	.	yes
AKT2	.	.	.	.	.	.	.	.	0.5873964	3.77E-08	.	.	.
ALAS1	.	.	0.4318612	0.000122	.	.	.	.	0.4243289	0.0001647	.	.	.
ALDH3A1	0.4379986	9.51E-05	.	.	.	.	.	.	.	.	.	.	.
ALDH3B1	.	.	.	.	.	.	.	.	.	.	0.702061	3.19E-12	.
ALDOA	.	.	.	.	.	.	0.53605	8.56E-07	.	.	.	.	yes
ALG1	.	.	.	.	.	.	.	.	0.4417274	8.15E-05	.	.	.
ALG3	.	.	.	.	.	.	.	.	0.4609421	3.58E-05	.	.	.
ALG8	.	.	.	.	.	.	.	.	0.6066521	1.01E-08	.	.	.
ALG9	.	.	.	.	.	.	.	.	0.5158245	2.56E-06	.	.	.
ALKBH5	.	.	.	.	.	.	.	.	0.3786347	0.0008796	.	.	yes
ALOX15	.	.	.	.	.	.	.	.	.	.	0.6334477	1.39E-09	.
ALOX15B	.	.	0.4861204	1.13E-05	.	.	.	.	.	.	.	.	.
ALPK2	.	.	0.3433349	0.0027463	.	.	0.4577727	4.12E-05	0.4680237	2.61E-05	.	.	.
ALPK3	.	.	0.5161279	2.52E-06	.	.	.	.	0.6739814	4.66E-11	.	.	.
ALPL	.	.	.	.	.	.	.	.	0.5111294	3.26E-06	.	.	.
ALPPL2	.	.	0.3961833	0.0004754	.	.	.	.	.	.	.	.	.
ALS2CL	.	.	.	.	0.5566118	2.61E-07	.	.	.	.	.	.	.
AMBRA1	.	.	.	.	.	.	.	.	0.7006998	3.65E-12	.	.	.
AMD1	.	.	.	.	.	.	.	.	0.4734432	2.04E-05	.	.	.
AMFR	.	.	0.4374179	9.74E-05	.	.	.	.	.	.	.	.	.
AMMECR1	.	.	.	.	.	.	.	.	0.5388774	7.31E-07	.	.	.
AMN1	.	.	.	.	.	.	.	.	0.5442953	5.37E-07	.	.	.
AMOTL1	.	.	.	.	.	.	.	.	0.3108507	0.0070252	0.4143837	0.0002421	yes
AMOTL2	.	.	0.3774107	0.000917	.	.	.	.	.	.	.	.	.
AMPH	.	.	.	.	.	.	.	.	0.512302	3.07E-06	.	.	.
ANAPC1	.	.	.	.	0.3346291	0.0035672	.	.	0.3368123	0.0033431	.	.	yes
ANAPC11	.	.	.	.	.	.	.	.	0.6777784	3.30E-11	.	.	.
ANAPC2	.	.	0.3753069	0.0009847	.	.	.	.	0.372409	0.0010853	.	.	yes
ANAPC5	.	.	.	.	0.3392456	0.0031081	.	.	.	.	.	.	yes
ANAPC7	.	.	.	.	.	.	.	.	0.4947092	7.45E-06	.	.	.
ANG	0.5536779	3.11E-07	.	.	.	.	.	.	.	.	.	.	.
ANGPT2	.	.	.	.	.	.	.	.	0.4713568	2.25E-05	.	.	.
ANK1	.	.	0.407824	0.0003102	.	.	.	.	.	.	.	.	.
ANK3	.	.	.	.	.	.	.	.	0.4572974	4.20E-05	.	.	.
ANKFY1	.	.	0.4485668	6.12E-05	.	.	.	.	.	.	.	.	.
ANKRD1	.	.	0.5776429	7.11E-08	.	.	.	.	0.5971502	1.96E-08	.	.	.
ANKRD11	.	.	.	.	.	.	.	.	.	.	.	.	yes
ANKRD13A	.	.	0.4998102	5.79E-06	.	.	.	.	0.4754667	1.86E-05	.	.	.
ANKRD17	.	.	0.402667	0.0003755	.	.	.	.	0.3387186	0.0031577	.	.	yes
ANKRD34A	.	.	.	.	.	.	.	.	.	.	0.8558648	2.63E-22	.
ANKRD35	.	.	.	.	.	.	.	.	.	.	0.6877953	1.29E-11	.
ANKRD52	.	.	.	.	.	.	.	.	0.6138826	6.02E-09	.	.	.
ANKRD6	.	.	.	.	.	.	.	.	.	.	0.6822559	2.18E-11	.
ANP32A	.	.	.	.	.	.	.	.	.	.	.	.	yes
ANP32B	.	.	0.3590722	0.0016794	.	.	.	.	.	.	.	.	yes
ANP32E	.	.	0.3967433	0.0004659	.	.	.	.	.	.	.	.	yes
ANTXR2	.	.	0.3429765	0.0027764	.	.	.	.	0.6672766	8.47E-11	.	.	.
ANXA5	.	.	0.4016083	0.0003904	.	.	.	.	0.5257614	1.51E-06	.	.	.
ANXA6	.	.	0.4095292	0.000291	.	.	.	.	.	.	.	.	.

Gene	Atrial Expression	p-value Atrial	AV-Canal Expression	p-value AV-Canal	Branchial Arches Expression	p-value Branchial Arches	Heart Tube Expression	p-value Heart Tube	Outflow Tract Expression	p-value Outflow Tract	Ventricle Expression	p-value Ventricle	DMP Expression
ANXA8	.	.	.	.	0.5198948	2.06E-06	.	.	.	.	.	.	.
AOX1	.	.	.	.	.	.	.	.	0.5509519	3.65E-07	.	.	.
AP1G1	.	.	.	.	.	.	.	.	.	.	.	.	yes
AP1S2	.	.	.	.	.	.	.	.	0.6304566	1.75E-09	.	.	.
AP2A1	.	.	.	.	.	.	.	.	0.5755808	8.11E-08	.	.	.
AP2A2	.	.	.	.	.	.	.	.	0.5231232	1.74E-06	.	.	yes
AP2B1	.	.	0.3856024	0.0006917	.	.	.	.	0.3627604	0.0014911	.	.	yes
AP2M1	.	.	0.3058334	0.0080514	.	.	.	.	0.5540625	3.04E-07	.	.	.
AP3S2	.	.	.	.	.	.	.	.	0.5971295	1.96E-08	.	.	.
AP4B1	.	.	.	.	.	.	.	.	0.6818703	2.26E-11	.	.	.
AP4M1	.	.	.	.	.	.	.	.	0.4498771	5.79E-05	.	.	.
AP5M1	.	.	0.4228619	0.0001745	.	.	.	.	.	.	.	.	.
APBB3	.	.	.	.	.	.	.	.	0.8176531	6.18E-19	.	.	.
API5	.	.	0.304364	0.0083758	.	.	.	.	.	.	.	.	yes
APLP2	.	.	.	.	.	.	.	.	.	.	.	.	yes
APOBEC2	.	.	0.371223	0.0011291	.	.	.	.	0.6059198	1.06E-08	.	.	.
APOL2	.	.	.	.	.	.	.	.	.	.	0.7004016	3.77E-12	.
APP	.	.	0.4656247	2.91E-05	.	.	.	.	.	.	.	.	yes
APPBP2	.	.	0.3832262	0.0007512	.	.	.	.	0.3034976	0.0085724	.	.	.
AQPEP	.	.	.	.	.	.	.	.	.	.	0.6233326	3.00E-09	.
ARCN1	.	.	0.4239242	0.0001674	.	.	.	.	.	.	.	.	yes
ARF3	.	.	0.4011574	0.0003969	.	.	.	.	0.4474078	6.43E-05	.	.	yes
ARF4	.	.	0.3829508	0.0007584	.	.	.	.	0.3229998	0.005002	.	.	yes
ARFGEF1	.	.	.	.	.	.	.	.	0.4999935	5.74E-06	.	.	.
ARFGEF2	.	.	.	.	.	.	.	.	0.5968985	1.99E-08	.	.	.
ARFIP2	.	.	.	.	0.4716403	2.22E-05	.	.	.	.	.	.	.
ARG1	.	.	.	.	.	.	.	.	.	.	.	.	yes
ARHGAP1	.	.	0.5885073	3.50E-08	.	.	.	.	.	.	.	.	.
ARHGAP28	.	.	.	.	0.4803116	1.49E-05	.	.	.	.	.	.	yes
ARHGAP29	.	.	0.5167883	2.43E-06	.	.	.	.	.	.	.	.	.
ARHGAP31	.	.	0.5646834	1.60E-07	.	.	.	.	0.4595253	3.81E-05	.	.	.
ARHGAP35	.	.	.	.	.	.	.	.	.	.	.	.	yes
ARHGAP40	.	.	.	.	.	.	.	.	.	.	0.5515258	3.53E-07	.
ARHGAP44	.	.	.	.	.	.	.	.	.	.	0.5272564	1.39E-06	.
ARHGDIA	.	.	.	.	.	.	.	.	0.6423991	6.85E-10	.	.	yes
ARHGEF10L	0.4582224	4.04E-05	.	.	.	.	.	.	.	.	.	.	.
ARHGEF12	.	.	0.4009294	0.0004002	.	.	.	.	0.4631047	3.26E-05	.	.	.
ARHGEF17	.	.	.	.	.	.	.	.	0.4504889	5.64E-05	.	.	yes
ARHGEF2	.	.	.	.	.	.	.	.	0.4410577	8.38E-05	.	.	.
ARID1A	.	.	0.2987028	0.0097351	.	.	.	.	0.3491214	0.0022986	.	.	yes
ARID2	.	.	.	.	.	.	.	.	.	.	.	.	yes
ARID3A	.	.	.	.	.	.	.	.	0.532725	1.03E-06	.	.	.
ARIH1	.	.	0.3230629	0.004993	.	.	.	.	.	.	.	.	yes
ARL4D	.	.	0.484132	1.24E-05	.	.	.	.	.	.	.	.	.
ARL5B	.	.	.	.	.	.	.	.	0.5266442	1.44E-06	.	.	.
ARL5C	.	.	0.3799275	0.0008416	.	.	.	.	.	.	.	.	.
ARL6IP1	.	.	.	.	.	.	.	.	.	.	.	.	yes
ARL6IP5	.	.	0.4033799	0.0003658	.	.	.	.	0.3608965	0.0015838	.	.	.
ARL6IP6	.	.	.	.	.	.	.	.	0.5084453	3.75E-06	.	.	.
ARL8B	.	.	.	.	.	.	.	.	0.641925	7.11E-10	.	.	.
ARMC10	.	.	.	.	.	.	.	.	0.6394362	8.67E-10	.	.	.
ARMC5	.	.	.	.	.	.	.	.	0.6922022	8.46E-12	.	.	.
ARMCX2	.	.	0.3798428	0.000844	.	.	.	.	.	.	.	.	yes
ARMCX3	.	.	0.3886265	0.0006222	.	.	.	.	0.6511819	3.35E-10	.	.	.
ARPC1A	.	.	.	.	.	.	.	.	0.4516112	5.37E-05	.	.	.
ARPC2	.	.	0.5308613	1.14E-06	.	.	.	.	0.4527694	5.11E-05	.	.	yes
ARPC3	.	.	.	.	.	.	.	.	0.6178504	4.51E-09	.	.	.
ARPC4	.	.	.	.	.	.	.	.	0.6258804	2.48E-09	.	.	.
ARPC5L	.	.	.	.	.	.	.	.	0.5748337	8.50E-08	.	.	.
ARPP19	.	.	.	.	.	.	.	.	.	.	.	.	yes
ARSA	.	.	0.4127017	0.0002581	.	.	.	.	.	.	.	.	.
ARSG	.	.	.	.	.	.	.	.	0.4569327	4.27E-05	.	.	.
ART3	.	.	.	.	.	.	.	.	0.6418797	7.14E-10	.	.	.
ARTN	.	.	.	.	.	.	.	.	.	.	0.6669659	8.70E-11	.
ASAP1	.	.	0.435106	0.000107	.	.	.	.	.	.	.	.	.
ASAP2	.	.	.	.	0.4528522	5.10E-05	.	.	.	.	.	.	.
ASB2	.	.	.	.	.	.	.	.	0.6504802	3.55E-10	.	.	.
ASB4	.	.	.	.	.	.	.	.	.	.	.	.	yes
ASB6	.	.	.	.	.	.	0.3010636	0.0091465	0.5083101	3.77E-06	.	.	.
ASB8	.	.	.	.	.	.	.	.	0.5305908	1.16E-06	.	.	.
ASL	.	.	.	.	.	.	.	.	.	.	0.4990471	6.02E-06	.
ASNA1	.	.	.	.	.	.	0.3605635	0.0016008	0.5398979	6.90E-07	.	.	.
ASPH	.	.	.	.	.	.	.	.	0.8351468	2.27E-20	.	.	.
ASPHD2	.	.	.	.	0.4616699	3.47E-05	.	.	.	.	.	.	.
ASXL2	.	.	.	.	.	.	0.5015044	5.32E-06	.	.	.	.	.

Gene	Atrial Expression	p-value Atrial	AV-Canal Expression	p-value AV-Canal	Branchial Arches Expression	p-value Branchial Arches	Heart Tube Expression	p-value Heart Tube	Outflow Tract Expression	p-value Outflow Tract	Ventricle Expression	p-value Ventricle	DMP Expression
ASXL3	.	.	.	.	0.5378779	7.73E-07	.	.	0.4136397	0.0002491	.	.	.
ATAD2	.	.	0.5289594	1.27E-06	.	.	.	.	.	.	.	.	yes
ATF3	.	.	0.5422109	6.05E-07	.	.	.	.	0.3547835	0.001925	.	.	.
ATF4	.	.	.	.	.	.	.	.	0.4569248	4.27E-05	.	.	yes
ATG12	.	.	0.4740951	1.98E-05	.	.	.	.	.	.	.	.	.
ATG2B	.	.	.	.	.	.	.	.	.	.	0.4795219	1.54E-05	.
ATG4C	.	.	.	.	.	.	.	.	.	.	0.4818473	1.38E-05	.
ATG5	.	.	0.4127012	0.0002581	.	.	.	.	.	.	.	.	.
ATG9B	.	.	.	.	.	.	.	.	0.4613124	3.52E-05	.	.	.
ATIC	.	.	.	.	.	.	.	.	.	.	.	.	yes
ATL2	.	.	0.5499959	3.86E-07	.	.	.	.	.	.	.	.	.
ATL3	.	.	0.3965937	0.0004685	.	.	.	.	.	.	.	.	.
ATOH7	.	.	.	.	.	.	.	.	.	.	0.5744769	8.70E-08	.
ATOH8	.	.	0.4249975	0.0001604	.	.	.	.	.	.	.	.	yes
ATP13A2	.	.	.	.	.	.	.	.	.	.	0.7007493	3.64E-12	.
ATP13A3	.	.	.	.	.	.	.	.	0.800304	1.18E-17	.	.	.
ATP13A5	.	.	.	.	.	.	.	.	.	.	0.5837044	4.80E-08	.
ATP1A1	.	.	0.4364374	0.0001014	.	.	.	.	0.5746478	8.60E-08	.	.	yes
ATP1A2	.	.	0.4279247	0.0001429	.	.	.	.	0.3021345	0.0088899	.	.	.
ATP1B1	.	.	0.3713467	0.0011245	.	.	.	.	0.6633695	1.19E-10	.	.	.
ATP1B2	.	.	0.4286496	0.0001388	.	.	.	.	.	.	.	.	.
ATP1B3	.	.	.	.	.	.	.	.	0.537571	7.86E-07	.	.	.
ATP2A2	.	.	.	.	.	.	.	.	.	.	.	.	yes
ATP2B1	.	.	0.4906477	9.08E-06	.	.	.	.	.	.	.	.	yes
ATP2B4	.	.	.	.	.	.	.	.	0.5372338	8.01E-07	.	.	.
ATP5A1	.	.	.	.	.	.	.	.	0.6279528	2.12E-09	.	.	yes
ATP5B	.	.	0.4195577	0.0001985	.	.	.	.	0.5339087	9.65E-07	.	.	yes
ATP5C1	.	.	0.4205723	0.0001908	.	.	.	.	.	.	.	.	yes
ATP5D	.	.	.	.	.	.	.	.	0.6360483	1.13E-09	.	.	yes
ATP5F1	.	.	.	.	.	.	.	.	0.5942438	2.38E-08	.	.	.
ATP5G1	.	.	.	.	.	.	.	.	0.5019565	5.20E-06	.	.	.
ATP5G2	.	.	0.5894346	3.29E-08	.	.	.	.	.	.	.	.	.
ATP5G3	.	.	.	.	.	.	.	.	.	.	.	.	yes
ATP5I	.	.	0.3434076	0.0027402	.	.	.	.	0.7675229	1.50E-15	.	.	.
ATP5J2	.	.	0.3026084	0.0087784	.	.	.	.	0.7738516	6.29E-16	.	.	.
ATP5O	.	.	0.5552019	2.84E-07	.	.	.	.	0.5278037	1.35E-06	.	.	.
ATP6V0A1	.	.	.	.	.	.	.	.	0.6607307	1.50E-10	.	.	.
ATP6V0A2	.	.	.	.	.	.	.	.	0.4524156	5.19E-05	.	.	.
ATP6V0D1	.	.	0.3363831	0.0033861	.	.	.	.	0.6110936	7.36E-09	.	.	.
ATP6V1A	.	.	.	.	.	.	.	.	0.7125065	1.08E-12	.	.	.
ATP6V1C1	.	.	.	.	.	.	.	.	0.5811745	5.66E-08	.	.	.
ATP8B2	.	.	0.4939047	7.75E-06	.	.	.	.	.	.	.	.	.
ATPAF1	.	.	0.4062817	0.0003285	.	.	.	.	.	.	.	.	.
ATRX	.	.	.	.	.	.	.	.	.	.	.	.	yes
ATXN10	.	.	0.3639256	0.0014358	.	.	.	.	0.3798974	0.0008424	.	.	yes
ATXN2	.	.	.	.	.	.	.	.	0.4486727	6.09E-05	.	.	.
ATXN2L	.	.	.	.	.	.	.	.	0.4186708	0.0002054	.	.	yes
ATXN7L3B	.	.	0.3859216	0.0006841	.	.	.	.	0.4695242	2.44E-05	.	.	yes
AUP1	.	.	.	.	.	.	.	.	0.4498883	5.78E-05	.	.	.
AXL	.	.	.	.	.	.	.	.	0.5241439	1.64E-06	.	.	.
AZIN1	.	.	.	.	.	.	.	.	0.3030081	0.0086852	.	.	yes
B3GALNT1	.	.	.	.	.	.	.	.	0.6754402	4.08E-11	.	.	.
B3GALTL	.	.	0.5868361	3.91E-08	.	.	.	.	.	.	.	.	.
B3GAT3	.	.	0.3814437	0.0007989	.	.	.	.	0.3976183	0.0004514	.	.	.
B3GNT1	.	.	.	.	.	.	.	.	0.5275055	1.37E-06	.	.	.
B3GNT5	.	.	.	.	0.4718605	2.20E-05	.	.	.	.	.	.	.
B3GNT7	.	.	0.3823323	0.0007748	.	.	.	.	.	.	.	.	.
B4GALNT4	.	.	.	.	.	.	.	.	.	.	0.5837771	4.78E-08	.
B4GALT5	.	.	.	.	.	.	.	.	.	.	0.6950383	6.41E-12	.
B4GALT6	.	.	0.3146955	0.0063185	.	.	.	.	0.7480087	1.88E-14	.	.	.
BAALC	.	.	.	.	.	.	.	.	0.6023377	1.37E-08	.	.	.
BAG2	.	.	.	.	.	.	.	.	0.5167275	2.44E-06	.	.	.
BAG4	.	.	.	.	.	.	.	.	0.4957536	7.08E-06	.	.	.
BAG6	.	.	0.458651	3.96E-05	.	.	.	.	.	.	.	.	yes
BAMBI	.	.	0.6788741	2.98E-11	.	.	.	.	.	.	.	.	.
BANF1	.	.	.	.	.	.	.	.	0.7193964	5.18E-13	.	.	.
BANK1	.	.	.	.	.	.	.	.	.	.	0.4870471	1.08E-05	.
BANP	.	.	0.3604467	0.0016069	.	.	.	.	0.576157	7.82E-08	.	.	.
BARX1	.	.	.	.	0.5374472	7.92E-07	.	.	.	.	.	.	.
BASP1	.	.	.	.	0.4331989	0.0001156	.	.	.	.	.	.	yes
BAZ1B	.	.	.	.	.	.	.	.	.	.	.	.	yes
BCAM	.	.	0.353324	0.0020157	.	.	.	.	0.5160188	2.53E-06	.	.	.
BCAR1	.	.	.	.	.	.	.	.	0.856121	2.48E-22	.	.	.
BCAT1	.	.	.	.	0.4452608	7.03E-05	.	.	.	.	.	.	.
BCHE	.	.	0.4427663	7.81E-05	.	.	.	.	.	.	.	.	.

Gene	Atrial Expression	p-value Atrial	AV-Canal Expression	p-value AV-Canal	Branchial Arches Expression	p-value Branchial Arches	Heart Tube Expression	p-value Heart Tube	Outflow Tract Expression	p-value Outflow Tract	Ventricle Expression	p-value Ventricle	DMP Expression
BCKDK	.	.	0.3146307	0.0063299	.	.	.	.	0.4647273	3.03E-05	.	.	.
BCL10	.	.	0.4158404	0.000229	.	.	.	.	0.5684243	1.27E-07	.	.	.
BCL7A	.	.	.	.	.	.	.	.	0.4943917	7.57E-06	.	.	.
BCL9	.	.	.	.	.	.	.	.	0.3529551	0.0020392	.	.	yes
BCLAF1	.	.	.	.	.	.	.	.	.	.	.	.	yes
BCOR	.	.	.	.	.	.	0.2981216	0.0098849	0.4880233	1.03E-05	.	.	.
BCORL1	.	.	0.5839965	4.71E-08	.	.	.	.	.	.	.	.	.
BCR	.	.	.	.	.	.	.	.	0.5911152	2.94E-08	.	.	.
BECN1	.	.	.	.	.	.	.	.	0.4367819	1.00E-04	.	.	.
BEND4	.	.	0.3631052	0.0014746	.	.	.	.	0.7263324	2.41E-13	.	.	.
BFSP1	.	.	0.3823648	0.0007739	.	.	.	.	.	.	.	.	.
BHLHB9	.	.	.	.	.	.	.	.	0.5577449	2.44E-07	.	.	.
BICD2	.	.	.	.	.	.	.	.	0.5549787	2.88E-07	.	.	.
BID	.	.	.	.	.	.	.	.	0.322749	0.0050379	0.4991653	5.98E-06	.
BIK	.	.	.	.	.	.	.	.	0.6646876	1.06E-10	.	.	.
BIRC6	.	.	.	.	.	.	.	.	.	.	.	.	yes
BLMH	.	.	.	.	.	.	.	.	0.3858381	0.0006861	.	.	yes
BLOC1S1	.	.	0.4369775	9.92E-05	.	.	.	.	.	.	.	.	.
BLOC1S4	.	.	.	.	.	.	.	.	0.6591108	1.72E-10	.	.	.
BLOC1S6	.	.	.	.	.	.	.	.	0.4560526	4.44E-05	.	.	.
BMP4	.	.	.	.	.	.	0.3480129	0.0023789	0.4852898	1.17E-05	.	.	.
BMP7	.	.	0.4731951	2.07E-05	.	.	.	.	0.3868376	0.0006625	.	.	.
BMPER	.	.	0.6101742	7.86E-09	.	.	.	.	.	.	.	.	.
BMPR1A	.	.	0.3847677	0.0007121	.	.	.	.	.	.	.	.	yes
BMS1	.	.	.	.	.	.	.	.	.	.	.	.	yes
BNIP2	.	.	.	.	0.5357477	8.71E-07	.	.	.	.	.	.	.
BNIP3	.	.	0.5653639	1.54E-07	.	.	.	.	.	.	.	.	.
BNIP3L	.	.	.	.	.	.	.	.	0.7023971	3.08E-12	.	.	.
BOD1	.	.	.	.	.	.	.	.	0.4789826	1.58E-05	.	.	.
BPIFB2	.	.	.	.	0.4377461	9.61E-05	.	.	.	.	.	.	.
BPTF	.	.	.	.	.	.	.	.	.	.	.	.	yes
BRAF	.	.	0.3825238	0.0007697	.	.	.	.	.	.	.	.	.
BRCC3	.	.	.	.	.	.	.	.	0.5682106	1.29E-07	.	.	.
BRD2	.	.	.	.	.	.	.	.	.	.	.	.	yes
BRD3	.	.	.	.	.	.	.	.	.	.	.	.	yes
BRD4	.	.	0.4003201	0.0004092	.	.	.	.	.	.	.	.	yes
BRD9	.	.	0.5018982	5.22E-06	.	.	.	.	.	.	.	.	.
BRF2	.	.	0.3251922	0.0046978	.	.	.	.	0.4686908	2.54E-05	.	.	.
BRI3	.	.	.	.	.	.	.	.	0.4466553	6.63E-05	.	.	.
BRI3BP	.	.	0.4425761	7.87E-05	.	.	.	.	0.4183911	0.0002076	.	.	.
BRSK1	.	.	.	.	.	.	.	.	0.6664703	9.09E-11	.	.	.
BRWD3	.	.	.	.	.	.	.	.	.	.	0.4599469	3.74E-05	.
BSG	.	.	.	.	.	.	.	.	0.3432006	0.0027575	.	.	yes
BTAF1	.	.	.	.	.	.	.	.	0.4567686	4.30E-05	.	.	.
BTBD1	.	.	0.3862718	0.0006758	.	.	.	.	0.3070284	0.0077958	.	.	.
BTBD6	.	.	.	.	.	.	.	.	.	.	0.4599604	3.74E-05	.
BTG4	.	.	.	.	.	.	.	.	.	.	0.6749837	4.25E-11	.
BTK	.	.	.	.	.	.	.	.	0.5921671	2.74E-08	.	.	.
BTN1A1	.	.	0.4093999	0.0002924	.	.	.	.	.	.	.	.	.
BTNL10	.	.	.	.	.	.	.	.	0.4379381	9.53E-05	.	.	.
BTRC	.	.	0.4363312	0.0001018	.	.	.	.	.	.	.	.	.
BUB1	.	.	0.3725024	0.0010819	.	.	.	.	.	.	.	.	yes
BUB1B	.	.	0.3070581	0.0077895	0.3226516	0.0050519	.	.	.	.	.	.	yes
BUB3	.	.	.	.	.	.	.	.	0.4438373	7.47E-05	.	.	yes
BVES	.	.	0.5879964	3.62E-08	.	.	.	.	.	.	.	.	.
BZW1	.	.	.	.	.	.	.	.	.	.	.	.	yes
BZW2	.	.	.	.	.	.	.	.	.	.	.	.	yes
C10orf118	.	.	0.457534	4.16E-05	.	.	.	.	.	.	.	.	.
C11orf58	.	.	.	.	.	.	.	.	0.536534	8.33E-07	.	.	.
C11orf86	.	.	.	.	0.5009653	5.47E-06	.	.	.	.	.	.	.
C12orf4	.	.	.	.	.	.	.	.	0.5224048	1.81E-06	.	.	.
C12orf5	.	.	.	.	.	.	.	.	0.764819	2.17E-15	.	.	.
C12orf65	.	.	.	.	.	.	.	.	0.6142503	5.86E-09	.	.	.
C12orf68	.	.	.	.	.	.	.	.	.	.	0.6906305	9.85E-12	.
C14orf119	.	.	.	.	.	.	.	.	0.4860629	1.13E-05	.	.	.
C14orf142	.	.	.	.	0.5399547	6.87E-07	.	.	.	.	.	.	.
C14orf159	.	.	.	.	.	.	.	.	.	.	0.6365091	1.09E-09	.
C14orf169	.	.	0.3939847	0.0005145	.	.	.	.	0.3446477	0.0026384	.	.	.
C14orf180	.	.	.	.	.	.	.	.	.	.	0.6127261	6.55E-09	.
C14orf2	.	.	0.4267039	0.00015	.	.	.	.	.	.	.	.	.
C14orf37	.	.	.	.	.	.	0.4551483	4.61E-05	0.7962125	2.26E-17	.	.	.
C15orf38-AP3S2	.	.	.	.	.	.	.	.	0.5399439	6.88E-07	.	.	.
C16orf52	.	.	.	.	.	.	.	.	0.6631091	1.22E-10	.	.	.
C16orf59	.	.	.	.	0.5715739	1.04E-07	.	.	.	.	.	.	.
C16orf72	.	.	.	.	.	.	.	.	0.4396207	8.90E-05	.	.	.

Gene	Atrial Expression	p-value Atrial	AV-Canal Expression	p-value AV-Canal	Branchial Arches Expression	p-value Branchial Arches	Heart Tube Expression	p-value Heart Tube	Outflow Tract Expression	p-value Outflow Tract	Ventricle Expression	p-value Ventricle	DMP Expression
C16orf74	.	.	.	.	.	.	.	.	0.6731376	5.02E-11	.	.	.
C16orf80	.	.	0.3484269	0.0023486	.	.	.	.	0.4858084	1.15E-05	.	.	.
C16orf91	.	.	0.3833144	0.000749	.	.	.	.	.	.	.	.	.
C17orf49	.	.	0.4207129	0.0001897	.	.	.	.	.	.	.	.	.
C17orf59	.	.	0.3383798	0.00319	.	.	.	.	0.567434	1.35E-07	.	.	.
C17orf96	.	.	.	.	0.6747911	4.33E-11	.	.	0.3069132	0.0078201	.	.	.
C19orf66	.	.	.	.	0.4474298	6.42E-05	.	.	.	.	.	.	.
C19orf68	.	.	.	.	.	.	.	.	0.5610289	2.00E-07	.	.	.
C1GALT1	.	.	.	.	.	.	.	.	0.6543551	2.57E-10	.	.	.
C1orf106	.	.	.	.	.	.	.	.	0.605643	1.08E-08	.	.	.
C1orf109	.	.	.	.	.	.	.	.	0.4961923	6.93E-06	.	.	.
C1orf111	0.5863359	4.04E-08	.	.	.	.	.	.	.	.	.	.	.
C1orf174	.	.	.	.	.	.	.	.	0.4901904	9.29E-06	.	.	.
C1orf198	.	.	0.4153687	0.0002332	.	.	.	.	0.4407132	8.50E-05	.	.	.
C1orf21	.	.	.	.	.	.	.	.	0.4822302	1.36E-05	.	.	.
C1orf227	.	.	0.3970575	0.0004607	.	.	.	.	.	.	.	.	.
C1orf43	.	.	.	.	.	.	.	.	0.6504828	3.55E-10	.	.	.
C1orf86	.	.	.	.	0.5359466	8.61E-07	.	.	.	.	.	.	.
C1QBP	.	.	.	.	.	.	0.4313607	0.0001245	.	.	.	.	yes
C20orf194	.	.	0.3945207	0.0005047	.	.	.	.	.	.	.	.	.
C21orf59	.	.	0.3031871	0.0086438	.	.	.	.	0.5853455	4.31E-08	.	.	.
C2CD5	.	.	.	.	.	.	.	.	.	.	0.7000136	3.91E-12	.
C2orf43	.	.	.	.	.	.	.	.	0.6320838	1.54E-09	.	.	.
C3orf80	.	.	0.4799585	1.51E-05	.	.	.	.	.	.	.	.	.
C4orf17	.	.	.	.	.	.	.	.	.	.	0.4628865	3.29E-05	.
C4orf46	.	.	.	.	.	.	.	.	0.5836283	4.83E-08	.	.	.
C5AR2	.	.	.	.	.	.	.	.	0.7111131	1.25E-12	.	.	.
C5orf15	.	.	0.4688893	2.51E-05	.	.	.	.	0.6044517	1.18E-08	.	.	yes
C5orf24	.	.	0.38267	0.0007658	.	.	.	.	.	.	.	.	yes
C5orf42	.	.	.	.	.	.	.	.	.	.	0.4560374	4.44E-05	.
C6orf106	.	.	.	.	.	.	.	.	0.5551622	2.85E-07	.	.	.
C6orf211	.	.	.	.	.	.	.	.	0.6070675	9.81E-09	.	.	.
C6orf222	.	.	0.4045984	0.0003497	.	.	.	.	.	.	.	.	.
C6orf47	.	.	0.3902335	0.0005879	.	.	.	.	.	.	.	.	.
C6orf62	.	.	0.4448905	7.14E-05	.	.	.	.	.	.	.	.	.
C7orf26	.	.	.	.	0.4544922	4.75E-05	.	.	.	.	.	.	.
C7orf50	.	.	.	.	0.4691904	2.48E-05	.	.	.	.	.	.	.
C7orf55	.	.	0.4379611	9.52E-05	.	.	.	.	0.7329449	1.14E-13	.	.	.
C8G	.	.	.	.	.	.	.	.	0.438069	9.48E-05	.	.	.
C8orf4	.	.	.	.	.	.	.	.	0.6714504	5.85E-11	.	.	.
C9orf116	0.5108247	3.32E-06	.	.	.	.	.	.	.	.	.	.	.
C9orf41	.	.	.	.	.	.	.	.	0.7174707	6.38E-13	.	.	.
C9orf72	.	.	.	.	.	.	.	.	0.4869713	1.08E-05	.	.	.
CA4	.	.	.	.	.	.	0.5349759	9.09E-07	0.3326	0.0037875	.	.	.
CAB39	.	.	0.3849788	0.0007069	.	.	.	.	.	.	.	.	.
CAB39L	.	.	0.4172228	0.0002172	.	.	.	.	.	.	.	.	.
CABP5	.	.	.	.	.	.	.	.	.	.	0.5658587	1.49E-07	.
CACNA1E	.	.	.	.	.	.	.	.	.	.	0.5455426	5.00E-07	.
CACNA1S	.	.	.	.	.	.	.	.	0.4898594	9.44E-06	.	.	.
CACNA2D2	.	.	0.4718868	2.19E-05	.	.	.	.	.	.	.	.	.
CACNB3	.	.	0.4358726	0.0001037	.	.	.	.	.	.	.	.	.
CACNG7	.	.	.	.	.	.	.	.	0.5103633	3.40E-06	.	.	.
CACTIN	.	.	0.3962238	0.0004748	.	.	.	.	0.3527454	0.0020527	.	.	.
CAD	.	.	.	.	.	.	.	.	.	.	.	.	yes
CADPS	.	.	.	.	.	.	.	.	.	.	0.6940904	7.04E-12	.
CADPS2	.	.	.	.	.	.	.	.	.	.	0.4518119	5.33E-05	.
CALCA	0.7473192	2.05E-14	.	.	.	.	.	.	.	.	.	.	.
CALR	.	.	0.336274	0.0033971	.	.	.	.	0.7865607	9.98E-17	.	.	yes
CALU	.	.	0.3432506	0.0027533	.	.	.	.	.	.	.	.	yes
CAMK2A	.	.	.	.	.	.	.	.	0.7480006	1.88E-14	.	.	.
CAMK2G	.	.	0.3752711	0.0009858	.	.	.	.	.	.	.	.	.
CAMLG	.	.	.	.	.	.	.	.	0.6394252	8.68E-10	.	.	.
CAMSAP3	.	.	.	.	.	.	.	.	.	.	0.5626585	1.81E-07	.
CAMTA2	.	.	.	.	.	.	.	.	0.4493563	5.92E-05	.	.	.
CAND1	.	.	0.4474914	6.40E-05	.	.	.	.	.	.	.	.	yes
CAND2	.	.	.	.	.	.	.	.	.	.	.	.	yes
CANX	.	.	.	.	.	.	.	.	0.5086632	3.71E-06	.	.	yes
CAP1	.	.	.	.	.	.	.	.	.	.	.	.	yes
CAP2	.	.	0.4041537	0.0003555	.	.	.	.	0.5887012	3.46E-08	.	.	.
CAPG	.	.	0.5609485	2.01E-07	.	.	.	.	.	.	.	.	.
CAPN2	.	.	0.3321371	0.0038394	.	.	.	.	0.6519788	3.13E-10	.	.	.
CAPN6	.	.	0.3533622	0.0020132	.	.	0.4230464	0.0001732	.	.	.	.	yes
CAPNS1	.	.	0.3009242	0.0091803	.	.	.	.	0.633679	1.36E-09	.	.	.
CAPRIN1	.	.	.	.	0.5145407	2.73E-06	.	.	0.3677581	0.0012664	.	.	yes
CAPS2	.	.	.	.	.	.	.	.	.	.	0.5758258	7.98E-08	.

Gene	Atrial Expression	p-value Atrial	AV-Canal Expression	p-value AV-Canal	Branchial Arches Expression	p-value Branchial Arches	Heart Tube Expression	p-value Heart Tube	Outflow Tract Expression	p-value Outflow Tract	Ventricle Expression	p-value Ventricle	DMP Expression
CAPZA1	.	.	0.4206646	0.0001901	.	.	.	.	.	.	.	.	.
CAPZA2	.	.	0.3680182	0.0012556	.	.	.	.	0.5413948	6.34E-07	.	.	.
CAPZB	.	.	.	.	.	.	.	.	.	.	0.4169863	0.0002192	yes
CARD14	.	.	.	.	.	.	.	.	0.4674919	2.68E-05	.	.	.
CARHSP1	.	.	0.3203636	0.0053908	.	.	.	.	0.5931855	2.56E-08	.	.	.
CARM1	.	.	.	.	.	.	.	.	.	.	.	.	yes
CARNS1	.	.	.	.	.	.	.	.	.	.	0.7010751	3.52E-12	.
CARS	.	.	.	.	.	.	.	.	.	.	0.7593964	4.44E-15	.
CASD1	.	.	.	.	.	.	.	.	0.6058311	1.07E-08	.	.	.
CASP12	.	.	0.4206949	0.0001899	.	.	.	.	.	.	.	.	.
CASP6	.	.	0.6147561	5.65E-09	.	.	.	.	0.3134231	0.0065451	.	.	.
CASP9	.	.	0.3695257	0.0011946	.	.	.	.	0.469032	2.50E-05	.	.	.
CASS4	.	.	.	.	.	.	.	.	.	.	0.7182058	5.89E-13	.
CASZ1	.	.	.	.	.	.	.	.	0.4571622	4.23E-05	.	.	.
CBFB	.	.	0.3859008	0.0006846	.	.	.	.	.	.	.	.	.
CBX2	.	.	.	.	.	.	.	.	.	.	.	.	yes
CBX5	.	.	0.321883	0.0051636	.	.	.	.	0.3637669	0.0014432	.	.	yes
CCAR1	.	.	.	.	.	.	.	.	.	.	.	.	yes
CCDC106	0.4687797	2.53E-05	.	.	.	.	.	.	.	.	.	.	.
CCDC108	.	.	.	.	.	.	.	.	.	.	0.4370909	9.87E-05	.
CCDC117	.	.	.	.	.	.	.	.	0.4861344	1.13E-05	.	.	.
CCDC12	.	.	0.4080749	0.0003073	.	.	.	.	.	.	.	.	.
CCDC122	.	.	.	.	.	.	.	.	.	.	0.5656035	1.51E-07	.
CCDC126	.	.	.	.	.	.	.	.	0.4751803	1.89E-05	.	.	.
CCDC13	.	.	.	.	.	.	.	.	.	.	0.5511994	3.60E-07	.
CCDC136	.	.	.	.	.	.	0.4645153	3.06E-05	.	.	.	.	.
CCDC138	.	.	.	.	.	.	.	.	.	.	0.6685803	7.55E-11	.
CCDC141	.	.	.	.	.	.	0.5068764	4.06E-06	.	.	.	.	.
CCDC166	.	.	0.3870461	0.0006577	.	.	.	.	.	.	.	.	.
CCDC170	.	.	.	.	.	.	.	.	0.5299057	1.20E-06	0.3340341	0.0036306	.
CCDC22	.	.	.	.	.	.	.	.	0.5600363	2.13E-07	.	.	.
CCDC25	.	.	0.4132199	0.0002531	.	.	0.3467272	0.0024752	0.3341758	0.0036154	.	.	.
CCDC28A	.	.	0.403918	0.0003586	.	.	.	.	.	.	.	.	.
CCDC36	.	.	.	.	0.5613721	1.96E-07	.	.	.	.	.	.	.
CCDC47	.	.	0.4036685	0.0003619	.	.	.	.	0.4784831	1.62E-05	.	.	.
CCDC58	.	.	.	.	.	.	.	.	0.6315789	1.60E-09	.	.	.
CCDC64	.	.	.	.	.	.	.	.	0.5745461	8.66E-08	.	.	.
CCDC66	.	.	.	.	.	.	.	.	0.5240408	1.65E-06	.	.	.
CCDC8	.	.	.	.	.	.	.	.	0.4883633	1.01E-05	.	.	.
CCDC87	.	.	.	.	.	.	.	.	.	.	0.4700606	2.38E-05	.
CCDC90B	.	.	.	.	.	.	.	.	0.5192817	2.13E-06	.	.	.
CCDC92	.	.	0.4504908	5.64E-05	.	.	.	.	.	.	.	.	.
CCDC94	.	.	0.3878011	0.0006406	.	.	.	.	.	.	.	.	.
CCL27	.	.	0.5424597	5.96E-07	.	.	.	.	.	.	.	.	.
CCL3L1	.	.	.	.	0.566635	1.42E-07	.	.	.	.	.	.	.
CCM2L	0.4601105	3.72E-05	.	.	.	.	.	.	.	.	.	.	.
CCNA2	.	.	.	.	.	.	.	.	.	.	.	.	yes
CCNB2	.	.	0.3940191	0.0005139	.	.	.	.	.	.	.	.	.
CCND1	.	.	0.3023058	0.0088494	.	.	.	.	0.5516269	3.51E-07	.	.	yes
CCND2	.	.	.	.	.	.	.	.	0.7345953	9.39E-14	.	.	yes
CCNK	.	.	.	.	.	.	.	.	0.4729158	2.09E-05	.	.	.
CCR1	.	.	.	.	.	.	.	.	0.6761591	3.82E-11	.	.	.
CCR5	.	.	.	.	.	.	.	.	.	.	0.6493079	3.91E-10	.
CCSER1	.	.	.	.	.	.	.	.	.	.	0.5646368	1.61E-07	.
CCT2	.	.	.	.	.	.	.	.	.	.	.	.	yes
CCT3	.	.	.	.	.	.	.	.	0.5670993	1.38E-07	.	.	yes
CCT4	.	.	0.3033459	0.0086072	.	.	.	.	0.6631623	1.21E-10	.	.	yes
CCT5	.	.	.	.	.	.	.	.	.	.	.	.	yes
CCT6A	.	.	.	.	.	.	0.3240644	0.0048522	0.6887938	1.17E-11	.	.	yes
CCT7	.	.	0.3248787	0.0047403	.	.	.	.	0.4259834	0.0001543	.	.	yes
CCT8	.	.	.	.	.	.	.	.	0.7241146	3.09E-13	.	.	yes
CCZ1B	.	.	.	.	.	.	.	.	0.5996397	1.65E-08	.	.	.
CD164	.	.	.	.	.	.	.	.	.	.	.	.	yes
CD19	.	.	.	.	.	.	.	.	.	.	0.5089276	3.66E-06	.
CD276	.	.	.	.	.	.	.	.	0.6011345	1.49E-08	.	.	.
CD2BP2	.	.	.	.	.	.	.	.	0.4470386	6.53E-05	.	.	.
CD300LD	.	.	.	.	0.4520159	5.28E-05	.	.	.	.	.	.	.
CD37	0.4578321	4.11E-05	.	.	.	.	.	.	.	.	.	.	.
CD40	.	.	.	.	.	.	.	.	0.5095075	3.55E-06	.	.	.
CD81	.	.	0.3230997	0.0049878	.	.	.	.	0.3028019	0.0087332	.	.	yes
CD83	.	.	.	.	.	.	.	.	.	.	0.6314642	1.62E-09	.
CD93	.	.	0.4102503	0.0002832	.	.	.	.	0.3173451	0.0058687	.	.	.
CD99L2	.	.	.	.	.	.	.	.	.	.	0.6978799	4.84E-12	.
CDAN1	.	.	0.4189437	0.0002032	.	.	.	.	.	.	.	.	.
CDC14B	.	.	0.5143164	2.77E-06	.	.	.	.	.	.	.	.	.

Gene	Atrial Expression	p-value Atrial	AV-Canal Expression	p-value AV-Canal	Branchial Arches Expression	p-value Branchial Arches	Heart Tube Expression	p-value Heart Tube	Outflow Tract Expression	p-value Outflow Tract	Ventricle Expression	p-value Ventricle	DMP Expression
CDC16	.	.	.	.	.	.	.	.	.	.	.	.	yes
CDC20	.	.	.	.	.	.	.	.	0.4831638	1.30E-05	.	.	.
CDC20B	.	.	.	.	.	.	.	.	0.6821265	2.21E-11	.	.	.
CDC27	.	.	.	.	.	.	.	.	0.5579846	2.40E-07	.	.	yes
CDC34	.	.	.	.	.	.	.	.	0.5800877	6.08E-08	.	.	.
CDC37	.	.	.	.	.	.	.	.	0.7129599	1.03E-12	.	.	yes
CDC40	.	.	.	.	.	.	.	.	.	.	0.6915654	9.00E-12	.
CDC42	.	.	.	.	.	.	.	.	.	.	.	.	yes
CDC42BPB	.	.	.	.	.	.	.	.	.	.	.	.	yes
CDC42EP5	.	.	0.623166	3.04E-09	.	.	.	.	.	.	.	.	.
CDH11	.	.	.	.	.	.	.	.	.	.	.	.	yes
CDH13	.	.	0.4047166	0.0003482	.	.	.	.	0.310936	0.0070088	.	.	.
CDH18	.	.	.	.	0.4482777	6.19E-05	.	.	.	.	.	.	.
CDH2	.	.	0.4845644	1.22E-05	.	.	.	.	0.3992792	0.000425	.	.	.
CDH20	.	.	0.4846635	1.21E-05	.	.	.	.	.	.	.	.	.
CDH23	.	.	.	.	.	.	.	.	0.4955802	7.14E-06	.	.	.
CDH3	.	.	.	.	.	.	.	.	0.5528789	3.26E-07	.	.	.
CDIP1	.	.	.	.	.	.	0.3902791	0.000587	0.61493	5.58E-09	.	.	.
CDK1	.	.	.	.	.	.	.	.	.	.	.	.	yes
CDK16	.	.	0.382011	0.0007834	.	.	.	.	.	.	.	.	yes
CDK2AP1	.	.	.	.	0.3016332	0.0090092	.	.	0.5422511	6.03E-07	.	.	.
CDK4	.	.	.	.	.	.	.	.	.	.	.	.	yes
CDK6	.	.	0.4090533	0.0002962	.	.	.	.	0.3750576	0.000993	.	.	.
CDK9	.	.	0.3779084	0.0009016	.	.	.	.	0.3008781	0.0091915	.	.	.
CDKL1	.	.	0.4446428	7.22E-05	.	.	.	.	.	.	.	.	.
CDKN1C	.	.	.	.	.	.	.	.	0.4853132	1.17E-05	.	.	yes
CDR2L	.	.	.	.	.	.	.	.	0.470776	2.31E-05	.	.	.
CDT1	.	.	.	.	0.486056	1.13E-05	.	.	.	.	.	.	.
CELF1	.	.	0.3437028	0.0027156	.	.	.	.	.	.	.	.	yes
CEND1	.	.	.	.	.	.	.	.	0.6126498	6.58E-09	.	.	.
CENPE	.	.	0.4882225	1.02E-05	.	.	.	.	.	.	.	.	.
CENPF	.	.	.	.	.	.	.	.	.	.	.	.	yes
CENPM	.	.	.	.	.	.	.	.	.	.	0.561598	1.93E-07	.
CENPN	.	.	.	.	.	.	.	.	0.4831196	1.30E-05	.	.	.
CENPO	.	.	.	.	.	.	.	.	.	.	0.4879172	1.04E-05	.
CENPT	.	.	.	.	.	.	.	.	0.5506751	3.71E-07	.	.	.
CEP19	.	.	.	.	.	.	.	.	0.442931	7.75E-05	.	.	.
CEP55	.	.	0.5111123	3.27E-06	.	.	.	.	.	.	.	.	.
CEP68	.	.	.	.	.	.	.	.	0.6634068	1.19E-10	.	.	.
CERS5	.	.	0.4155689	0.0002314	.	.	.	.	.	.	.	.	.
CES1	.	.	.	.	.	.	.	.	.	.	0.6952951	6.25E-12	.
CFDP1	.	.	.	.	.	.	.	.	.	.	.	.	yes
CFL1	.	.	.	.	.	.	.	.	0.6607398	1.49E-10	.	.	yes
CGNL1	.	.	0.3966744	0.0004671	.	.	.	.	0.538987	7.26E-07	.	.	yes
CHAF1A	.	.	.	.	.	.	.	.	.	.	.	.	yes
CHCHD10	.	.	.	.	.	.	0.318088	0.0057477	0.6122355	6.78E-09	.	.	.
CHCHD3	.	.	.	.	.	.	.	.	0.6505889	3.51E-10	.	.	.
CHD2	.	.	0.3980045	0.0004452	.	.	.	.	0.3120485	0.0067979	.	.	.
CHD3	.	.	.	.	.	.	.	.	.	.	.	.	yes
CHD4	.	.	.	.	.	.	.	.	0.7145499	8.73E-13	.	.	yes
CHD5	.	.	.	.	.	.	.	.	.	.	0.6220695	3.30E-09	.
CHD7	.	.	.	.	0.3601516	0.0016222	.	.	.	.	.	.	yes
CHD8	.	.	.	.	0.4543818	4.77E-05	.	.	.	.	.	.	yes
CHERP	.	.	0.3713795	0.0011232	.	.	.	.	.	.	.	.	yes
CHIT1	.	.	0.420826	0.0001889	.	.	.	.	.	.	.	.	.
CHML	.	.	0.5377241	7.80E-07	.	.	.	.	.	.	.	.	.
CHMP1A	.	.	.	.	.	.	.	.	0.6733036	4.95E-11	.	.	.
CHMP1B	.	.	.	.	.	.	.	.	0.4550907	4.63E-05	.	.	.
CHMP4B	.	.	0.2999468	0.0094209	.	.	.	.	0.6542362	2.59E-10	.	.	.
CHMP6	.	.	0.4688673	2.52E-05	.	.	.	.	.	.	.	.	.
CHRD	.	.	.	.	.	.	.	.	0.8307254	5.41E-20	.	.	.
CHRNA10	.	.	0.3881347	0.0006331	.	.	.	.	.	.	.	.	.
CHST14	.	.	0.3008699	0.0091935	.	.	.	.	0.6312272	1.65E-09	.	.	.
CHST15	.	.	.	.	.	.	.	.	0.4899395	9.40E-06	.	.	.
CHST8	.	.	.	.	.	.	.	.	.	.	0.6997042	4.04E-12	.
CHSY1	.	.	.	.	.	.	.	.	0.5936481	2.48E-08	.	.	.
CHTF8	.	.	.	.	.	.	.	.	.	.	.	.	yes
CIAO1	.	.	.	.	.	.	.	.	0.4378355	9.57E-05	.	.	.
CIAPIN1	.	.	.	.	.	.	.	.	0.7263887	2.39E-13	.	.	.
CIR1	.	.	0.4149203	0.0002372	.	.	.	.	.	.	.	.	.
CISD3	.	.	.	.	.	.	.	.	0.5628457	1.79E-07	.	.	.
CIT	.	.	.	.	.	.	.	.	.	.	0.5932889	2.54E-08	.
CITED1	.	.	.	.	.	.	.	.	.	.	0.6602847	1.55E-10	.
CITED4	.	.	0.470932	2.29E-05	.	.	.	.	.	.	.	.	.
CKAP4	.	.	0.3475045	0.0024165	.	.	.	.	0.6769187	3.57E-11	.	.	yes

Gene	Atrial Expression	p-value Atrial	AV-Canal Expression	p-value AV-Canal	Branchial Arches Expression	p-value Branchial Arches	Heart Tube Expression	p-value Heart Tube	Outflow Tract Expression	p-value Outflow Tract	Ventricle Expression	p-value Ventricle	DMP Expression
CKAP5	.	.	0.4055782	0.0003372	.	.	.	.	.	.	.	.	yes
CLASP1	.	.	.	.	.	.	.	.	.	.	.	.	yes
CLASP2	.	.	.	.	.	.	.	.	.	.	0.627014	2.27E-09	.
CLCN5	.	.	.	.	.	.	.	.	0.4446834	7.21E-05	.	.	.
CLDND1	.	.	0.4279668	0.0001426	.	.	.	.	.	.	.	.	.
CLEC2D	.	.	.	.	0.5070944	4.01E-06	.	.	.	.	.	.	.
CLIC1	.	.	0.4713055	2.25E-05	.	.	.	.	0.55931	2.22E-07	.	.	.
CLIC4	.	.	0.4920626	8.48E-06	.	.	.	.	0.6320192	1.55E-09	.	.	yes
CLIP3	.	.	.	.	0.4990881	6.01E-06	.	.	.	.	.	.	.
CLK3	.	.	0.4083362	0.0003043	.	.	.	.	.	.	.	.	.
CLN5	.	.	0.5230409	1.74E-06	.	.	.	.	0.4705412	2.33E-05	.	.	.
CLN6	.	.	.	.	.	.	.	.	0.5737702	9.10E-08	.	.	.
CLN8	.	.	0.5482195	4.28E-07	.	.	.	.	.	.	.	.	.
CLOCK	.	.	.	.	.	.	.	.	.	.	0.6915327	9.02E-12	.
CLPP	.	.	.	.	.	.	.	.	0.4671709	2.72E-05	.	.	.
CLPTM1	.	.	.	.	.	.	.	.	0.7511936	1.27E-14	.	.	.
CLPTM1L	.	.	0.4444788	7.27E-05	.	.	.	.	0.6521363	3.09E-10	.	.	.
CLTA	.	.	.	.	.	.	.	.	0.6604841	1.53E-10	.	.	.
CLTC	.	.	.	.	.	.	.	.	.	.	.	.	yes
CLUH	.	.	.	.	.	.	.	.	0.8185925	5.22E-19	.	.	.
CLVS2	.	.	.	.	.	.	0.4834159	1.28E-05	.	.	.	.	.
CMAS	.	.	0.3340244	0.0036317	.	.	.	.	0.545905	4.89E-07	.	.	.
CMTM5	.	.	.	.	.	.	0.5605009	2.07E-07	.	.	.	.	.
CMTM7	.	.	.	.	.	.	.	.	0.7463965	2.30E-14	.	.	.
CMTR1	.	.	.	.	.	.	.	.	0.4504221	5.65E-05	.	.	.
CNBP	.	.	.	.	.	.	.	.	.	.	.	.	yes
CNEP1R1	.	.	0.4087748	0.0002993	.	.	.	.	0.4214459	0.0001844	.	.	.
CNGA3	.	.	.	.	.	.	.	.	.	.	0.4680097	2.61E-05	.
CNIH1	.	.	0.3249695	0.0047279	.	.	.	.	0.7065333	2.02E-12	.	.	.
CNKSR2	.	.	.	.	.	.	.	.	0.5254997	1.53E-06	.	.	.
CNN1	.	.	.	.	.	.	.	.	0.7680528	1.40E-15	.	.	.
CNN2	.	.	0.3698941	0.0011801	.	.	.	.	0.7087505	1.61E-12	.	.	yes
CNN3	.	.	0.4298769	0.0001322	.	.	.	.	0.6084902	8.87E-09	.	.	yes
CNNM1	.	.	.	.	.	.	.	.	.	.	0.5673495	1.36E-07	.
CNOT1	.	.	.	.	0.5056764	4.31E-06	.	.	.	.	.	.	yes
CNOT10	.	.	0.3023654	0.0088354	.	.	.	.	0.5987887	1.75E-08	.	.	.
CNOT7	.	.	0.3929672	0.0005335	.	.	.	.	.	.	.	.	.
CNPY3	.	.	.	.	.	.	.	.	0.6935881	7.39E-12	.	.	.
CNST	.	.	.	.	.	.	.	.	0.5107417	3.33E-06	.	.	.
CNTNAP1	.	.	.	.	.	.	.	.	.	.	0.7002859	3.81E-12	.
CNTNAP3	.	.	.	.	.	.	.	.	.	.	0.7012598	3.45E-12	.
COA5	.	.	0.3516475	0.0021245	.	.	0.3086678	0.0074566	0.6310445	1.67E-09	.	.	.
COG6	.	.	.	.	.	.	.	.	0.5797495	6.21E-08	.	.	.
COG7	.	.	.	.	.	.	.	.	0.5331241	1.01E-06	.	.	.
COG8	.	.	.	.	.	.	.	.	0.5277532	1.35E-06	.	.	.
COL18A1	.	.	.	.	.	.	.	.	0.6885797	1.20E-11	.	.	yes
COL1A1	.	.	.	.	.	.	.	.	.	.	.	.	yes
COL1A2	.	.	.	.	.	.	.	.	.	.	.	.	yes
COL26A1	.	.	.	.	.	.	.	.	.	.	.	.	yes
COL2A1	.	.	.	.	.	.	.	.	0.9260268	3.46E-32	.	.	yes
COL3A1	.	.	.	.	.	.	.	.	.	.	.	.	yes
COL4A1	.	.	.	.	.	.	.	.	0.5086416	3.71E-06	.	.	yes
COL4A2	.	.	.	.	.	.	.	.	0.3752967	0.000985	.	.	yes
COL4A5	.	.	0.4917944	8.59E-06	.	.	.	.	.	.	.	.	.
COL4A6	.	.	0.4948974	7.39E-06	.	.	.	.	0.3319016	0.003866	.	.	.
COL5A1	.	.	.	.	.	.	.	.	0.4723413	2.15E-05	.	.	yes
COL9A3	.	.	0.5698349	1.16E-07	.	.	.	.	.	.	.	.	.
COLQ	.	.	.	.	.	.	.	.	0.7192236	5.28E-13	.	.	.
COMMD8	.	.	0.502355	5.10E-06	.	.	.	.	.	.	.	.	.
COMMD9	.	.	.	.	.	.	.	.	0.5912078	2.92E-08	.	.	.
COPA	.	.	0.4331389	0.0001159	.	.	.	.	.	.	.	.	yes
COPB1	.	.	0.390566	0.0005811	.	.	.	.	0.6569416	2.07E-10	.	.	.
COPB2	.	.	0.3608513	0.0015861	.	.	.	.	.	.	.	.	yes
COPG1	.	.	0.3171837	0.0058952	.	.	.	.	0.3606219	0.0015978	.	.	yes
COPG2	.	.	0.4408344	8.46E-05	.	.	.	.	.	.	.	.	.
COPS2	.	.	.	.	.	.	.	.	.	.	.	.	yes
COPS3	.	.	.	.	.	.	.	.	0.4542801	4.79E-05	.	.	.
COPS5	.	.	0.5539224	3.06E-07	.	.	.	.	.	.	.	.	.
COPS8	.	.	0.3831876	0.0007522	.	.	.	.	0.5777235	7.07E-08	.	.	.
COPZ1	.	.	.	.	.	.	.	.	0.5740221	8.95E-08	.	.	yes
COQ10B	.	.	0.4451157	7.08E-05	.	.	.	.	.	.	.	.	.
COQ3	.	.	.	.	.	.	.	.	0.4439873	7.42E-05	.	.	.
COQ6	.	.	.	.	.	.	.	.	0.5941698	2.40E-08	.	.	.
COQ7	.	.	0.3979745	0.0004457	.	.	0.3508422	0.0021787	.	.	.	.	.
CORO1C	.	.	.	.	.	.	.	.	0.8404458	7.71E-21	.	.	yes

Gene	Atrial Expression	p-value Atrial	AV-Canal Expression	p-value AV-Canal	Branchial Arches Expression	p-value Branchial Arches	Heart Tube Expression	p-value Heart Tube	Outflow Tract Expression	p-value Outflow Tract	Ventricle Expression	p-value Ventricle	DMP Expression
COX17	.	.	0.4924825	8.31E-06	.	.	.	.	0.4772047	1.72E-05	.	.	.
COX4I1	.	.	.	.	.	.	.	.	.	.	.	.	yes
COX4I2	.	.	.	.	.	.	.	.	0.7403808	4.75E-14	.	.	.
COX5A	.	.	0.3859738	0.0006828	.	.	.	.	.	.	.	.	.
COX5B	.	.	0.6481813	4.29E-10	.	.	.	.	.	.	.	.	.
COX6A1	.	.	0.3345859	0.0035718	.	.	.	.	0.7216609	4.05E-13	.	.	.
COX6A2	.	.	0.699902	3.96E-12	.	.	.	.	.	.	.	.	.
COX6B1	.	.	0.4033903	0.0003656	.	.	.	.	.	.	.	.	.
COX6C	.	.	0.5781075	6.90E-08	.	.	.	.	0.4879219	1.04E-05	.	.	.
COX7A1	.	.	0.5819088	5.40E-08	.	.	.	.	0.5764044	7.70E-08	.	.	.
COX7B	.	.	0.4317544	0.0001226	.	.	.	.	0.3093621	0.007317	.	.	.
COX8A	.	.	0.3972132	0.0004581	.	.	.	.	0.717305	6.50E-13	.	.	.
CPD	.	.	0.5262597	1.47E-06	.	.	.	.	.	.	.	.	.
CPEB1	0.5720136	1.02E-07	.	.	.	.	.	.	.	.	.	.	.
CPM	0.3209779	0.0052979	.	.	.	.	.	.	0.5087082	3.70E-06	.	.	.
CPNE5	.	.	0.4231283	0.0001727	.	.	.	.	.	.	.	.	.
CPSF6	.	.	.	.	.	.	.	.	.	.	.	.	yes
CPSF7	.	.	.	.	.	.	.	.	0.5680654	1.30E-07	.	.	.
CPT1B	.	.	.	.	.	.	0.3484083	0.0023499	0.6726203	5.26E-11	.	.	.
CRABP1	.	.	.	.	0.5024368	5.08E-06	.	.	.	.	.	.	yes
CREB3	.	.	0.3844332	0.0007205	.	.	.	.	.	.	.	.	.
CREB3L2	.	.	0.5208101	1.97E-06	.	.	.	.	0.2997236	0.0094766	.	.	.
CRELD1	.	.	0.5472791	4.52E-07	.	.	.	.	.	.	.	.	.
CRIP1	.	.	0.5378841	7.73E-07	.	.	.	.	0.3588893	0.0016893	.	.	.
CRIP2	.	.	.	.	.	.	.	.	0.4985398	6.17E-06	.	.	.
CRIPT	.	.	0.4287923	0.000138	.	.	.	.	0.4489748	6.01E-05	.	.	.
CRISP2	.	.	.	.	.	.	.	.	.	.	0.5508538	3.67E-07	.
CRK	.	.	.	.	.	.	.	.	.	.	.	.	yes
CRLF3	.	.	0.4094131	0.0002922	.	.	.	.	.	.	.	.	.
CRYBB3	.	.	.	.	.	.	.	.	0.4840813	1.24E-05	.	.	.
CS	0.3117231	0.006859	0.3394316	0.0030908	.	.	.	.	0.5162031	2.51E-06	.	.	yes
CSDE1	.	.	.	.	.	.	.	.	0.5698565	1.16E-07	.	.	yes
CSE1L	.	.	.	.	.	.	0.3950031	0.0004961	.	.	.	.	yes
CSNK1A1	.	.	.	.	.	.	.	.	0.324207	0.0048324	.	.	yes
CSNK1D	.	.	0.3467352	0.0024746	.	.	.	.	0.3499122	0.0022427	.	.	yes
CSNK1E	.	.	.	.	.	.	.	.	0.5138876	2.83E-06	.	.	.
CSNK1G2	.	.	.	.	.	.	.	.	0.3637678	0.0014432	.	.	yes
CSNK1G3	.	.	.	.	.	.	.	.	0.5045046	4.58E-06	.	.	.
CSNK2A2	.	.	0.4813545	1.41E-05	.	.	.	.	0.3887017	0.0006206	.	.	.
CSRNP1	.	.	.	.	.	.	.	.	0.5504489	3.76E-07	.	.	.
CSRP2	0.3321104	0.0038424	0.3079844	0.0075964	.	.	.	.	0.5445914	5.28E-07	.	.	.
CSTF1	.	.	.	.	.	.	.	.	0.4465924	6.65E-05	.	.	.
CSTF2	.	.	.	.	.	.	.	.	0.5660062	1.48E-07	.	.	.
CSTF2T	.	.	0.3572466	0.0017803	0.4812778	1.42E-05	.	.	.	.	.	.	.
CSTF3	.	.	0.3276532	0.0043759	.	.	.	.	0.6030248	1.30E-08	.	.	.
CTBP1	.	.	.	.	.	.	.	.	.	.	.	.	yes
CTBP2	.	.	.	.	.	.	.	.	.	.	0.6324402	1.50E-09	.
CTC1	0.4761436	1.80E-05	.	.	.	.	.	.	.	.	.	.	.
CTCF	.	.	.	.	.	.	.	.	0.3646573	0.0014019	.	.	yes
CTDNEP1	.	.	.	.	.	.	.	.	0.4621038	3.40E-05	.	.	.
CTDP1	.	.	.	.	0.4889934	9.84E-06	.	.	.	.	.	.	.
CTDSP2	.	.	0.3406209	0.0029819	.	.	.	.	.	.	.	.	yes
CTDSPL	.	.	.	.	.	.	.	.	0.4538956	4.87E-05	.	.	.
CTHRC1	.	.	0.5177335	2.31E-06	.	.	.	.	.	.	.	.	.
CTNNA1	.	.	.	.	.	.	.	.	.	.	.	.	yes
CTNNA3	.	.	.	.	.	.	.	.	0.4760637	1.81E-05	.	.	.
CTNNB1	.	.	.	.	.	.	.	.	.	.	.	.	yes
CTNND1	.	.	.	.	.	.	.	.	0.3225385	0.0050682	.	.	yes
CTPS2	.	.	0.4308908	0.0001269	.	.	.	.	0.3482684	0.0023601	.	.	.
CTSB	.	.	0.3177939	0.0057953	.	.	.	.	0.5354618	8.85E-07	.	.	.
CTSD	.	.	0.3347989	0.0035493	.	.	.	.	0.5756162	8.09E-08	.	.	.
CTSG	.	.	.	.	.	.	.	.	0.4608178	3.60E-05	.	.	.
CTTN	.	.	.	.	.	.	.	.	.	.	.	.	yes
CTTNBP2NL	.	.	0.4596345	3.79E-05	.	.	.	.	0.3519454	0.0021048	.	.	.
CTU2	.	.	.	.	.	.	.	.	0.5228552	1.76E-06	.	.	.
CTXN2	.	.	.	.	.	.	.	.	.	.	0.6833669	1.96E-11	.
CTXN3	.	.	.	.	.	.	.	.	.	.	0.4682992	2.58E-05	.
CUL1	.	.	.	.	.	.	0.588501	3.50E-08	.	.	.	.	yes
CUL3	.	.	.	.	.	.	.	.	0.5575233	2.47E-07	.	.	.
CUL4B	.	.	0.3185186	0.0056786	.	.	.	.	0.4679863	2.62E-05	.	.	yes
CUL7	.	.	.	.	.	.	.	.	0.5752383	8.29E-08	.	.	yes
CUTA	.	.	.	.	.	.	.	.	0.6667177	8.89E-11	.	.	.
CUTC	.	.	.	.	0.5102788	3.41E-06	.	.	.	.	.	.	.
CWF19L1	.	.	.	.	.	.	.	.	0.7146503	8.63E-13	.	.	.
CXCR4	.	.	0.3801607	0.0008349	.	.	.	.	.	.	.	.	.

Gene	Atrial Expression	p-value Atrial	AV-Canal Expression	p-value AV-Canal	Branchial Arches Expression	p-value Branchial Arches	Heart Tube Expression	p-value Heart Tube	Outflow Tract Expression	p-value Outflow Tract	Ventricle Expression	p-value Ventricle	DMP Expression
CXorf22	.	.	.	.	.	.	.	.	.	.	0.4629605	3.28E-05	.
CXorf56	.	.	0.479327	1.56E-05	.	.	.	.	0.4997317	5.82E-06	.	.	.
CXorf57	.	.	0.3042424	0.0084032	.	.	.	.	0.4440198	7.41E-05	.	.	.
CXorf58	.	.	0.4328157	0.0001174	.	.	.	.	.	.	.	.	.
CXXC1	.	.	0.3928561	0.0005357	.	.	.	.	0.3718953	0.0011041	.	.	.
CXXC5	.	.	0.5360398	8.57E-07	.	.	.	.	.	.	.	.	.
CYB561D2	.	.	0.3825848	0.0007681	.	.	.	.	.	.	.	.	.
CYB5B	.	.	.	.	.	.	.	.	0.6768422	3.59E-11	.	.	yes
CYC1	.	.	0.3510865	0.0021621	.	.	.	.	0.7713394	8.92E-16	.	.	.
CYHR1	.	.	0.4426484	7.85E-05	.	.	.	.	0.3391915	0.0031132	.	.	.
CYP1A2	.	.	.	.	.	.	.	.	0.451706	5.35E-05	.	.	.
CYP2E1	.	.	.	.	0.4802199	1.49E-05	.	.	0.3315915	0.0039014	.	.	.
CYP4F12	.	.	.	.	.	.	.	.	.	.	0.6280368	2.10E-09	.
CYR61	.	.	0.4913079	8.80E-06	.	.	.	.	0.3514536	0.0021375	.	.	.
CYTIP	.	.	.	.	.	.	.	.	.	.	0.6026185	1.34E-08	.
DAAM1	.	.	0.4509353	5.53E-05	.	.	.	.	.	.	.	.	.
DACT3	.	.	.	.	0.3572011	0.0017828	.	.	0.5858151	4.18E-08	.	.	.
DAD1	.	.	0.4406041	8.54E-05	.	.	.	.	0.5079976	3.83E-06	.	.	.
DAG1	.	.	0.5172201	2.38E-06	.	.	.	.	.	.	.	.	yes
DAP3	.	.	.	.	.	.	.	.	0.6185527	4.28E-09	.	.	.
DARS	.	.	0.366603	0.0013155	.	.	.	.	0.6088998	8.61E-09	.	.	.
DBI	.	.	0.385244	0.0007004	.	.	.	.	.	.	.	.	.
DBN1	.	.	.	.	0.611231	7.29E-09	.	.	0.3761472	0.0009571	.	.	yes
DBNL	.	.	.	.	.	.	.	.	0.4629115	3.28E-05	.	.	.
DBR1	.	.	.	.	.	.	0.4518763	5.31E-05	.	.	.	.	.
DCAF10	.	.	.	.	.	.	.	.	0.6745557	4.42E-11	.	.	.
DCAF13	.	.	0.4349205	0.0001078	.	.	.	.	0.4892283	9.73E-06	.	.	.
DCAF15	.	.	.	.	0.5834266	4.89E-08	.	.	.	.	.	.	.
DCAF7	.	.	.	.	.	.	.	.	.	.	.	.	yes
DCAKD	.	.	.	.	0.470839	2.30E-05	.	.	.	.	.	.	yes
DCBLD2	.	.	0.3668414	0.0013052	.	.	.	.	.	.	.	.	yes
DCC	.	.	0.380077	0.0008373	.	.	.	.	.	.	.	.	.
DCDC5	.	.	.	.	.	.	.	.	.	.	0.6788129	3.00E-11	.
DCHS1	.	.	.	.	.	.	.	.	.	.	.	.	yes
DCP1A	.	.	.	.	0.5698617	1.16E-07	.	.	.	.	.	.	.
DCP2	.	.	.	.	.	.	.	.	.	.	.	.	yes
DCTN1	.	.	.	.	.	.	.	.	0.5945678	2.33E-08	.	.	yes
DCTN2	.	.	.	.	.	.	.	.	.	.	.	.	yes
DCTN4	.	.	.	.	.	.	.	.	0.5544393	2.97E-07	.	.	.
DCTN6	.	.	0.3781841	0.0008932	.	.	.	.	.	.	.	.	.
DDAH1	.	.	.	.	.	.	.	.	0.611874	6.96E-09	.	.	.
DDAH2	.	.	.	.	.	.	.	.	0.5476664	4.42E-07	.	.	yes
DDB1	.	.	.	.	.	.	.	.	0.6004659	1.56E-08	.	.	yes
DDHD2	.	.	0.3764404	0.0009476	.	.	.	.	.	.	.	.	.
DDOST	.	.	.	.	.	.	.	.	0.5443832	5.34E-07	.	.	yes
DDR1	.	.	0.3893289	0.000607	.	.	.	.	.	.	.	.	.
DDT	.	.	.	.	.	.	.	.	0.5024455	5.08E-06	.	.	.
DDX1	.	.	.	.	.	.	.	.	.	.	.	.	yes
DDX17	.	.	.	.	.	.	.	.	.	.	.	.	yes
DDX21	.	.	.	.	.	.	.	.	0.4911426	8.87E-06	.	.	yes
DDX39B	.	.	.	.	.	.	.	.	.	.	.	.	yes
DDX3X	.	.	.	.	0.4049887	0.0003447	.	.	.	.	.	.	yes
DDX42	.	.	.	.	0.4038437	0.0003596	.	.	.	.	.	.	yes
DDX46	.	.	0.362871	0.0014858	.	.	.	.	0.4192962	0.0002005	.	.	yes
DDX5	.	.	.	.	.	.	.	.	.	.	.	.	yes
DDX51	.	.	.	.	.	.	.	.	0.6461317	5.07E-10	.	.	.
DDX54	.	.	.	.	.	.	.	.	0.6207877	3.63E-09	.	.	.
DDX56	.	.	.	.	.	.	.	.	0.4889989	9.84E-06	.	.	.
DDX6	.	.	.	.	.	.	.	.	.	.	.	.	yes
DECR1	.	.	0.4130457	0.0002548	.	.	.	.	0.4735707	2.03E-05	.	.	.
DEDD	.	.	.	.	.	.	.	.	0.6412135	7.53E-10	.	.	.
DEGS1	.	.	0.3364324	0.0033812	.	.	.	.	0.7472358	2.07E-14	.	.	.
DEK	.	.	.	.	.	.	.	.	.	.	.	.	yes
DENND2A	.	.	.	.	.	.	.	.	0.5740406	8.94E-08	.	.	.
DENND4B	.	.	.	.	.	.	.	.	0.4807053	1.46E-05	.	.	.
DENND5B	.	.	.	.	.	.	.	.	0.6363586	1.11E-09	.	.	.
DENND6A	.	.	.	.	.	.	0.4721219	2.17E-05	.	.	.	.	.
DENR	.	.	.	.	.	.	.	.	0.5045551	4.57E-06	.	.	.
DEPDC1B	.	.	.	.	.	.	.	.	0.3498318	0.0022484	0.4658914	2.88E-05	.
DERA	.	.	.	.	.	.	.	.	0.451529	5.39E-05	.	.	.
DES	.	.	0.3386498	0.0031642	.	.	.	.	0.5876819	3.70E-08	.	.	.
DESI2	.	.	.	.	.	.	.	.	0.637334	1.02E-09	.	.	.
DFFA	.	.	.	.	.	.	.	.	0.5663761	1.44E-07	.	.	.
DGCR14	.	.	.	.	.	.	.	.	0.5142907	2.77E-06	.	.	.
DGCR2	.	.	.	.	.	.	.	.	.	.	.	.	yes

Gene	Atrial Expression	p-value Atrial	AV-Canal Expression	p-value AV-Canal	Branchial Arches Expression	p-value Branchial Arches	Heart Tube Expression	p-value Heart Tube	Outflow Tract Expression	p-value Outflow Tract	Ventricle Expression	p-value Ventricle	DMP Expression
DHCR24	.	.	.	.	0.4498721	5.79E-05	.	.	.	.	.	.	yes
DHDH	.	.	0.5179475	2.29E-06	.	.	.	.	0.3647835	0.0013962	.	.	.
DHRS1	.	.	.	.	.	.	.	.	0.4389672	9.14E-05	.	.	.
DHRS3	.	.	.	.	.	.	.	.	.	.	0.6936564	7.34E-12	.
DHRS7	.	.	0.4881238	1.03E-05	.	.	.	.	.	.	.	.	.
DHTKD1	.	.	.	.	.	.	.	.	.	.	0.6613593	1.42E-10	.
DHX15	.	.	.	.	.	.	.	.	.	.	.	.	yes
DHX35	.	.	.	.	.	.	.	.	.	.	0.4648844	3.01E-05	.
DHX37	.	.	.	.	.	.	.	.	0.7312489	1.38E-13	.	.	.
DHX8	.	.	.	.	0.4963225	6.89E-06	.	.	.	.	.	.	.
DHX9	.	.	.	.	.	.	.	.	0.4242691	0.0001651	.	.	yes
DICER1	.	.	0.4066624	0.0003239	.	.	.	.	.	.	.	.	yes
DIDO1	.	.	.	.	.	.	.	.	.	.	0.5350676	9.04E-07	.
DKK3	.	.	0.3791224	0.000865	.	.	.	.	.	.	.	.	.
DLAT	.	.	.	.	.	.	.	.	0.6507501	3.47E-10	.	.	.
DLC1	.	.	.	.	.	.	.	.	0.5481645	4.29E-07	.	.	.
DLG2	.	.	.	.	.	.	.	.	.	.	0.4794834	1.54E-05	.
DLK1	.	.	.	.	.	.	.	.	.	.	.	.	yes
DLL1	.	.	.	.	.	.	.	.	0.4442463	7.34E-05	.	.	.
DLL4	.	.	.	.	.	.	.	.	0.5463685	4.76E-07	.	.	.
DLST	.	.	0.6080676	9.14E-09	.	.	.	.	.	.	.	.	yes
DLX2	.	.	.	.	0.5005408	5.59E-06	.	.	.	.	.	.	.
DLX3	.	.	.	.	0.4497607	5.82E-05	.	.	.	.	.	.	.
DLX5	.	.	.	.	0.6810517	2.44E-11	.	.	.	.	0.3002545	0.0093446	.
DLX6	.	.	.	.	0.6916813	8.89E-12	.	.	.	.	.	.	.
DMTN	.	.	.	.	.	.	.	.	.	.	0.6711366	6.01E-11	.
DNAJA2	.	.	0.3803402	0.0008297	0.4077043	0.0003116	.	.	0.4230023	0.0001735	.	.	yes
DNAJB1	.	.	.	.	.	.	.	.	0.6577207	1.93E-10	.	.	.
DNAJB11	.	.	0.3861909	0.0006777	.	.	.	.	0.5399214	6.89E-07	.	.	.
DNAJB12	.	.	.	.	.	.	.	.	0.4848397	1.20E-05	.	.	.
DNAJB4	.	.	.	.	.	.	.	.	0.471983	2.18E-05	.	.	.
DNAJC10	.	.	0.4751883	1.88E-05	.	.	.	.	0.629799	1.84E-09	.	.	yes
DNAJC11	.	.	0.301514	0.0090378	0.3707228	0.0011481	.	.	0.4804199	1.48E-05	.	.	.
DNAJC14	.	.	0.3137231	0.006491	.	.	.	.	0.5705975	1.11E-07	.	.	.
DNAJC17	.	.	.	.	.	.	.	.	0.6239782	2.86E-09	.	.	.
DNAJC4	.	.	.	.	.	.	.	.	0.4687403	2.53E-05	.	.	.
DNAJC5	.	.	0.311059	0.0069852	.	.	.	.	.	.	.	.	yes
DNAJC7	.	.	0.4850285	1.19E-05	.	.	.	.	.	.	.	.	yes
DNASE2	.	.	.	.	.	.	.	.	0.4448286	7.16E-05	.	.	.
DNM1L	.	.	0.37903	0.0008678	.	.	.	.	.	.	.	.	.
DNM2	.	.	.	.	.	.	.	.	0.9082631	5.85E-29	.	.	.
DNMT1	.	.	.	.	.	.	.	.	.	.	.	.	yes
DNMT3A	.	.	.	.	.	.	.	.	0.4899158	9.41E-06	.	.	yes
DNMT3B	.	.	.	.	.	.	.	.	.	.	0.6778941	3.26E-11	.
DNPEP	.	.	.	.	.	.	.	.	0.7353283	8.62E-14	.	.	.
DOCK6	.	.	.	.	.	.	.	.	0.7425443	3.67E-14	.	.	.
DOK4	.	.	.	.	.	.	.	.	0.5880313	3.61E-08	.	.	.
DOK5	.	.	.	.	0.4460877	6.79E-05	.	.	.	.	.	.	.
DPF2	.	.	0.3908059	0.0005762	.	.	.	.	.	.	.	.	.
DPF3	.	.	.	.	.	.	.	.	0.8916596	1.73E-26	.	.	.
DPH2	.	.	0.4145196	0.0002409	.	.	.	.	.	.	.	.	.
DPH5	.	.	.	.	0.5538737	3.07E-07	.	.	.	.	.	.	.
DPM2	.	.	.	.	.	.	.	.	0.8493695	1.14E-21	.	.	.
DPP3	.	.	0.3820606	0.0007821	.	.	.	.	0.446395	6.71E-05	.	.	yes
DPY30	.	.	0.3886447	0.0006218	.	.	.	.	.	.	.	.	.
DPYSL3	.	.	0.525768	1.51E-06	.	.	.	.	0.5163618	2.49E-06	.	.	.
DRD3	.	.	0.4581947	4.04E-05	.	.	.	.	.	.	.	.	.
DRD4	.	.	.	.	.	.	.	.	0.4784459	1.62E-05	.	.	.
DROSHA	.	.	.	.	.	.	.	.	.	.	0.4085984	0.0003013	yes
DSCAML1	.	.	0.3870466	0.0006577	.	.	.	.	.	.	.	.	.
DSEL	.	.	.	.	.	.	.	.	0.5052531	4.41E-06	.	.	.
DSP	.	.	.	.	.	.	.	.	0.5110112	3.28E-06	.	.	yes
DSPP	.	.	.	.	.	.	.	.	.	.	0.5277869	1.35E-06	.
DSTN	.	.	0.5892424	3.33E-08	.	.	.	.	0.4723074	2.15E-05	.	.	yes
DTX3	.	.	.	.	.	.	.	.	0.565049	1.57E-07	.	.	.
DUSP11	.	.	0.4583264	4.02E-05	.	.	.	.	0.33649	0.0033754	.	.	.
DUSP23	.	.	0.4636339	3.18E-05	.	.	.	.	.	.	.	.	.
DUSP27	.	.	.	.	.	.	.	.	0.6266048	2.35E-09	.	.	.
DUSP3	.	.	.	.	.	.	.	.	0.7048462	2.40E-12	.	.	.
DUSP6	.	.	.	.	0.3798784	0.000843	.	.	0.5912718	2.91E-08	.	.	.
DUSP7	.	.	.	.	0.5370662	8.09E-07	.	.	.	.	.	.	.
DUSP9	.	.	.	.	0.4262393	0.0001528	.	.	.	.	.	.	yes
DUT	.	.	.	.	0.552621	3.31E-07	.	.	.	.	.	.	yes
DVL1	.	.	.	.	.	.	.	.	0.4581239	4.05E-05	.	.	.
DVL2	.	.	0.5028192	4.98E-06	.	.	.	.	.	.	.	.	.

Gene	Atrial Expression	p-value Atrial	AV-Canal Expression	p-value AV-Canal	Branchial Arches Expression	p-value Branchial Arches	Heart Tube Expression	p-value Heart Tube	Outflow Tract Expression	p-value Outflow Tract	Ventricle Expression	p-value Ventricle	DMP Expression
DVL3	.	.	.	.	.	.	.	.	0.5028565	4.97E-06	.	.	.
DYNC1H1	.	.	.	.	0.3358673	0.0034385	.	.	0.3324102	0.0038087	.	.	yes
DYNC1LI1	.	.	.	.	.	.	.	.	0.6847261	1.73E-11	.	.	.
DYNC1LI2	.	.	0.5166324	2.45E-06	.	.	.	.	0.4108916	0.0002764	.	.	.
DYNLL1	.	.	0.3698008	0.0011838	.	.	.	.	0.5997742	1.63E-08	.	.	.
DYNLL2	.	.	.	.	.	.	.	.	0.354383	0.0019495	.	.	yes
DYNLRB1	.	.	.	.	.	.	.	.	0.6129428	6.45E-09	.	.	.
DYRK2	.	.	.	.	.	.	.	.	0.6734366	4.89E-11	.	.	.
DYRK3	0.4587174	3.95E-05	.	.	.	.	.	.	.	.	.	.	.
E2F1	.	.	0.4110743	0.0002745	0.5659125	1.49E-07	.	.	.	.	.	.	.
E2F3	.	.	0.5316296	1.09E-06	.	.	.	.	.	.	.	.	.
E2F7	.	.	.	.	.	.	.	.	0.6007955	1.52E-08	.	.	.
EBF2	.	.	.	.	.	.	.	.	.	.	0.6749335	4.27E-11	.
ECE1	.	.	.	.	.	.	.	.	0.6516691	3.21E-10	.	.	yes
ECHS1	.	.	.	.	.	.	.	.	0.4682264	2.59E-05	.	.	.
ECM1	.	.	0.3296441	0.00413	.	.	.	.	.	.	0.4747682	1.92E-05	.
EDIL3	.	.	0.5379942	7.68E-07	.	.	.	.	.	.	.	.	.
EDN1	.	.	0.4086081	0.0003012	.	.	.	.	0.6071359	9.76E-09	.	.	.
EEF1A2	.	.	0.4972056	6.59E-06	.	.	.	.	.	.	.	.	.
EEF1B2	.	.	.	.	.	.	0.3471847	0.0024405	0.4040197	0.0003572	.	.	yes
EEF1D	.	.	.	.	.	.	.	.	.	.	0.5511764	3.60E-07	yes
EEF1G	.	.	0.376681	0.00094	.	.	.	.	0.3785493	0.0008821	.	.	.
EEF2	.	.	.	.	.	.	.	.	0.6597675	1.62E-10	.	.	yes
EEPD1	.	.	0.4008497	0.0004014	.	.	.	.	.	.	.	.	.
EFCAB2	.	.	0.4546915	4.71E-05	.	.	.	.	.	.	.	.	.
EFCAB4B	.	.	.	.	.	.	.	.	0.5647291	1.60E-07	.	.	.
EFCC1	.	.	.	.	.	.	.	.	0.5516775	3.50E-07	.	.	.
EFHD2	.	.	0.5726915	9.74E-08	.	.	.	.	0.5478268	4.38E-07	.	.	.
EFNA2	.	.	.	.	0.608429	8.91E-09	.	.	0.3082414	0.0075436	.	.	.
EFNB2	.	.	.	.	.	.	.	.	0.8502594	9.39E-22	.	.	.
EFTUD2	.	.	.	.	.	.	.	.	.	.	.	.	yes
EHBP1L1	.	.	0.4672782	2.70E-05	.	.	.	.	.	.	.	.	.
EHMT1	.	.	.	.	.	.	.	.	.	.	.	.	yes
EHMT2	.	.	.	.	.	.	.	.	0.5623528	1.85E-07	.	.	yes
EID1	.	.	0.6023969	1.36E-08	.	.	.	.	.	.	.	.	yes
EID2	.	.	.	.	.	.	.	.	0.6846495	1.74E-11	.	.	.
EIF1	.	.	.	.	0.4998694	5.78E-06	.	.	.	.	.	.	.
EIF1AD	.	.	.	.	0.4982743	6.25E-06	.	.	0.3122362	0.0067629	.	.	.
EIF1AX	.	.	.	.	.	.	.	.	0.4384511	9.33E-05	.	.	.
EIF2S1	.	.	0.3239278	0.0048712	.	.	.	.	0.6490566	3.99E-10	.	.	.
EIF2S3	.	.	0.3990752	0.0004282	.	.	.	.	.	.	.	.	.
EIF3A	.	.	0.4530084	5.06E-05	.	.	.	.	0.5016146	5.29E-06	.	.	yes
EIF3B	.	.	.	.	0.378562	0.0008817	.	.	0.5825466	5.18E-08	.	.	yes
EIF3C	.	.	.	.	.	.	.	.	0.3921521	0.0005493	.	.	yes
EIF3D	.	.	.	.	.	.	.	.	0.3903345	0.0005858	.	.	yes
EIF3F	.	.	.	.	.	.	.	.	.	.	.	.	yes
EIF3G	.	.	0.3729738	0.001065	.	.	.	.	0.5578843	2.42E-07	.	.	.
EIF3I	.	.	0.3237192	0.0049003	.	.	.	.	0.3583545	0.0017184	.	.	yes
EIF3K	.	.	.	.	.	.	.	.	0.4047831	0.0003473	.	.	yes
EIF3L	.	.	.	.	.	.	.	.	0.5273494	1.38E-06	.	.	yes
EIF4A2	.	.	.	.	.	.	.	.	.	.	.	.	yes
EIF4B	.	.	.	.	.	.	.	.	.	.	.	.	yes
EIF4E	.	.	.	.	.	.	.	.	0.5771198	7.35E-08	.	.	.
EIF4EBP1	.	.	0.4951647	7.29E-06	.	.	.	.	0.4479476	6.28E-05	.	.	.
EIF4G1	.	.	.	.	.	.	.	.	.	.	.	.	yes
EIF4G2	.	.	0.3079893	0.0075954	.	.	.	.	0.3890204	0.0006137	.	.	yes
EIF4G3	.	.	.	.	.	.	.	.	.	.	.	.	yes
EIF4H	.	.	0.4490906	5.98E-05	.	.	.	.	0.4613094	3.53E-05	.	.	yes
EIF5	.	.	.	.	.	.	.	.	0.7226582	3.63E-13	.	.	yes
EIF5A	.	.	0.3880135	0.0006358	.	.	.	.	0.4649461	3.00E-05	.	.	yes
EIF5A2	.	.	0.6011846	1.48E-08	.	.	.	.	.	.	.	.	.
EIF5B	.	.	0.3480416	0.0023768	.	.	.	.	.	.	.	.	yes
ELAVL1	.	.	.	.	.	.	.	.	0.7444109	2.93E-14	.	.	yes
ELAVL2	.	.	.	.	.	.	.	.	.	.	0.4636688	3.18E-05	.
ELF5	.	.	.	.	.	.	.	.	.	.	0.5626547	1.81E-07	.
ELL2	.	.	0.395227	0.0004921	.	.	.	.	.	.	.	.	.
ELMOD3	.	.	.	.	.	.	.	.	0.5160782	2.52E-06	.	.	.
ELMSAN1	.	.	.	.	.	.	.	.	0.8841492	1.69E-25	.	.	.
ELP2	.	.	.	.	0.3390238	0.0031289	.	.	.	.	.	.	yes
ELP6	.	.	0.3431247	0.0027639	.	.	.	.	0.5244811	1.61E-06	.	.	.
ELTD1	.	.	.	.	.	.	.	.	.	.	0.4752896	1.88E-05	.
EMC1	.	.	0.4082768	0.000305	.	.	.	.	.	.	.	.	.
EMILIN1	.	.	.	.	.	.	.	.	0.3438843	0.0027006	.	.	yes
EMILIN2	.	.	0.4138557	0.0002471	.	.	.	.	.	.	.	.	.
EML4	.	.	.	.	.	.	.	.	0.7956985	2.45E-17	.	.	yes

Gene	Atrial Expression	p-value Atrial	AV-Canal Expression	p-value AV-Canal	Branchial Arches Expression	p-value Branchial Arches	Heart Tube Expression	p-value Heart Tube	Outflow Tract Expression	p-value Outflow Tract	Ventricle Expression	p-value Ventricle	DMP Expression
EMX1	.	.	.	.	0.576028	7.88E-08	.	.	.	.	.	.	.
ENC1	.	.	0.4591744	3.87E-05	.	.	.	.	0.3805491	0.0008238	.	.	.
ENDOG	.	.	.	.	.	.	.	.	0.5403511	6.72E-07	.	.	.
ENKD1	.	.	.	.	.	.	.	.	0.6396672	8.52E-10	.	.	.
ENO2	.	.	0.4235652	0.0001697	.	.	.	.	.	.	.	.	.
EP400	.	.	.	.	.	.	.	.	0.3726756	0.0010757	.	.	yes
EPB41	.	.	.	.	.	.	.	.	.	.	.	.	yes
EPB42	.	.	0.3841308	0.0007281	.	.	.	.	.	.	.	.	.
EPC1	.	.	0.4031616	0.0003687	.	.	.	.	.	.	.	.	.
EPDR1	.	.	.	.	.	.	.	.	0.5130226	2.96E-06	0.3045911	0.0083249	.
EPHB4	.	.	.	.	.	.	.	.	.	.	.	.	yes
EPHX2	.	.	.	.	.	.	.	.	0.6376337	1.00E-09	.	.	.
EPHX4	0.4602697	3.69E-05	.	.	.	.	.	.	.	.	.	.	.
EPRS	.	.	.	.	.	.	.	.	.	.	.	.	yes
EPT1	.	.	.	.	.	.	.	.	0.5498342	3.90E-07	.	.	.
ERBB2	.	.	0.3863013	0.0006751	.	.	.	.	0.361858	0.0015354	.	.	.
ERCC4	.	.	.	.	.	.	.	.	0.7039577	2.63E-12	.	.	.
ERF	.	.	0.437807	9.58E-05	.	.	.	.	0.5417927	6.19E-07	.	.	.
ERGIC1	.	.	.	.	.	.	.	.	0.4626798	3.32E-05	.	.	yes
ERICH1	.	.	0.3912826	0.0005665	.	.	.	.	0.3814652	0.0007983	.	.	.
ERICH2	.	.	0.4555919	4.53E-05	.	.	.	.	.	.	.	.	.
ERO1L	.	.	0.3947468	0.0005006	.	.	.	.	0.309232	0.007343	.	.	.
ESRRA	.	.	.	.	.	.	.	.	0.6025922	1.34E-08	.	.	.
ESYT1	.	.	0.3970051	0.0004616	.	.	.	.	.	.	.	.	.
ETF1	.	.	.	.	.	.	0.3806833	0.00082	0.5953385	2.21E-08	.	.	yes
ETFA	.	.	0.4388945	9.17E-05	.	.	.	.	0.6042384	1.20E-08	.	.	.
ETS1	.	.	0.3677096	0.0012685	.	.	.	.	.	.	.	.	yes
ETV2	.	.	0.466663	2.78E-05	.	.	.	.	.	.	.	.	.
ETV5	.	.	.	.	.	.	.	.	0.5195409	2.10E-06	.	.	.
EVX1	.	.	.	.	.	.	0.5697202	1.17E-07	.	.	.	.	.
EWSR1	.	.	.	.	.	.	.	.	.	.	.	.	yes
EXO1	.	.	.	.	0.5535301	3.13E-07	.	.	.	.	.	.	.
EXOC4	.	.	0.3873275	0.0006513	.	.	.	.	.	.	.	.	.
EXOSC6	.	.	.	.	.	.	.	.	0.5386335	7.41E-07	.	.	.
EXOSC9	.	.	0.5163668	2.49E-06	.	.	.	.	.	.	.	.	.
EXT1	.	.	0.4031925	0.0003683	.	.	.	.	0.5267665	1.43E-06	.	.	.
EXTL3	.	.	.	.	.	.	.	.	0.4613326	3.52E-05	.	.	.
EYA1	.	.	.	.	0.3046519	0.0083114	.	.	0.459007	3.90E-05	.	.	.
EZH2	.	.	.	.	.	.	.	.	.	.	.	.	yes
F2R	.	.	0.4642101	3.10E-05	.	.	.	.	0.6931551	7.71E-12	.	.	yes
F2RL2	.	.	0.4346736	0.0001089	.	.	.	.	.	.	.	.	.
F7	.	.	.	.	.	.	.	.	.	.	0.67393	4.68E-11	.
F8	.	.	.	.	.	.	.	.	0.5631593	1.76E-07	.	.	.
FABP3	.	.	0.4950757	7.32E-06	.	.	.	.	.	.	0.357108	0.0017881	.
FADD	.	.	0.3471254	0.002445	.	.	.	.	0.5316472	1.09E-06	.	.	.
FADS1	.	.	.	.	0.4067025	0.0003234	.	.	.	.	.	.	yes
FAM104A	.	.	0.3049692	0.0082408	.	.	.	.	0.6819616	2.24E-11	.	.	.
FAM109A	.	.	.	.	0.5139782	2.82E-06	.	.	.	.	.	.	.
FAM114A1	.	.	0.4567556	4.30E-05	.	.	.	.	.	.	.	.	.
FAM114A2	.	.	.	.	.	.	.	.	0.6014925	1.45E-08	.	.	.
FAM117B	.	.	.	.	.	.	.	.	0.4376018	9.67E-05	.	.	.
FAM118B	.	.	.	.	0.6105604	7.65E-09	.	.	.	.	.	.	.
FAM120A	.	.	.	.	.	.	.	.	.	.	.	.	yes
FAM120B	.	.	.	.	.	.	.	.	0.5652436	1.55E-07	.	.	.
FAM122B	.	.	0.4273865	0.000146	.	.	.	.	.	.	.	.	.
FAM126B	.	.	.	.	.	.	.	.	0.3839561	0.0007325	0.5395898	7.02E-07	.
FAM127C	.	.	.	.	.	.	.	.	0.4913452	8.78E-06	.	.	.
FAM13A	.	.	.	.	.	.	.	.	.	.	0.5079647	3.84E-06	.
FAM13B	.	.	.	.	.	.	.	.	0.5121974	3.09E-06	.	.	.
FAM161B	.	.	0.4555516	4.53E-05	.	.	.	.	.	.	.	.	.
FAM163A	.	.	0.470418	2.34E-05	.	.	.	.	0.6017853	1.42E-08	.	.	.
FAM168A	.	.	0.4360415	0.000103	.	.	.	.	.	.	.	.	.
FAM168B	.	.	.	.	.	.	.	.	.	.	.	.	yes
FAM171A1	.	.	.	.	.	.	.	.	0.628658	2.01E-09	.	.	.
FAM178A	.	.	.	.	.	.	.	.	0.5777652	7.05E-08	.	.	.
FAM179A	.	.	.	.	.	.	.	.	.	.	0.4994975	5.88E-06	.
FAM181A	.	.	0.4178169	0.0002123	.	.	.	.	.	.	.	.	.
FAM189A2	.	.	.	.	.	.	.	.	0.5380057	7.67E-07	.	.	.
FAM192A	.	.	.	.	.	.	.	.	0.5323349	1.05E-06	.	.	.
FAM198B	.	.	0.4464276	6.70E-05	.	.	.	.	.	.	.	.	.
FAM19A1	.	.	.	.	.	.	.	.	0.509237	3.60E-06	.	.	.
FAM203A	.	.	.	.	.	.	.	.	0.6874272	1.34E-11	.	.	.
FAM205A	.	.	0.3829838	0.0007576	.	.	.	.	.	.	.	.	.
FAM206A	.	.	.	.	.	.	.	.	0.5935423	2.50E-08	.	.	.
FAM20B	.	.	0.3891271	0.0006114	.	.	.	.	.	.	.	.	.

Gene	Atrial Expression	p-value Atrial	AV-Canal Expression	p-value AV-Canal	Branchial Arches Expression	p-value Branchial Arches	Heart Tube Expression	p-value Heart Tube	Outflow Tract Expression	p-value Outflow Tract	Ventricle Expression	p-value Ventricle	DMP Expression
FAM210A	.	.	0.3995292	0.0004212	.	.	.	.	0.3947632	0.0005003	.	.	.
FAM210B	.	.	.	.	.	.	.	.	.	.	0.4563896	4.37E-05	.
FAM213A	.	.	.	.	.	.	.	.	0.6358712	1.15E-09	.	.	.
FAM214B	.	.	0.3898798	0.0005953	.	.	.	.	.	.	.	.	.
FAM221B	.	.	0.4626359	3.32E-05	.	.	.	.	.	.	.	.	.
FAM26D	.	.	.	.	.	.	.	.	0.5036595	4.78E-06	.	.	.
FAM26E	.	.	0.4613849	3.51E-05	.	.	.	.	.	.	.	.	.
FAM43B	.	.	.	.	.	.	.	.	0.6744326	4.47E-11	.	.	.
FAM46D	.	.	.	.	.	.	.	.	.	.	0.5261248	1.48E-06	.
FAM58A	.	.	.	.	.	.	.	.	0.7526561	1.05E-14	.	.	.
FAM60A	.	.	.	.	.	.	.	.	0.6764763	3.71E-11	.	.	.
FAM69C	.	.	.	.	.	.	.	.	0.6665955	8.99E-11	.	.	.
FAM71E2	.	.	.	.	.	.	.	.	0.6689495	7.30E-11	.	.	.
FAM72A	.	.	0.4166866	0.0002217	.	.	.	.	.	.	.	.	.
FAM73B	.	.	0.3510125	0.0021671	.	.	.	.	0.4979351	6.36E-06	.	.	.
FAM76A	.	.	0.418288	0.0002084	.	.	.	.	.	.	.	.	.
FAM78A	.	.	0.6577965	1.92E-10	.	.	.	.	0.5361301	8.52E-07	.	.	.
FAM81A	.	.	0.3844043	0.0007212	.	.	.	.	.	.	.	.	.
FAM8A1	.	.	0.3343537	0.0035965	.	.	.	.	0.5777952	7.04E-08	.	.	.
FAM96B	.	.	0.4336395	0.0001136	.	.	.	.	0.5422606	6.03E-07	.	.	.
FAM98B	.	.	.	.	.	.	.	.	0.5267793	1.43E-06	.	.	.
FANCG	.	.	.	.	.	.	.	.	.	.	0.5107806	3.32E-06	.
FANCI	.	.	.	.	.	.	.	.	0.4877456	1.04E-05	.	.	.
FARP1	.	.	.	.	.	.	.	.	.	.	.	.	yes
FARSB	.	.	0.4016213	0.0003902	.	.	.	.	.	.	.	.	.
FASN	.	.	.	.	.	.	.	.	0.3553748	0.0018893	.	.	yes
FASTK	.	.	.	.	.	.	.	.	0.5121938	3.09E-06	.	.	.
FASTKD2	.	.	0.4152098	0.0002346	.	.	.	.	.	.	.	.	.
FASTKD3	.	.	.	.	.	.	.	.	0.515448	2.61E-06	.	.	.
FAT1	.	.	.	.	.	.	.	.	.	.	.	.	yes
FBLIM1	.	.	.	.	.	.	.	.	0.8814432	3.70E-25	.	.	.
FBLN1	.	.	0.4937668	7.81E-06	.	.	.	.	.	.	.	.	yes
FBLN2	.	.	0.3753916	0.0009818	.	.	.	.	.	.	.	.	.
FBN2	.	.	0.4558055	4.48E-05	.	.	.	.	.	.	.	.	yes
FBXL14	.	.	.	.	.	.	.	.	0.6784849	3.09E-11	.	.	.
FBXL17	.	.	.	.	.	.	.	.	0.614598	5.72E-09	.	.	.
FBXL19	.	.	.	.	0.3227135	0.005043	.	.	.	.	.	.	yes
FBXL3	.	.	.	.	.	.	.	.	0.4664799	2.80E-05	.	.	.
FBXL5	.	.	.	.	.	.	.	.	0.4749123	1.91E-05	.	.	.
FBXL6	.	.	.	.	.	.	.	.	0.5987231	1.76E-08	.	.	.
FBXL8	.	.	0.3997031	0.0004185	.	.	.	.	.	.	.	.	.
FBXO15	.	.	.	.	.	.	.	.	0.4931096	8.06E-06	.	.	.
FBXO18	.	.	0.3871217	0.000656	.	.	.	.	.	.	.	.	.
FBXO21	.	.	.	.	.	.	.	.	0.4707459	2.31E-05	.	.	.
FBXO27	.	.	.	.	.	.	.	.	.	.	0.5478427	4.37E-07	.
FBXO39	.	.	.	.	.	.	.	.	.	.	0.7885339	7.41E-17	.
FBXW7	.	.	.	.	.	.	.	.	0.4765669	1.77E-05	.	.	.
FBXW8	.	.	0.3872033	0.0006541	.	.	.	.	0.539442	7.08E-07	.	.	yes
FCGR1A	.	.	.	.	.	.	.	.	.	.	0.4609944	3.57E-05	.
FCRLA	.	.	.	.	.	.	.	.	.	.	0.6714725	5.83E-11	.
FDFT1	.	.	.	.	.	.	.	.	0.5428815	5.82E-07	.	.	.
FECH	.	.	0.4883196	1.02E-05	.	.	.	.	.	.	.	.	.
FEM1B	.	.	.	.	.	.	.	.	0.3398248	0.0030544	.	.	yes
FERMT2	0.4597358	3.78E-05	.	.	.	.	.	.	.	.	.	.	.
FFAR4	0.4620292	3.41E-05	.	.	.	.	.	.	.	.	.	.	.
FGD5	.	.	.	.	.	.	.	.	0.511514	3.20E-06	.	.	.
FGF11	.	.	.	.	.	.	0.3742182	0.0010214	0.4996559	5.84E-06	.	.	.
FGF12	.	.	.	.	.	.	.	.	.	.	0.5734831	9.26E-08	.
FGFR1	.	.	.	.	.	.	.	.	.	.	.	.	yes
FGFRL1	.	.	.	.	.	.	.	.	0.6293678	1.90E-09	.	.	.
FGL2	.	.	0.47374	2.01E-05	.	.	.	.	.	.	.	.	.
FHL2	.	.	0.6904115	1.01E-11	.	.	.	.	.	.	.	.	.
FIGN	.	.	.	.	0.5978837	1.86E-08	.	.	.	.	.	.	.
FIGNL2	.	.	.	.	0.5369408	8.15E-07	.	.	.	.	.	.	.
FITM1	.	.	0.4994303	5.90E-06	.	.	.	.	0.5023281	5.11E-06	.	.	.
FITM2	.	.	.	.	.	.	.	.	0.5898141	3.21E-08	.	.	.
FKBP14	.	.	.	.	.	.	.	.	0.4783221	1.63E-05	.	.	.
FKBP15	.	.	.	.	.	.	.	.	.	.	0.6948544	6.53E-12	.
FKBP1A	.	.	0.4957505	7.08E-06	.	.	.	.	.	.	.	.	yes
FKBP3	.	.	.	.	.	.	.	.	.	.	.	.	yes
FKBP4	.	.	.	.	.	.	.	.	0.5517192	3.49E-07	.	.	yes
FKBP8	.	.	.	.	.	.	.	.	.	.	.	.	yes
FKBP9	.	.	.	.	.	.	.	.	.	.	.	.	yes
FLI1	.	.	.	.	0.6257285	2.51E-09	.	.	.	.	.	.	.
FLJ22184	.	.	.	.	.	.	.	.	.	.	0.4606583	3.63E-05	.

Gene	Atrial Expression	p-value Atrial	AV-Canal Expression	p-value AV-Canal	Branchial Arches Expression	p-value Branchial Arches	Heart Tube Expression	p-value Heart Tube	Outflow Tract Expression	p-value Outflow Tract	Ventricle Expression	p-value Ventricle	DMP Expression
FLNA	.	.	0.5030878	4.92E-06	.	.	.	.	.	.	.	.	yes
FLNB	.	.	0.4379876	9.51E-05	.	.	.	.	.	.	.	.	yes
FLNC	.	.	.	.	.	.	.	.	.	.	.	.	yes
FLRT2	.	.	.	.	.	.	.	.	.	.	.	.	yes
FLVCR2	.	.	0.5311543	1.12E-06	.	.	.	.	0.3494847	0.0022728	.	.	.
FN1	.	.	0.3460553	0.0025269	.	.	.	.	0.3976798	0.0004504	.	.	yes
FNBP1L	.	.	.	.	.	.	.	.	.	.	.	.	yes
FNDC3B	.	.	0.5019847	5.20E-06	.	.	.	.	.	.	.	.	.
FNDC4	.	.	.	.	.	.	.	.	0.5146267	2.72E-06	.	.	.
FNTA	.	.	0.4297846	0.0001327	.	.	.	.	0.3467523	0.0024733	.	.	.
FOXC2	.	.	0.3532866	0.002018	.	.	.	.	0.5380045	7.67E-07	.	.	.
FOXL1	.	.	.	.	.	.	.	.	0.4416779	8.17E-05	.	.	.
FOXO1	.	.	.	.	.	.	.	.	0.453647	4.92E-05	.	.	.
FOXO4	.	.	.	.	0.393833	0.0005173	.	.	0.5291483	1.25E-06	.	.	.
FOXP1	.	.	.	.	.	.	.	.	.	.	.	.	yes
FOXP4	.	.	.	.	.	.	.	.	0.5317948	1.08E-06	.	.	yes
FRAS1	.	.	.	.	.	.	.	.	.	.	.	.	yes
FRAT2	.	.	.	.	.	.	.	.	0.556969	2.56E-07	.	.	.
FREM2	.	.	.	.	.	.	.	.	.	.	.	.	yes
FRG1	.	.	0.4020546	0.000384	.	.	.	.	0.5014942	5.33E-06	.	.	.
FRG2B	.	.	.	.	.	.	.	.	.	.	0.6271386	2.25E-09	.
FRS3	.	.	.	.	.	.	.	.	0.4393851	8.98E-05	.	.	.
FSCN1	.	.	.	.	0.597825	1.87E-08	.	.	0.3392896	0.003104	.	.	yes
FSD2	.	.	0.4792554	1.56E-05	.	.	0.3219358	0.0051559	0.4109385	0.0002759	.	.	.
FSTL1	.	.	0.3466679	0.0024797	.	.	.	.	0.3610843	0.0015742	.	.	yes
FSTL4	.	.	0.3992442	0.0004256	.	.	.	.	.	.	.	.	.
FTCD	.	.	0.4577791	4.12E-05	.	.	.	.	.	.	.	.	.
FTH1	.	.	.	.	.	.	.	.	.	.	.	.	yes
FTL	.	.	.	.	.	.	.	.	.	.	.	.	yes
FTSJ2	.	.	0.3774931	0.0009144	.	.	.	.	.	.	.	.	.
FUBP1	.	.	.	.	.	.	.	.	0.4760172	1.81E-05	.	.	yes
FUCA1	.	.	.	.	.	.	.	.	0.5146581	2.72E-06	.	.	.
FURIN	.	.	0.4238293	0.000168	.	.	.	.	.	.	.	.	.
FUS	.	.	.	.	0.9164012	2.38E-30	.	.	.	.	.	.	yes
FXN	.	.	.	.	.	.	.	.	0.6333811	1.40E-09	.	.	.
FXR1	.	.	.	.	.	.	.	.	0.7340071	1.01E-13	.	.	yes
FZD2	.	.	.	.	.	.	.	.	0.3414715	0.0029062	.	.	yes
FZD7	.	.	.	.	.	.	.	.	.	.	.	.	yes
FZD8	.	.	.	.	.	.	.	.	0.4999097	5.77E-06	.	.	.
G0S2	.	.	.	.	.	.	.	.	0.5838928	4.75E-08	.	.	.
G3BP1	.	.	.	.	.	.	.	.	0.4540606	4.84E-05	.	.	yes
G3BP2	.	.	0.5511037	3.62E-07	.	.	.	.	.	.	.	.	yes
G6PC3	.	.	.	.	.	.	.	.	0.585938	4.15E-08	.	.	.
GAB1	.	.	0.3929824	0.0005333	.	.	.	.	0.4098285	0.0002877	.	.	.
GABPA	.	.	0.5296231	1.22E-06	.	.	.	.	.	.	.	.	.
GADD45B	.	.	0.4012395	0.0003957	.	.	.	.	0.5257302	1.51E-06	.	.	.
GADD45GIP1	.	.	0.3938224	0.0005175	.	.	.	.	0.4023734	0.0003796	.	.	.
GAL	.	.	0.4226873	0.0001757	.	.	.	.	.	.	.	.	.
GALK1	.	.	0.3195345	0.0055185	.	.	.	.	0.6963267	5.65E-12	.	.	.
GALNS	.	.	0.4005605	0.0004056	.	.	.	.	.	.	.	.	.
GALNT1	.	.	.	.	.	.	.	.	0.8866692	8.02E-26	.	.	.
GALNT13	.	.	.	.	.	.	.	.	.	.	0.6879119	1.28E-11	.
GANAB	.	.	.	.	.	.	.	.	.	.	.	.	yes
GAPDH	0.4641325	3.11E-05	.	.	.	.	.	.	.	.	.	.	.
GAPDHS	.	.	.	.	.	.	.	.	0.5595999	2.18E-07	.	.	.
GARS	.	.	.	.	.	.	.	.	.	.	.	.	yes
GART	.	.	.	.	.	.	.	.	.	.	.	.	yes
GAS2L1	.	.	.	.	0.4559941	4.45E-05	.	.	.	.	.	.	.
GAS2L3	.	.	.	.	.	.	.	.	0.4479528	6.28E-05	.	.	.
GATA1	.	.	0.3878905	0.0006385	.	.	.	.	.	.	.	.	.
GATA3	.	.	.	.	.	.	.	.	0.7369956	7.09E-14	.	.	.
GATA4	.	.	0.5558913	2.72E-07	.	.	.	.	0.3369647	0.0033279	.	.	.
GATA5	.	.	0.5864782	4.00E-08	.	.	.	.	0.3708528	0.0011431	.	.	.
GATA6	.	.	0.4992966	5.94E-06	.	.	.	.	.	.	.	.	.
GATAD1	.	.	0.4423625	7.94E-05	.	.	.	.	0.6032035	1.29E-08	.	.	.
GATAD2A	.	.	.	.	.	.	.	.	0.550788	3.68E-07	.	.	yes
GATC	.	.	.	.	.	.	.	.	0.4544133	4.76E-05	.	.	.
GBA2	.	.	0.378387	0.000887	.	.	.	.	.	.	.	.	.
GBP4	.	.	0.5301076	1.19E-06	.	.	.	.	.	.	.	.	.
GCDH	.	.	0.6129567	6.44E-09	.	.	.	.	.	.	.	.	.
GCFC2	.	.	.	.	0.4441949	7.36E-05	.	.	.	.	.	.	.
GCLC	.	.	.	.	.	.	.	.	0.4758207	1.83E-05	.	.	.
GCN1L1	.	.	0.3095023	0.007289	.	.	.	.	0.3931526	0.00053	.	.	yes
GCSAM	.	.	.	.	.	.	.	.	.	.	0.6409199	7.71E-10	.
GDF6	.	.	0.6615865	1.39E-10	.	.	.	.	.	.	.	.	.

Gene	Atrial Expression	p-value Atrial	AV-Canal Expression	p-value AV-Canal	Branchial Arches Expression	p-value Branchial Arches	Heart Tube Expression	p-value Heart Tube	Outflow Tract Expression	p-value Outflow Tract	Ventricle Expression	p-value Ventricle	DMP Expression
GDI1	.	.	.	.	.	.	.	.	0.5031024	4.91E-06	.	.	.
GDI2	.	.	0.3525248	0.0020669	.	.	.	.	.	.	.	.	yes
GDPGP1	.	.	.	.	.	.	.	.	0.6456177	5.28E-10	.	.	.
GEM	.	.	0.3941071	0.0005123	.	.	.	.	0.6217498	3.38E-09	.	.	.
GEMIN6	.	.	.	.	.	.	.	.	0.5091966	3.61E-06	.	.	.
GFER	.	.	.	.	0.482891	1.32E-05	.	.	.	.	.	.	.
GFI1	.	.	.	.	.	.	.	.	.	.	0.5365409	8.33E-07	.
GFM1	.	.	0.5446109	5.27E-07	.	.	.	.	0.5568278	2.58E-07	.	.	.
GFOD1	.	.	0.3864933	0.0006706	.	.	.	.	0.3964884	0.0004702	.	.	.
GFPT2	.	.	0.5821381	5.32E-08	.	.	.	.	.	.	.	.	.
GFRA1	.	.	.	.	.	.	.	.	.	.	0.7027339	2.98E-12	.
GGA2	.	.	.	.	.	.	.	.	.	.	.	.	yes
GGCX	.	.	.	.	.	.	.	.	0.8273446	1.04E-19	.	.	.
GHDC	.	.	.	.	.	.	.	.	0.4693997	2.46E-05	.	.	.
GHITM	.	.	0.5407884	6.56E-07	.	.	.	.	0.3457382	0.0025516	.	.	yes
GID4	.	.	0.2987719	0.0097174	.	.	.	.	0.5570612	2.54E-07	.	.	.
GID8	.	.	0.409611	0.0002901	.	.	.	.	.	.	.	.	.
GIMAP5	.	.	0.3859861	0.0006826	.	.	.	.	.	.	.	.	.
GINS1	.	.	.	.	.	.	0.4390752	9.10E-05	.	.	.	.	.
GIT2	.	.	.	.	.	.	.	.	0.4656449	2.91E-05	.	.	.
GJA1	.	.	.	.	0.5275587	1.37E-06	.	.	.	.	.	.	yes
GJA4	.	.	.	.	.	.	.	.	0.7995115	1.34E-17	.	.	.
GJA5	.	.	.	.	.	.	.	.	0.4903597	9.21E-06	0.4909874	8.94E-06	.
GJC3	.	.	0.4079904	0.0003082	.	.	.	.	.	.	.	.	.
GLDC	.	.	.	.	.	.	.	.	0.5430765	5.76E-07	.	.	.
GLE1	.	.	.	.	.	.	.	.	0.555311	2.82E-07	.	.	.
GLIPR1L2	.	.	0.5119748	3.12E-06	.	.	.	.	.	.	.	.	.
GLIPR2	.	.	0.418495	0.0002068	.	.	.	.	0.7270758	2.22E-13	.	.	.
GLMN	.	.	.	.	.	.	.	.	0.4879788	1.03E-05	.	.	.
GLO1	.	.	0.3437339	0.002713	.	.	.	.	0.5215727	1.89E-06	.	.	.
GLRA4	.	.	.	.	.	.	0.4663949	2.81E-05	.	.	.	.	.
GLRX5	.	.	.	.	.	.	.	.	0.7576747	5.55E-15	.	.	.
GLT8D1	.	.	0.4535626	4.94E-05	.	.	.	.	.	.	.	.	.
GLTSCR2	.	.	.	.	.	.	.	.	0.5064795	4.14E-06	.	.	.
GLYR1	.	.	.	.	.	.	.	.	.	.	.	.	yes
GM2A	.	.	0.3922692	0.000547	.	.	.	.	0.3730876	0.0010609	.	.	.
GMFB	.	.	.	.	.	.	.	.	.	.	.	.	yes
GMPPB	.	.	0.4480964	6.24E-05	.	.	.	.	0.3481839	0.0023663	.	.	.
GMPR	.	.	0.4546706	4.71E-05	.	.	.	.	0.537517	7.89E-07	.	.	.
GNA11	.	.	.	.	.	.	.	.	0.6222821	3.25E-09	.	.	.
GNA12	.	.	.	.	.	.	.	.	.	.	0.6013407	1.47E-08	.
GNAI2	.	.	.	.	.	.	.	.	.	.	.	.	yes
GNAI3	.	.	0.5005004	5.60E-06	.	.	.	.	.	.	.	.	yes
GNAO1	.	.	0.3774046	0.0009172	.	.	.	.	.	.	.	.	.
GNAT2	0.5688654	1.24E-07	.	.	.	.	.	.	.	.	.	.	.
GNB1	.	.	.	.	.	.	.	.	0.5761537	7.82E-08	.	.	yes
GNB2	.	.	0.3767466	0.0009379	.	.	.	.	.	.	.	.	yes
GNB2L1	.	.	.	.	.	.	.	.	0.4255143	0.0001572	.	.	yes
GNB3	.	.	0.5453878	5.04E-07	.	.	.	.	.	.	.	.	.
GNB4	.	.	0.5857147	4.21E-08	.	.	.	.	0.5003882	5.63E-06	.	.	.
GNG7	.	.	.	.	.	.	.	.	0.4848875	1.20E-05	.	.	.
GNPAT	.	.	0.3551003	0.0019058	.	.	0.3076024	0.0076755	0.5090061	3.64E-06	.	.	yes
GNPDA2	.	.	0.4353343	0.000106	.	.	.	.	.	.	.	.	.
GNPTAB	.	.	.	.	.	.	.	.	0.6861625	1.51E-11	.	.	.
GNS	.	.	.	.	.	.	.	.	0.4508636	5.55E-05	.	.	.
GOLGA2	.	.	.	.	.	.	.	.	0.6099368	8.00E-09	.	.	.
GOLGA4	.	.	0.3882567	0.0006304	.	.	.	.	.	.	.	.	.
GOLGA7	.	.	.	.	.	.	.	.	0.4743719	1.96E-05	.	.	.
GOLIM4	.	.	0.5332095	1.00E-06	.	.	.	.	.	.	.	.	.
GOLM1	.	.	.	.	.	.	.	.	.	.	.	.	yes
GON4L	.	.	.	.	.	.	.	.	0.5539132	3.06E-07	.	.	.
GOPC	.	.	.	.	.	.	0.5323924	1.05E-06	.	.	.	.	.
GORASP2	.	.	.	.	.	.	.	.	0.6749131	4.28E-11	.	.	.
GOT1L1	0.5554206	2.80E-07	.	.	.	.	.	.	.	.	.	.	.
GOT2	.	.	.	.	.	.	.	.	0.5305246	1.16E-06	.	.	.
GPATCH2L	.	.	0.4541733	4.81E-05	.	.	.	.	.	.	.	.	.
GPC1	.	.	.	.	0.3534436	0.0020081	.	.	.	.	.	.	yes
GPC3	.	.	.	.	.	.	.	.	0.4911221	8.88E-06	.	.	yes
GPD2	.	.	0.4030293	0.0003705	.	.	.	.	.	.	.	.	.
GPI	.	.	.	.	.	.	.	.	.	.	.	.	yes
GPN3	.	.	.	.	.	.	.	.	0.5327669	1.03E-06	.	.	.
GPR101	.	.	.	.	.	.	.	.	0.4727998	2.10E-05	.	.	.
GPR107	.	.	0.3370341	0.003321	.	.	.	.	0.5258882	1.50E-06	.	.	.
GPR113	.	.	.	.	.	.	.	.	.	.	0.848151	1.49E-21	.
GPR114	.	.	.	.	.	.	.	.	.	.	0.5086062	3.72E-06	.

Gene	Atrial Expression	p-value Atrial	AV-Canal Expression	p-value AV-Canal	Branchial Arches Expression	p-value Branchial Arches	Heart Tube Expression	p-value Heart Tube	Outflow Tract Expression	p-value Outflow Tract	Ventricle Expression	p-value Ventricle	DMP Expression
GPR124	.	.	.	.	.	.	.	.	0.6300236	1.81E-09	.	.	yes
GPR125	.	.	0.520262	2.02E-06	.	.	.	.	0.4463418	6.72E-05	.	.	yes
GPR126	.	.	.	.	.	.	.	.	0.7295459	1.68E-13	.	.	.
GPR171	.	.	.	.	.	.	.	.	.	.	0.4771104	1.72E-05	.
GPR180	.	.	.	.	.	.	.	.	0.5609434	2.01E-07	.	.	.
GPR182	.	.	0.681591	2.32E-11	.	.	.	.	.	.	.	.	.
GPR22	.	.	.	.	0.4837304	1.26E-05	.	.	.	.	.	.	.
GPR4	.	.	.	.	.	.	.	.	0.6170911	4.77E-09	.	.	.
GPR45	.	.	.	.	.	.	.	.	.	.	0.5428122	5.84E-07	.
GPR56	.	.	.	.	.	.	.	.	.	.	0.6170947	4.77E-09	.
GPR97	.	.	0.3775639	0.0009122	.	.	.	.	.	.	.	.	.
GPSM1	.	.	.	.	.	.	.	.	0.6384285	9.39E-10	.	.	.
GPX1	.	.	.	.	.	.	.	.	.	.	.	.	yes
GPX3	.	.	.	.	.	.	.	.	0.3025292	0.0087969	.	.	yes
GPX4	.	.	.	.	.	.	.	.	0.6297233	1.85E-09	.	.	.
GPX7	.	.	0.3989072	0.0004308	.	.	.	.	0.4224836	0.0001771	.	.	.
GRAP2	.	.	0.4013427	0.0003942	.	.	.	.	.	.	.	.	.
GRASP	.	.	.	.	.	.	.	.	0.5382238	7.58E-07	.	.	.
GRB10	.	.	.	.	.	.	.	.	0.4608462	3.60E-05	.	.	yes
GRB14	.	.	.	.	.	.	0.4430555	7.71E-05	.	.	.	.	.
GREB1	.	.	.	.	.	.	.	.	0.5428846	5.82E-07	0.2986335	0.0097528	.
GRHL1	.	.	.	.	.	.	.	.	.	.	0.6748756	4.29E-11	.
GRHPR	.	.	0.3735667	0.001044	.	.	.	.	0.4748366	1.92E-05	.	.	.
GRID1	.	.	.	.	.	.	.	.	0.5433673	5.66E-07	.	.	.
GRIN2C	.	.	.	.	.	.	.	.	.	.	0.668363	7.69E-11	.
GRPEL1	.	.	.	.	.	.	.	.	0.498405	6.21E-06	.	.	.
GSK3B	.	.	0.3727962	0.0010713	.	.	.	.	.	.	.	.	yes
GSPT1	.	.	.	.	0.6325122	1.49E-09	.	.	0.3565626	0.0018194	.	.	yes
GSR	.	.	.	.	.	.	.	.	0.6083176	8.98E-09	.	.	.
GSTCD	.	.	.	.	.	.	.	.	.	.	0.6704771	6.38E-11	.
GSTO2	.	.	.	.	.	.	.	.	0.4595246	3.81E-05	.	.	.
GTF2A2	.	.	0.7113468	1.22E-12	.	.	.	.	.	.	.	.	.
GTF2E2	.	.	0.4896096	9.55E-06	.	.	.	.	.	.	.	.	.
GTF2H4	.	.	0.4643577	3.08E-05	.	.	.	.	.	.	.	.	.
GTF2I	.	.	.	.	0.3678097	0.0012643	.	.	.	.	.	.	yes
GTF2IRD1	.	.	.	.	.	.	0.5570546	2.54E-07	.	.	.	.	.
GTF3C1	.	.	.	.	.	.	.	.	.	.	.	.	yes
GTF3C2	.	.	.	.	.	.	.	.	.	.	.	.	yes
GTPBP10	.	.	.	.	.	.	.	.	0.542931	5.80E-07	.	.	.
GTPBP4	.	.	.	.	.	.	.	.	0.6434466	6.29E-10	.	.	.
GTPBP8	.	.	.	.	.	.	.	.	0.4579491	4.09E-05	.	.	.
GUCD1	.	.	.	.	.	.	.	.	0.4947342	7.44E-06	.	.	.
GXYLT1	.	.	0.4117664	0.0002674	.	.	.	.	.	.	.	.	.
GYG1	.	.	0.3854004	0.0006966	.	.	.	.	.	.	.	.	.
GYS1	.	.	0.503616	4.79E-06	.	.	.	.	0.4264378	0.0001516	.	.	.
H1F0	.	.	0.3770317	0.0009288	.	.	.	.	0.428345	0.0001405	.	.	yes
H1FX	.	.	.	.	0.4730985	2.07E-05	.	.	0.5219988	1.84E-06	.	.	.
H2AFX	.	.	.	.	.	.	.	.	.	.	.	.	yes
H2AFY	.	.	.	.	.	.	.	.	.	.	.	.	yes
H2AFY2	.	.	.	.	.	.	.	.	.	.	.	.	yes
H6PD	.	.	0.3937422	0.000519	.	.	.	.	.	.	.	.	.
HADHA	.	.	0.493705	7.83E-06	.	.	.	.	0.3390718	0.0031244	.	.	.
HADHB	.	.	.	.	.	.	.	.	0.5151554	2.65E-06	.	.	.
HAND1	.	.	.	.	.	.	0.4453913	7.00E-05	0.5926391	2.66E-08	.	.	.
HAND2	.	.	0.4507957	5.56E-05	.	.	.	.	0.6694526	6.99E-11	.	.	.
HAPLN1	.	.	0.7314722	1.35E-13	.	.	.	.	0.4001859	0.0004112	.	.	.
HARS	.	.	.	.	.	.	.	.	0.7816186	2.07E-16	.	.	.
HAS2	.	.	0.4327464	0.0001178	.	.	.	.	0.7443907	2.93E-14	.	.	.
HAUS2	.	.	.	.	.	.	.	.	0.5075168	3.93E-06	.	.	.
HAUS4	.	.	.	.	.	.	.	.	0.7729152	7.17E-16	.	.	.
HAUS8	.	.	0.4298325	0.0001324	.	.	.	.	0.3034161	0.0085911	.	.	.
HBEGF	.	.	.	.	.	.	0.6039365	1.22E-08	0.3173907	0.0058612	.	.	.
HBG1	.	.	0.5699662	1.16E-07	.	.	.	.	.	.	.	.	yes
HBG2	.	.	.	.	.	.	.	.	.	.	.	.	yes
HBS1L	.	.	.	.	.	.	.	.	0.4913169	8.79E-06	.	.	.
HBZ	.	.	0.4411498	8.35E-05	.	.	.	.	.	.	.	.	yes
HCFC1	.	.	0.4056916	0.0003358	.	.	.	.	.	.	.	.	yes
HCFC1R1	.	.	0.3733288	0.0010524	.	.	.	.	0.8105859	2.13E-18	.	.	.
HCN4	.	.	.	.	.	.	.	.	.	.	0.4493895	5.91E-05	.
HDAC10	.	.	0.5434424	5.64E-07	.	.	.	.	.	.	.	.	.
HDAC2	.	.	.	.	.	.	.	.	.	.	.	.	yes
HDAC3	.	.	.	.	0.5831153	4.99E-08	.	.	.	.	.	.	.
HDAC5	.	.	.	.	.	.	.	.	0.6881054	1.25E-11	.	.	.
HDAC6	.	.	0.3910157	0.0005719	.	.	.	.	.	.	.	.	.
HDAC8	.	.	.	.	.	.	.	.	.	.	0.6851892	1.65E-11	.

Gene	Atrial Expression	p-value Atrial	AV-Canal Expression	p-value AV-Canal	Branchial Arches Expression	p-value Branchial Arches	Heart Tube Expression	p-value Heart Tube	Outflow Tract Expression	p-value Outflow Tract	Ventricle Expression	p-value Ventricle	DMP Expression
HDGF	.	.	.	.	.	.	.	.	.	.	.	.	yes
HDLBP	.	.	.	.	.	.	.	.	.	.	.	.	yes
HEATR1	.	.	.	.	0.4716584	2.22E-05	.	.	.	.	.	.	.
HEATR2	.	.	0.3233892	0.0049467	.	.	.	.	0.5970636	1.97E-08	.	.	.
HEATR5A	.	.	0.5318414	1.08E-06	.	.	.	.	.	.	.	.	.
HEATR5B	.	.	0.4505226	5.63E-05	.	.	.	.	.	.	.	.	.
HECTD1	.	.	.	.	.	.	.	.	.	.	.	.	yes
HEG1	.	.	0.4497052	5.83E-05	.	.	.	.	.	.	.	.	.
HELT	.	.	.	.	.	.	.	.	0.4870532	1.08E-05	.	.	.
HERC6	.	.	0.4010821	0.000398	.	.	.	.	.	.	.	.	.
HERPUD2	.	.	.	.	.	.	.	.	0.5242738	1.63E-06	.	.	.
HES7	.	.	.	.	.	.	.	.	.	.	0.4410544	8.38E-05	.
HESX1	.	.	0.3867134	0.0006654	.	.	.	.	.	.	.	.	.
HEY2	.	.	0.4556895	4.51E-05	.	.	.	.	0.694317	6.88E-12	.	.	.
HGS	.	.	.	.	.	.	.	.	0.7464764	2.27E-14	.	.	.
HIBADH	.	.	0.4118756	0.0002663	.	.	.	.	.	.	.	.	.
HIF1A	.	.	0.3451725	0.0025963	.	.	.	.	0.5003944	5.63E-06	.	.	yes
HIF1AN	.	.	.	.	.	.	.	.	0.7179886	6.03E-13	.	.	.
HIGD2A	.	.	0.525262	1.55E-06	.	.	.	.	0.3955964	0.0004856	.	.	.
HINT3	0.5426443	5.90E-07	.	.	.	.	.	.	.	.	.	.	.
HIP1	.	.	.	.	.	.	.	.	0.3800356	0.0008384	0.610784	7.53E-09	yes
HIPK3	.	.	.	.	.	.	.	.	0.6996566	4.06E-12	.	.	.
HIST3H2A	.	.	0.3811159	0.0008079	.	.	.	.	.	.	.	.	.
HK1	.	.	.	.	.	.	.	.	0.6813577	2.37E-11	.	.	.
HK2	.	.	0.3157462	0.0061366	0.4303374	0.0001298	.	.	.	.	.	.	yes
HLA-DMA	.	.	0.3909807	0.0005726	.	.	.	.	.	.	.	.	.
HLA-DOB	.	.	.	.	.	.	.	.	.	.	0.4764741	1.78E-05	.
HLCS	.	.	.	.	.	.	.	.	.	.	0.4978879	6.37E-06	.
HLTF	.	.	.	.	.	.	.	.	0.6550622	2.42E-10	.	.	.
HM13	.	.	0.4200525	0.0001947	.	.	.	.	.	.	.	.	.
HMCES	.	.	.	.	.	.	.	.	0.4697349	2.42E-05	.	.	.
HMG20B	.	.	0.5822541	5.28E-08	.	.	.	.	.	.	.	.	.
HMGA2	.	.	.	.	.	.	.	.	.	.	.	.	yes
HMGCR	.	.	.	.	.	.	.	.	.	.	.	.	yes
HMGCS1	.	.	.	.	.	.	.	.	.	.	.	.	yes
HMGN2	.	.	.	.	.	.	0.4897123	9.50E-06	.	.	.	.	.
HMX3	.	.	.	.	.	.	.	.	0.5125291	3.04E-06	.	.	.
HN1L	.	.	0.3115481	0.0068921	.	.	.	.	0.5728836	9.62E-08	.	.	yes
HNRNPA0	.	.	.	.	0.3945681	0.0005039	.	.	.	.	.	.	yes
HNRNPA2B1	.	.	0.4751073	1.89E-05	.	.	.	.	.	.	.	.	yes
HNRNPAB	.	.	.	.	.	.	.	.	.	.	.	.	yes
HNRNPC	.	.	.	.	.	.	.	.	0.4629764	3.27E-05	.	.	yes
HNRNPD	.	.	.	.	0.3486032	0.0023358	.	.	.	.	.	.	yes
HNRNPF	.	.	.	.	.	.	.	.	0.6018911	1.41E-08	.	.	.
HNRNPH1	.	.	.	.	.	.	.	.	0.5744073	8.74E-08	.	.	yes
HNRNPK	.	.	.	.	0.3407688	0.0029686	.	.	.	.	.	.	yes
HNRNPL	.	.	.	.	0.4396155	8.90E-05	.	.	.	.	.	.	yes
HNRNPM	.	.	0.3326497	0.0037819	.	.	.	.	.	.	.	.	yes
HNRNPR	.	.	.	.	.	.	.	.	.	.	0.6319833	1.55E-09	.
HNRNPU	.	.	0.3004309	0.0093011	0.3509928	0.0021685	.	.	0.55748	2.48E-07	.	.	yes
HNRNPUL1	.	.	0.3452718	0.0025884	.	.	.	.	.	.	.	.	yes
HNRNPUL2	.	.	.	.	.	.	.	.	.	.	.	.	yes
HOMER2	.	.	0.3389572	0.0031352	.	.	.	.	0.487763	1.04E-05	.	.	.
HOOK2	.	.	.	.	.	.	.	.	0.4526132	5.15E-05	.	.	.
HOXA9	.	.	.	.	.	.	.	.	.	.	0.4732179	2.06E-05	.
HOXB1	.	.	0.3964101	0.0004716	.	.	.	.	.	.	.	.	.
HOXC4	.	.	.	.	.	.	.	.	.	.	0.6930019	7.82E-12	.
HP1BP3	.	.	.	.	.	.	.	.	.	.	.	.	yes
HPRT1	.	.	0.3520563	0.0020975	.	.	.	.	0.5088173	3.68E-06	.	.	.
HRC	.	.	0.3568101	0.0018052	.	.	.	.	0.6300615	1.80E-09	.	.	.
HS1BP3	.	.	0.5767918	7.51E-08	.	.	.	.	.	.	.	.	.
HS3ST1	.	.	0.3770902	0.000927	.	.	.	.	.	.	.	.	.
HSBP1L1	.	.	0.4019193	0.000386	.	.	.	.	.	.	.	.	.
HSD17B12	.	.	0.4617445	3.46E-05	.	.	.	.	.	.	.	.	.
HSD17B4	.	.	0.4921423	8.45E-06	.	.	.	.	0.3743934	0.0010154	.	.	.
HSDL2	.	.	0.44407	7.39E-05	.	.	.	.	.	.	.	.	.
HSF1	.	.	.	.	0.5330179	1.01E-06	.	.	.	.	.	.	.
HSP90AA1	.	.	.	.	.	.	.	.	.	.	.	.	yes
HSP90AB1	.	.	.	.	.	.	.	.	.	.	.	.	yes
HSP90B1	.	.	.	.	.	.	.	.	.	.	.	.	yes
HSPA14	.	.	.	.	.	.	.	.	0.6439533	6.04E-10	.	.	.
HSPA4	.	.	.	.	.	.	.	.	0.7036142	2.72E-12	.	.	yes
HSPA5	.	.	.	.	.	.	.	.	.	.	.	.	yes
HSPA8	.	.	.	.	0.3264	0.0045373	.	.	.	.	.	.	yes
HSPA9	.	.	0.3648854	0.0013915	.	.	.	.	0.5725754	9.81E-08	.	.	yes

Gene	Atrial Expression	p-value Atrial	AV-Canal Expression	p-value AV-Canal	Branchial Arches Expression	p-value Branchial Arches	Heart Tube Expression	p-value Heart Tube	Outflow Tract Expression	p-value Outflow Tract	Ventricle Expression	p-value Ventricle	DMP Expression
HSPB1	.	.	0.4356318	0.0001048	.	.	.	.	0.7506285	1.36E-14	.	.	.
HSPB2	.	.	0.4608969	3.59E-05	.	.	0.3142096	0.0064042	0.5151867	2.64E-06	.	.	.
HSPB3	.	.	0.6291881	1.93E-09	.	.	.	.	0.4848181	1.20E-05	.	.	.
HSPB7	.	.	0.3980698	0.0004441	.	.	.	.	0.7305683	1.49E-13	.	.	.
HSPB8	.	.	0.3789872	0.000869	.	.	.	.	0.3665431	0.0013181	.	.	.
HSPBAP1	.	.	.	.	.	.	.	.	0.49215	8.45E-06	.	.	.
HSPD1	.	.	.	.	0.4780419	1.65E-05	.	.	.	.	.	.	yes
HSPG2	.	.	.	.	.	.	.	.	0.6671154	8.59E-11	.	.	yes
HSPH1	.	.	.	.	0.7542787	8.58E-15	0.3578343	0.0017472	.	.	.	.	yes
HTRA1	.	.	0.4001439	0.0004119	.	.	.	.	.	.	.	.	.
HTT	.	.	.	.	.	.	.	.	0.8232466	2.23E-19	.	.	.
HUWE1	.	.	.	.	.	.	.	.	.	.	.	.	yes
HYOU1	.	.	.	.	.	.	.	.	.	.	.	.	yes
HYPK	.	.	.	.	.	.	.	.	0.7245309	2.95E-13	.	.	.
IARS	.	.	0.3230914	0.004989	.	.	.	.	.	.	.	.	yes
IBA57	.	.	.	.	.	.	.	.	0.4464525	6.69E-05	.	.	.
ID1	.	.	0.3768094	0.0009359	.	.	.	.	.	.	.	.	.
ID2	.	.	0.6113726	7.22E-09	.	.	.	.	0.4636231	3.18E-05	.	.	yes
ID3	.	.	.	.	.	.	.	.	.	.	.	.	yes
IDH2	.	.	.	.	.	.	.	.	.	.	.	.	yes
IDH3A	.	.	0.5433206	5.68E-07	.	.	.	.	0.3484859	0.0023443	.	.	.
IER3	.	.	0.4726939	2.11E-05	.	.	.	.	0.3346861	0.0035612	.	.	.
IER5	.	.	.	.	.	.	.	.	0.5188632	2.18E-06	.	.	.
IFIH1	.	.	.	.	.	.	0.58293	5.05E-08	.	.	.	.	.
IFNGR2	.	.	0.4407452	8.49E-05	.	.	.	.	0.4604629	3.66E-05	.	.	.
IFNK	0.450663	5.60E-05	.	.	.	.	.	.	.	.	.	.	.
IFT140	.	.	.	.	.	.	.	.	.	.	0.4988091	6.09E-06	.
IFT46	.	.	.	.	0.4770125	1.73E-05	.	.	.	.	.	.	.
IGDCC3	.	.	.	.	.	.	.	.	0.352782	0.0020503	.	.	yes
IGF1	.	.	0.4007552	0.0004028	.	.	.	.	.	.	.	.	.
IGF1R	.	.	.	.	.	.	.	.	.	.	.	.	yes
IGF2	.	.	.	.	.	.	.	.	0.805294	5.20E-18	.	.	yes
IGF2BP1	.	.	.	.	.	.	.	.	0.5335987	9.81E-07	.	.	yes
IGF2BP2	.	.	0.4303357	0.0001298	.	.	.	.	0.5599623	2.13E-07	.	.	yes
IGF2BP3	.	.	.	.	.	.	.	.	.	.	.	.	yes
IGF2R	.	.	0.4860119	1.13E-05	.	.	.	.	0.4668777	2.75E-05	.	.	yes
IGFBP4	.	.	.	.	.	.	.	.	0.3033678	0.0086022	.	.	yes
IGFBP5	.	.	0.5284947	1.30E-06	.	.	.	.	0.3265053	0.0045235	.	.	yes
IGSF21	.	.	.	.	.	.	.	.	0.6142699	5.86E-09	.	.	.
IKBIP	.	.	.	.	.	.	.	.	0.4843556	1.23E-05	.	.	.
IKBKAP	.	.	.	.	0.5119456	3.13E-06	.	.	.	.	.	.	yes
IKBKE	.	.	.	.	.	.	.	.	0.6461204	5.07E-10	.	.	.
IKZF3	.	.	0.3755907	0.0009753	.	.	.	.	.	.	.	.	.
IL13RA1	.	.	0.5960713	2.10E-08	.	.	.	.	.	.	.	.	.
IL27	.	.	.	.	.	.	.	.	0.6352457	1.21E-09	.	.	.
ILF2	.	.	.	.	.	.	.	.	0.6166187	4.93E-09	.	.	yes
ILF3	.	.	.	.	.	.	.	.	0.4148296	0.000238	.	.	yes
ILK	.	.	.	.	.	.	.	.	0.5369815	8.13E-07	.	.	.
IMMT	.	.	.	.	.	.	.	.	0.6808055	2.49E-11	.	.	yes
IMP3	.	.	0.3655023	0.0013638	.	.	.	.	0.5326558	1.03E-06	.	.	.
IMPA2	.	.	0.4427513	7.81E-05	.	.	.	.	0.400461	0.0004071	.	.	.
IMPACT	.	.	.	.	0.5147048	2.71E-06	.	.	0.3481048	0.0023721	.	.	.
IMPAD1	.	.	0.4036189	0.0003626	.	.	.	.	0.3951793	0.0004929	.	.	.
IMPDH1	.	.	.	.	.	.	.	.	0.7899708	5.96E-17	.	.	.
INCENP	.	.	.	.	.	.	.	.	.	.	.	.	yes
INF2	.	.	.	.	.	.	.	.	0.5659921	1.48E-07	.	.	.
ING2	.	.	.	.	0.3597849	0.0016414	.	.	0.4737905	2.01E-05	.	.	.
INHBB	.	.	0.375008	0.0009946	.	.	.	.	.	.	.	.	.
INIP	.	.	0.4719219	2.19E-05	.	.	.	.	0.4599088	3.75E-05	.	.	.
INO80	.	.	0.4745767	1.94E-05	.	.	.	.	.	.	.	.	.
INO80E	.	.	.	.	.	.	.	.	0.437049	9.89E-05	.	.	.
INPP5D	.	.	.	.	.	.	.	.	.	.	0.5901487	3.14E-08	.
INPP5E	.	.	.	.	.	.	.	.	0.4933547	7.96E-06	.	.	.
INPPL1	.	.	0.3345564	0.0035749	.	.	.	.	.	.	.	.	yes
INTS1	.	.	.	.	0.4483031	6.19E-05	.	.	.	.	.	.	.
INTS10	.	.	0.4851416	1.18E-05	.	.	.	.	.	.	.	.	.
INTS3	.	.	.	.	.	.	.	.	0.6239835	2.86E-09	.	.	yes
INTS6	.	.	0.4108799	0.0002765	.	.	.	.	.	.	.	.	.
INTS8	.	.	.	.	.	.	.	.	.	.	0.4481666	6.22E-05	.
INVS	.	.	.	.	.	.	0.4582728	4.03E-05	.	.	.	.	.
IPO11	.	.	.	.	.	.	.	.	0.6001246	1.59E-08	.	.	.
IPO4	.	.	0.4284754	0.0001398	.	.	.	.	.	.	.	.	.
IPO5	.	.	.	.	.	.	.	.	.	.	.	.	yes
IPO7	.	.	.	.	.	.	.	.	.	.	.	.	yes
IPO9	.	.	.	.	.	.	.	.	.	.	.	.	yes

Gene	Atrial Expression	p-value Atrial	AV-Canal Expression	p-value AV-Canal	Branchial Arches Expression	p-value Branchial Arches	Heart Tube Expression	p-value Heart Tube	Outflow Tract Expression	p-value Outflow Tract	Ventricle Expression	p-value Ventricle	DMP Expression
IQCG	.	.	.	.	.	.	.	.	.	.	0.4689983	2.50E-05	.
IQCH	.	.	.	.	.	.	0.5382239	7.58E-07	.	.	0.3445969	0.0026425	.
IQCJ-SCHIP1	.	.	.	.	.	.	.	.	0.5796854	6.24E-08	.	.	.
IQUB	.	.	.	.	.	.	.	.	.	.	0.4728149	2.10E-05	.
IRAK2	.	.	.	.	.	.	.	.	0.5493932	4.00E-07	.	.	.
IREB2	.	.	.	.	.	.	.	.	0.3254988	0.0046566	.	.	yes
IRF3	.	.	0.4130851	0.0002544	.	.	.	.	0.3879188	0.0006379	.	.	.
IRF5	.	.	.	.	.	.	.	.	0.6396532	8.53E-10	.	.	.
IRS1	.	.	0.3821695	0.0007792	.	.	.	.	0.3495746	0.0022664	.	.	.
IRS2	.	.	.	.	0.4737699	2.01E-05	.	.	.	.	.	.	.
IRX4	.	.	0.5713081	1.06E-07	.	.	.	.	.	.	.	.	.
ISCU	.	.	0.3812728	0.0008036	.	.	.	.	.	.	.	.	.
ISG20L2	.	.	0.3853759	0.0006972	.	.	.	.	0.4071612	0.0003179	.	.	.
ISM1	.	.	.	.	.	.	.	.	0.6674628	8.33E-11	.	.	.
ISY1-RAB43	.	.	.	.	.	.	.	.	0.444603	7.23E-05	.	.	.
ISYNA1	.	.	.	.	.	.	.	.	0.3950427	0.0004954	.	.	yes
ITCH	.	.	0.39077	0.0005769	.	.	.	.	.	.	.	.	.
ITGA11	.	.	.	.	.	.	.	.	.	.	0.6214046	3.47E-09	.
ITGA2B	.	.	0.5260117	1.49E-06	.	.	.	.	.	.	.	.	.
ITGA3	.	.	.	.	.	.	.	.	0.4392412	9.04E-05	.	.	.
ITGA6	.	.	0.4035671	0.0003633	.	.	.	.	0.3452741	0.0025882	.	.	.
ITGAD	.	.	.	.	.	.	.	.	0.8122276	1.61E-18	.	.	.
ITGB1	.	.	0.4341888	0.0001111	.	.	.	.	0.4317704	0.0001225	.	.	yes
ITGB3	.	.	0.3025212	0.0087988	.	.	.	.	0.4381047	9.47E-05	.	.	.
ITGB3BP	.	.	.	.	.	.	.	.	0.4732549	2.06E-05	.	.	.
ITGB5	.	.	0.4488204	6.05E-05	.	.	.	.	0.382709	0.0007648	.	.	.
ITM2C	.	.	0.4085111	0.0003023	.	.	.	.	0.4175919	0.0002141	.	.	.
ITSN1	.	.	.	.	.	.	.	.	.	.	.	.	yes
IVNS1ABP	.	.	.	.	.	.	.	.	.	.	.	.	yes
JADE3	.	.	.	.	.	.	.	.	0.5276414	1.36E-06	.	.	.
JAG1	.	.	0.3113349	0.0069325	.	.	.	.	0.7632507	2.67E-15	.	.	.
JAGN1	.	.	.	.	.	.	.	.	0.732772	1.16E-13	.	.	.
JAKMIP1	.	.	0.5569394	2.56E-07	.	.	.	.	.	.	.	.	.
JMJD4	.	.	.	.	.	.	.	.	0.5852771	4.33E-08	.	.	.
JMJD6	.	.	.	.	0.4405521	8.56E-05	.	.	.	.	.	.	.
JMJD8	.	.	.	.	.	.	.	.	0.788689	7.24E-17	.	.	.
JOSD2	.	.	.	.	.	.	.	.	0.6395359	8.61E-10	.	.	.
JPH2	.	.	.	.	.	.	.	.	0.8689777	1.08E-23	.	.	.
JTB	.	.	.	.	.	.	.	.	0.5174878	2.34E-06	.	.	.
JUN	.	.	0.5110623	3.28E-06	.	.	.	.	0.4302989	0.00013	.	.	.
JUND	.	.	.	.	.	.	.	.	0.6197439	3.92E-09	.	.	.
KARS	.	.	0.3506669	0.0021906	.	.	.	.	.	.	.	.	yes
KAT5	.	.	.	.	.	.	.	.	0.8624565	5.51E-23	.	.	.
KAT6A	.	.	.	.	.	.	.	.	0.5385395	7.45E-07	.	.	.
KATNA1	.	.	.	.	0.5099779	3.46E-06	.	.	.	.	.	.	.
KBTBD11	.	.	.	.	.	.	.	.	0.515878	2.55E-06	.	.	.
KBTBD8	.	.	.	.	0.4022149	0.0003818	.	.	.	.	0.5566557	2.60E-07	.
KCNA2	.	.	.	.	.	.	.	.	0.4837555	1.26E-05	.	.	.
KCNA4	.	.	.	.	.	.	.	.	.	.	0.7001447	3.86E-12	.
KCND3	.	.	.	.	.	.	.	.	.	.	0.4495511	5.87E-05	.
KCNE1	.	.	0.8058011	4.78E-18	.	.	.	.	.	.	.	.	.
KCNG1	.	.	0.4713957	2.24E-05	.	.	.	.	.	.	.	.	.
KCNG2	.	.	.	.	.	.	.	.	0.6151099	5.51E-09	.	.	.
KCNIP1	.	.	.	.	.	.	.	.	0.484901	1.20E-05	.	.	.
KCNJ11	.	.	.	.	.	.	.	.	0.5904662	3.07E-08	.	.	.
KCNJ13	.	.	0.3874411	0.0006487	.	.	.	.	.	.	.	.	.
KCNJ3	.	.	0.512244	3.08E-06	.	.	.	.	.	.	.	.	.
KCNJ4	.	.	0.7567652	6.24E-15	.	.	.	.	.	.	.	.	.
KCNJ5	.	.	0.3898198	0.0005966	.	.	.	.	.	.	.	.	.
KCNMA1	.	.	.	.	.	.	.	.	.	.	0.5033277	4.86E-06	.
KCNMB1	.	.	.	.	.	.	.	.	0.6321545	1.53E-09	.	.	.
KCNN2	.	.	.	.	.	.	.	.	0.4948826	7.39E-06	.	.	.
KCNQ1	.	.	.	.	.	.	.	.	.	.	0.5293842	1.24E-06	.
KCTD15	.	.	.	.	.	.	.	.	0.5034928	4.82E-06	.	.	.
KCTD5	.	.	.	.	.	.	.	.	0.5568773	2.57E-07	.	.	.
KCTD7	.	.	.	.	.	.	.	.	0.6073757	9.60E-09	.	.	.
KDELR2	.	.	.	.	.	.	.	.	0.4795395	1.54E-05	.	.	.
KDM1A	.	.	.	.	.	.	.	.	0.387892	0.0006385	.	.	yes
KDM2A	.	.	0.4242375	0.0001653	.	.	.	.	.	.	.	.	yes
KDM3A	.	.	.	.	.	.	.	.	.	.	.	.	yes
KDM4D	0.6954644	6.15E-12	.	.	.	.	.	.	.	.	.	.	.
KDM5B	.	.	.	.	.	.	.	.	.	.	0.5030351	4.93E-06	yes
KDM5C	.	.	.	.	.	.	.	.	0.4165586	0.0002228	.	.	yes
KDM6B	.	.	0.3656891	0.0013555	.	.	.	.	.	.	.	.	yes
KHDRBS1	.	.	.	.	.	.	.	.	.	.	.	.	yes

Gene	Atrial Expression	p-value Atrial	AV-Canal Expression	p-value AV-Canal	Branchial Arches Expression	p-value Branchial Arches	Heart Tube Expression	p-value Heart Tube	Outflow Tract Expression	p-value Outflow Tract	Ventricle Expression	p-value Ventricle	DMP Expression
KHSRP	.	.	.	.	.	.	.	.	0.5682488	1.29E-07	.	.	yes
KIAA0100	.	.	.	.	.	.	.	.	.	.	.	.	yes
KIAA0141	.	.	0.3824598	0.0007714	.	.	.	.	0.6180381	4.45E-09	.	.	.
KIAA0319	.	.	.	.	.	.	.	.	.	.	0.6034247	1.27E-08	.
KIAA0513	.	.	.	.	.	.	.	.	0.6360732	1.13E-09	.	.	.
KIAA0556	.	.	.	.	0.4797149	1.53E-05	.	.	.	.	.	.	.
KIAA0895L	.	.	.	.	.	.	.	.	.	.	0.4821237	1.36E-05	.
KIAA1191	.	.	0.3834787	0.0007447	.	.	.	.	.	.	.	.	.
KIAA1211	.	.	.	.	.	.	.	.	0.6937029	7.31E-12	.	.	.
KIAA1468	.	.	.	.	.	.	.	.	.	.	0.5574346	2.49E-07	.
KIAA1524	.	.	.	.	.	.	.	.	0.6711495	6.01E-11	.	.	.
KIAA1549	.	.	.	.	0.6410491	7.63E-10	.	.	.	.	.	.	.
KIAA1614	.	.	0.4653348	2.95E-05	.	.	.	.	.	.	.	.	.
KIAA1731	.	.	.	.	.	.	.	.	0.6938974	7.17E-12	.	.	.
KIAA1841	.	.	.	.	.	.	.	.	.	.	0.5739428	9.00E-08	.
KIAA2013	.	.	.	.	.	.	.	.	0.4795165	1.54E-05	.	.	.
KIF11	.	.	.	.	.	.	.	.	.	.	.	.	yes
KIF13A	.	.	.	.	.	.	.	.	0.6883333	1.23E-11	.	.	.
KIF14	.	.	.	.	0.4514785	5.40E-05	.	.	.	.	.	.	.
KIF20A	0.4290922	0.0001364	.	.	.	.	.	.	.	.	.	.	yes
KIF22	.	.	0.4109142	0.0002762	.	.	.	.	0.357072	0.0017902	.	.	.
KIF5B	.	.	0.3731801	0.0010576	.	.	.	.	.	.	.	.	yes
KIN	.	.	.	.	.	.	.	.	0.7347973	9.17E-14	.	.	.
KIRREL	.	.	.	.	.	.	.	.	.	.	.	.	yes
KISS1R	.	.	.	.	.	.	.	.	.	.	0.5414159	6.33E-07	.
KITLG	.	.	0.4211008	0.0001869	.	.	.	.	.	.	.	.	.
KLC2	.	.	0.3109123	0.0070134	.	.	.	.	0.5146999	2.71E-06	.	.	.
KLC3	.	.	.	.	.	.	.	.	0.6153965	5.39E-09	.	.	.
KLF1	.	.	0.4004278	0.0004076	.	.	.	.	.	.	.	.	.
KLF16	.	.	.	.	.	.	.	.	0.7146302	8.65E-13	.	.	.
KLF2	.	.	0.5537625	3.09E-07	.	.	.	.	0.6509423	3.41E-10	.	.	.
KLF6	.	.	0.4514181	5.42E-05	.	.	.	.	.	.	.	.	.
KLHDC10	.	.	.	.	.	.	.	.	.	.	.	.	yes
KLHDC4	.	.	.	.	.	.	.	.	.	.	0.683238	1.99E-11	.
KLHDC7A	.	.	.	.	.	.	.	.	0.6156927	5.28E-09	.	.	.
KLHL12	.	.	.	.	.	.	.	.	.	.	0.5368038	8.21E-07	.
KLHL20	.	.	.	.	.	.	.	.	.	.	0.5339526	9.62E-07	.
KLHL30	.	.	0.4525062	5.17E-05	.	.	.	.	0.6412903	7.48E-10	.	.	.
KLHL31	.	.	0.3455637	0.0025653	.	.	.	.	0.5175504	2.34E-06	.	.	.
KLHL4	.	.	0.4013834	0.0003936	.	.	.	.	.	.	.	.	.
KLHL6	.	.	0.3439277	0.0026971	.	.	.	.	0.7751261	5.26E-16	.	.	.
KLHL7	.	.	.	.	0.4492661	5.94E-05	.	.	.	.	.	.	.
KLHL9	.	.	0.41531	0.0002337	.	.	.	.	0.4835718	1.27E-05	.	.	.
KLK11	.	.	.	.	.	.	.	.	.	.	0.6048036	1.15E-08	.
KLK3	.	.	.	.	.	.	.	.	.	.	0.4473852	6.43E-05	.
KLRB1	.	.	.	.	.	.	.	.	.	.	0.5916114	2.85E-08	.
KNOP1	.	.	0.3873236	0.0006514	.	.	.	.	0.3776571	0.0009093	.	.	.
KPNA1	.	.	0.3776271	0.0009102	.	.	.	.	.	.	.	.	.
KPNA3	.	.	0.5282236	1.32E-06	.	.	.	.	.	.	.	.	.
KPNB1	.	.	0.3510774	0.0021627	.	.	.	.	0.6168894	4.84E-09	.	.	yes
KRAS	.	.	.	.	.	.	.	.	0.4391821	9.06E-05	.	.	.
KRBA1	.	.	.	.	.	.	.	.	0.5314738	1.10E-06	.	.	.
KRTCAP2	0.4374623	9.72E-05	.	.	.	.	.	.	.	.	.	.	.
KSR2	.	.	.	.	.	.	.	.	.	.	0.6877077	1.30E-11	.
KTN1	.	.	.	.	.	.	.	.	.	.	0.6652624	1.01E-10	yes
LACTB	.	.	0.4645537	3.05E-05	.	.	.	.	.	.	.	.	.
LAG3	.	.	.	.	.	.	.	.	0.4632549	3.23E-05	.	.	.
LAMA4	.	.	0.4847083	1.21E-05	.	.	.	.	.	.	.	.	.
LAMA5	.	.	.	.	.	.	.	.	.	.	.	.	yes
LAMB1	.	.	.	.	.	.	.	.	0.4844975	1.22E-05	.	.	yes
LAMC1	.	.	0.4124409	0.0002607	.	.	.	.	0.5722827	9.99E-08	.	.	yes
LAMP1	.	.	0.389677	0.0005996	.	.	.	.	.	.	.	.	yes
LAMTOR4	.	.	0.5021263	5.16E-06	.	.	.	.	0.530579	1.16E-06	.	.	.
LAP3	.	.	.	.	.	.	.	.	0.5291704	1.25E-06	.	.	.
LAPTM4A	.	.	.	.	.	.	.	.	.	.	.	.	yes
LAPTM4B	.	.	0.3022115	0.0088717	.	.	.	.	0.5279953	1.34E-06	.	.	.
LARP1	.	.	.	.	.	.	.	.	0.392004	0.0005522	.	.	yes
LARP4B	.	.	0.4850163	1.19E-05	.	.	.	.	.	.	.	.	.
LARP7	.	.	0.5162653	2.50E-06	.	.	.	.	0.3945236	0.0005047	.	.	.
LARS	.	.	.	.	.	.	.	.	0.4553249	4.58E-05	.	.	.
LARS2	.	.	.	.	.	.	.	.	.	.	.	.	yes
LAT2	.	.	.	.	.	.	.	.	0.5369962	8.12E-07	.	.	.
LBH	.	.	0.4793397	1.55E-05	.	.	.	.	0.5547716	2.91E-07	.	.	yes
LBR	.	.	.	.	.	.	.	.	0.5256509	1.52E-06	.	.	.
LCA5	.	.	0.639183	8.85E-10	.	.	.	.	.	.	.	.	.

Gene	Atrial Expression	p-value Atrial	AV-Canal Expression	p-value AV-Canal	Branchial Arches Expression	p-value Branchial Arches	Heart Tube Expression	p-value Heart Tube	Outflow Tract Expression	p-value Outflow Tract	Ventricle Expression	p-value Ventricle	DMP Expression
LCN10	.	.	.	.	.	.	.	.	0.6660223	9.45E-11	.	.	.
LDB1	.	.	.	.	.	.	.	.	.	.	.	.	yes
LDHA	.	.	0.3002787	0.0093386	.	.	0.353673	0.0019936	.	.	.	.	yes
LDLRAD2	.	.	0.3845603	0.0007173	.	.	.	.	.	.	.	.	.
LDLRAD3	.	.	0.3887683	0.0006191	.	.	.	.	.	.	.	.	.
LEF1	.	.	0.4722337	2.16E-05	.	.	.	.	.	.	.	.	.
LENEP	.	.	0.4274588	0.0001455	.	.	.	.	.	.	.	.	.
LENG1	.	.	.	.	.	.	.	.	0.5393319	7.12E-07	.	.	.
LENG8	.	.	0.3310392	0.003965	.	.	.	.	.	.	.	.	yes
LEPREL4	.	.	0.3366993	0.0033544	.	.	.	.	0.6319469	1.56E-09	.	.	.
LHFP	.	.	0.4294789	0.0001343	.	.	.	.	.	.	.	.	.
LHFPL1	.	.	.	.	.	.	.	.	0.85413	3.93E-22	.	.	.
LHX2	.	.	.	.	.	.	.	.	.	.	0.5475752	4.44E-07	.
LHX6	.	.	.	.	0.5442922	5.37E-07	.	.	.	.	.	.	.
LIFR	.	.	.	.	0.5134199	2.90E-06	.	.	.	.	.	.	.
LIG1	.	.	.	.	.	.	.	.	0.3545472	0.0019394	.	.	yes
LILRA5	.	.	.	.	.	.	.	.	0.6650255	1.03E-10	.	.	.
LIMA1	.	.	.	.	0.5180593	2.27E-06	.	.	.	.	.	.	.
LIMD2	.	.	0.4052986	0.0003407	.	.	.	.	0.3341334	0.00362	.	.	.
LIMK1	.	.	.	.	.	.	.	.	0.5331549	1.01E-06	.	.	.
LIMS2	.	.	.	.	.	.	.	.	0.5859747	4.14E-08	.	.	.
LIN28A	.	.	.	.	0.3602798	0.0016155	.	.	.	.	.	.	yes
LIN28B	.	.	.	.	0.533763	9.72E-07	.	.	.	.	.	.	.
LINGO1	.	.	.	.	.	.	.	.	.	.	.	.	yes
LIPI	.	.	.	.	.	.	0.4375514	9.69E-05	.	.	0.4499931	5.76E-05	.
LKAAEAR1	.	.	.	.	.	.	.	.	.	.	0.511106	3.27E-06	.
LLGL1	.	.	.	.	.	.	.	.	0.6146696	5.69E-09	.	.	yes
LMAN1	.	.	0.4807229	1.46E-05	.	.	.	.	.	.	.	.	.
LMAN2	.	.	0.3705859	0.0011533	.	.	.	.	0.59664	2.02E-08	.	.	yes
LMBRD2	.	.	0.4265713	0.0001508	.	.	.	.	.	.	.	.	.
LMNB1	.	.	.	.	0.5981191	1.83E-08	.	.	.	.	.	.	yes
LMOD1	.	.	.	.	.	.	.	.	0.436871	9.96E-05	.	.	.
LMOD2	.	.	0.6147025	5.67E-09	.	.	.	.	.	.	.	.	.
LNPEP	.	.	.	.	.	.	.	.	0.5639142	1.68E-07	.	.	.
LNX1	.	.	0.3952026	0.0004925	.	.	.	.	.	.	.	.	.
LOC645177	.	.	.	.	.	.	.	.	.	.	0.6098685	8.04E-09	.
LONP1	.	.	.	.	0.4048037	0.000347	.	.	.	.	.	.	yes
LOXL2	.	.	0.3859811	0.0006827	.	.	.	.	0.569404	1.20E-07	.	.	yes
LPHN1	.	.	0.3221836	0.0051197	.	.	.	.	0.5014602	5.34E-06	.	.	yes
LPHN2	.	.	.	.	.	.	.	.	.	.	.	.	yes
LPL	.	.	0.5424394	5.97E-07	.	.	.	.	.	.	.	.	.
LPXN	.	.	.	.	.	.	.	.	.	.	0.679953	2.70E-11	.
LRBA	.	.	.	.	.	.	.	.	.	.	0.5967384	2.01E-08	.
LRCH2	.	.	0.5188392	2.18E-06	.	.	.	.	.	.	.	.	.
LRIG2	.	.	0.4282581	0.000141	.	.	.	.	.	.	.	.	.
LRP1	.	.	.	.	.	.	.	.	0.3366005	0.0033643	.	.	yes
LRP6	.	.	.	.	.	.	.	.	.	.	.	.	yes
LRR1	.	.	.	.	.	.	.	.	0.5639371	1.68E-07	.	.	.
LRRC10	.	.	0.3631972	0.0014702	.	.	.	.	0.6323042	1.52E-09	.	.	.
LRRC14B	.	.	0.5058401	4.28E-06	.	.	.	.	.	.	.	.	.
LRRC20	.	.	.	.	.	.	.	.	0.5750431	8.39E-08	.	.	.
LRRC23	.	.	.	.	.	.	.	.	.	.	0.7002456	3.82E-12	.
LRRC32	.	.	.	.	.	.	.	.	.	.	0.529224	1.25E-06	.
LRRC39	.	.	.	.	.	.	.	.	0.5522584	3.38E-07	.	.	.
LRRC41	.	.	.	.	.	.	.	.	0.737393	6.77E-14	.	.	.
LRRC42	.	.	.	.	.	.	.	.	0.4511894	5.47E-05	.	.	.
LRRC47	.	.	.	.	.	.	.	.	0.4690531	2.49E-05	.	.	.
LRRC52	.	.	.	.	.	.	.	.	.	.	0.5782954	6.82E-08	.
LRRC58	.	.	.	.	.	.	.	.	0.4643483	3.08E-05	.	.	yes
LRRC59	.	.	.	.	.	.	.	.	.	.	.	.	yes
LRRC61	.	.	.	.	.	.	.	.	0.4367837	9.99E-05	.	.	.
LRRC69	.	.	.	.	.	.	.	.	.	.	0.4605274	3.65E-05	.
LRRC7	.	.	.	.	.	.	.	.	.	.	0.6053005	1.11E-08	.
LRRC8E	.	.	.	.	.	.	.	.	0.6822278	2.18E-11	.	.	.
LRRK1	.	.	.	.	.	.	.	.	0.574786	8.53E-08	.	.	.
LRRN4CL	.	.	.	.	.	.	.	.	0.6719218	5.60E-11	.	.	.
LRTM1	0.4948761	7.39E-06	.	.	.	.	.	.	.	.	.	.	.
LRWD1	.	.	.	.	0.60137	1.46E-08	.	.	0.3038142	0.0085001	.	.	.
LSM11	.	.	0.4216317	0.0001831	.	.	.	.	0.3055039	0.0081232	.	.	.
LSM14A	.	.	.	.	.	.	.	.	0.4870078	1.08E-05	0.3612094	0.0015679	yes
LSM14B	.	.	.	.	.	.	.	.	0.31482	0.0062967	.	.	yes
LSM5	.	.	.	.	0.4611406	3.55E-05	.	.	.	.	.	.	.
LSMEM1	0.4484997	6.14E-05	0.3235114	0.0049295	.	.	.	.	.	.	.	.	.
LTA4H	.	.	.	.	.	.	0.5290771	1.26E-06	.	.	.	.	.
LUC7L	.	.	0.3627703	0.0014907	.	.	.	.	.	.	.	.	yes

Gene	Atrial Expression	p-value Atrial	AV-Canal Expression	p-value AV-Canal	Branchial Arches Expression	p-value Branchial Arches	Heart Tube Expression	p-value Heart Tube	Outflow Tract Expression	p-value Outflow Tract	Ventricle Expression	p-value Ventricle	DMP Expression
LUC7L2	.	.	.	.	.	.	.	.	.	.	.	.	yes
LUC7L3	.	.	.	.	.	.	.	.	.	.	.	.	yes
LUZP1	.	.	.	.	.	.	.	.	.	.	.	.	yes
LY6G6F	.	.	.	.	.	.	.	.	.	.	0.6132352	6.31E-09	.
LY75	.	.	.	.	.	.	.	.	0.4475649	6.38E-05	.	.	.
LYPD6	.	.	0.4398119	8.83E-05	.	.	.	.	.	.	.	.	.
LYPLAL1	.	.	0.3824115	0.0007727	.	.	.	.	.	.	.	.	.
LYRM2	.	.	0.3801538	0.0008351	.	.	.	.	0.3116986	0.0068637	.	.	.
LZTS2	.	.	0.4545856	4.73E-05	.	.	.	.	.	.	.	.	.
M1AP	.	.	.	.	.	.	.	.	.	.	0.5987854	1.75E-08	.
M6PR	.	.	.	.	.	.	.	.	0.470903	2.29E-05	.	.	.
MAATS1	.	.	0.4229417	0.0001739	.	.	.	.	.	.	.	.	.
MACROD2	.	.	.	.	.	.	.	.	0.5662762	1.45E-07	.	.	.
MAD2L1	.	.	.	.	.	.	.	.	0.4643732	3.08E-05	.	.	.
MAEA	.	.	.	.	.	.	.	.	.	.	.	.	yes
MAFG	.	.	0.4003285	0.0004091	.	.	.	.	0.3344306	0.0035883	.	.	.
MAGEA10	.	.	.	.	.	.	.	.	.	.	0.6049707	1.14E-08	.
MAGED1	.	.	.	.	.	.	.	.	.	.	.	.	yes
MAGED2	.	.	.	.	.	.	.	.	0.7272419	2.18E-13	.	.	yes
MAGEE2	.	.	.	.	.	.	.	.	0.5052589	4.41E-06	.	.	.
MAGI3	.	.	0.5005035	5.60E-06	.	.	.	.	.	.	.	.	.
MAGT1	.	.	0.4435621	7.55E-05	.	.	.	.	.	.	.	.	.
MAMSTR	.	.	.	.	.	.	.	.	0.6996555	4.06E-12	.	.	.
MAN1A1	.	.	0.5198157	2.07E-06	.	.	.	.	.	.	.	.	.
MAN1C1	.	.	0.6775311	3.37E-11	.	.	.	.	.	.	.	.	.
MAN2A2	.	.	.	.	.	.	.	.	0.448109	6.24E-05	.	.	.
MANBA	.	.	.	.	.	.	.	.	.	.	0.7119348	1.15E-12	.
MANEA	.	.	.	.	.	.	.	.	0.6095861	8.20E-09	.	.	.
MAP1A	.	.	.	.	.	.	.	.	.	.	0.5534424	3.15E-07	.
MAP1B	.	.	.	.	.	.	.	.	.	.	.	.	yes
MAP2K6	.	.	.	.	0.3835188	0.0007437	.	.	0.603549	1.26E-08	.	.	.
MAP3K7	.	.	0.4198792	0.000196	.	.	.	.	.	.	.	.	.
MAP4	.	.	.	.	.	.	.	.	.	.	.	.	yes
MAP4K5	.	.	.	.	.	.	.	.	0.5684691	1.27E-07	.	.	.
MAP7D1	.	.	0.5479946	4.34E-07	.	.	.	.	.	.	.	.	yes
MAPK1	.	.	.	.	.	.	.	.	.	.	.	.	yes
MAPK14	.	.	0.4026618	0.0003756	.	.	.	.	0.378568	0.0008816	.	.	.
MAPK1IP1L	.	.	0.5130545	2.95E-06	.	.	.	.	.	.	.	.	yes
MAPK6	.	.	.	.	0.6171621	4.74E-09	.	.	.	.	.	.	yes
MAPK7	.	.	.	.	.	.	.	.	.	.	0.4440882	7.39E-05	.
MAPK8IP3	.	.	.	.	.	.	.	.	0.5786558	6.66E-08	.	.	.
MAPK9	.	.	0.4242661	0.0001651	.	.	.	.	.	.	.	.	.
MAPKAP1	.	.	0.3780992	0.0008958	.	.	.	.	.	.	.	.	.
MAPKAPK2	.	.	0.3929348	0.0005342	.	.	.	.	0.4258294	0.0001553	.	.	yes
MAPKAPK5	.	.	0.3973247	0.0004563	.	.	.	.	0.4387563	9.22E-05	.	.	.
MAPRE1	.	.	0.4783495	1.63E-05	.	.	.	.	0.5776003	7.13E-08	.	.	yes
MARCH11	.	.	.	.	.	.	.	.	.	.	0.7325569	1.19E-13	.
MARCH6	.	.	0.5973368	1.93E-08	.	.	.	.	.	.	.	.	.
MARCH7	.	.	.	.	.	.	.	.	0.6289246	1.97E-09	.	.	.
MARCH8	.	.	0.4650428	2.99E-05	.	.	.	.	.	.	.	.	.
MARCKS	.	.	0.3622359	0.0015167	.	.	.	.	.	.	.	.	yes
MARCKSL1	.	.	0.3816598	0.000793	.	.	.	.	0.5261765	1.47E-06	.	.	yes
MARK4	.	.	0.40049	0.0004067	.	.	.	.	0.2977522	0.0099812	.	.	.
MAT2A	.	.	.	.	.	.	.	.	0.3536973	0.0019921	.	.	yes
MATN3	.	.	0.5232255	1.73E-06	.	.	.	.	.	.	.	.	.
MATR3	.	.	.	.	.	.	.	.	.	.	.	.	yes
MAX	.	.	.	.	0.5464264	4.75E-07	.	.	.	.	.	.	.
MAZ	.	.	0.5967194	2.01E-08	.	.	.	.	.	.	.	.	yes
MB	.	.	.	.	.	.	.	.	.	.	0.6631057	1.22E-10	.
MB21D2	.	.	.	.	.	.	.	.	0.7198656	4.92E-13	.	.	.
MBD2	.	.	0.4128004	0.0002572	.	.	.	.	0.5514884	3.54E-07	.	.	.
MBD3	.	.	.	.	.	.	.	.	0.5891363	3.36E-08	.	.	yes
MBTPS1	.	.	.	.	.	.	.	.	.	.	.	.	yes
MCAT	.	.	.	.	0.4371153	9.86E-05	.	.	.	.	.	.	.
MCL1	.	.	0.2979124	0.0099394	.	.	.	.	0.4000472	0.0004133	.	.	yes
MCM2	.	.	0.3703979	0.0011605	0.3292624	0.0041761	.	.	0.3571934	0.0017833	.	.	yes
MCM3	.	.	0.3259595	0.0045952	.	.	.	.	0.447252	6.47E-05	.	.	yes
MCM4	.	.	0.3163186	0.0060395	.	.	.	.	0.3725753	0.0010793	.	.	yes
MCM5	.	.	.	.	.	.	.	.	.	.	.	.	yes
MCM6	.	.	.	.	.	.	.	.	.	.	.	.	yes
MCM7	.	.	.	.	0.318541	0.0056751	.	.	.	.	.	.	yes
MCPH1	.	.	0.4011422	0.0003971	.	.	.	.	.	.	.	.	.
MCU	0.5295126	1.23E-06	.	.	.	.	.	.	.	.	.	.	.
MCUR1	.	.	0.3482682	0.0023602	.	.	.	.	0.4813879	1.41E-05	.	.	.
MDC1	.	.	.	.	.	.	.	.	.	.	.	.	yes

Gene	Atrial Expression	p-value Atrial	AV-Canal Expression	p-value AV-Canal	Branchial Arches Expression	p-value Branchial Arches	Heart Tube Expression	p-value Heart Tube	Outflow Tract Expression	p-value Outflow Tract	Ventricle Expression	p-value Ventricle	DMP Expression
MDGA1	.	.	.	.	.	.	.	.	.	.	0.7108719	1.29E-12	.
MDH1	0.3582315	0.0017252	0.385445	0.0006956	.	.	.	.	.	.	.	.	yes
MDH2	.	.	0.5084106	3.75E-06	.	.	.	.	0.5493408	4.01E-07	.	.	yes
MDK	.	.	.	.	.	.	.	.	.	.	.	.	yes
MDM2	.	.	0.5118185	3.15E-06	.	.	.	.	.	.	.	.	.
MDN1	.	.	.	.	.	.	.	.	.	.	.	.	yes
MDP1	.	.	.	.	.	.	.	.	0.5896705	3.24E-08	.	.	.
ME2	.	.	0.4285812	0.0001392	.	.	.	.	.	.	.	.	.
MED12	.	.	.	.	.	.	.	.	0.68617	1.51E-11	.	.	.
MED24	.	.	.	.	.	.	.	.	0.603914	1.22E-08	.	.	.
MED28	.	.	.	.	.	.	.	.	0.6272271	2.24E-09	.	.	.
MED7	0.4575524	4.16E-05	.	.	.	.	.	.	.	.	.	.	.
MED8	.	.	.	.	.	.	.	.	0.5664893	1.43E-07	.	.	.
MEDAG	.	.	.	.	.	.	.	.	0.6078618	9.27E-09	.	.	.
MEF2A	.	.	0.4725208	2.13E-05	.	.	.	.	.	.	.	.	.
MEF2BNB	.	.	0.5864207	4.02E-08	.	.	.	.	0.4489508	6.02E-05	.	.	.
MEIS1	.	.	.	.	.	.	.	.	0.4079496	0.0003087	.	.	yes
MEIS2	.	.	.	.	.	.	.	.	.	.	.	.	yes
MEOX1	.	.	0.6409695	7.68E-10	.	.	.	.	.	.	.	.	.
MESP1	.	.	0.4193713	0.0001999	.	.	.	.	.	.	.	.	.
MEST	.	.	.	.	.	.	.	.	0.3094538	0.0072987	0.4309974	0.0001264	yes
METRN	.	.	.	.	.	.	.	.	.	.	.	.	yes
METTL10	.	.	.	.	.	.	.	.	.	.	0.7019434	3.22E-12	.
METTL13	.	.	.	.	.	.	.	.	.	.	0.6437387	6.15E-10	.
METTL20	.	.	.	.	.	.	.	.	0.6358124	1.15E-09	.	.	.
METTL21A	.	.	.	.	.	.	.	.	0.7462872	2.33E-14	.	.	.
METTL22	.	.	0.4904767	9.16E-06	.	.	.	.	0.6656643	9.75E-11	.	.	.
METTL3	.	.	0.3980797	0.000444	.	.	.	.	.	.	.	.	.
METTL4	.	.	.	.	.	.	.	.	0.57171	1.04E-07	.	.	.
MEX3A	.	.	0.4060597	0.0003312	.	.	.	.	0.3039496	0.0084693	.	.	yes
MFHAS1	.	.	.	.	.	.	.	.	0.5037361	4.76E-06	.	.	.
MFN1	.	.	.	.	.	.	.	.	0.7212266	4.24E-13	.	.	.
MFSD1	.	.	0.4051035	0.0003432	.	.	.	.	0.382437	0.000772	.	.	.
MFSD10	.	.	0.4331229	0.000116	.	.	.	.	.	.	.	.	.
MFSD8	.	.	.	.	.	.	.	.	0.6279149	2.12E-09	.	.	.
MFSD9	.	.	.	.	.	.	.	.	.	.	0.6956108	6.06E-12	.
MGAT4B	.	.	0.3861599	0.0006784	.	.	.	.	.	.	.	.	.
MGEA5	.	.	.	.	.	.	.	.	.	.	.	.	yes
MGRN1	.	.	.	.	.	.	.	.	.	.	0.6502024	3.63E-10	.
MICAL2	.	.	0.603775	1.24E-08	.	.	.	.	0.4924469	8.32E-06	.	.	.
MICALL2	.	.	.	.	0.6102871	7.80E-09	.	.	.	.	.	.	.
MICU1	.	.	0.4309863	0.0001264	.	.	.	.	.	.	.	.	.
MIDN	.	.	0.3271572	0.0044391	.	.	.	.	.	.	.	.	yes
MIEF1	.	.	0.4624143	3.36E-05	.	.	.	.	.	.	.	.	.
MIEF2	.	.	.	.	.	.	.	.	0.4539658	4.86E-05	.	.	.
MIEN1	.	.	.	.	.	.	.	.	0.707157	1.89E-12	.	.	.
MIER1	.	.	.	.	.	.	.	.	.	.	0.4437949	7.48E-05	.
MIF	.	.	0.4001954	0.0004111	.	.	.	.	0.5230493	1.74E-06	.	.	.
MINPP1	.	.	.	.	.	.	.	.	0.6347188	1.26E-09	.	.	.
MIS18A	.	.	0.4264622	0.0001514	0.4004667	0.000407	.	.	0.442289	7.96E-05	.	.	.
MKI67	.	.	.	.	.	.	.	.	.	.	.	.	yes
MKKS	.	.	.	.	.	.	.	.	0.7560382	6.85E-15	.	.	.
MKL2	.	.	0.4610107	3.57E-05	.	.	.	.	.	.	.	.	.
MKNK2	.	.	.	.	0.432327	0.0001198	.	.	.	.	.	.	yes
MKRN2	.	.	.	.	.	.	.	.	0.6611945	1.44E-10	.	.	.
MLEC	.	.	0.4087045	0.0003001	.	.	.	.	0.4993361	5.93E-06	.	.	yes
MLF2	.	.	.	.	.	.	.	.	0.4383833	9.36E-05	.	.	yes
MLLT1	.	.	.	.	.	.	.	.	0.4933258	7.98E-06	0.3917473	0.0005572	.
MLLT4	.	.	.	.	.	.	.	.	.	.	.	.	yes
MMD	.	.	0.4509025	5.54E-05	.	.	.	.	0.5154343	2.61E-06	.	.	.
MMP24	.	.	.	.	.	.	.	.	0.4956876	7.10E-06	.	.	.
MMP27	.	.	0.3957455	0.000483	.	.	.	.	.	.	.	.	.
MMRN2	.	.	.	.	.	.	.	.	0.5337902	9.71E-07	.	.	.
MNT	.	.	.	.	0.4489066	6.03E-05	.	.	.	.	.	.	.
MOB1B	.	.	0.4854095	1.17E-05	.	.	.	.	0.4199669	0.0001953	.	.	.
MOB2	.	.	.	.	.	.	.	.	0.5461097	4.84E-07	.	.	.
MOB3A	.	.	0.369769	0.001185	.	.	.	.	0.563383	1.73E-07	.	.	.
MOGAT2	.	.	.	.	0.6541637	2.61E-10	.	.	.	.	.	.	.
MORC2	.	.	.	.	.	.	.	.	0.6460698	5.09E-10	.	.	.
MORC3	.	.	.	.	.	.	.	.	.	.	0.6935179	7.44E-12	.
MORF4L2	.	.	.	.	0.3715516	0.0011168	.	.	.	.	.	.	yes
MOSPD1	.	.	.	.	.	.	.	.	0.514499	2.74E-06	.	.	.
MOSPD2	.	.	.	.	.	.	.	.	.	.	0.5442411	5.39E-07	.
MOV10L1	.	.	.	.	.	.	.	.	0.8772545	1.20E-24	.	.	.
MPHOSPH10	.	.	0.3484163	0.0023494	.	.	.	.	0.588085	3.60E-08	.	.	.

Gene	Atrial Expression	p-value Atrial	AV-Canal Expression	p-value AV-Canal	Branchial Arches Expression	p-value Branchial Arches	Heart Tube Expression	p-value Heart Tube	Outflow Tract Expression	p-value Outflow Tract	Ventricle Expression	p-value Ventricle	DMP Expression
MPHOSPH6	.	.	.	.	.	.	.	.	0.5203076	2.02E-06	.	.	.
MPHOSPH8	.	.	.	.	.	.	.	.	0.5315834	1.10E-06	.	.	.
MPO	.	.	.	.	0.5042505	4.64E-06	.	.	.	.	.	.	.
MPP2	.	.	.	.	.	.	.	.	0.7163535	7.20E-13	.	.	.
MPP6	.	.	.	.	.	.	.	.	0.6039513	1.22E-08	.	.	.
MPPE1	.	.	.	.	.	.	.	.	0.601094	1.49E-08	.	.	.
MPPED2	.	.	.	.	.	.	0.4428121	7.79E-05	0.4546692	4.71E-05	.	.	.
MPRIP	.	.	.	.	.	.	.	.	0.5754377	8.18E-08	0.3031564	0.0086509	yes
MPZL1	.	.	.	.	.	.	.	.	0.6384351	9.39E-10	.	.	.
MRE11A	.	.	.	.	.	.	0.5402141	6.77E-07	.	.	.	.	.
MRGBP	.	.	.	.	.	.	.	.	0.4797894	1.52E-05	.	.	.
MRGPRE	.	.	.	.	.	.	.	.	0.4713428	2.25E-05	.	.	.
MRM1	.	.	.	.	.	.	.	.	0.8146405	1.05E-18	.	.	.
MROH8	.	.	.	.	.	.	.	.	.	.	0.4798839	1.52E-05	.
MRPL12	.	.	.	.	.	.	.	.	0.6205073	3.71E-09	.	.	.
MRPL16	.	.	0.3913405	0.0005653	.	.	.	.	.	.	.	.	.
MRPL17	.	.	.	.	.	.	.	.	0.5653303	1.54E-07	.	.	.
MRPL3	.	.	0.4125832	0.0002593	.	.	.	.	0.6035216	1.26E-08	.	.	.
MRPL30	.	.	0.3216873	0.0051924	.	.	.	.	0.5730246	9.53E-08	.	.	.
MRPL34	.	.	.	.	.	.	.	.	0.7387362	5.78E-14	.	.	.
MRPL36	.	.	0.3592706	0.0016687	.	.	.	.	0.6632405	1.20E-10	.	.	.
MRPL38	.	.	.	.	0.346048	0.0025275	.	.	0.5632843	1.74E-07	.	.	.
MRPL44	.	.	0.3639521	0.0014345	.	.	.	.	0.5044465	4.59E-06	.	.	.
MRPL45	.	.	0.4158001	0.0002294	0.4020183	0.0003846	.	.	0.4622837	3.38E-05	.	.	.
MRPL52	.	.	0.3599122	0.0016347	.	.	.	.	0.5190609	2.16E-06	.	.	.
MRPL55	.	.	.	.	.	.	.	.	0.6500299	3.68E-10	.	.	.
MRPL9	.	.	.	.	.	.	.	.	0.5023564	5.10E-06	.	.	.
MRPS11	.	.	0.443646	7.53E-05	.	.	.	.	.	.	.	.	.
MRPS22	.	.	.	.	.	.	.	.	0.4684367	2.56E-05	.	.	.
MRPS23	.	.	0.4103567	0.0002821	.	.	.	.	.	.	.	.	.
MRPS26	.	.	0.4217516	0.0001822	.	.	.	.	.	.	.	.	.
MRPS27	.	.	0.453279	5.00E-05	.	.	.	.	.	.	.	.	.
MRPS30	.	.	0.4063621	0.0003275	.	.	.	.	0.5945537	2.33E-08	.	.	.
MRPS34	.	.	.	.	.	.	.	.	0.6651343	1.02E-10	.	.	.
MRPS35	.	.	.	.	.	.	.	.	0.6482816	4.25E-10	.	.	.
MRPS7	.	.	.	.	.	.	.	.	0.5279874	1.34E-06	.	.	.
MRPS9	.	.	0.4770328	1.73E-05	.	.	.	.	.	.	.	.	.
MS4A1	.	.	.	.	.	.	.	.	.	.	0.5291305	1.25E-06	.
MSANTD1	.	.	0.5392063	7.17E-07	.	.	.	.	.	.	.	.	.
MSH6	.	.	.	.	.	.	.	.	.	.	.	.	yes
MSI1	.	.	.	.	0.5931696	2.56E-08	.	.	.	.	.	.	.
MSN	.	.	0.3027955	0.0087347	.	.	.	.	0.404457	0.0003515	.	.	yes
MST1R	.	.	.	.	.	.	.	.	.	.	0.6199759	3.85E-09	.
MSTO1	.	.	.	.	.	.	.	.	0.4425881	7.87E-05	.	.	.
MSX2	.	.	0.7048955	2.39E-12	.	.	.	.	.	.	.	.	.
MT3	.	.	0.4995188	5.88E-06	.	.	.	.	.	.	.	.	.
MTA1	.	.	.	.	.	.	.	.	.	.	.	.	yes
MTA2	.	.	.	.	.	.	.	.	.	.	.	.	yes
MTAP	.	.	.	.	0.5542772	3.00E-07	.	.	.	.	.	.	.
MTCH1	.	.	.	.	.	.	.	.	.	.	.	.	yes
MTF1	.	.	.	.	.	.	.	.	0.6752941	4.13E-11	.	.	.
MTF2	.	.	.	.	.	.	.	.	0.4634025	3.21E-05	.	.	.
MTFR1L	.	.	.	.	.	.	.	.	0.5442563	5.38E-07	.	.	.
MTIF3	.	.	.	.	.	.	.	.	0.5404708	6.68E-07	.	.	.
MTMR14	.	.	.	.	.	.	.	.	0.5723369	9.96E-08	.	.	.
MTMR3	.	.	0.3762519	0.0009537	.	.	.	.	0.4733115	2.05E-05	.	.	.
MTMR4	.	.	.	.	0.3292216	0.0041811	.	.	.	.	.	.	yes
MTNR1A	.	.	.	.	.	.	.	.	.	.	0.6047146	1.16E-08	.
MTO1	.	.	0.3839721	0.0007321	.	.	.	.	0.5166659	2.45E-06	.	.	.
MTPN	.	.	0.3349489	0.0035336	.	.	.	.	0.3421897	0.0028436	.	.	yes
MTSS1	.	.	0.3356577	0.00346	.	.	.	.	0.4838775	1.26E-05	.	.	.
MTSS1L	.	.	.	.	0.3583355	0.0017195	.	.	0.5253441	1.54E-06	.	.	.
MTUS2	.	.	.	.	.	.	.	.	0.6508971	3.43E-10	.	.	.
MUS81	.	.	0.5013333	5.37E-06	.	.	.	.	.	.	.	.	.
MUSK	.	.	.	.	.	.	.	.	.	.	0.6681775	7.82E-11	.
MYADM	.	.	.	.	.	.	.	.	0.6552394	2.39E-10	.	.	.
MYBBP1A	.	.	.	.	.	.	.	.	.	.	.	.	yes
MYBPC3	.	.	.	.	.	.	.	.	0.6847301	1.73E-11	.	.	.
MYBPHL	0.4078198	0.0003102	0.4419432	8.08E-05	.	.	.	.	.	.	.	.	.
MYCN	.	.	.	.	0.7101961	1.38E-12	.	.	.	.	.	.	.
MYH1	.	.	.	.	.	.	.	.	.	.	0.6158154	5.23E-09	.
MYH10	.	.	0.3408809	0.0029586	.	.	.	.	0.3463477	0.0025043	.	.	yes
MYH4	.	.	.	.	.	.	.	.	0.6305532	1.74E-09	.	.	.
MYH6	.	.	.	.	.	.	.	.	0.6644186	1.09E-10	.	.	yes
MYH7	.	.	.	.	.	.	.	.	0.8096118	2.52E-18	.	.	.

Gene	Atrial Expression	p-value Atrial	AV-Canal Expression	p-value AV-Canal	Branchial Arches Expression	p-value Branchial Arches	Heart Tube Expression	p-value Heart Tube	Outflow Tract Expression	p-value Outflow Tract	Ventricle Expression	p-value Ventricle	DMP Expression
MYH9	.	.	.	.	.	.	.	.	0.6593256	1.69E-10	.	.	yes
MYL12A	.	.	0.3138579	0.0064669	.	.	.	.	0.5352286	8.96E-07	.	.	.
MYL2	.	.	.	.	.	.	.	.	.	.	0.632706	1.47E-09	.
MYL3	.	.	0.4338239	0.0001127	.	.	.	.	0.6953798	6.20E-12	.	.	.
MYL4	.	.	.	.	.	.	.	.	0.9941315	2.71E-71	.	.	yes
MYL6	.	.	.	.	.	.	.	.	0.6765136	3.70E-11	.	.	.
MYL7	.	.	0.5020509	5.18E-06	.	.	0.4248988	0.0001611	0.5133324	2.91E-06	.	.	yes
MYL9	.	.	0.693923	7.15E-12	.	.	.	.	.	.	.	.	.
MYLK3	.	.	0.3146094	0.0063336	.	.	0.3165035	0.0060084	0.6978034	4.88E-12	.	.	.
MYLK4	.	.	.	.	.	.	.	.	.	.	0.5425836	5.92E-07	.
MYO18A	.	.	.	.	.	.	.	.	.	.	0.4478489	6.31E-05	.
MYO18B	.	.	.	.	.	.	.	.	0.4925159	8.30E-06	.	.	.
MYO1G	.	.	.	.	.	.	.	.	0.4851548	1.18E-05	.	.	.
MYO3B	.	.	.	.	.	.	.	.	.	.	0.6708858	6.15E-11	.
MYO7A	.	.	.	.	.	.	.	.	.	.	0.6979348	4.82E-12	.
MYO9B	.	.	.	.	.	.	.	.	0.6515185	3.25E-10	.	.	.
MYOCD	.	.	.	.	.	.	0.3065396	0.0078995	0.5501781	3.82E-07	.	.	.
MYOM1	.	.	0.3212934	0.0052508	.	.	.	.	0.4696379	2.43E-05	.	.	.
MYPOP	.	.	.	.	0.4679674	2.62E-05	.	.	.	.	.	.	.
MYRF	.	.	0.3833106	0.0007491	.	.	.	.	.	.	.	.	yes
MZT1	.	.	.	.	.	.	.	.	0.4423845	7.93E-05	.	.	.
MZT2B	.	.	.	.	.	.	.	.	0.5039578	4.71E-06	.	.	.
NAA15	.	.	.	.	.	.	.	.	0.3227609	0.0050362	.	.	yes
NAA40	.	.	.	.	.	.	.	.	0.5811773	5.66E-08	.	.	.
NAA50	.	.	0.3625993	0.001499	.	.	.	.	.	.	.	.	yes
NAB1	.	.	0.4727576	2.11E-05	.	.	.	.	.	.	.	.	.
NACC2	.	.	0.4565293	4.35E-05	.	.	.	.	.	.	.	.	.
NADK2	.	.	.	.	.	.	.	.	0.7388599	5.69E-14	.	.	.
NAP1L1	.	.	.	.	.	.	0.3395351	0.0030811	0.4926845	8.23E-06	.	.	yes
NAP1L4	.	.	0.3338654	0.0036488	.	.	.	.	.	.	.	.	yes
NARFL	.	.	0.3510742	0.002163	.	.	.	.	0.6501033	3.66E-10	.	.	.
NARS	.	.	.	.	0.325936	0.0045983	.	.	.	.	.	.	yes
NARS2	.	.	0.5629886	1.78E-07	.	.	.	.	.	.	.	.	.
NASP	.	.	.	.	.	.	.	.	.	.	.	.	yes
NAT9	.	.	.	.	.	.	.	.	0.4784103	1.62E-05	.	.	.
NAV3	.	.	.	.	.	.	.	.	.	.	0.6289999	1.96E-09	.
NBN	.	.	.	.	.	.	.	.	0.5793325	6.38E-08	.	.	.
NBR1	.	.	.	.	.	.	.	.	.	.	0.6726965	5.23E-11	.
NCAM1	.	.	0.3832354	0.000751	.	.	.	.	.	.	.	.	.
NCAPD2	.	.	.	.	.	.	.	.	0.4274879	0.0001454	.	.	yes
NCBP1	.	.	.	.	.	.	.	.	.	.	.	.	yes
NCBP2	.	.	.	.	.	.	.	.	0.5001167	5.71E-06	.	.	.
NCK2	.	.	0.3896717	0.0005997	.	.	.	.	.	.	.	.	.
NCKAP1	.	.	.	.	.	.	.	.	.	.	.	.	yes
NCKAP5L	0.4749246	1.91E-05	.	.	.	.	.	.	0.318411	0.0056958	.	.	.
NCL	.	.	.	.	.	.	.	.	.	.	.	.	yes
NCLN	.	.	.	.	.	.	.	.	0.7123364	1.10E-12	.	.	.
NCOA1	.	.	.	.	.	.	.	.	.	.	0.6364394	1.10E-09	.
NCOA6	.	.	0.4766131	1.76E-05	.	.	.	.	.	.	.	.	.
NCOR1	.	.	0.342177	0.0028447	.	.	.	.	.	.	.	.	yes
NCOR2	.	.	.	.	.	.	.	.	.	.	.	.	yes
NDFIP1	.	.	0.521433	1.90E-06	.	.	.	.	0.395726	0.0004833	.	.	.
NDUFA10	.	.	0.5231784	1.73E-06	.	.	.	.	0.5141555	2.79E-06	.	.	.
NDUFA2	.	.	0.5334168	9.91E-07	.	.	.	.	0.430458	0.0001291	.	.	.
NDUFA3	.	.	0.4460389	6.81E-05	.	.	.	.	.	.	.	.	.
NDUFA5	.	.	0.4244562	0.0001639	.	.	.	.	0.3003021	0.0093328	.	.	.
NDUFA6	.	.	0.459859	3.76E-05	.	.	.	.	.	.	.	.	.
NDUFA9	.	.	0.4195804	0.0001983	.	.	.	.	.	.	.	.	.
NDUFAF2	.	.	.	.	.	.	.	.	0.4888604	9.90E-06	.	.	.
NDUFAF6	.	.	.	.	.	.	.	.	.	.	0.7407355	4.56E-14	.
NDUFB10	.	.	0.3592431	0.0016702	.	.	.	.	0.7262046	2.44E-13	.	.	.
NDUFB7	.	.	0.4057804	0.0003347	.	.	.	.	0.7009564	3.56E-12	.	.	.
NDUFB8	.	.	0.4267561	0.0001497	.	.	.	.	.	.	.	.	.
NDUFS2	.	.	0.4148605	0.0002378	.	.	.	.	0.3539907	0.0019738	.	.	yes
NDUFS3	.	.	0.3508648	0.0021771	.	.	.	.	0.5946194	2.32E-08	.	.	.
NDUFS5	.	.	0.4132355	0.0002529	.	.	.	.	0.3487356	0.0023262	.	.	.
NDUFS8	.	.	.	.	.	.	.	.	0.5104345	3.38E-06	.	.	.
NDUFV1	.	.	0.4447013	7.20E-05	.	.	.	.	0.5122184	3.09E-06	.	.	.
NDUFV2	.	.	0.3599948	0.0016304	.	.	.	.	0.6054139	1.10E-08	.	.	.
NDUFV3	.	.	0.3255178	0.004654	.	.	.	.	0.6708466	6.17E-11	.	.	.
NECAP1	.	.	.	.	.	.	.	.	0.4804606	1.48E-05	.	.	.
NEDD4	.	.	0.5088208	3.68E-06	.	.	.	.	.	.	.	.	yes
NEIL1	.	.	.	.	.	.	.	.	.	.	0.5987838	1.75E-08	.
NEIL3	.	.	0.578504	6.73E-08	.	.	.	.	.	.	.	.	.
NEK3	.	.	.	.	.	.	.	.	0.5341096	9.54E-07	.	.	.

Gene	Atrial Expression	p-value Atrial	AV-Canal Expression	p-value AV-Canal	Branchial Arches Expression	p-value Branchial Arches	Heart Tube Expression	p-value Heart Tube	Outflow Tract Expression	p-value Outflow Tract	Ventricle Expression	p-value Ventricle	DMP Expression
NEK6	.	.	0.4678014	2.64E-05	.	.	.	.	.	.	.	.	.
NEO1	.	.	.	.	.	.	.	.	.	.	.	.	yes
NES	.	.	.	.	.	.	.	.	0.5450035	5.15E-07	.	.	yes
NEU3	.	.	0.4451179	7.08E-05	.	.	.	.	.	.	.	.	.
NEURL2	.	.	.	.	.	.	.	.	0.777931	3.53E-16	.	.	.
NF2	.	.	.	.	.	.	.	.	.	.	0.4720266	2.18E-05	.
NFAT5	.	.	0.3752856	0.0009854	.	.	.	.	.	.	.	.	.
NFATC1	.	.	0.5509763	3.64E-07	.	.	.	.	.	.	.	.	.
NFE2L1	.	.	.	.	.	.	.	.	0.5011067	5.43E-06	.	.	yes
NFE2L2	.	.	.	.	.	.	0.5522249	3.39E-07	.	.	.	.	.
NFIA	.	.	0.3821417	0.0007799	.	.	.	.	.	.	.	.	.
NFIC	.	.	.	.	.	.	.	.	0.784994	1.26E-16	.	.	.
NFIL3	.	.	.	.	.	.	.	.	0.6082586	9.02E-09	.	.	.
NFKBIB	.	.	.	.	.	.	.	.	0.5606508	2.05E-07	.	.	.
NFKBID	.	.	.	.	.	.	.	.	0.4837638	1.26E-05	.	.	.
NFYA	.	.	0.5359213	8.62E-07	.	.	.	.	.	.	.	.	.
NGFRAP1	.	.	.	.	.	.	.	.	0.5894292	3.29E-08	.	.	yes
NID1	.	.	.	.	.	.	.	.	.	.	.	.	yes
NID2	.	.	0.4906172	9.10E-06	.	.	.	.	0.5033665	4.85E-06	.	.	.
NINJ1	.	.	0.4383272	9.38E-05	.	.	.	.	.	.	.	.	.
NIPA2	.	.	.	.	.	.	0.6416531	7.27E-10	.	.	.	.	.
NIPAL4	.	.	0.387312	0.0006516	.	.	.	.	.	.	.	.	.
NIPSNAP3A	.	.	.	.	.	.	.	.	.	.	0.7018192	3.26E-12	.
NIPSNAP3B	.	.	0.4645404	3.05E-05	.	.	.	.	0.3402349	0.0030169	.	.	.
NKAIN1	.	.	.	.	0.7170822	6.65E-13	.	.	.	.	.	.	.
NKAP	.	.	.	.	.	.	.	.	.	.	0.7091911	1.53E-12	.
NKTR	.	.	.	.	.	.	.	.	.	.	.	.	yes
NKX2-1	.	.	.	.	.	.	.	.	0.4436109	7.54E-05	.	.	.
NKX2-5	.	.	0.4314536	0.0001241	.	.	.	.	.	.	.	.	.
NKX2-6	.	.	.	.	.	.	.	.	0.7639276	2.44E-15	.	.	.
NLE1	.	.	0.3275293	0.0043916	.	.	.	.	0.4938223	7.78E-06	.	.	.
NLK	.	.	.	.	.	.	.	.	0.49076	9.03E-06	.	.	.
NLRP5	.	.	0.4148421	0.0002379	.	.	.	.	.	.	.	.	.
NME1	.	.	.	.	.	.	.	.	0.4525039	5.17E-05	.	.	yes
NMRK2	.	.	0.5383463	7.53E-07	.	.	.	.	.	.	.	.	.
NNAT	.	.	.	.	.	.	.	.	.	.	.	.	yes
NNMT	.	.	.	.	.	.	.	.	0.7315904	1.33E-13	.	.	.
NOC2L	.	.	0.303603	0.0085483	.	.	.	.	0.3141539	0.0064141	.	.	yes
NOC3L	.	.	.	.	.	.	.	.	0.7436478	3.21E-14	.	.	.
NOG	.	.	0.4245817	0.0001631	.	.	.	.	0.4005631	0.0004056	.	.	.
NOL9	.	.	.	.	.	.	.	.	0.5318024	1.08E-06	.	.	.
NOLC1	.	.	.	.	.	.	.	.	.	.	.	.	yes
NOM1	.	.	.	.	0.5364929	8.35E-07	.	.	.	.	.	.	.
NOMO1	.	.	.	.	.	.	.	.	0.6271438	2.25E-09	.	.	.
NONO	.	.	.	.	.	.	.	.	.	.	.	.	yes
NOP14	.	.	.	.	.	.	.	.	0.7792215	2.93E-16	.	.	.
NOP2	.	.	0.507899	3.85E-06	.	.	.	.	0.4191058	0.000202	.	.	.
NOP58	.	.	.	.	0.3609539	0.0015808	.	.	.	.	.	.	yes
NOS3	.	.	0.4287613	0.0001382	.	.	.	.	.	.	.	.	.
NOTCH3	.	.	.	.	.	.	.	.	.	.	.	.	yes
NOX1	.	.	0.4477401	6.34E-05	.	.	.	.	.	.	.	.	.
NPAS4	.	.	0.4618536	3.44E-05	.	.	.	.	.	.	.	.	.
NPBWR1	.	.	.	.	.	.	.	.	.	.	0.5239973	1.66E-06	.
NPEPPS	.	.	0.4712767	2.25E-05	.	.	.	.	.	.	.	.	.
NPHS1	.	.	.	.	.	.	.	.	0.5104827	3.37E-06	.	.	.
NPLOC4	.	.	0.6052971	1.11E-08	.	.	.	.	.	.	.	.	.
NPM1	.	.	.	.	.	.	.	.	.	.	.	.	yes
NPPA	0.3550859	0.0019067	.	.	.	.	.	.	.	.	0.4433196	7.63E-05	.
NPR3	.	.	0.4752439	1.88E-05	.	.	.	.	.	.	.	.	.
NPTX2	.	.	.	.	.	.	0.3903822	0.0005849	0.6804399	2.58E-11	.	.	.
NR0B2	.	.	0.5600948	2.12E-07	.	.	.	.	.	.	.	.	.
NR1D2	.	.	0.5006565	5.55E-06	.	.	.	.	.	.	.	.	.
NR1H3	.	.	.	.	.	.	.	.	0.7129569	1.03E-12	.	.	.
NR2F2	.	.	.	.	.	.	.	.	.	.	.	.	yes
NR2F6	.	.	.	.	.	.	.	.	0.4463793	6.71E-05	.	.	.
NR4A2	.	.	.	.	.	.	0.5417794	6.20E-07	.	.	.	.	.
NR4A3	.	.	.	.	.	.	.	.	.	.	0.653044	2.87E-10	.
NRAS	.	.	0.3097222	0.0072454	.	.	.	.	.	.	.	.	yes
NRCAM	.	.	.	.	.	.	.	.	.	.	0.6035739	1.25E-08	.
NRD1	.	.	.	.	.	.	.	.	.	.	.	.	yes
NREP	.	.	.	.	.	.	.	.	.	.	.	.	yes
NRIP3	.	.	.	.	.	.	.	.	.	.	0.5761268	7.83E-08	.
NRM	.	.	.	.	.	.	0.4539583	4.86E-05	.	.	.	.	.
NRP1	.	.	0.4228343	0.0001747	.	.	.	.	.	.	.	.	.
NSD1	.	.	.	.	.	.	.	.	.	.	.	.	yes

Gene	Atrial Expression	p-value Atrial	AV-Canal Expression	p-value AV-Canal	Branchial Arches Expression	p-value Branchial Arches	Heart Tube Expression	p-value Heart Tube	Outflow Tract Expression	p-value Outflow Tract	Ventricle Expression	p-value Ventricle	DMP Expression
NSMCE4A	.	.	.	.	.	.	.	.	0.6179649	4.47E-09	.	.	.
NSUN2	.	.	.	.	.	.	.	.	.	.	.	.	yes
NSUN7	.	.	.	.	.	.	.	.	0.4973788	6.54E-06	.	.	.
NT5M	.	.	.	.	.	.	.	.	0.7169521	6.75E-13	.	.	.
NTF3	.	.	0.4252382	0.0001589	.	.	0.3966196	0.000468	.	.	.	.	.
NTM	.	.	.	.	.	.	.	.	0.7255016	2.64E-13	.	.	.
NTN4	.	.	0.4082666	0.0003051	.	.	.	.	.	.	.	.	.
NTPCR	.	.	0.4570725	4.24E-05	.	.	.	.	.	.	.	.	.
NUAK1	.	.	.	.	.	.	.	.	0.6090101	8.55E-09	.	.	.
NUB1	.	.	.	.	.	.	.	.	0.4875601	1.05E-05	.	.	.
NUBP1	.	.	0.5427682	5.86E-07	.	.	.	.	.	.	.	.	.
NUCKS1	.	.	.	.	.	.	.	.	0.768306	1.35E-15	.	.	yes
NUDT12	.	.	.	.	.	.	0.4412162	8.33E-05	.	.	.	.	.
NUDT19	.	.	0.4007879	0.0004023	.	.	.	.	.	.	.	.	.
NUDT2	.	.	.	.	.	.	.	.	0.4857087	1.15E-05	.	.	.
NUDT3	.	.	.	.	.	.	.	.	0.4846672	1.21E-05	.	.	.
NUDT4	.	.	0.3015015	0.0090408	.	.	.	.	.	.	.	.	yes
NUDT5	.	.	.	.	.	.	.	.	0.4666886	2.77E-05	.	.	.
NUFIP2	.	.	.	.	.	.	0.4390235	9.12E-05	0.3164506	0.0060173	.	.	.
NUMA1	.	.	.	.	.	.	.	.	.	.	.	.	yes
NUMBL	.	.	.	.	0.4934952	7.91E-06	.	.	.	.	.	.	.
NUP133	.	.	.	.	.	.	.	.	0.5595406	2.19E-07	.	.	.
NUP153	.	.	.	.	0.43775	9.61E-05	.	.	.	.	.	.	yes
NUP155	.	.	.	.	.	.	.	.	0.4695932	2.43E-05	.	.	yes
NUP188	.	.	.	.	.	.	.	.	.	.	.	.	yes
NUP205	.	.	.	.	.	.	.	.	.	.	.	.	yes
NUP62	.	.	.	.	.	.	.	.	0.4647834	3.02E-05	.	.	yes
NUP88	.	.	0.3852987	0.0006991	.	.	.	.	.	.	.	.	.
NUS1	.	.	.	.	.	.	.	.	0.5103612	3.40E-06	.	.	.
NUTF2	.	.	.	.	.	.	.	.	0.6770711	3.52E-11	.	.	.
NXF1	.	.	.	.	.	.	.	.	0.3407316	0.002972	.	.	yes
NXF3	.	.	.	.	.	.	.	.	.	.	0.4536215	4.93E-05	.
NXPE3	.	.	.	.	.	.	.	.	0.7185297	5.69E-13	.	.	.
NYNRIN	.	.	.	.	.	.	.	.	.	.	.	.	yes
OBSCN	.	.	.	.	.	.	.	.	0.7374644	6.71E-14	.	.	.
OBSL1	.	.	.	.	.	.	.	.	0.73346	1.07E-13	.	.	yes
OCLN	.	.	.	.	.	.	.	.	.	.	0.4820978	1.37E-05	.
ODC1	.	.	0.4074289	0.0003148	.	.	.	.	0.5746382	8.61E-08	.	.	.
ODF2L	.	.	.	.	.	.	.	.	.	.	0.6385661	9.29E-10	.
OGDH	.	.	0.3980705	0.0004441	.	.	.	.	.	.	.	.	yes
OGFR	.	.	.	.	.	.	.	.	0.5542006	3.01E-07	.	.	.
OGG1	.	.	.	.	.	.	.	.	0.6497802	3.76E-10	.	.	.
OGT	.	.	.	.	.	.	.	.	0.4017604	0.0003882	.	.	yes
OLFML2B	.	.	.	.	.	.	.	.	0.469632	2.43E-05	.	.	.
OMD	.	.	.	.	.	.	.	.	.	.	0.6971037	5.23E-12	.
OPA1	.	.	.	.	.	.	.	.	0.6233646	2.99E-09	.	.	.
OPN1SW	.	.	.	.	.	.	.	.	0.8069254	3.96E-18	.	.	.
OPN3	.	.	.	.	.	.	.	.	0.4875836	1.05E-05	.	.	.
OPN4	.	.	0.3554007	0.0018878	.	.	.	.	0.590639	3.04E-08	.	.	.
OPTN	.	.	.	.	.	.	.	.	0.5580318	2.40E-07	.	.	.
OSBPL3	.	.	.	.	.	.	.	.	.	.	0.4989023	6.06E-06	.
OSGIN1	.	.	0.4307854	0.0001274	.	.	.	.	.	.	.	.	.
OSTC	.	.	.	.	.	.	.	.	0.543752	5.54E-07	.	.	.
OSTM1	.	.	0.5344168	9.38E-07	.	.	.	.	0.5791738	6.44E-08	.	.	.
OTUB1	.	.	.	.	.	.	.	.	0.5293134	1.24E-06	.	.	.
OTUB2	.	.	.	.	.	.	.	.	.	.	0.5717242	1.03E-07	.
OTUD1	.	.	.	.	.	.	.	.	0.622988	3.08E-09	.	.	.
OTUD4	.	.	.	.	.	.	.	.	.	.	.	.	yes
OTUD5	.	.	.	.	.	.	.	.	0.530243	1.18E-06	.	.	.
OTUD6B	.	.	0.4362609	0.0001021	.	.	.	.	.	.	.	.	.
OXA1L	.	.	0.4200869	0.0001944	.	.	.	.	0.5214164	1.90E-06	.	.	.
OXCT1	.	.	0.3856379	0.0006909	.	.	.	.	.	.	.	.	.
OXNAD1	.	.	0.3356474	0.0034611	.	.	.	.	0.4380422	9.49E-05	.	.	.
P2RX6	.	.	.	.	.	.	0.4561243	4.42E-05	.	.	.	.	.
P2RY12	.	.	.	.	.	.	.	.	.	.	0.6970409	5.26E-12	.
P4HB	.	.	.	.	.	.	.	.	0.5276178	1.36E-06	.	.	yes
P4HTM	.	.	.	.	.	.	.	.	0.6729914	5.09E-11	.	.	.
PA2G4	.	.	0.315554	0.0061695	0.5100464	3.45E-06	.	.	.	.	.	.	yes
PABPC1	.	.	0.4787403	1.60E-05	.	.	.	.	0.4558097	4.48E-05	.	.	yes
PABPC4	.	.	0.4291654	0.000136	.	.	.	.	.	.	.	.	.
PABPC4L	.	.	.	.	.	.	.	.	.	.	0.4772789	1.71E-05	.
PABPN1	.	.	.	.	0.3681965	0.0012483	.	.	0.3953833	0.0004893	.	.	yes
PACSIN2	.	.	.	.	.	.	.	.	0.6873653	1.35E-11	.	.	.
PADI1	.	.	.	.	.	.	.	.	0.5572603	2.51E-07	.	.	.
PADI3	.	.	0.375278	0.0009856	.	.	.	.	.	.	.	.	.

Gene	Atrial Expression	p-value Atrial	AV-Canal Expression	p-value AV-Canal	Branchial Arches Expression	p-value Branchial Arches	Heart Tube Expression	p-value Heart Tube	Outflow Tract Expression	p-value Outflow Tract	Ventricle Expression	p-value Ventricle	DMP Expression
PAF1	.	.	.	.	.	.	.	.	0.7382068	6.15E-14	.	.	.
PAFAH1B1	.	.	.	.	.	.	.	.	.	.	.	.	yes
PAFAH1B2	.	.	.	.	.	.	.	.	0.3851439	0.0007029	.	.	yes
PAG1	.	.	.	.	.	.	.	.	0.4908998	8.97E-06	.	.	.
PAGR1	.	.	.	.	.	.	.	.	0.7060648	2.12E-12	.	.	.
PAICS	.	.	.	.	0.3904478	0.0005835	.	.	.	.	.	.	yes
PAIP2	.	.	.	.	.	.	.	.	.	.	.	.	yes
PAK2	.	.	.	.	.	.	.	.	0.7626006	2.91E-15	.	.	.
PALM2	.	.	0.4052785	0.000341	.	.	.	.	0.6813293	2.38E-11	.	.	.
PALM2-AKAP2	.	.	0.3044231	0.0083626	.	.	.	.	0.6196011	3.96E-09	.	.	.
PAM16	.	.	.	.	.	.	.	.	0.5121159	3.10E-06	.	.	.
PANX1	.	.	.	.	0.5957259	2.16E-08	.	.	.	.	.	.	.
PAPOLA	.	.	0.3498109	0.0022498	.	.	.	.	0.3385791	0.0031709	.	.	yes
PAPOLB	.	.	.	.	0.5552149	2.84E-07	.	.	.	.	.	.	.
PAPSS1	.	.	.	.	.	.	.	.	0.6027158	1.33E-08	.	.	.
PAPSS2	.	.	.	.	.	.	.	.	0.4537719	4.90E-05	.	.	.
PAQR4	.	.	0.4771311	1.72E-05	.	.	.	.	0.5058604	4.27E-06	.	.	.
PARD6A	.	.	.	.	.	.	.	.	0.6369471	1.06E-09	.	.	.
PARD6B	.	.	0.5167879	2.43E-06	.	.	.	.	.	.	.	.	.
PARN	.	.	.	.	.	.	.	.	0.760991	3.60E-15	.	.	.
PARP1	.	.	.	.	0.323717	0.0049006	.	.	0.7388217	5.72E-14	.	.	yes
PARP2	.	.	.	.	0.5459038	4.89E-07	.	.	.	.	.	.	.
PARP3	.	.	.	.	.	.	.	.	0.5367915	8.21E-07	.	.	.
PARVB	.	.	0.4389686	9.14E-05	.	.	.	.	0.5908665	2.99E-08	.	.	.
PATE3	.	.	.	.	.	.	.	.	.	.	0.708257	1.69E-12	.
PAWR	.	.	.	.	.	.	0.3468148	0.0024685	0.7436515	3.21E-14	.	.	.
PAX2	.	.	0.4464482	6.69E-05	.	.	.	.	.	.	.	.	.
PBRM1	.	.	.	.	.	.	.	.	.	.	0.6874856	1.33E-11	yes
PBX1	.	.	0.4809664	1.44E-05	.	.	.	.	.	.	.	.	yes
PBX2	.	.	.	.	.	.	.	.	0.743859	3.13E-14	.	.	yes
PBXIP1	.	.	.	.	.	.	.	.	0.7717182	8.46E-16	.	.	.
PCBP1	.	.	.	.	.	.	.	.	0.3584903	0.001711	.	.	yes
PCBP2	.	.	.	.	.	.	.	.	0.5938863	2.44E-08	.	.	yes
PCBP4	.	.	0.3008296	0.0092034	.	.	.	.	.	.	.	.	yes
PCDHA12	.	.	.	.	.	.	.	.	.	.	0.5473402	4.50E-07	.
PCDHAC1	.	.	.	.	.	.	.	.	.	.	0.6226907	3.15E-09	.
PCDHGA3	.	.	.	.	0.4970523	6.64E-06	.	.	.	.	.	.	.
PCDHGA4	.	.	.	.	.	.	.	.	0.6175952	4.59E-09	.	.	.
PCDHGA7	.	.	0.4025147	0.0003776	.	.	.	.	.	.	.	.	.
PCDHGA8	.	.	0.51581	2.56E-06	.	.	.	.	.	.	.	.	.
PCDHGB4	.	.	.	.	.	.	.	.	0.5036423	4.78E-06	.	.	.
PCGF2	.	.	.	.	.	.	.	.	.	.	0.6696368	6.87E-11	.
PCGF5	.	.	0.4764212	1.78E-05	.	.	.	.	.	.	.	.	.
PCGF6	.	.	.	.	.	.	.	.	0.5155089	2.60E-06	.	.	.
PCID2	.	.	0.4510312	5.51E-05	.	.	.	.	.	.	.	.	.
PCM1	.	.	.	.	.	.	.	.	0.4514161	5.42E-05	.	.	yes
PCNP	.	.	0.3854352	0.0006958	.	.	.	.	0.3363648	0.003388	.	.	.
PCNX	.	.	0.5056849	4.31E-06	.	.	.	.	.	.	.	.	.
PCOLCE2	.	.	0.377585	0.0009116	.	.	.	.	.	.	.	.	.
PCSK7	.	.	.	.	.	.	.	.	0.655288	2.38E-10	.	.	.
PCTP	.	.	.	.	.	.	.	.	0.9241492	8.25E-32	.	.	.
PDAP1	.	.	.	.	0.4301485	0.0001307	.	.	.	.	.	.	yes
PDCD10	.	.	.	.	.	.	.	.	0.5783782	6.78E-08	.	.	.
PDCD11	.	.	.	.	.	.	.	.	0.6586309	1.79E-10	.	.	.
PDE1A	.	.	0.4927802	8.19E-06	.	.	.	.	.	.	.	.	.
PDE3A	.	.	.	.	.	.	.	.	0.5039295	4.71E-06	.	.	.
PDE6D	.	.	0.4602265	3.70E-05	.	.	.	.	0.4912767	8.81E-06	.	.	.
PDGFRA	.	.	.	.	.	.	.	.	.	.	.	.	yes
PDHB	.	.	0.3481033	0.0023722	.	.	.	.	0.7048469	2.40E-12	.	.	.
PDIA3	.	.	.	.	.	.	.	.	0.4951761	7.29E-06	.	.	yes
PDIA4	.	.	.	.	.	.	.	.	.	.	.	.	yes
PDIA6	.	.	.	.	.	.	.	.	0.5494938	3.97E-07	.	.	yes
PDK1	.	.	0.4593502	3.84E-05	.	.	.	.	.	.	.	.	yes
PDK3	.	.	0.4663232	2.82E-05	.	.	.	.	0.7123094	1.11E-12	.	.	.
PDK4	.	.	0.4943857	7.57E-06	.	.	.	.	0.3314811	0.003914	.	.	.
PDLIM1	.	.	.	.	.	.	.	.	0.7511212	1.28E-14	.	.	.
PDPN	.	.	.	.	.	.	.	.	0.7066828	1.99E-12	.	.	.
PDS5B	.	.	0.4901568	9.30E-06	.	.	.	.	.	.	.	.	yes
PDSS2	.	.	.	.	.	.	.	.	0.4410467	8.39E-05	.	.	.
PDZD2	.	.	.	.	.	.	.	.	0.7289714	1.79E-13	.	.	.
PDZD9	.	.	.	.	.	.	.	.	0.4813085	1.42E-05	.	.	.
PEA15	.	.	.	.	.	.	.	.	0.5543462	2.99E-07	.	.	.
PEAR1	.	.	.	.	.	.	.	.	.	.	0.4382379	9.42E-05	.
PEG10	.	.	.	.	.	.	.	.	0.5376982	7.81E-07	.	.	yes
PEG3	.	.	.	.	.	.	.	.	.	.	.	.	yes

Gene	Atrial Expression	p-value Atrial	AV-Canal Expression	p-value AV-Canal	Branchial Arches Expression	p-value Branchial Arches	Heart Tube Expression	p-value Heart Tube	Outflow Tract Expression	p-value Outflow Tract	Ventricle Expression	p-value Ventricle	DMP Expression
PELP1	.	.	.	.	.	.	.	.	0.4491035	5.98E-05	.	.	.
PER3	.	.	.	.	.	.	.	.	.	.	0.4852711	1.18E-05	.
PES1	.	.	0.3054373	0.0081378	.	.	.	.	0.5932659	2.55E-08	.	.	.
PEX2	.	.	.	.	.	.	.	.	0.4687395	2.53E-05	.	.	.
PEX6	.	.	.	.	.	.	.	.	0.49494	7.37E-06	.	.	.
PEX7	.	.	.	.	.	.	.	.	0.4499451	5.77E-05	.	.	.
PF4	.	.	0.4323826	0.0001195	.	.	.	.	.	.	.	.	.
PFAS	.	.	.	.	0.6115425	7.13E-09	.	.	0.3714684	0.0011199	.	.	.
PFKL	.	.	.	.	.	.	.	.	0.6880447	1.26E-11	.	.	yes
PFN1	.	.	.	.	.	.	.	.	.	.	.	.	yes
PGAM1	.	.	0.424251	0.0001652	.	.	.	.	0.5689306	1.23E-07	.	.	.
PGAM2	.	.	0.3448098	0.0026253	.	.	.	.	0.6897964	1.07E-11	.	.	.
PGAM5	.	.	0.4113906	0.0002713	.	.	.	.	.	.	.	.	.
PGD	.	.	.	.	.	.	.	.	0.3811085	0.0008081	.	.	yes
PGK1	.	.	0.3447137	0.002633	.	.	.	.	0.4542822	4.79E-05	.	.	.
PGM1	.	.	0.3004394	0.009299	.	.	0.3742422	0.0010206	0.5520899	3.41E-07	.	.	.
PGRMC1	.	.	.	.	.	.	.	.	.	.	.	.	yes
PHACTR1	.	.	.	.	.	.	.	.	.	.	0.5991839	1.70E-08	.
PHAX	.	.	.	.	.	.	.	.	0.5837001	4.81E-08	.	.	.
PHB	.	.	.	.	.	.	.	.	0.5087823	3.68E-06	.	.	.
PHB2	.	.	.	.	.	.	.	.	.	.	.	.	yes
PHC1	.	.	.	.	0.4658813	2.88E-05	.	.	.	.	.	.	.
PHC2	.	.	.	.	.	.	.	.	0.3201919	0.0054171	.	.	yes
PHF2	.	.	.	.	.	.	.	.	0.3495658	0.002267	0.4725575	2.13E-05	.
PHF20L1	.	.	.	.	.	.	.	.	.	.	0.685519	1.60E-11	.
PHF5A	.	.	.	.	.	.	.	.	0.5102666	3.41E-06	.	.	.
PHIP	.	.	.	.	.	.	.	.	.	.	.	.	yes
PHKA2	.	.	.	.	.	.	.	.	0.4716422	2.22E-05	.	.	.
PHLDA2	.	.	.	.	.	.	.	.	0.902758	4.32E-28	.	.	.
PHLDB1	.	.	.	.	.	.	.	.	.	.	0.6740787	4.62E-11	.
PHPT1	.	.	0.3994436	0.0004225	.	.	.	.	.	.	.	.	.
PHRF1	.	.	.	.	.	.	.	.	.	.	0.6942484	6.93E-12	yes
PHTF1	.	.	0.3874815	0.0006478	.	.	.	.	.	.	.	.	.
PI15	.	.	.	.	.	.	0.4393048	9.01E-05	.	.	.	.	.
PIANP	.	.	.	.	.	.	.	.	0.4813968	1.41E-05	.	.	.
PIAS1	.	.	.	.	.	.	.	.	0.4527881	5.11E-05	.	.	.
PIAS2	.	.	0.4422395	7.98E-05	.	.	.	.	.	.	.	.	.
PICALM	.	.	0.3932315	0.0005285	.	.	.	.	.	.	.	.	yes
PIEZO2	.	.	.	.	.	.	.	.	0.4976917	6.44E-06	.	.	.
PIK3C2A	.	.	.	.	.	.	.	.	0.6899173	1.05E-11	.	.	.
PILRA	.	.	.	.	.	.	0.5427495	5.86E-07	.	.	.	.	.
PIM1	.	.	.	.	.	.	.	.	0.5469116	4.62E-07	.	.	.
PIP4K2B	.	.	.	.	0.6245917	2.73E-09	.	.	.	.	.	.	.
PIP5K1A	.	.	0.4545027	4.75E-05	.	.	.	.	.	.	.	.	.
PITPNB	.	.	0.5162536	2.50E-06	.	.	.	.	.	.	.	.	.
PITPNM1	.	.	.	.	.	.	.	.	0.5995289	1.66E-08	.	.	.
PITPNM3	.	.	.	.	.	.	.	.	.	.	0.6067216	1.01E-08	.
PITRM1	.	.	.	.	.	.	.	.	0.3847493	0.0007126	.	.	yes
PITX2	.	.	0.4213643	0.000185	.	.	.	.	.	.	.	.	.
PIWIL4	.	.	.	.	.	.	0.3487806	0.002323	.	.	0.4619105	3.43E-05	.
PKD2L2	.	.	.	.	.	.	.	.	.	.	0.5387261	7.37E-07	.
PKDCC	.	.	.	.	.	.	.	.	.	.	.	.	yes
PKIG	.	.	.	.	.	.	.	.	0.4517839	5.33E-05	.	.	.
PKM	.	.	.	.	.	.	.	.	.	.	0.3982383	0.0004414	yes
PKP2	.	.	0.3114292	0.0069146	.	.	.	.	0.7556431	7.21E-15	.	.	.
PLA2G12A	.	.	0.5070483	4.02E-06	.	.	.	.	.	.	.	.	.
PLA2G5	.	.	.	.	.	.	.	.	0.5364736	8.36E-07	.	.	.
PLAGL1	.	.	0.4727688	2.11E-05	.	.	.	.	.	.	.	.	yes
PLAT	.	.	0.56396	1.67E-07	.	.	.	.	.	.	.	.	.
PLBD2	.	.	.	.	.	.	.	.	0.4560259	4.44E-05	.	.	.
PLCG1	.	.	.	.	.	.	.	.	.	.	.	.	yes
PLEK	.	.	0.4040164	0.0003573	.	.	.	.	.	.	.	.	.
PLEK2	.	.	0.4155813	0.0002313	.	.	.	.	.	.	.	.	.
PLEKHA1	.	.	.	.	.	.	.	.	0.7808606	2.31E-16	.	.	.
PLEKHA3	.	.	0.5549673	2.88E-07	.	.	.	.	.	.	.	.	.
PLEKHA7	.	.	.	.	.	.	.	.	.	.	0.5547631	2.91E-07	.
PLEKHD1	.	.	.	.	0.5275063	1.37E-06	.	.	.	.	.	.	.
PLEKHM3	.	.	0.408961	0.0002972	.	.	.	.	.	.	.	.	.
PLEKHO1	.	.	0.4317144	0.0001228	.	.	.	.	0.4132442	0.0002529	.	.	.
PLIN3	.	.	0.4096696	0.0002894	.	.	.	.	0.3705887	0.0011532	.	.	.
PLK4	.	.	0.5495951	3.95E-07	.	.	.	.	.	.	.	.	.
PLN	.	.	.	.	.	.	.	.	0.4926545	8.24E-06	0.4998844	5.77E-06	.
PLOD3	.	.	.	.	.	.	.	.	0.5432632	5.70E-07	.	.	.
PLSCR1	.	.	.	.	.	.	.	.	.	.	0.6937441	7.28E-12	.
PLVAP	.	.	0.3146822	0.0063208	.	.	.	.	0.7592586	4.52E-15	.	.	.

Gene	Atrial Expression	p-value Atrial	AV-Canal Expression	p-value AV-Canal	Branchial Arches Expression	p-value Branchial Arches	Heart Tube Expression	p-value Heart Tube	Outflow Tract Expression	p-value Outflow Tract	Ventricle Expression	p-value Ventricle	DMP Expression
PLXNA1	.	.	.	.	.	.	.	.	0.3979827	0.0004455	.	.	yes
PLXNA4	.	.	0.5242184	1.64E-06	.	.	.	.	0.2980589	0.0099012	.	.	.
PLXNB1	.	.	.	.	.	.	.	.	.	.	.	.	yes
PLXNB2	.	.	.	.	.	.	.	.	.	.	.	.	yes
PLXNC1	.	.	0.6874793	1.33E-11	.	.	.	.	.	.	.	.	.
PLXND1	.	.	0.3617964	0.0015384	.	.	.	.	0.7255271	2.64E-13	.	.	yes
PMM2	.	.	.	.	.	.	.	.	0.6679024	8.01E-11	.	.	.
PMS2	.	.	.	.	.	.	.	.	0.4702528	2.36E-05	.	.	.
PNCK	.	.	.	.	.	.	.	.	.	.	0.5734974	9.25E-08	.
PNISR	.	.	.	.	.	.	.	.	.	.	.	.	yes
PNKD	.	.	.	.	.	.	.	.	0.4653132	2.95E-05	.	.	.
PNMT	.	.	0.5677906	1.32E-07	.	.	.	.	.	.	.	.	.
PNN	.	.	.	.	.	.	.	.	.	.	.	.	yes
PODXL	.	.	0.5904833	3.07E-08	.	.	.	.	0.3372888	0.0032959	.	.	yes
POFUT2	.	.	.	.	.	.	.	.	0.6277102	2.16E-09	.	.	.
POLB	.	.	.	.	.	.	.	.	0.5058786	4.27E-06	.	.	.
POLD1	.	.	.	.	0.6259577	2.46E-09	.	.	.	.	.	.	.
POLDIP3	.	.	.	.	.	.	.	.	.	.	.	.	yes
POLE3	.	.	0.3762599	0.0009534	.	.	.	.	0.3636607	0.0014482	.	.	.
POLL	.	.	.	.	0.4685417	2.55E-05	.	.	0.3480286	0.0023777	.	.	.
POLR1C	.	.	.	.	.	.	.	.	0.4600599	3.72E-05	.	.	.
POLR1D	.	.	0.456663	4.32E-05	.	.	.	.	.	.	.	.	.
POLR1E	.	.	.	.	.	.	.	.	0.5681101	1.30E-07	.	.	.
POLR2A	.	.	.	.	.	.	.	.	0.5782724	6.83E-08	.	.	yes
POLR2B	.	.	.	.	.	.	.	.	.	.	.	.	yes
POLR2C	.	.	0.3302478	0.0040579	.	.	.	.	0.5390006	7.26E-07	.	.	.
POLR2D	.	.	0.3976545	0.0004508	.	.	.	.	.	.	.	.	.
POLR2E	.	.	.	.	.	.	.	.	0.5773972	7.22E-08	.	.	.
POLR2F	.	.	0.3291278	0.0041925	.	.	.	.	0.6280586	2.10E-09	.	.	.
POLR2M	.	.	.	.	.	.	.	.	0.4751347	1.89E-05	.	.	yes
POM121L2	.	.	.	.	.	.	.	.	.	.	0.4840246	1.25E-05	.
POMT2	.	.	.	.	.	.	.	.	0.4492894	5.93E-05	.	.	.
PON2	.	.	.	.	.	.	.	.	0.5407211	6.58E-07	.	.	.
POPDC2	.	.	.	.	.	.	.	.	0.6728481	5.16E-11	.	.	.
PPARD	.	.	.	.	.	.	.	.	0.6978581	4.85E-12	.	.	.
PPHLN1	.	.	0.5719839	1.02E-07	.	.	.	.	.	.	.	.	.
PPIA	.	.	.	.	.	.	.	.	0.523812	1.67E-06	.	.	.
PPIB	.	.	.	.	.	.	.	.	0.612541	6.64E-09	.	.	.
PPIC	.	.	0.4232487	0.0001719	.	.	.	.	.	.	.	.	.
PPID	.	.	0.4428122	7.79E-05	.	.	.	.	.	.	.	.	.
PPIE	.	.	.	.	0.4948762	7.39E-06	.	.	.	.	.	.	.
PPM1E	.	.	.	.	.	.	.	.	.	.	0.5113509	3.23E-06	.
PPM1G	.	.	.	.	.	.	.	.	0.841844	5.76E-21	.	.	yes
PPM1J	.	.	0.3616884	0.0015438	.	.	.	.	0.6840284	1.85E-11	.	.	.
PPM1K	.	.	0.3500313	0.0022344	.	.	.	.	0.4805882	1.47E-05	.	.	.
PPME1	.	.	.	.	.	.	.	.	0.5884138	3.52E-08	.	.	.
PPOX	0.5158418	2.56E-06	.	.	.	.	.	.	.	.	.	.	.
PPP1CA	.	.	0.3563606	0.0018312	.	.	.	.	.	.	.	.	yes
PPP1CB	.	.	0.3273228	0.0044179	.	.	.	.	0.6070118	9.85E-09	.	.	yes
PPP1R13B	.	.	.	.	.	.	.	.	0.5132553	2.92E-06	.	.	.
PPP1R14B	.	.	.	.	.	.	.	.	0.7494672	1.57E-14	.	.	.
PPP1R14C	.	.	0.5543285	2.99E-07	.	.	.	.	.	.	.	.	.
PPP1R15A	.	.	.	.	.	.	.	.	0.4446745	7.21E-05	.	.	.
PPP1R15B	.	.	.	.	.	.	.	.	0.6663357	9.20E-11	.	.	.
PPP1R17	.	.	0.6532535	2.82E-10	.	.	0.3069402	0.0078144	.	.	.	.	.
PPP1R37	.	.	0.3248325	0.0047465	.	.	.	.	0.6102515	7.82E-09	.	.	.
PPP1R3D	.	.	.	.	.	.	.	.	0.64523	5.45E-10	.	.	.
PPP1R9A	.	.	0.4067171	0.0003232	.	.	.	.	.	.	.	.	.
PPP2CA	.	.	0.3146596	0.0063248	.	.	.	.	0.5282614	1.32E-06	.	.	yes
PPP2R1A	.	.	.	.	.	.	.	.	.	.	.	.	yes
PPP2R2D	.	.	.	.	.	.	.	.	0.5202112	2.03E-06	.	.	.
PPP2R3A	.	.	0.4487381	6.07E-05	.	.	.	.	.	.	.	.	.
PPP2R4	.	.	.	.	.	.	.	.	.	.	.	.	yes
PPP2R5A	.	.	.	.	.	.	.	.	0.5167753	2.43E-06	.	.	.
PPP2R5D	.	.	.	.	.	.	.	.	.	.	.	.	yes
PPP4C	.	.	.	.	.	.	.	.	.	.	.	.	yes
PPP6R1	.	.	.	.	.	.	.	.	0.5563759	2.65E-07	.	.	.
PPP6R3	.	.	.	.	.	.	.	.	0.4793093	1.56E-05	0.5031632	4.90E-06	yes
PPTC7	.	.	.	.	.	.	.	.	0.6937521	7.27E-12	.	.	.
PRAM1	.	.	.	.	.	.	.	.	0.6359715	1.14E-09	.	.	.
PRDM6	.	.	0.4099765	0.0002861	.	.	.	.	.	.	.	.	.
PRDX1	.	.	.	.	.	.	.	.	0.6443591	5.85E-10	.	.	.
PRDX2	.	.	.	.	.	.	.	.	0.5390396	7.24E-07	.	.	yes
PRDX6	.	.	.	.	.	.	.	.	0.5448311	5.21E-07	.	.	yes
PRG3	.	.	.	.	.	.	.	.	0.676925	3.56E-11	.	.	.

Gene	Atrial Expression	p-value Atrial	AV-Canal Expression	p-value AV-Canal	Branchial Arches Expression	p-value Branchial Arches	Heart Tube Expression	p-value Heart Tube	Outflow Tract Expression	p-value Outflow Tract	Ventricle Expression	p-value Ventricle	DMP Expression
PRKAA2	.	.	0.390303	0.0005865	.	.	.	.	0.4841075	1.24E-05	.	.	.
PRKAB2	.	.	.	.	.	.	.	.	0.6747477	4.34E-11	.	.	.
PRKACA	.	.	0.4611133	3.56E-05	.	.	.	.	0.3854066	0.0006965	.	.	.
PRKAR1A	.	.	.	.	.	.	.	.	0.3515969	0.0021279	.	.	yes
PRKCDBP	.	.	.	.	.	.	.	.	0.7913163	4.85E-17	.	.	.
PRKCH	.	.	.	.	.	.	.	.	0.483598	1.27E-05	.	.	.
PRKCI	.	.	.	.	.	.	.	.	0.4936312	7.86E-06	.	.	.
PRKCSH	.	.	.	.	0.3670065	0.0012981	.	.	.	.	.	.	yes
PRKD3	.	.	.	.	.	.	.	.	.	.	.	.	yes
PRKDC	.	.	.	.	.	.	.	.	.	.	0.5446428	5.26E-07	.
PRMT1	.	.	.	.	.	.	.	.	.	.	.	.	yes
PRMT5	.	.	.	.	.	.	.	.	.	.	.	.	yes
PRMT6	.	.	0.4780986	1.65E-05	.	.	.	.	0.5811932	5.66E-08	.	.	.
PRMT9	.	.	.	.	.	.	.	.	.	.	0.6286189	2.01E-09	.
PRND	.	.	.	.	.	.	.	.	0.4957373	7.09E-06	.	.	.
PROSER1	.	.	.	.	.	.	.	.	.	.	0.5411802	6.41E-07	.
PRPF18	.	.	.	.	0.4473183	6.45E-05	.	.	.	.	.	.	.
PRPF19	.	.	.	.	0.3139182	0.0064561	.	.	.	.	.	.	yes
PRPF38B	.	.	.	.	.	.	.	.	0.469607	2.43E-05	.	.	.
PRPF40A	.	.	.	.	.	.	.	.	0.5658767	1.49E-07	.	.	yes
PRPF4B	.	.	0.375283	0.0009854	.	.	.	.	.	.	.	.	yes
PRPF8	.	.	.	.	.	.	.	.	0.6899118	1.06E-11	.	.	yes
PRPS2	.	.	.	.	.	.	.	.	0.5229203	1.76E-06	.	.	.
PRR5L	0.5343595	9.41E-07	.	.	.	.	.	.	.	.	.	.	.
PRRC2A	.	.	.	.	.	.	.	.	0.5582233	2.37E-07	.	.	yes
PRRC2B	.	.	0.3090947	0.0073705	.	.	.	.	.	.	.	.	yes
PRRC2C	.	.	.	.	.	.	.	.	.	.	.	.	yes
PRRT4	.	.	.	.	.	.	.	.	0.6148877	5.60E-09	.	.	.
PRRX2	.	.	0.5129809	2.97E-06	.	.	.	.	.	.	.	.	.
PRSS36	.	.	0.3846651	0.0007147	.	.	.	.	.	.	.	.	.
PRTG	.	.	.	.	.	.	0.3416016	0.0028948	0.4283956	0.0001402	.	.	yes
PRUNE	.	.	.	.	.	.	.	.	0.4780234	1.65E-05	.	.	.
PRX	.	.	.	.	.	.	.	.	.	.	0.6321192	1.54E-09	.
PSAP	.	.	.	.	.	.	.	.	.	.	.	.	yes
PSAT1	.	.	.	.	0.4297357	0.0001329	.	.	.	.	.	.	yes
PSEN1	.	.	.	.	.	.	.	.	0.5748881	8.47E-08	.	.	.
PSIP1	.	.	.	.	.	.	.	.	.	.	.	.	yes
PSKH1	.	.	.	.	.	.	.	.	.	.	0.7192524	5.26E-13	.
PSMA1	.	.	.	.	.	.	.	.	0.4613337	3.52E-05	.	.	yes
PSMA6	.	.	.	.	.	.	.	.	0.501435	5.34E-06	.	.	yes
PSMA7	.	.	.	.	.	.	.	.	.	.	.	.	yes
PSMB3	.	.	.	.	.	.	.	.	0.461537	3.49E-05	.	.	.
PSMB4	.	.	0.3628467	0.001487	.	.	.	.	.	.	.	.	yes
PSMB7	.	.	.	.	.	.	.	.	0.464102	3.11E-05	.	.	yes
PSMC2	.	.	0.3202563	0.0054072	.	.	.	.	0.6419566	7.09E-10	.	.	yes
PSMC3	.	.	.	.	.	.	.	.	0.3069254	0.0078175	.	.	yes
PSMC3IP	.	.	.	.	.	.	.	.	0.4731332	2.07E-05	.	.	.
PSMC4	.	.	.	.	.	.	.	.	0.3030576	0.0086738	.	.	yes
PSMC5	.	.	.	.	0.4317168	0.0001228	.	.	0.4397758	8.84E-05	.	.	yes
PSMC6	.	.	.	.	.	.	.	.	.	.	.	.	yes
PSMD1	.	.	0.5425221	5.94E-07	.	.	.	.	0.4093633	0.0002928	.	.	yes
PSMD10	.	.	.	.	.	.	.	.	0.4616275	3.48E-05	.	.	.
PSMD12	.	.	0.3012213	0.0091083	.	.	.	.	0.5220983	1.84E-06	.	.	.
PSMD2	.	.	.	.	0.3625547	0.0015011	.	.	.	.	.	.	yes
PSMD3	.	.	.	.	.	.	.	.	0.5126092	3.02E-06	.	.	yes
PSMD4	.	.	.	.	.	.	.	.	0.6832825	1.98E-11	.	.	yes
PSMD7	.	.	.	.	.	.	.	.	0.5234265	1.71E-06	.	.	yes
PSMD8	.	.	0.4079351	0.0003089	.	.	.	.	0.368195	0.0012483	.	.	.
PSMD9	.	.	.	.	.	.	.	.	0.5802214	6.02E-08	.	.	.
PSME3	.	.	.	.	.	.	.	.	.	.	.	.	yes
PSME4	.	.	0.3133182	0.0065641	.	.	.	.	.	.	.	.	yes
PSMG2	.	.	.	.	.	.	.	.	0.5089474	3.65E-06	.	.	.
PTBP1	.	.	.	.	.	.	.	.	.	.	.	.	yes
PTBP2	.	.	.	.	0.4663723	2.81E-05	.	.	.	.	.	.	yes
PTCD2	.	.	0.3946562	0.0005023	.	.	.	.	0.4785017	1.62E-05	.	.	.
PTCHD2	.	.	.	.	.	.	.	.	0.4995337	5.87E-06	.	.	.
PTEN	.	.	.	.	.	.	.	.	0.5661922	1.46E-07	.	.	.
PTGES	.	.	.	.	.	.	.	.	0.8190498	4.81E-19	.	.	.
PTGES2	.	.	.	.	.	.	.	.	0.6271254	2.26E-09	.	.	.
PTGFRN	.	.	.	.	.	.	.	.	0.6251104	2.63E-09	.	.	.
PTHLH	.	.	0.3953029	0.0004907	.	.	.	.	.	.	.	.	.
PTK2	.	.	.	.	.	.	.	.	.	.	0.6417551	7.21E-10	.
PTK7	.	.	0.3558997	0.0018581	.	.	.	.	.	.	.	.	yes
PTMS	.	.	0.3821527	0.0007796	.	.	.	.	0.5344264	9.37E-07	.	.	yes
PTN	.	.	.	.	.	.	.	.	.	.	.	.	yes

Gene	Atrial Expression	p-value Atrial	AV-Canal Expression	p-value AV-Canal	Branchial Arches Expression	p-value Branchial Arches	Heart Tube Expression	p-value Heart Tube	Outflow Tract Expression	p-value Outflow Tract	Ventricle Expression	p-value Ventricle	DMP Expression
PTOV1	.	.	.	.	.	.	.	.	.	.	.	.	yes
PTP4A2	.	.	.	.	.	.	0.4745426	1.94E-05	.	.	.	.	yes
PTP4A3	.	.	0.572022	1.02E-07	.	.	.	.	.	.	.	.	.
PTPN1	.	.	.	.	.	.	.	.	0.7505673	1.37E-14	.	.	.
PTPN11	.	.	.	.	.	.	0.449512	5.88E-05	.	.	.	.	yes
PTPN13	.	.	.	.	.	.	.	.	.	.	.	.	yes
PTPN4	.	.	.	.	.	.	0.3949411	0.0004972	0.468162	2.60E-05	.	.	.
PTPN9	.	.	0.4201568	0.0001939	.	.	.	.	.	.	.	.	.
PTPRB	.	.	0.4171449	0.0002178	.	.	.	.	0.3523957	0.0020753	.	.	.
PTPRE	.	.	.	.	.	.	.	.	.	.	0.4508616	5.55E-05	.
PTPRF	.	.	.	.	.	.	.	.	0.8534618	4.57E-22	.	.	yes
PTPRR	.	.	0.4132517	0.0002528	.	.	.	.	.	.	.	.	.
PTPRS	.	.	.	.	.	.	.	.	0.3219391	0.0051554	.	.	yes
PTPRT	.	.	.	.	.	.	.	.	.	.	0.504909	4.49E-06	.
PTRF	.	.	0.3364805	0.0033763	.	.	.	.	0.6234461	2.98E-09	.	.	.
PTS	.	.	0.491832	8.58E-06	.	.	.	.	.	.	.	.	.
PTTG1IP	.	.	0.4157743	0.0002296	.	.	.	.	0.5543526	2.99E-07	.	.	.
PUF60	.	.	.	.	.	.	.	.	.	.	.	.	yes
PUM1	.	.	.	.	.	.	.	.	.	.	.	.	yes
PUM2	.	.	.	.	.	.	.	.	0.6083476	8.96E-09	.	.	yes
PURB	.	.	0.4104436	0.0002811	.	.	.	.	0.6799817	2.69E-11	.	.	yes
PUS3	.	.	.	.	.	.	0.5005246	5.59E-06	.	.	.	.	.
PVRL1	.	.	0.3823028	0.0007756	.	.	.	.	.	.	.	.	.
PVRL2	.	.	.	.	.	.	.	.	0.6630339	1.23E-10	.	.	.
PWWP2A	.	.	0.4108002	0.0002774	.	.	.	.	.	.	.	.	.
PXDN	.	.	0.3009063	0.0091847	.	.	.	.	0.3716013	0.001115	.	.	yes
PYCR2	.	.	.	.	0.5427258	5.87E-07	.	.	.	.	.	.	.
PYGM	.	.	.	.	.	.	.	.	0.6625329	1.28E-10	.	.	.
PYGO2	.	.	.	.	.	.	.	.	0.5151504	2.65E-06	.	.	.
QARS	.	.	.	.	.	.	.	.	.	.	.	.	yes
QKI	.	.	0.4621762	3.39E-05	.	.	.	.	0.3455831	0.0025638	.	.	yes
QPCT	.	.	.	.	.	.	.	.	.	.	0.5018116	5.24E-06	.
QRICH1	.	.	.	.	.	.	.	.	0.7437758	3.16E-14	.	.	yes
QTRTD1	.	.	0.3768058	0.000936	.	.	.	.	.	.	.	.	.
RAB10	.	.	0.3986034	0.0004356	.	.	.	.	0.4175134	0.0002148	.	.	.
RAB14	.	.	0.3360266	0.0034223	.	.	.	.	0.5711575	1.07E-07	.	.	.
RAB1A	.	.	0.4130231	0.000255	.	.	.	.	0.4106525	0.0002789	.	.	yes
RAB1B	0.308819	0.007426	.	.	.	.	.	.	.	.	.	.	yes
RAB22A	.	.	0.3109659	0.0070031	.	.	.	.	0.5299983	1.20E-06	.	.	.
RAB28	.	.	0.4039326	0.0003584	.	.	.	.	.	.	.	.	.
RAB2A	.	.	0.5211229	1.93E-06	.	.	.	.	0.3000392	0.0093979	.	.	.
RAB31	.	.	0.428718	0.0001384	.	.	.	.	.	.	.	.	.
RAB36	.	.	.	.	.	.	.	.	0.4731448	2.07E-05	.	.	.
RAB3D	.	.	.	.	.	.	.	.	0.5419872	6.13E-07	.	.	.
RAB3GAP2	.	.	0.3779279	0.000901	.	.	.	.	.	.	.	.	.
RAB7A	.	.	0.409952	0.0002864	.	.	.	.	0.4107997	0.0002774	.	.	.
RABAC1	.	.	.	.	.	.	.	.	0.539111	7.21E-07	.	.	.
RABEP1	.	.	.	.	.	.	.	.	0.6717049	5.71E-11	.	.	.
RABGGTB	.	.	.	.	.	.	0.4729483	2.09E-05	.	.	.	.	.
RABIF	0.489121	9.78E-06	.	.	.	.	.	.	.	.	.	.	.
RABL6	.	.	.	.	.	.	.	.	0.6696981	6.83E-11	.	.	.
RAC1	.	.	.	.	.	.	.	.	.	.	.	.	yes
RAD21	.	.	.	.	.	.	.	.	.	.	.	.	yes
RAD23A	.	.	0.4593383	3.84E-05	.	.	.	.	.	.	.	.	.
RAD23B	.	.	0.4683606	2.57E-05	.	.	.	.	0.5421526	6.07E-07	.	.	yes
RAD51AP1	.	.	.	.	.	.	.	.	.	.	0.6708455	6.17E-11	.
RAD54L	.	.	.	.	.	.	.	.	.	.	0.5924157	2.70E-08	.
RAD9A	.	.	.	.	.	.	.	.	0.4549503	4.65E-05	.	.	.
RADIL	.	.	.	.	.	.	.	.	.	.	0.5671648	1.37E-07	.
RAE1	.	.	.	.	.	.	.	.	0.622874	3.11E-09	.	.	.
RAF1	.	.	0.3074776	0.0077015	0.3017442	0.0089827	.	.	.	.	.	.	yes
RAI1	.	.	0.5054222	4.37E-06	.	.	.	.	.	.	.	.	.
RALA	.	.	0.5379609	7.69E-07	.	.	.	.	0.527578	1.37E-06	.	.	.
RALB	.	.	0.3237219	0.0048999	.	.	.	.	0.4494638	5.89E-05	.	.	.
RALY	.	.	.	.	.	.	.	.	0.4791239	1.57E-05	.	.	yes
RALYL	.	.	.	.	.	.	.	.	.	.	0.4762587	1.79E-05	.
RAMP2	.	.	0.5203689	2.01E-06	.	.	.	.	0.5277307	1.35E-06	.	.	.
RAN	.	.	.	.	.	.	.	.	0.7255379	2.63E-13	.	.	yes
RANBP1	.	.	.	.	.	.	.	.	.	.	.	.	yes
RANBP2	.	.	.	.	.	.	.	.	.	.	.	.	yes
RANGAP1	.	.	.	.	.	.	.	.	.	.	.	.	yes
RAP1B	.	.	0.4781895	1.64E-05	.	.	.	.	0.5958869	2.13E-08	.	.	.
RAP1GAP	.	.	.	.	.	.	.	.	.	.	0.6657698	9.66E-11	.
RAP2C	.	.	0.3285403	0.0042647	.	.	.	.	0.5995004	1.66E-08	.	.	.
RAPGEF1	.	.	0.3695818	0.0011924	.	.	.	.	.	.	.	.	yes

Gene	Atrial Expression	p-value Atrial	AV-Canal Expression	p-value AV-Canal	Branchial Arches Expression	p-value Branchial Arches	Heart Tube Expression	p-value Heart Tube	Outflow Tract Expression	p-value Outflow Tract	Ventricle Expression	p-value Ventricle	DMP Expression
RAPGEF2	.	.	.	.	.	.	.	.	.	.	0.5931018	2.57E-08	.
RAPGEF3	.	.	0.3790259	0.0008679	.	.	.	.	.	.	.	.	.
RARA	.	.	.	.	0.356147	0.0018436	.	.	0.5492382	4.03E-07	.	.	.
RARB	.	.	0.3627552	0.0014914	.	.	.	.	0.5656466	1.51E-07	.	.	.
RASD1	.	.	.	.	.	.	0.5838226	4.77E-08	.	.	.	.	.
RASEF	.	.	.	.	.	.	.	.	.	.	0.5508642	3.67E-07	.
RASIP1	.	.	0.4776251	1.68E-05	.	.	.	.	.	.	.	.	.
RASL10B	.	.	0.5460625	4.85E-07	.	.	.	.	0.4718174	2.20E-05	.	.	.
RASL11B	.	.	0.4145586	0.0002405	.	.	.	.	0.7340636	9.99E-14	.	.	.
RASSF3	.	.	0.6651503	1.02E-10	.	.	.	.	.	.	.	.	.
RASSF5	.	.	.	.	.	.	.	.	0.8895709	3.32E-26	.	.	.
RB1CC1	.	.	0.4814059	1.41E-05	.	.	.	.	.	.	.	.	.
RBBP4	.	.	0.3059871	0.0080181	0.355287	0.0018946	.	.	.	.	.	.	yes
RBBP6	.	.	.	.	.	.	.	.	.	.	.	.	yes
RBBP7	.	.	0.5015763	5.30E-06	.	.	.	.	.	.	.	.	yes
RBFA	.	.	.	.	.	.	.	.	0.5827436	5.11E-08	.	.	.
RBFOX2	.	.	.	.	0.4601471	3.71E-05	.	.	.	.	.	.	yes
RBM12	.	.	.	.	.	.	.	.	0.6556867	2.30E-10	.	.	.
RBM20	.	.	0.437945	9.53E-05	.	.	.	.	.	.	.	.	.
RBM22	.	.	.	.	.	.	.	.	0.7281967	1.95E-13	.	.	.
RBM24	.	.	0.4587817	3.94E-05	.	.	.	.	0.6988374	4.40E-12	.	.	.
RBM25	.	.	.	.	.	.	.	.	.	.	.	.	yes
RBM27	.	.	.	.	.	.	.	.	0.6121114	6.84E-09	.	.	.
RBM28	.	.	0.5100288	3.45E-06	.	.	.	.	.	.	.	.	.
RBM39	.	.	.	.	.	.	.	.	.	.	.	.	yes
RBM6	.	.	.	.	.	.	.	.	0.5842503	4.64E-08	.	.	.
RBM8A	.	.	0.409956	0.0002863	.	.	.	.	.	.	.	.	.
RBMS2	.	.	0.3050328	0.0082268	.	.	.	.	0.5116311	3.18E-06	.	.	.
RBMXL1	.	.	0.3809552	0.0008124	.	.	.	.	.	.	.	.	.
RBPMS	.	.	0.3841113	0.0007286	.	.	.	.	.	.	.	.	.
RBPMS2	.	.	0.3867539	0.0006645	.	.	.	.	.	.	.	.	.
RCAN3	.	.	.	.	.	.	.	.	0.47582	1.83E-05	.	.	.
RCBTB2	.	.	0.3020168	0.0089178	.	.	.	.	.	.	.	.	yes
RCC2	.	.	.	.	.	.	.	.	.	.	.	.	yes
RCN1	.	.	0.5519704	3.44E-07	.	.	.	.	0.4967963	6.73E-06	.	.	yes
RDX	.	.	.	.	.	.	.	.	.	.	.	.	yes
RELA	.	.	.	.	.	.	.	.	0.444831	7.16E-05	.	.	.
RELL1	.	.	.	.	.	.	.	.	0.6413717	7.43E-10	.	.	.
RELT	.	.	.	.	.	.	.	.	0.5342273	9.48E-07	.	.	.
REPS1	.	.	0.382475	0.000771	.	.	.	.	.	.	.	.	.
RER1	.	.	0.404927	0.0003455	.	.	.	.	.	.	.	.	.
RERE	.	.	.	.	.	.	.	.	0.4605875	3.64E-05	.	.	yes
RETSAT	.	.	0.4138475	0.0002471	.	.	.	.	.	.	.	.	.
RFC1	.	.	.	.	.	.	.	.	0.3390897	0.0031227	.	.	yes
RFESD	.	.	.	.	.	.	.	.	0.5941267	2.40E-08	.	.	.
RFK	.	.	0.320326	0.0053966	.	.	.	.	0.5396498	6.99E-07	.	.	.
RFWD3	.	.	.	.	.	.	.	.	0.5890314	3.38E-08	.	.	.
RGP1	.	.	.	.	.	.	.	.	0.5165359	2.46E-06	.	.	.
RGS5	.	.	.	.	.	.	.	.	0.5740987	8.91E-08	.	.	.
RGS6	.	.	0.4096421	0.0002897	.	.	.	.	.	.	.	.	.
RGSL1	.	.	.	.	.	.	.	.	.	.	0.6981223	4.73E-12	.
RHBDD2	.	.	.	.	.	.	.	.	0.4878426	1.04E-05	.	.	.
RHO	.	.	.	.	.	.	.	.	.	.	0.6052608	1.11E-08	.
RHOB	.	.	0.4643095	3.09E-05	.	.	.	.	.	.	.	.	.
RHOBTB3	.	.	.	.	.	.	.	.	.	.	.	.	yes
RHOD	.	.	0.3480964	0.0023727	.	.	.	.	0.5222305	1.82E-06	.	.	.
RHOT1	.	.	0.4211966	0.0001862	.	.	.	.	.	.	.	.	.
RHOU	.	.	0.5244269	1.62E-06	.	.	.	.	0.5829279	5.05E-08	.	.	.
RIC8A	.	.	0.3357551	0.00345	.	.	.	.	0.6032866	1.28E-08	.	.	.
RIC8B	.	.	.	.	.	.	.	.	.	.	0.447712	6.34E-05	.
RIMKLB	.	.	.	.	.	.	.	.	.	.	0.5624233	1.84E-07	.
RIN1	.	.	.	.	.	.	0.5010392	5.45E-06	.	.	.	.	.
RIPK2	.	.	0.4118156	0.0002669	.	.	.	.	.	.	.	.	.
RIPPLY3	.	.	.	.	.	.	.	.	0.6132004	6.33E-09	.	.	.
RLF	.	.	.	.	.	.	.	.	0.4756185	1.85E-05	.	.	.
RLIM	.	.	.	.	.	.	.	.	.	.	.	.	yes
RMDN3	.	.	.	.	.	.	.	.	0.489978	9.38E-06	.	.	.
RNASE2	.	.	.	.	.	.	.	.	.	.	0.7645011	2.26E-15	.
RNF10	.	.	.	.	.	.	.	.	0.6318061	1.58E-09	.	.	yes
RNF112	0.5036805	4.77E-06	.	.	.	.	.	.	.	.	.	.	.
RNF114	.	.	.	.	.	.	.	.	0.6030914	1.30E-08	.	.	.
RNF139	.	.	.	.	.	.	0.5861707	4.09E-08	.	.	.	.	.
RNF145	.	.	.	.	.	.	.	.	0.4420609	8.04E-05	.	.	yes
RNF149	.	.	.	.	.	.	.	.	0.4881779	1.02E-05	.	.	.
RNF168	.	.	0.3966686	0.0004672	.	.	.	.	0.3058805	0.0080412	.	.	.

Gene	Atrial Expression	p-value Atrial	AV-Canal Expression	p-value AV-Canal	Branchial Arches Expression	p-value Branchial Arches	Heart Tube Expression	p-value Heart Tube	Outflow Tract Expression	p-value Outflow Tract	Ventricle Expression	p-value Ventricle	DMP Expression
RNF17	.	.	.	.	.	.	.	.	.	.	0.6777365	3.31E-11	.
RNF170	.	.	.	.	.	.	.	.	0.4399737	8.77E-05	.	.	.
RNF187	.	.	0.3051877	0.0081926	.	.	.	.	0.7481152	1.86E-14	.	.	yes
RNF207	.	.	0.6856473	1.58E-11	.	.	.	.	.	.	.	.	.
RNF219	.	.	.	.	0.4514173	5.42E-05	.	.	.	.	.	.	.
RNF25	.	.	0.4609191	3.59E-05	.	.	.	.	.	.	.	.	.
RNF44	.	.	.	.	.	.	.	.	0.4065533	0.0003252	.	.	yes
RNF5	.	.	0.4875031	1.06E-05	.	.	.	.	0.4917421	8.61E-06	.	.	.
RNMT	.	.	.	.	.	.	.	.	0.6015112	1.45E-08	.	.	.
RNMTL1	.	.	.	.	.	.	.	.	0.4585968	3.97E-05	.	.	.
ROCK2	.	.	.	.	.	.	.	.	0.548609	4.18E-07	.	.	.
RP11-503N18.1	.	.	.	.	.	.	.	.	.	.	0.596642	2.02E-08	.
RPA1	.	.	.	.	.	.	.	.	0.3820277	0.000783	.	.	yes
RPAP3	.	.	0.3240164	0.0048588	.	.	.	.	0.4746735	1.93E-05	.	.	.
RPF2	.	.	.	.	.	.	.	.	0.6824295	2.14E-11	.	.	.
RPGR	.	.	0.4281228	0.0001418	.	.	.	.	.	.	.	.	.
RPL13A	.	.	.	.	.	.	.	.	.	.	.	.	yes
RPL22	.	.	.	.	.	.	.	.	.	.	.	.	yes
RPL32	.	.	.	.	.	.	.	.	.	.	.	.	yes
RPL36A-HNRNPH2	.	.	.	.	.	.	.	.	0.5001735	5.69E-06	.	.	.
RPL37A	.	.	.	.	.	.	.	.	0.503503	4.82E-06	.	.	.
RPL3L	.	.	.	.	.	.	.	.	.	.	0.560284	2.09E-07	.
RPL4	.	.	.	.	.	.	.	.	.	.	.	.	yes
RPL7	.	.	.	.	.	.	.	.	0.6219135	3.34E-09	.	.	.
RPL7A	.	.	.	.	.	.	.	.	0.5567511	2.59E-07	.	.	.
RPL7L1	.	.	0.3276845	0.0043719	.	.	.	.	0.4863242	1.12E-05	.	.	.
RPL8	.	.	.	.	.	.	.	.	.	.	.	.	yes
RPLP0	.	.	.	.	.	.	.	.	0.3890254	0.0006136	.	.	yes
RPLP1	.	.	.	.	.	.	.	.	0.3510894	0.0021619	.	.	yes
RPN1	.	.	.	.	.	.	.	.	0.6490688	3.98E-10	.	.	yes
RPN2	.	.	.	.	.	.	.	.	0.5461062	4.84E-07	.	.	yes
RPP25L	.	.	.	.	.	.	0.5528353	3.27E-07	.	.	.	.	.
RPRD2	.	.	.	.	.	.	.	.	0.8906133	2.40E-26	.	.	.
RPS10-NUDT3	.	.	.	.	.	.	.	.	0.6593752	1.68E-10	.	.	.
RPS11	.	.	.	.	.	.	.	.	0.7340505	1.00E-13	.	.	yes
RPS12	.	.	0.6136279	6.13E-09	.	.	.	.	.	.	.	.	.
RPS14	.	.	.	.	.	.	.	.	.	.	.	.	yes
RPS15	.	.	0.4529107	5.08E-05	.	.	.	.	.	.	.	.	yes
RPS19	.	.	.	.	.	.	.	.	0.6178985	4.49E-09	.	.	.
RPS25	.	.	0.4131973	0.0002533	.	.	.	.	.	.	.	.	.
RPS26	.	.	.	.	.	.	.	.	.	.	.	.	yes
RPS27	.	.	.	.	.	.	.	.	0.4425611	7.87E-05	.	.	.
RPS3	.	.	.	.	.	.	.	.	.	.	.	.	yes
RPS5	.	.	.	.	.	.	.	.	.	.	.	.	yes
RPS9	.	.	.	.	.	.	.	.	.	.	.	.	yes
RPTOR	.	.	.	.	.	.	.	.	.	.	0.5050095	4.46E-06	.
RRAD	.	.	0.7478783	1.91E-14	.	.	.	.	.	.	.	.	.
RRAGD	.	.	.	.	.	.	.	.	0.5464521	4.74E-07	.	.	.
RRAS	.	.	0.4824415	1.34E-05	.	.	.	.	.	.	.	.	.
RRAS2	.	.	0.3958258	0.0004816	.	.	.	.	0.4867173	1.10E-05	.	.	.
RRM1	.	.	0.3860355	0.0006814	.	.	.	.	0.3345181	0.003579	.	.	yes
RRM2	.	.	.	.	0.3311378	0.0039536	.	.	.	.	.	.	yes
RRN3	.	.	0.3304236	0.0040371	.	.	.	.	0.5043144	4.62E-06	.	.	.
RRP1	.	.	.	.	.	.	.	.	.	.	.	.	yes
RRS1	.	.	.	.	.	.	.	.	0.481744	1.39E-05	.	.	.
RSG1	.	.	.	.	.	.	.	.	.	.	0.6344267	1.29E-09	.
RSL1D1	.	.	.	.	.	.	.	.	.	.	.	.	yes
RSL24D1	.	.	.	.	.	.	.	.	0.475867	1.83E-05	.	.	.
RSPH10B2	.	.	.	.	0.6100052	7.96E-09	.	.	.	.	.	.	.
RSPO3	.	.	.	.	.	.	.	.	0.5581565	2.38E-07	.	.	.
RTBDN	.	.	.	.	.	.	.	.	0.6409399	7.70E-10	.	.	.
RTF1	.	.	.	.	0.4760231	1.81E-05	.	.	.	.	.	.	.
RTKN	.	.	.	.	.	.	.	.	0.6366125	1.08E-09	.	.	.
RTN2	.	.	.	.	.	.	0.4844304	1.22E-05	.	.	.	.	.
RTN3	.	.	.	.	.	.	.	.	0.338637	0.0031654	.	.	yes
RTN4	.	.	0.6718773	5.63E-11	.	.	.	.	.	.	.	.	yes
RTN4RL1	.	.	0.4637772	3.16E-05	.	.	.	.	0.3678268	0.0012636	.	.	.
RUVBL1	.	.	.	.	0.5931658	2.56E-08	.	.	.	.	.	.	.
RUVBL2	.	.	.	.	0.3702147	0.0011676	.	.	.	.	.	.	yes
RWDD4	.	.	0.3094044	0.0073085	0.5025817	5.04E-06	.	.	.	.	.	.	.
RXRG	.	.	.	.	.	.	.	.	.	.	0.6962319	5.70E-12	.
RYK	.	.	.	.	.	.	.	.	0.4585639	3.98E-05	.	.	.
S100A1	.	.	0.6173332	4.68E-09	.	.	.	.	0.3469884	0.0024553	.	.	.
SAE1	.	.	.	.	.	.	.	.	0.827659	9.76E-20	.	.	yes
SAFB	.	.	.	.	.	.	.	.	0.4193404	0.0002001	.	.	yes

Gene	Atrial Expression	p-value Atrial	AV-Canal Expression	p-value AV-Canal	Branchial Arches Expression	p-value Branchial Arches	Heart Tube Expression	p-value Heart Tube	Outflow Tract Expression	p-value Outflow Tract	Ventricle Expression	p-value Ventricle	DMP Expression
SAMD1	.	.	0.4120211	0.0002649	.	.	.	.	0.4235504	0.0001698	.	.	.
SAMD11	.	.	.	.	.	.	.	.	0.6979474	4.81E-12	.	.	.
SAMD4A	.	.	.	.	.	.	0.4812676	1.42E-05	0.47458	1.94E-05	.	.	.
SAMD4B	.	.	0.3949879	0.0004963	.	.	.	.	0.4720028	2.18E-05	.	.	.
SAMM50	.	.	0.333321	0.0037079	.	.	.	.	0.5424719	5.96E-07	.	.	.
SAP130	.	.	.	.	.	.	.	.	0.7237921	3.20E-13	.	.	.
SAP30	.	.	0.4347116	0.0001088	0.3388201	0.0031481	.	.	0.5233786	1.71E-06	.	.	.
SAP30L	.	.	0.3697636	0.0011852	.	.	.	.	0.5362765	8.45E-07	.	.	.
SAR1A	.	.	0.4059342	0.0003328	.	.	.	.	.	.	.	.	yes
SARS2	.	.	.	.	.	.	.	.	0.5953669	2.21E-08	.	.	.
SART3	.	.	.	.	0.4373378	9.77E-05	.	.	.	.	.	.	.
SAT1	.	.	.	.	.	.	.	.	0.5821067	5.33E-08	.	.	.
SBK1	.	.	.	.	0.3433238	0.0027472	.	.	.	.	.	.	yes
SBNO1	.	.	0.456548	4.34E-05	.	.	.	.	.	.	.	.	.
SCAF1	.	.	.	.	.	.	.	.	.	.	.	.	yes
SCAF11	.	.	0.4486393	6.10E-05	.	.	.	.	.	.	.	.	yes
SCAF4	.	.	.	.	.	.	.	.	.	.	0.7192861	5.24E-13	.
SCAMP4	.	.	.	.	.	.	.	.	0.5241808	1.64E-06	.	.	.
SCAND1	.	.	0.3942364	0.0005099	.	.	.	.	0.4711077	2.27E-05	.	.	.
SCCPDH	.	.	0.4188468	0.000204	.	.	.	.	.	.	.	.	.
SCGB1C1	.	.	.	.	.	.	.	.	.	.	0.6307239	1.71E-09	.
SCHIP1	.	.	.	.	.	.	.	.	0.5155124	2.60E-06	.	.	.
SCMH1	.	.	.	.	.	.	.	.	.	.	0.5048773	4.49E-06	.
SCML2	.	.	.	.	.	.	.	.	.	.	0.4960034	7.00E-06	.
SCO1	.	.	0.4025441	0.0003772	.	.	.	.	.	.	.	.	.
SCPEP1	.	.	.	.	.	.	.	.	0.4484828	6.14E-05	.	.	.
SCRIB	.	.	.	.	.	.	.	.	.	.	.	.	yes
SCRN2	.	.	0.423082	0.000173	.	.	.	.	.	.	.	.	.
SDC2	.	.	.	.	.	.	.	.	0.446075	6.80E-05	.	.	.
SDCCAG3	.	.	0.5829216	5.06E-08	.	.	.	.	.	.	.	.	.
SDHA	.	.	0.3347708	0.0035523	.	.	.	.	.	.	.	.	yes
SDHAF1	.	.	.	.	.	.	.	.	0.7417576	4.03E-14	.	.	.
SDHB	.	.	0.3261624	0.0045684	.	.	.	.	0.6410126	7.65E-10	.	.	.
SDHC	.	.	.	.	.	.	.	.	0.7050462	2.35E-12	.	.	.
SDHD	.	.	0.3499392	0.0022409	.	.	.	.	0.5558889	2.73E-07	.	.	.
SEC11A	.	.	0.4795933	1.54E-05	.	.	.	.	0.4300876	0.0001311	.	.	.
SEC14L1	.	.	0.5292237	1.25E-06	.	.	.	.	.	.	.	.	.
SEC23A	.	.	0.3794207	0.0008563	.	.	.	.	0.4573881	4.19E-05	.	.	.
SEC23IP	.	.	.	.	0.5667966	1.41E-07	.	.	.	.	.	.	.
SEC24C	.	.	.	.	.	.	.	.	.	.	.	.	yes
SEC31A	.	.	.	.	.	.	.	.	.	.	0.3848615	0.0007098	yes
SEC61a1	.	.	.	.	.	.	.	.	.	.	.	.	yes
SEC63	.	.	.	.	.	.	.	.	0.6145703	5.73E-09	.	.	.
SECISBP2	.	.	.	.	.	.	.	.	0.5135963	2.87E-06	.	.	.
SEH1L	.	.	.	.	.	.	.	.	0.3095088	0.0072877	.	.	yes
SELP	.	.	.	.	.	.	.	.	.	.	0.5447809	5.22E-07	.
SELT	.	.	0.5690892	1.22E-07	.	.	.	.	0.3032384	0.008632	.	.	.
SEPHS1	.	.	.	.	.	.	.	.	.	.	.	.	yes
SEPN1	.	.	.	.	.	.	.	.	0.4770817	1.73E-05	.	.	.
SERBP1	.	.	.	.	.	.	.	.	0.5615315	1.94E-07	.	.	yes
SERF2	.	.	.	.	.	.	.	.	0.450466	5.64E-05	.	.	.
SERHL2	.	.	.	.	.	.	.	.	0.4723793	2.14E-05	.	.	.
SERP1	.	.	.	.	.	.	.	.	.	.	.	.	yes
SERPINB8	.	.	.	.	.	.	.	.	0.5750086	8.41E-08	.	.	.
SERPINE1	.	.	.	.	.	.	.	.	0.668303	7.73E-11	.	.	.
SERPINH1	.	.	.	.	.	.	.	.	.	.	.	.	yes
SERPINI1	.	.	.	.	.	.	.	.	.	.	0.5934189	2.52E-08	.
SERTAD3	.	.	.	.	.	.	.	.	0.4499547	5.77E-05	.	.	.
SESTD1	.	.	.	.	.	.	.	.	.	.	0.5999778	1.61E-08	.
SETD1A	.	.	.	.	.	.	.	.	0.7138317	9.42E-13	.	.	.
SETD5	.	.	0.3061045	0.0079928	.	.	.	.	.	.	.	.	yes
SETD8	.	.	0.4197257	0.0001972	.	.	.	.	0.555343	2.82E-07	.	.	.
SF1	.	.	.	.	0.3952114	0.0004924	.	.	.	.	.	.	yes
SF3A1	.	.	.	.	.	.	.	.	.	.	.	.	yes
SF3B1	.	.	.	.	.	.	.	.	.	.	.	.	yes
SF3B2	.	.	.	.	.	.	.	.	.	.	.	.	yes
SF3B3	.	.	.	.	.	.	.	.	0.391098	0.0005702	.	.	yes
SF3B4	.	.	.	.	0.6191357	4.10E-09	.	.	.	.	.	.	.
SF3B5	.	.	.	.	.	.	.	.	0.5355244	8.82E-07	.	.	.
SFPQ	.	.	.	.	.	.	.	.	.	.	.	.	yes
SFR1	.	.	.	.	.	.	.	.	0.5615958	1.93E-07	.	.	yes
SFRP1	.	.	.	.	.	.	.	.	.	.	.	.	yes
SFT2D1	.	.	0.484595	1.21E-05	.	.	.	.	0.3068385	0.0078359	.	.	.
SFT2D2	.	.	0.5616853	1.92E-07	.	.	.	.	.	.	.	.	.
SGMS1	.	.	.	.	.	.	.	.	0.6328684	1.45E-09	.	.	.

Gene	Atrial Expression	p-value Atrial	AV-Canal Expression	p-value AV-Canal	Branchial Arches Expression	p-value Branchial Arches	Heart Tube Expression	p-value Heart Tube	Outflow Tract Expression	p-value Outflow Tract	Ventricle Expression	p-value Ventricle	DMP Expression
SGPP1	.	.	.	.	.	.	.	.	0.5861387	4.09E-08	.	.	.
SGSM2	.	.	0.3809006	0.0008139	.	.	.	.	.	.	.	.	.
SH2B3	.	.	.	.	.	.	.	.	0.5080919	3.82E-06	.	.	.
SH3BGR	.	.	0.5160632	2.53E-06	.	.	0.3436664	0.0027186	0.5998974	1.62E-08	.	.	.
SH3BGRL	.	.	.	.	.	.	.	.	.	.	.	.	yes
SH3BP4	.	.	.	.	.	.	.	.	0.5959102	2.13E-08	.	.	.
SH3GLB1	.	.	0.4508498	5.55E-05	.	.	.	.	.	.	.	.	.
SH3GLB2	.	.	.	.	.	.	.	.	0.5482518	4.27E-07	.	.	.
SH3PXD2A	.	.	.	.	.	.	.	.	.	.	.	.	yes
SH3RF3	.	.	0.3884976	0.0006251	.	.	.	.	.	.	.	.	.
SH3TC2	.	.	0.473867	2.00E-05	.	.	.	.	.	.	.	.	.
SHC4	.	.	0.3462207	0.0025141	.	.	.	.	0.5105774	3.36E-06	.	.	.
SHH	.	.	.	.	.	.	.	.	0.4550848	4.63E-05	.	.	.
SHISA4	0.3051771	0.0081949	.	.	.	.	.	.	0.4854023	1.17E-05	.	.	.
SHKBP1	.	.	.	.	.	.	.	.	0.8711315	6.18E-24	.	.	.
SHOC2	.	.	0.4503436	5.67E-05	.	.	.	.	0.472586	2.12E-05	.	.	.
SHOX2	.	.	0.4398511	8.81E-05	.	.	.	.	.	.	.	.	.
SIK3	.	.	0.3913699	0.0005647	.	.	.	.	.	.	.	.	.
SIKE1	.	.	0.3302671	0.0040556	.	.	.	.	0.5325169	1.04E-06	.	.	.
SIN3A	.	.	.	.	.	.	.	.	.	.	.	.	yes
SIPA1L1	.	.	0.4621354	3.40E-05	.	.	.	.	.	.	.	.	.
SIPA1L2	.	.	.	.	.	.	.	.	0.6090116	8.55E-09	.	.	.
SIRPA	.	.	.	.	.	.	.	.	0.5783831	6.78E-08	.	.	.
SIX4	.	.	.	.	0.5166168	2.45E-06	.	.	.	.	.	.	.
SKA1	.	.	0.4742624	1.97E-05	.	.	.	.	0.3360319	0.0034217	.	.	.
SKIL	.	.	0.3831541	0.0007531	.	.	.	.	0.335308	0.0034961	.	.	.
SKP1	.	.	.	.	.	.	.	.	.	.	.	.	yes
SLA2	.	.	.	.	.	.	.	.	0.446317	6.73E-05	.	.	.
SLAIN2	.	.	0.5416375	6.25E-07	.	.	.	.	0.3362252	0.0034021	.	.	.
SLC10A4	.	.	.	.	.	.	.	.	0.5932399	2.55E-08	.	.	.
SLC12A9	.	.	.	.	.	.	.	.	0.7724768	7.62E-16	.	.	.
SLC14A1	.	.	.	.	.	.	.	.	0.4947335	7.45E-06	.	.	.
SLC16A1	.	.	0.4056001	0.0003369	.	.	.	.	0.5202129	2.03E-06	.	.	.
SLC16A3	.	.	.	.	.	.	.	.	0.7531527	9.90E-15	.	.	yes
SLC22A13	.	.	.	.	.	.	.	.	.	.	0.5635845	1.71E-07	.
SLC22A16	.	.	0.40828	0.0003049	.	.	.	.	.	.	.	.	.
SLC22A17	.	.	.	.	.	.	.	.	0.8612166	7.44E-23	.	.	.
SLC22A3	.	.	.	.	.	.	.	.	0.6168213	4.86E-09	.	.	.
SLC24A4	.	.	0.486024	1.13E-05	.	.	.	.	.	.	.	.	.
SLC25A10	.	.	.	.	.	.	.	.	0.4480109	6.26E-05	.	.	.
SLC25A15	.	.	.	.	.	.	.	.	0.5576552	2.45E-07	.	.	.
SLC25A17	.	.	0.3878071	0.0006404	.	.	.	.	0.3658535	0.0013482	.	.	.
SLC25A20	.	.	0.3997799	0.0004174	.	.	.	.	.	.	.	.	.
SLC25A22	.	.	.	.	.	.	.	.	0.4894975	9.60E-06	.	.	.
SLC25A24	.	.	0.3294385	0.0041548	.	.	.	.	.	.	0.453297	5.00E-05	.
SLC25A25	.	.	0.4981618	6.29E-06	.	.	.	.	.	.	.	.	.
SLC25A3	.	.	0.4623877	3.36E-05	.	.	.	.	.	.	.	.	yes
SLC25A37	.	.	0.4436953	7.51E-05	.	.	.	.	.	.	.	.	.
SLC25A39	.	.	.	.	.	.	.	.	0.5918963	2.79E-08	.	.	.
SLC25A4	.	.	0.445181	7.06E-05	.	.	.	.	0.3546117	0.0019355	.	.	yes
SLC25A46	.	.	0.4445607	7.24E-05	.	.	.	.	.	.	.	.	.
SLC27A4	.	.	.	.	.	.	.	.	0.4922456	8.41E-06	.	.	.
SLC2A1	.	.	0.3007624	0.0092198	.	.	.	.	0.7624295	2.98E-15	.	.	yes
SLC2A3	.	.	.	.	.	.	.	.	0.4437095	7.51E-05	.	.	yes
SLC2A5	.	.	.	.	.	.	.	.	.	.	0.6428582	6.60E-10	.
SLC2A6	.	.	.	.	0.549217	4.04E-07	.	.	.	.	.	.	.
SLC2A8	.	.	.	.	.	.	.	.	0.6017726	1.42E-08	.	.	.
SLC30A7	.	.	0.5543394	2.99E-07	.	.	.	.	.	.	.	.	.
SLC30A9	.	.	0.55381	3.08E-07	.	.	.	.	.	.	.	.	.
SLC31A1	.	.	.	.	.	.	.	.	0.608021	9.17E-09	.	.	.
SLC35A4	0.5515388	3.53E-07	.	.	.	.	.	.	.	.	.	.	.
SLC35D2	.	.	.	.	0.746685	2.22E-14	.	.	.	.	.	.	.
SLC35E2B	.	.	.	.	.	.	.	.	0.4442213	7.35E-05	.	.	.
SLC35E3	.	.	.	.	0.4411764	8.34E-05	.	.	0.4238742	0.0001677	.	.	.
SLC35E4	.	.	.	.	.	.	.	.	0.4957929	7.07E-06	.	.	.
SLC36A4	.	.	0.3990552	0.0004285	.	.	.	.	.	.	.	.	.
SLC38A1	.	.	.	.	.	.	.	.	.	.	.	.	yes
SLC38A2	.	.	.	.	.	.	.	.	.	.	.	.	yes
SLC38A4	.	.	.	.	.	.	.	.	0.6823249	2.16E-11	.	.	yes
SLC39A1	.	.	0.3962809	0.0004738	.	.	.	.	.	.	.	.	.
SLC39A10	.	.	0.3138942	0.0064604	.	.	.	.	.	.	.	.	yes
SLC39A7	.	.	0.3419497	0.0028644	.	.	.	.	.	.	.	.	yes
SLC39A9	.	.	.	.	0.564167	1.65E-07	.	.	.	.	.	.	.
SLC41A1	.	.	.	.	.	.	.	.	0.5528759	3.26E-07	.	.	.
SLC41A3	.	.	0.41994	0.0001955	.	.	.	.	.	.	.	.	.

Gene	Atrial Expression	p-value Atrial	AV-Canal Expression	p-value AV-Canal	Branchial Arches Expression	p-value Branchial Arches	Heart Tube Expression	p-value Heart Tube	Outflow Tract Expression	p-value Outflow Tract	Ventricle Expression	p-value Ventricle	DMP Expression
SLC50A1	.	.	.	.	.	.	.	.	0.6029546	1.31E-08	.	.	.
SLC7A14	.	.	.	.	.	.	0.5470413	4.58E-07	.	.	.	.	.
SLC7A6OS	.	.	0.4584348	4.00E-05	.	.	.	.	0.4492284	5.95E-05	.	.	.
SLC9B1	.	.	0.4356675	0.0001046	.	.	.	.	0.4296003	0.0001336	.	.	.
SLCO1C1	.	.	.	.	.	.	.	.	.	.	0.5358913	8.64E-07	.
SLCO3A1	.	.	0.5462476	4.80E-07	.	.	.	.	0.3863063	0.000675	.	.	.
SLCO4C1	.	.	0.4854803	1.16E-05	.	.	.	.	0.3215892	0.0052069	.	.	.
SLIRP	.	.	.	.	.	.	.	.	0.5420091	6.12E-07	.	.	.
SLITRK5	.	.	0.4691589	2.48E-05	.	.	.	.	.	.	.	.	.
SLMAP	.	.	.	.	.	.	.	.	.	.	0.4920504	8.49E-06	.
SLMO2	.	.	.	.	.	.	.	.	0.4953858	7.21E-06	.	.	.
SMAD5	.	.	.	.	.	.	.	.	.	.	.	.	yes
SMAD6	.	.	0.6379183	9.78E-10	.	.	.	.	.	.	.	.	.
SMAD7	.	.	0.4575985	4.15E-05	.	.	.	.	0.5253475	1.54E-06	.	.	.
SMARCA4	.	.	.	.	.	.	.	.	.	.	.	.	yes
SMARCA5	.	.	0.3801202	0.000836	.	.	.	.	.	.	.	.	.
SMARCB1	.	.	.	.	.	.	.	.	.	.	.	.	yes
SMARCC1	.	.	.	.	.	.	.	.	.	.	.	.	yes
SMARCC2	.	.	.	.	.	.	.	.	.	.	.	.	yes
SMARCD1	.	.	.	.	0.7456745	2.51E-14	.	.	.	.	.	.	yes
SMARCD3	.	.	.	.	.	.	.	.	0.647061	4.70E-10	.	.	.
SMC1A	.	.	.	.	.	.	.	.	.	.	.	.	yes
SMC2	.	.	.	.	.	.	.	.	.	.	.	.	yes
SMC3	.	.	.	.	.	.	.	.	.	.	.	.	yes
SMC4	.	.	.	.	.	.	.	.	0.3138048	0.0064764	.	.	yes
SMCHD1	.	.	0.3383976	0.0031883	.	.	.	.	.	.	.	.	yes
SMCR8	.	.	0.5701014	1.15E-07	.	.	.	.	.	.	.	.	.
SMG1	.	.	.	.	.	.	.	.	.	.	0.3250183	0.0047213	yes
SMIM12	.	.	.	.	.	.	.	.	0.7178358	6.13E-13	.	.	.
SMIM20	.	.	.	.	.	.	.	.	0.7203948	4.65E-13	.	.	.
SMIM7	.	.	0.5433941	5.65E-07	.	.	.	.	0.3256745	0.0046331	.	.	.
SMO	.	.	.	.	.	.	.	.	0.4214235	0.0001846	.	.	yes
SMPD2	.	.	.	.	.	.	.	.	0.4935647	7.88E-06	.	.	.
SMPDL3A	.	.	.	.	.	.	.	.	0.4496476	5.84E-05	.	.	.
SMPX	.	.	.	.	.	.	.	.	0.8482416	1.46E-21	.	.	.
SMS	.	.	.	.	.	.	.	.	0.4568975	4.28E-05	.	.	.
SMTNL2	.	.	.	.	.	.	.	.	0.7717686	8.41E-16	.	.	.
SMUG1	.	.	.	.	.	.	.	.	0.4541405	4.82E-05	.	.	.
SMURF1	.	.	.	.	.	.	.	.	0.5180128	2.28E-06	.	.	.
SMYD1	.	.	.	.	.	.	.	.	0.9612557	4.96E-42	.	.	.
SMYD5	.	.	.	.	0.300994	0.0091634	.	.	0.5612677	1.97E-07	.	.	.
SNAI2	.	.	.	.	0.5612198	1.98E-07	.	.	.	.	.	.	.
SNAP23	.	.	.	.	.	.	.	.	0.5325354	1.04E-06	.	.	.
SND1	.	.	.	.	.	.	.	.	0.7402005	4.86E-14	.	.	yes
SNED1	.	.	.	.	.	.	.	.	0.498508	6.18E-06	.	.	.
SNRNP200	.	.	0.3519722	0.002103	.	.	.	.	0.42629	0.0001525	.	.	yes
SNRNP40	.	.	.	.	.	.	.	.	0.6841477	1.83E-11	.	.	.
SNRNP70	.	.	.	.	.	.	.	.	.	.	.	.	yes
SNRPB	.	.	.	.	.	.	.	.	.	.	.	.	yes
SNRPD1	.	.	.	.	.	.	.	.	0.4425455	7.88E-05	.	.	.
SNRPD3	.	.	.	.	0.3517163	0.00212	.	.	.	.	.	.	yes
SNRPN	.	.	.	.	.	.	0.6787275	3.02E-11	.	.	.	.	.
SNTA1	.	.	.	.	.	.	.	.	0.4831425	1.30E-05	.	.	.
SNUPN	.	.	.	.	.	.	.	.	.	.	0.699389	4.17E-12	.
SNX14	.	.	.	.	.	.	.	.	0.571147	1.07E-07	.	.	.
SNX17	.	.	.	.	.	.	.	.	0.4906974	9.06E-06	.	.	.
SNX18	.	.	0.3759983	0.0009619	.	.	.	.	0.3184016	0.0056973	.	.	.
SNX21	.	.	.	.	.	.	.	.	0.6287193	2.00E-09	.	.	.
SNX3	.	.	0.4386216	9.27E-05	.	.	.	.	0.3414641	0.0029068	.	.	.
SNX5	.	.	.	.	0.3470471	0.0024509	.	.	.	.	.	.	yes
SOD1	.	.	0.3717526	0.0011094	.	.	.	.	0.5963951	2.06E-08	.	.	.
SOD2	.	.	0.4096672	0.0002895	.	.	.	.	.	.	.	.	.
SON	.	.	.	.	.	.	.	.	.	.	.	.	yes
SORBS1	.	.	.	.	.	.	.	.	0.3393943	0.0030942	0.496084	6.97E-06	.
SORT1	.	.	.	.	.	.	.	.	.	.	0.5781982	6.86E-08	.
SOST	0.4160465	0.0002272	.	.	.	.	.	.	0.467079	2.73E-05	.	.	.
SOX11	.	.	.	.	0.4086753	0.0003004	.	.	.	.	.	.	yes
SOX12	.	.	.	.	.	.	.	.	.	.	.	.	yes
SOX9	.	.	0.4301277	0.0001308	.	.	.	.	.	.	.	.	.
SP1	.	.	.	.	.	.	.	.	.	.	.	.	yes
SPAG4	.	.	.	.	.	.	.	.	0.694341	6.87E-12	.	.	.
SPARC	.	.	.	.	.	.	.	.	.	.	.	.	yes
SPAST	.	.	.	.	.	.	.	.	0.6335282	1.38E-09	.	.	.
SPATA13	.	.	.	.	.	.	.	.	0.6235244	2.96E-09	.	.	.
SPATA18	.	.	.	.	.	.	.	.	0.6814709	2.34E-11	.	.	.

Gene	Atrial Expression	p-value Atrial	AV-Canal Expression	p-value AV-Canal	Branchial Arches Expression	p-value Branchial Arches	Heart Tube Expression	p-value Heart Tube	Outflow Tract Expression	p-value Outflow Tract	Ventricle Expression	p-value Ventricle	DMP Expression
SPATA24	.	.	.	.	.	.	.	.	0.489543	9.58E-06	.	.	.
SPATA33	.	.	.	.	.	.	.	.	0.7018999	3.24E-12	.	.	.
SPATC1L	.	.	.	.	.	.	.	.	.	.	0.5225675	1.79E-06	.
SPATS2	.	.	.	.	.	.	.	.	0.6285607	2.02E-09	.	.	.
SPCS2	.	.	.	.	.	.	.	.	0.5496883	3.93E-07	.	.	.
SPCS3	.	.	.	.	.	.	.	.	0.46456	3.05E-05	.	.	.
SPEG	.	.	.	.	.	.	.	.	0.5383265	7.54E-07	.	.	.
SPIN1	.	.	.	.	.	.	.	.	0.462139	3.40E-05	.	.	.
SPINK5	.	.	.	.	.	.	.	.	.	.	0.6103436	7.77E-09	.
SPNS1	.	.	.	.	0.3303085	0.0040507	.	.	0.6485302	4.16E-10	.	.	.
SPOCK3	.	.	.	.	.	.	.	.	.	.	0.6957267	5.99E-12	.
SPON1	.	.	0.7912327	4.91E-17	.	.	.	.	.	.	.	.	.
SPPL3	.	.	.	.	.	.	.	.	0.5125913	3.03E-06	.	.	.
SPR	.	.	.	.	.	.	.	.	0.4617257	3.46E-05	.	.	.
SPRYD3	.	.	0.4275733	0.0001449	.	.	.	.	.	.	.	.	.
SPRYD7	.	.	.	.	.	.	.	.	0.4820368	1.37E-05	.	.	.
SPSB3	.	.	.	.	.	.	.	.	0.5587495	2.30E-07	.	.	.
SPSB4	.	.	.	.	.	.	.	.	0.526933	1.41E-06	.	.	.
SPTAN1	.	.	.	.	.	.	.	.	.	.	.	.	yes
SPTBN1	.	.	0.4228853	0.0001743	.	.	.	.	0.5491955	4.04E-07	.	.	yes
SPTSSB	.	.	.	.	.	.	.	.	.	.	0.5332254	1.00E-06	.
SRC	.	.	.	.	0.4526084	5.15E-05	.	.	.	.	.	.	.
SRCRB4D	.	.	.	.	.	.	.	.	.	.	0.4638992	3.14E-05	.
SREBF2	.	.	0.3328769	0.0037567	.	.	.	.	0.3082605	0.0075397	.	.	yes
SREK1	.	.	0.4742118	1.97E-05	.	.	.	.	0.3209349	0.0053044	.	.	.
SRF	.	.	.	.	.	.	.	.	0.5671652	1.37E-07	.	.	.
SRGAP2	.	.	.	.	.	.	.	.	0.4506149	5.61E-05	.	.	.
SRM	.	.	.	.	0.5434041	5.65E-07	.	.	.	.	.	.	.
SRP68	.	.	0.3347062	0.0035591	.	.	.	.	0.6539099	2.67E-10	.	.	.
SRPK3	.	.	.	.	.	.	.	.	0.702276	3.12E-12	.	.	.
SRRM1	.	.	.	.	.	.	.	.	0.4366976	0.0001003	.	.	yes
SRRM2	.	.	.	.	.	.	.	.	.	.	.	.	yes
SRRT	.	.	.	.	.	.	.	.	0.5895086	3.28E-08	.	.	yes
SRSF1	.	.	.	.	.	.	.	.	.	.	.	.	yes
SRSF11	.	.	.	.	.	.	.	.	0.4574908	4.17E-05	.	.	yes
SRSF2	.	.	.	.	.	.	.	.	.	.	.	.	yes
SRSF3	.	.	.	.	.	.	.	.	.	.	.	.	yes
SRSF6	.	.	0.3278855	0.0043465	.	.	.	.	.	.	.	.	yes
SRSF7	.	.	.	.	.	.	.	.	.	.	.	.	yes
SS18L1	.	.	.	.	.	.	.	.	.	.	0.6949775	6.45E-12	.
SS18L2	.	.	0.4179757	0.000211	.	.	.	.	.	.	.	.	.
SSB	.	.	.	.	.	.	.	.	.	.	.	.	yes
SSBP3	.	.	.	.	.	.	.	.	.	.	.	.	yes
SSBP4	0.504255	4.64E-06	.	.	.	.	.	.	.	.	.	.	.
SSH1	.	.	.	.	.	.	.	.	0.3433985	0.0027409	0.4867175	1.10E-05	.
SSR1	.	.	0.4156345	0.0002308	.	.	.	.	0.3100473	0.0071814	.	.	yes
SSR3	.	.	0.3671981	0.00129	.	.	.	.	0.7805458	2.42E-16	.	.	yes
SSRP1	.	.	.	.	0.3527494	0.0020524	.	.	0.5435994	5.59E-07	.	.	yes
SSU72	.	.	.	.	.	.	.	.	0.6106806	7.58E-09	.	.	.
SSUH2	.	.	0.4004605	0.0004071	.	.	.	.	.	.	.	.	.
ST13	.	.	.	.	.	.	.	.	.	.	.	.	yes
ST3GAL5	.	.	0.5310544	1.13E-06	.	.	.	.	.	.	.	.	.
ST6GAL2	.	.	.	.	.	.	.	.	.	.	0.6980491	4.76E-12	.
ST6GALNAC4	.	.	.	.	.	.	.	.	0.7793861	2.86E-16	.	.	.
ST6GALNAC5	.	.	.	.	.	.	.	.	.	.	0.7023218	3.10E-12	.
ST7L	.	.	0.4407147	8.50E-05	.	.	.	.	.	.	.	.	.
STAP1	.	.	.	.	.	.	.	.	0.6354786	1.18E-09	.	.	.
STAP2	.	.	0.4676347	2.66E-05	.	.	.	.	.	.	.	.	.
STARD3	.	.	.	.	.	.	0.4484756	6.14E-05	.	.	.	.	.
STARD5	.	.	.	.	.	.	.	.	0.4438514	7.46E-05	.	.	.
STEAP3	.	.	0.4692642	2.47E-05	.	.	.	.	.	.	.	.	.
STIP1	.	.	.	.	.	.	.	.	0.6873633	1.35E-11	.	.	yes
STK11	.	.	0.3951714	0.0004931	.	.	.	.	.	.	.	.	.
STK11IP	.	.	.	.	.	.	.	.	0.4612756	3.53E-05	.	.	.
STK16	.	.	.	.	.	.	.	.	0.6639714	1.13E-10	.	.	.
STK24	.	.	0.4342648	0.0001107	.	.	.	.	.	.	.	.	.
STK3	.	.	0.3430404	0.002771	.	.	.	.	0.4937549	7.81E-06	.	.	.
STK32A	.	.	.	.	.	.	.	.	0.6847042	1.73E-11	.	.	.
STK38L	.	.	.	.	0.4470651	6.52E-05	.	.	.	.	.	.	.
STK4	.	.	0.3948264	0.0004992	.	.	.	.	.	.	.	.	.
STRAP	.	.	0.4534732	4.96E-05	.	.	.	.	0.4311193	0.0001257	.	.	yes
STRN3	.	.	.	.	.	.	.	.	0.5244537	1.62E-06	.	.	.
STRN4	.	.	0.3827274	0.0007643	.	.	.	.	.	.	.	.	.
STT3A	.	.	.	.	.	.	.	.	0.5191457	2.15E-06	.	.	yes
STT3B	.	.	0.5833988	4.90E-08	.	.	.	.	0.4102633	0.000283	.	.	yes

Gene	Atrial Expression	p-value Atrial	AV-Canal Expression	p-value AV-Canal	Branchial Arches Expression	p-value Branchial Arches	Heart Tube Expression	p-value Heart Tube	Outflow Tract Expression	p-value Outflow Tract	Ventricle Expression	p-value Ventricle	DMP Expression
STX12	.	.	.	.	.	.	.	.	0.5482349	4.28E-07	.	.	.
STX1A	.	.	.	.	.	.	.	.	0.6285487	2.02E-09	.	.	.
STX1B	.	.	0.3966498	0.0004675	.	.	.	.	.	.	.	.	.
STX2	.	.	.	.	.	.	.	.	0.643983	6.03E-10	.	.	.
STXBP1	.	.	.	.	.	.	.	.	0.6227729	3.13E-09	.	.	.
STXBP5L	.	.	.	.	.	.	.	.	.	.	0.6397895	8.43E-10	.
STXBP6	.	.	.	.	.	.	.	.	0.520509	2.00E-06	.	.	.
SUB1	.	.	.	.	.	.	.	.	0.6052411	1.12E-08	.	.	yes
SUCLA2	.	.	0.4329541	0.0001168	.	.	.	.	.	.	.	.	.
SUCLG1	.	.	.	.	.	.	.	.	0.5490355	4.08E-07	.	.	.
SUDS3	.	.	.	.	.	.	.	.	0.7820522	1.94E-16	.	.	.
SUGP1	.	.	.	.	.	.	.	.	0.6043032	1.19E-08	.	.	.
SUGT1	.	.	0.5587178	2.30E-07	.	.	.	.	0.3083914	0.0075129	.	.	.
SULF2	.	.	.	.	.	.	.	.	.	.	.	.	yes
SUMO3	.	.	.	.	.	.	0.3393089	0.0031022	0.3265424	0.0045187	.	.	yes
SUPT16H	.	.	.	.	.	.	.	.	.	.	.	.	yes
SUPT5H	.	.	.	.	.	.	.	.	0.3665853	0.0013162	.	.	yes
SUPT6H	.	.	.	.	.	.	.	.	.	.	.	.	yes
SUPT7L	.	.	.	.	.	.	.	.	0.4471769	6.49E-05	.	.	.
SURF1	.	.	.	.	.	.	.	.	0.4674216	2.68E-05	.	.	.
SURF4	.	.	.	.	.	.	0.3070702	0.0077869	0.6656783	9.74E-11	.	.	yes
SUV39H2	.	.	.	.	.	.	.	.	.	.	0.4799006	1.51E-05	.
SVEP1	.	.	.	.	.	.	.	.	0.461043	3.57E-05	.	.	.
SYAP1	.	.	0.5421056	6.08E-07	.	.	.	.	0.316425	0.0060216	.	.	.
SYCE1	.	.	.	.	.	.	.	.	0.441973	8.07E-05	.	.	.
SYMPK	.	.	.	.	.	.	.	.	.	.	0.3798996	0.0008424	yes
SYN1	.	.	0.3817769	0.0007898	.	.	.	.	.	.	.	.	.
SYNCRIP	.	.	.	.	.	.	.	.	.	.	.	.	yes
SYNE2	.	.	0.3105494	0.0070834	.	.	.	.	.	.	.	.	yes
SYNE3	.	.	.	.	.	.	.	.	.	.	0.4828393	1.32E-05	.
SYNGAP1	.	.	.	.	.	.	.	.	.	.	0.6747229	4.35E-11	.
SYNPO2	.	.	.	.	.	.	.	.	.	.	0.6239755	2.86E-09	.
SYNPO2L	.	.	0.398837	0.0004319	.	.	.	.	.	.	.	.	.
SYTL5	.	.	.	.	.	.	.	.	.	.	0.5145955	2.73E-06	.
SZRD1	.	.	.	.	.	.	.	.	0.4422042	7.99E-05	.	.	.
T	.	.	.	.	0.4555971	4.53E-05	.	.	.	.	0.3709964	0.0011377	.
TAB2	.	.	.	.	.	.	.	.	0.4100255	0.0002856	.	.	yes
TAB3	.	.	.	.	.	.	.	.	0.5359084	8.63E-07	.	.	.
TACC3	.	.	0.3154187	0.0061928	.	.	.	.	.	.	.	.	yes
TADA1	.	.	.	.	.	.	.	.	0.4819021	1.38E-05	.	.	.
TAF10	.	.	0.4405478	8.56E-05	.	.	.	.	0.5166035	2.46E-06	.	.	.
TAF13	.	.	0.4043771	0.0003526	.	.	.	.	0.4328596	0.0001172	.	.	.
TAF15	.	.	.	.	0.5173046	2.37E-06	.	.	.	.	.	.	yes
TAF5L	.	.	.	.	.	.	.	.	0.5093091	3.58E-06	.	.	.
TAF8	.	.	.	.	.	.	.	.	0.5045946	4.56E-06	.	.	.
TAF9	.	.	0.4242103	0.0001655	.	.	.	.	.	.	.	.	.
TAGLN	.	.	.	.	.	.	.	.	0.8356651	2.04E-20	.	.	.
TAGLN2	.	.	0.4257903	0.0001555	.	.	.	.	0.5326357	1.04E-06	.	.	.
TARDBP	.	.	.	.	.	.	.	.	.	.	.	.	yes
TARSL2	.	.	.	.	.	.	.	.	.	.	0.4396352	8.89E-05	.
TASP1	.	.	.	.	.	.	.	.	0.5783425	6.80E-08	.	.	.
TAX1BP3	.	.	0.4911952	8.85E-06	.	.	.	.	.	.	.	.	.
TBC1D1	.	.	.	.	.	.	.	.	0.5809293	5.75E-08	.	.	.
TBC1D16	.	.	.	.	.	.	.	.	.	.	.	.	yes
TBC1D17	.	.	.	.	.	.	.	.	0.530553	1.16E-06	.	.	.
TBC1D21	.	.	.	.	.	.	.	.	.	.	0.5219018	1.85E-06	.
TBC1D23	.	.	0.4196482	0.0001978	.	.	.	.	.	.	.	.	.
TBC1D31	.	.	0.4161775	0.0002261	.	.	.	.	.	.	.	.	.
TBC1D32	.	.	0.3850885	0.0007042	.	.	.	.	0.3733525	0.0010516	.	.	.
TBC1D7	.	.	.	.	.	.	.	.	0.5814669	5.56E-08	.	.	.
TBCB	.	.	0.4052758	0.000341	.	.	.	.	0.5348136	9.17E-07	.	.	.
TBCC	.	.	.	.	.	.	.	.	0.64235	6.87E-10	.	.	.
TBCCD1	.	.	0.3870782	0.000657	.	.	.	.	.	.	.	.	.
TBCE	.	.	.	.	.	.	.	.	0.4986687	6.13E-06	.	.	.
TBL3	.	.	.	.	0.481584	1.40E-05	.	.	.	.	.	.	.
TBRG1	.	.	0.3000606	0.0093926	.	.	.	.	0.5892617	3.33E-08	.	.	.
TBX18	.	.	0.5553393	2.82E-07	.	.	.	.	.	.	.	.	.
TBX20	.	.	0.4861277	1.13E-05	.	.	.	.	0.6787031	3.03E-11	.	.	.
TBX5	.	.	0.4691368	2.48E-05	.	.	.	.	.	.	.	.	.
TCAP	.	.	0.4352967	0.0001062	.	.	.	.	.	.	0.3663659	0.0013257	.
TCERG1	.	.	.	.	.	.	.	.	.	.	.	.	yes
TCF12	.	.	.	.	.	.	.	.	.	.	.	.	yes
TCF25	.	.	.	.	.	.	.	.	.	.	.	.	yes
TCF3	.	.	.	.	0.4028315	0.0003732	.	.	.	.	.	.	yes
TCFL5	.	.	0.4166087	0.0002224	.	.	.	.	0.3127359	0.0066704	.	.	.

Gene	Atrial Expression	p-value Atrial	AV-Canal Expression	p-value AV-Canal	Branchial Arches Expression	p-value Branchial Arches	Heart Tube Expression	p-value Heart Tube	Outflow Tract Expression	p-value Outflow Tract	Ventricle Expression	p-value Ventricle	DMP Expression
TCOF1	.	.	.	.	.	.	.	.	.	.	.	.	yes
TCP1	.	.	.	.	.	.	.	.	0.4441401	7.37E-05	.	.	yes
TCP11L1	.	.	.	.	.	.	.	.	0.8392736	9.81E-21	.	.	.
TCTA	.	.	0.3825319	0.0007695	.	.	.	.	.	.	.	.	.
TCTEX1D1	.	.	.	.	.	.	.	.	.	.	0.6733311	4.94E-11	.
TDGF1	.	.	.	.	.	.	0.3153111	0.0062113	0.5883124	3.55E-08	.	.	.
TDRD7	.	.	.	.	.	.	.	.	0.6644034	1.09E-10	.	.	.
TEAD1	.	.	.	.	.	.	.	.	0.3159063	0.0061093	.	.	yes
TEAD2	.	.	.	.	0.3483575	0.0023536	.	.	.	.	.	.	yes
TEC	.	.	.	.	.	.	.	.	.	.	0.6222603	3.25E-09	.
TECR	.	.	.	.	.	.	.	.	0.550152	3.82E-07	.	.	.
TECRL	.	.	0.487697	1.05E-05	.	.	.	.	.	.	.	.	.
TECTA	.	.	.	.	.	.	.	.	.	.	0.6499077	3.72E-10	.
TENM3	.	.	.	.	.	.	.	.	.	.	.	.	yes
TESC	.	.	0.4958579	7.05E-06	.	.	.	.	.	.	.	.	.
TEX10	.	.	.	.	0.4393959	8.98E-05	.	.	.	.	.	.	.
TEX13B	.	.	.	.	.	.	.	.	0.6435768	6.23E-10	.	.	.
TEX261	.	.	0.3916359	0.0005594	.	.	.	.	.	.	.	.	.
TEX35	.	.	.	.	.	.	.	.	.	.	0.4863062	1.12E-05	.
TEX37	.	.	.	.	.	.	0.5383782	7.51E-07	.	.	.	.	.
TFAM	.	.	0.5318924	1.08E-06	.	.	.	.	.	.	.	.	.
TFCP2	.	.	.	.	.	.	.	.	0.5293221	1.24E-06	.	.	.
TFE3	.	.	0.2978981	0.0099431	.	.	.	.	0.5904735	3.07E-08	.	.	.
TFEB	.	.	0.4443645	7.30E-05	.	.	.	.	.	.	.	.	.
TFIP11	.	.	.	.	.	.	.	.	0.5662008	1.46E-07	.	.	.
TFPI	.	.	0.5346944	9.23E-07	.	.	.	.	.	.	0.3583841	0.0017168	.
TFRC	.	.	0.3583438	0.001719	.	.	.	.	.	.	.	.	yes
TGFB1	.	.	0.4618391	3.44E-05	.	.	.	.	0.4418036	8.13E-05	.	.	.
TGFB2	.	.	.	.	.	.	.	.	0.4573081	4.20E-05	.	.	.
TGFBR1	.	.	0.3936721	0.0005203	.	.	.	.	.	.	.	.	yes
TGFBRAP1	.	.	0.3238646	0.00488	.	.	.	.	.	.	0.5342322	9.47E-07	.
TGIF2	.	.	.	.	.	.	.	.	0.5635716	1.71E-07	.	.	.
THBS1	.	.	0.4722067	2.16E-05	.	.	.	.	.	.	.	.	.
THBS4	.	.	0.4748727	1.91E-05	.	.	.	.	.	.	.	.	.
THOC1	.	.	.	.	.	.	.	.	0.5186448	2.20E-06	.	.	.
THPO	.	.	.	.	.	.	.	.	.	.	0.646904	4.76E-10	.
THSD4	.	.	.	.	.	.	.	.	0.5758847	7.95E-08	.	.	.
THUMPD1	.	.	0.3017046	0.0089921	.	.	.	.	0.5318337	1.08E-06	.	.	.
TIMELESS	.	.	0.5079476	3.84E-06	.	.	.	.	.	.	.	.	.
TIMM10B	.	.	0.3896377	0.0006005	.	.	.	.	.	.	.	.	.
TIMM13	.	.	.	.	.	.	.	.	0.6528975	2.90E-10	.	.	.
TIMM17A	.	.	0.3478262	0.0023927	.	.	.	.	0.4476613	6.36E-05	.	.	.
TIMM21	.	.	0.3806305	0.0008215	.	.	.	.	0.5224215	1.80E-06	.	.	.
TIMM22	.	.	.	.	.	.	.	.	0.7887535	7.17E-17	.	.	.
TIMM44	.	.	.	.	.	.	.	.	0.5742292	8.84E-08	.	.	.
TIMM8A	.	.	0.3942419	0.0005098	0.3740525	0.0010271	.	.	.	.	.	.	.
TIMM8B	.	.	0.4963735	6.87E-06	.	.	.	.	.	.	.	.	.
TIMM9	.	.	.	.	.	.	.	.	0.5108302	3.32E-06	.	.	.
TJP1	.	.	.	.	.	.	.	.	.	.	0.6986481	4.49E-12	yes
TJP2	.	.	.	.	.	.	.	.	0.6591139	1.72E-10	.	.	.
TKT	.	.	.	.	0.4699228	2.40E-05	.	.	.	.	.	.	yes
TLE3	.	.	0.3994332	0.0004227	.	.	.	.	.	.	.	.	yes
TLN1	.	.	0.3104186	0.0071088	.	.	.	.	0.3499843	0.0022377	.	.	yes
TLN2	.	.	.	.	.	.	.	.	0.5655494	1.52E-07	.	.	.
TLX1	.	.	.	.	.	.	.	.	0.8978211	2.35E-27	.	.	.
TM6SF1	.	.	.	.	.	.	.	.	0.691419	9.12E-12	.	.	.
TM7SF3	.	.	.	.	.	.	.	.	0.5475529	4.45E-07	.	.	.
TM9SF3	.	.	.	.	.	.	.	.	0.5601588	2.11E-07	.	.	yes
TM9SF4	.	.	.	.	.	.	.	.	0.5557371	2.75E-07	.	.	.
TMA16	.	.	.	.	0.4628095	3.30E-05	.	.	.	.	.	.	.
TMBIM1	.	.	0.6071287	9.77E-09	.	.	.	.	.	.	.	.	.
TMBIM6	.	.	.	.	.	.	.	.	.	.	.	.	yes
TMC7	.	.	.	.	.	.	0.5831807	4.97E-08	.	.	.	.	.
TMCO1	.	.	0.3820972	0.0007811	.	.	.	.	.	.	.	.	.
TMED10	.	.	.	.	.	.	.	.	0.5019624	5.20E-06	.	.	.
TMED4	.	.	.	.	.	.	.	.	0.45	5.76E-05	.	.	.
TMED9	.	.	.	.	.	.	.	.	.	.	.	.	yes
TMEM115	.	.	0.4010219	0.0003989	.	.	.	.	.	.	.	.	.
TMEM116	.	.	.	.	.	.	.	.	.	.	0.5138791	2.83E-06	.
TMEM123	.	.	0.6435244	6.26E-10	.	.	.	.	.	.	.	.	.
TMEM129	.	.	0.4121996	0.0002631	.	.	.	.	.	.	.	.	.
TMEM132A	.	.	.	.	.	.	.	.	.	.	.	.	yes
TMEM147	.	.	0.3314486	0.0039178	.	.	.	.	0.6771626	3.49E-11	.	.	.
TMEM14C	0.4102184	0.0002835	.	.	.	.	.	.	0.6035037	1.26E-08	.	.	.
TMEM150A	.	.	.	.	.	.	.	.	0.4474484	6.42E-05	.	.	.

Gene	Atrial Expression	p-value Atrial	AV-Canal Expression	p-value AV-Canal	Branchial Arches Expression	p-value Branchial Arches	Heart Tube Expression	p-value Heart Tube	Outflow Tract Expression	p-value Outflow Tract	Ventricle Expression	p-value Ventricle	DMP Expression
TMEM161B	.	.	0.3944477	0.000506	.	.	.	.	.	.	.	.	.
TMEM163	0.3831667	0.0007528	0.5486361	4.18E-07	.	.	.	.	.	.	.	.	.
TMEM164	.	.	.	.	.	.	0.4857643	1.15E-05	.	.	.	.	yes
TMEM167A	.	.	0.3246145	0.0047763	.	.	.	.	0.5439352	5.48E-07	.	.	.
TMEM167B	.	.	.	.	.	.	.	.	0.5758183	7.99E-08	.	.	.
TMEM177	.	.	.	.	.	.	.	.	0.5009404	5.48E-06	.	.	.
TMEM178B	.	.	0.3987068	0.000434	.	.	.	.	.	.	.	.	.
TMEM185A	.	.	.	.	.	.	.	.	0.4467095	6.62E-05	.	.	.
TMEM186	.	.	0.3013446	0.0090785	.	.	.	.	0.446204	6.76E-05	.	.	.
TMEM2	.	.	0.475811	1.83E-05	.	.	.	.	.	.	.	.	.
TMEM200A	.	.	.	.	.	.	.	.	0.631864	1.57E-09	.	.	.
TMEM200B	.	.	0.455745	4.50E-05	.	.	.	.	.	.	.	.	.
TMEM201	.	.	.	.	.	.	.	.	0.4416461	8.18E-05	0.300543	0.0092735	.
TMEM206	.	.	0.4008419	0.0004015	.	.	.	.	.	.	.	.	.
TMEM211	0.4482304	6.21E-05	.	.	.	.	.	.	.	.	.	.	.
TMEM214	.	.	.	.	.	.	.	.	0.5692562	1.21E-07	.	.	.
TMEM225	.	.	.	.	.	.	.	.	.	.	0.4387988	9.20E-05	.
TMEM242	.	.	0.503694	4.77E-06	.	.	.	.	.	.	.	.	.
TMEM246	.	.	.	.	.	.	.	.	0.5190258	2.16E-06	.	.	.
TMEM248	.	.	.	.	0.3322006	0.0038322	.	.	0.5839759	4.72E-08	.	.	.
TMEM30A	.	.	0.3392363	0.003109	.	.	.	.	0.7356377	8.32E-14	.	.	.
TMEM33	.	.	.	.	.	.	.	.	0.4512126	5.47E-05	.	.	.
TMEM50A	.	.	.	.	.	.	.	.	0.577365	7.24E-08	.	.	.
TMEM55A	.	.	0.3621548	0.0015207	.	.	.	.	0.5220223	1.84E-06	.	.	.
TMEM64	.	.	0.5961886	2.09E-08	.	.	.	.	.	.	.	.	.
TMEM88	.	.	0.4153273	0.0002336	.	.	.	.	.	.	.	.	.
TMEM95	.	.	.	.	0.4417427	8.15E-05	.	.	.	.	.	.	.
TMEM9B	.	.	0.3296095	0.0041341	.	.	.	.	0.4779881	1.66E-05	.	.	.
TMF1	.	.	0.3283867	0.0042838	.	.	.	.	0.529456	1.23E-06	.	.	.
TMOD1	.	.	0.310648	0.0070643	.	.	.	.	0.4444097	7.29E-05	.	.	.
TMOD3	.	.	.	.	.	.	.	.	.	.	0.6947976	6.57E-12	.
TMOD4	.	.	.	.	.	.	.	.	.	.	0.4573619	4.19E-05	.
TMPO	.	.	.	.	.	.	.	.	.	.	.	.	yes
TMTC3	.	.	0.494055	7.70E-06	.	.	.	.	.	.	.	.	.
TMUB1	.	.	.	.	.	.	.	.	0.7516694	1.19E-14	.	.	.
TMX1	.	.	.	.	.	.	.	.	0.5097807	3.50E-06	.	.	.
TMX4	.	.	0.5466056	4.70E-07	.	.	.	.	.	.	.	.	.
TNC	.	.	.	.	.	.	.	.	0.9791735	1.33E-51	.	.	yes
TNFRSF12A	.	.	0.4109538	0.0002758	.	.	.	.	0.5100817	3.45E-06	.	.	.
TNFRSF21	.	.	.	.	.	.	.	.	0.4651883	2.97E-05	.	.	.
TNIP1	.	.	.	.	.	.	.	.	0.5751158	8.35E-08	.	.	.
TNIP2	.	.	0.4293694	0.0001349	.	.	.	.	.	.	.	.	.
TNKS2	.	.	.	.	.	.	.	.	.	.	.	.	yes
TNNC1	.	.	0.5498961	3.88E-07	.	.	.	.	0.5275744	1.37E-06	.	.	.
TNNI1	.	.	.	.	.	.	.	.	0.6244504	2.76E-09	.	.	.
TNNI3	.	.	0.6877725	1.29E-11	.	.	.	.	.	.	.	.	.
TNNI3K	.	.	0.3546075	0.0019357	.	.	.	.	0.6810838	2.43E-11	.	.	.
TNNT2	.	.	0.3653022	0.0013727	.	.	.	.	.	.	0.4436685	7.52E-05	.
TNPO1	.	.	.	.	0.4065689	0.000325	.	.	0.3159709	0.0060983	.	.	yes
TNPO2	.	.	0.4118012	0.0002671	.	.	.	.	.	.	0.3623005	0.0015135	.
TNPO3	.	.	.	.	.	.	.	.	.	.	.	.	yes
TNRC6A	.	.	.	.	.	.	.	.	.	.	.	.	yes
TNRC6C	.	.	.	.	.	.	.	.	0.731033	1.42E-13	.	.	.
TNS3	.	.	.	.	.	.	.	.	.	.	.	.	yes
TOE1	.	.	.	.	.	.	.	.	0.6437953	6.12E-10	.	.	.
TOM1	.	.	.	.	.	.	.	.	0.4521373	5.25E-05	.	.	.
TOM1L1	.	.	0.5323219	1.05E-06	.	.	.	.	.	.	.	.	.
TOMM70A	.	.	.	.	.	.	.	.	.	.	.	.	yes
TOP1	.	.	.	.	.	.	.	.	.	.	.	.	yes
TOP2A	.	.	.	.	.	.	.	.	0.4606952	3.62E-05	.	.	yes
TOP2B	.	.	.	.	0.5117565	3.16E-06	.	.	.	.	.	.	yes
TOP3B	.	.	.	.	.	.	.	.	0.5780958	6.91E-08	.	.	.
TOPBP1	.	.	.	.	.	.	.	.	.	.	.	.	yes
TOPORS	.	.	0.4145636	0.0002405	.	.	.	.	0.5639833	1.67E-07	.	.	.
TOR1AIP2	.	.	.	.	.	.	.	.	0.5816176	5.50E-08	.	.	.
TP53BP1	.	.	.	.	.	.	.	.	.	.	.	.	yes
TP53BP2	.	.	0.3834046	0.0007466	.	.	.	.	0.4242628	0.0001651	.	.	.
TP53I13	.	.	.	.	.	.	.	.	0.6742943	4.53E-11	.	.	.
TP53RK	.	.	.	.	.	.	.	.	0.5744181	8.73E-08	.	.	.
TPGS1	.	.	0.3437958	0.0027079	.	.	.	.	0.5441206	5.42E-07	.	.	.
TPI1	.	.	.	.	.	.	0.446996	6.54E-05	.	.	.	.	yes
TPM3	.	.	.	.	.	.	.	.	0.7611441	3.53E-15	.	.	.
TPMT	.	.	0.4022447	0.0003814	.	.	.	.	.	.	.	.	.
TPR	.	.	.	.	.	.	.	.	.	.	.	.	yes
TPT1	.	.	.	.	.	.	.	.	.	.	.	.	yes

Gene	Atrial Expression	p-value Atrial	AV-Canal Expression	p-value AV-Canal	Branchial Arches Expression	p-value Branchial Arches	Heart Tube Expression	p-value Heart Tube	Outflow Tract Expression	p-value Outflow Tract	Ventricle Expression	p-value Ventricle	DMP Expression
TPX2	.	.	0.3761558	0.0009568	.	.	.	.	.	.	.	.	yes
TRA2A	.	.	.	.	.	.	.	.	.	.	.	.	yes
TRABD	.	.	.	.	.	.	.	.	0.4413199	8.29E-05	.	.	.
TRABD2B	.	.	.	.	.	.	.	.	.	.	0.5302927	1.18E-06	.
TRAF7	.	.	0.3324028	0.0038095	.	.	.	.	0.4396412	8.89E-05	.	.	.
TRAFD1	.	.	.	.	.	.	.	.	0.4457508	6.89E-05	.	.	.
TRAK2	.	.	0.4378514	9.57E-05	.	.	.	.	.	.	.	.	.
TRAM1	.	.	.	.	.	.	.	.	0.5403561	6.72E-07	.	.	.
TRAPPC1	.	.	0.4320284	0.0001212	.	.	.	.	.	.	.	.	.
TRAPPC2L	.	.	.	.	.	.	.	.	0.4441368	7.37E-05	.	.	.
TRAPPC6B	.	.	.	.	.	.	.	.	0.6465532	4.89E-10	.	.	.
TREM2	.	.	.	.	.	.	.	.	0.5382116	7.59E-07	.	.	.
TREML1	.	.	0.4077784	0.0003107	.	.	.	.	.	.	.	.	.
TREML2	.	.	0.4824497	1.34E-05	.	.	.	.	.	.	.	.	.
TRIB2	.	.	0.4365478	0.0001009	.	.	.	.	.	.	.	.	.
TRIL	.	.	.	.	.	.	.	.	0.5343893	9.39E-07	.	.	.
TRIM11	.	.	0.3844693	0.0007196	.	.	.	.	.	.	.	.	.
TRIM13	.	.	0.5217526	1.87E-06	.	.	.	.	.	.	.	.	.
TRIM15	.	.	.	.	.	.	.	.	.	.	0.6487101	4.10E-10	.
TRIM28	.	.	.	.	.	.	.	.	0.5337815	9.71E-07	.	.	yes
TRIM35	.	.	0.3007524	0.0092222	.	.	.	.	0.3344506	0.0035861	.	.	yes
TRIM44	.	.	.	.	.	.	.	.	0.5625247	1.83E-07	.	.	yes
TRIM47	.	.	.	.	.	.	.	.	0.9002765	1.02E-27	.	.	.
TRIM5	.	.	0.5588338	2.28E-07	.	.	.	.	.	.	.	.	.
TRIM54	.	.	.	.	.	.	0.4861813	1.13E-05	.	.	.	.	.
TRIM56	.	.	.	.	.	.	.	.	.	.	0.8903994	2.57E-26	.
TRIM63	.	.	0.4410949	8.37E-05	.	.	.	.	0.6786968	3.03E-11	.	.	.
TRIM7	.	.	.	.	.	.	.	.	.	.	0.4717025	2.21E-05	.
TRIP12	.	.	.	.	.	.	.	.	.	.	0.5188719	2.18E-06	yes
TRIP13	.	.	.	.	0.4104733	0.0002808	0.525609	1.52E-06	.	.	.	.	.
TRMT1	.	.	0.3365086	0.0033735	.	.	.	.	0.4586263	3.97E-05	.	.	.
TRMT2A	.	.	0.4810051	1.44E-05	.	.	.	.	.	.	.	.	.
TRMT61A	.	.	.	.	.	.	0.6481344	4.30E-10	.	.	.	.	.
TROAP	.	.	0.5619386	1.89E-07	.	.	.	.	.	.	.	.	.
TRPC4	.	.	.	.	0.626319	2.40E-09	.	.	.	.	.	.	.
TRPC4AP	.	.	.	.	.	.	.	.	0.8181422	5.66E-19	.	.	yes
TRPC6	.	.	.	.	.	.	0.496486	6.83E-06	.	.	.	.	.
TRPM3	.	.	.	.	.	.	.	.	.	.	0.5086482	3.71E-06	.
TRPM7	.	.	0.5080324	3.83E-06	.	.	.	.	.	.	.	.	.
TRRAP	.	.	.	.	.	.	.	.	.	.	.	.	yes
TSC2	.	.	.	.	.	.	0.4399644	8.77E-05	.	.	.	.	.
TSC22D1	.	.	0.3210186	0.0052918	.	.	.	.	0.3100187	0.007187	.	.	yes
TSC22D4	.	.	0.5327579	1.03E-06	.	.	.	.	0.4136131	0.0002493	.	.	.
TSEN15	.	.	0.4070811	0.0003189	.	.	.	.	0.3420794	0.0028531	.	.	.
TSEN34	.	.	.	.	0.487592	1.05E-05	.	.	.	.	.	.	.
TSFM	.	.	.	.	0.600661	1.54E-08	.	.	.	.	.	.	.
TSG101	.	.	.	.	0.3964457	0.000471	.	.	0.5371712	8.04E-07	.	.	.
TSN	.	.	.	.	.	.	.	.	0.3797532	0.0008466	.	.	yes
TSPAN13	.	.	0.4309633	0.0001265	.	.	.	.	.	.	.	.	.
TSPAN18	.	.	0.3794258	0.0008561	.	.	.	.	0.5201973	2.03E-06	.	.	.
TSPAN3	.	.	.	.	.	.	.	.	.	.	.	.	yes
TSPAN6	.	.	0.3894589	0.0006043	.	.	.	.	0.638	9.72E-10	.	.	.
TSPAN7	.	.	.	.	0.4967627	6.74E-06	.	.	.	.	.	.	.
TSPAN9	.	.	0.3903525	0.0005855	.	.	.	.	.	.	.	.	.
TSPYL1	.	.	0.3681702	0.0012493	.	.	.	.	0.4754607	1.86E-05	.	.	.
TSPYL5	.	.	0.3917179	0.0005578	.	.	.	.	.	.	.	.	.
TSSC4	.	.	.	.	.	.	.	.	0.5910573	2.95E-08	.	.	.
TTC1	.	.	.	.	.	.	.	.	0.4836875	1.27E-05	.	.	.
TTC14	.	.	.	.	.	.	.	.	.	.	0.6484776	4.18E-10	.
TTC16	.	.	.	.	.	.	.	.	.	.	0.5617067	1.92E-07	.
TTC19	.	.	0.38014	0.0008355	.	.	.	.	.	.	.	.	.
TTC3	.	.	.	.	.	.	.	.	.	.	.	.	yes
TTC30A	.	.	0.3781125	0.0008953	.	.	.	.	0.34569	0.0025554	.	.	.
TTC33	.	.	.	.	.	.	.	.	0.5089421	3.65E-06	.	.	.
TTI2	.	.	.	.	.	.	.	.	0.6028279	1.32E-08	.	.	.
TTLL10	.	.	.	.	.	.	.	.	.	.	0.6167356	4.89E-09	.
TTLL12	.	.	.	.	.	.	.	.	0.4973769	6.54E-06	.	.	.
TTLL5	.	.	.	.	.	.	.	.	0.4400486	8.74E-05	.	.	.
TTPAL	.	.	.	.	.	.	.	.	0.5076049	3.91E-06	.	.	.
TTYH3	.	.	.	.	0.30935	0.0073194	.	.	.	.	.	.	yes
TUBA1A	.	.	0.3125699	0.0067011	.	.	.	.	.	.	.	.	yes
TUBAL3	0.4653305	2.95E-05	.	.	.	.	.	.	.	.	.	.	.
TUBB	.	.	.	.	.	.	.	.	0.3974037	0.000455	.	.	yes
TUBG1	.	.	.	.	.	.	.	.	0.744751	2.81E-14	.	.	.
TUFM	.	.	.	.	.	.	.	.	0.659687	1.64E-10	.	.	.

Gene	Atrial Expression	p-value Atrial	AV-Canal Expression	p-value AV-Canal	Branchial Arches Expression	p-value Branchial Arches	Heart Tube Expression	p-value Heart Tube	Outflow Tract Expression	p-value Outflow Tract	Ventricle Expression	p-value Ventricle	DMP Expression
TULP2	.	.	.	.	.	.	.	.	.	.	0.6987472	4.44E-12	.
TULP4	.	.	.	.	.	.	.	.	0.5483741	4.24E-07	.	.	.
TUSC2	.	.	0.3145305	0.0063475	.	.	.	.	0.5947963	2.30E-08	.	.	.
TWF2	.	.	0.3425269	0.0028146	.	.	.	.	0.5984507	1.79E-08	.	.	.
TWIST1	.	.	0.4585267	3.98E-05	0.426439	0.0001516	.	.	.	.	.	.	.
TXLNA	.	.	.	.	.	.	.	.	0.836054	1.89E-20	.	.	yes
TXLNB	.	.	0.4348669	0.0001081	.	.	.	.	0.6066798	1.01E-08	.	.	.
TXNDC5	.	.	.	.	.	.	.	.	0.3491108	0.0022993	.	.	yes
TXNDC9	.	.	.	.	.	.	.	.	0.4614515	3.50E-05	.	.	.
TXNL1	.	.	.	.	0.3743013	0.0010186	.	.	.	.	.	.	yes
TXNL4A	.	.	.	.	0.363526	0.0014545	.	.	.	.	.	.	yes
TXNRD1	.	.	.	.	0.450273	5.69E-05	.	.	.	.	.	.	yes
TYK2	.	.	.	.	.	.	.	.	0.7955368	2.52E-17	.	.	.
TYMS	.	.	.	.	.	.	.	.	0.7295081	1.68E-13	.	.	.
U2AF1L4	.	.	.	.	.	.	.	.	0.4599554	3.74E-05	.	.	.
U2AF2	.	.	0.3117111	0.0068613	.	.	.	.	0.3909553	0.0005731	.	.	yes
U2SURP	.	.	0.3610087	0.001578	.	.	.	.	.	.	.	.	yes
UAP1	.	.	.	.	.	.	.	.	0.8415189	6.16E-21	.	.	.
UBA1	.	.	0.413946	0.0002462	.	.	.	.	.	.	.	.	yes
UBA2	.	.	.	.	.	.	.	.	.	.	.	.	yes
UBA7	.	.	.	.	.	.	.	.	.	.	0.6843307	1.79E-11	.
UBALD2	.	.	0.5147221	2.71E-06	.	.	.	.	.	.	.	.	.
UBAP2	.	.	.	.	.	.	.	.	.	.	.	.	yes
UBAP2L	.	.	.	.	.	.	.	.	.	.	.	.	yes
UBASH3B	.	.	.	.	.	.	.	.	0.5951197	2.25E-08	.	.	.
UBE2C	.	.	.	.	.	.	.	.	0.6072639	9.67E-09	.	.	.
UBE2D1	.	.	0.4001119	0.0004123	.	.	.	.	0.4441063	7.38E-05	.	.	.
UBE2D3	.	.	.	.	.	.	.	.	0.4976184	6.46E-06	.	.	.
UBE2F	.	.	0.4025365	0.0003773	0.3290806	0.0041983	.	.	.	.	.	.	.
UBE2G1	.	.	0.4277411	0.0001439	.	.	.	.	.	.	.	.	.
UBE2G2	.	.	0.498729	6.11E-06	.	.	.	.	.	.	.	.	.
UBE2H	.	.	0.4017625	0.0003882	.	.	.	.	0.5575579	2.47E-07	.	.	.
UBE2I	.	.	0.3691864	0.0012081	.	.	.	.	0.5274485	1.38E-06	.	.	.
UBE2J1	.	.	0.403968	0.0003579	.	.	.	.	.	.	.	.	.
UBE2J2	.	.	0.3870251	0.0006582	.	.	.	.	.	.	.	.	.
UBE2L3	.	.	.	.	.	.	.	.	0.642164	6.98E-10	.	.	.
UBE2M	.	.	.	.	.	.	.	.	0.6892028	1.13E-11	.	.	.
UBE2O	.	.	.	.	.	.	.	.	0.4561331	4.42E-05	.	.	.
UBE2R2	.	.	0.3743836	0.0010158	.	.	.	.	.	.	.	.	yes
UBE2S	.	.	0.4729452	2.09E-05	.	.	.	.	0.6322477	1.52E-09	.	.	.
UBE2Z	.	.	0.335831	0.0034422	.	.	.	.	0.5518127	3.47E-07	.	.	.
UBE3B	.	.	.	.	.	.	.	.	0.860691	8.44E-23	.	.	.
UBE3C	.	.	0.4046422	0.0003491	.	.	.	.	0.5869074	3.89E-08	.	.	.
UBE3D	.	.	.	.	.	.	.	.	0.4741288	1.98E-05	.	.	.
UBE4B	.	.	0.419157	0.0002016	.	.	.	.	0.336757	0.0033486	.	.	yes
UBFD1	.	.	.	.	.	.	.	.	0.305471	0.0081304	.	.	yes
UBL3	.	.	0.4882246	1.02E-05	.	.	.	.	.	.	.	.	.
UBQLN1	.	.	.	.	0.3359926	0.0034257	.	.	.	.	.	.	yes
UBQLN2	.	.	.	.	.	.	.	.	.	.	.	.	yes
UBQLN3	.	.	.	.	.	.	.	.	.	.	0.5468088	4.64E-07	.
UBQLN4	.	.	.	.	.	.	.	.	0.589308	3.32E-08	.	.	yes
UBR4	.	.	0.354732	0.0019281	.	.	.	.	.	.	.	.	yes
UBR5	.	.	.	.	.	.	.	.	.	.	0.6451853	5.47E-10	yes
UBTF	.	.	.	.	0.3733499	0.0010516	.	.	.	.	.	.	yes
UBXN2B	.	.	.	.	.	.	.	.	.	.	0.4630294	3.27E-05	.
UBXN4	.	.	.	.	.	.	.	.	0.5805659	5.89E-08	.	.	.
UCHL3	.	.	.	.	.	.	.	.	0.5493136	4.02E-07	.	.	.
UCK2	.	.	0.4350525	0.0001073	.	.	.	.	0.4582365	4.03E-05	.	.	.
UFC1	.	.	.	.	.	.	.	.	0.5741289	8.89E-08	.	.	.
UFL1	.	.	.	.	.	.	.	.	0.4374463	9.73E-05	.	.	.
UGGT1	.	.	.	.	.	.	.	.	0.4979271	6.36E-06	.	.	.
UGP2	.	.	0.4098959	0.000287	.	.	.	.	.	.	.	.	.
UGT1A4	.	.	.	.	.	.	0.5250173	1.57E-06	.	.	.	.	.
UGT2A2	.	.	.	.	.	.	.	.	0.4479724	6.27E-05	.	.	.
UGT2B17	.	.	.	.	.	.	.	.	0.5477878	4.39E-07	.	.	.
UHRF1	.	.	.	.	0.4557688	4.49E-05	.	.	.	.	.	.	yes
UHRF1BP1	.	.	0.4044569	0.0003515	.	.	.	.	0.570118	1.14E-07	.	.	.
ULK2	.	.	.	.	.	.	.	.	.	.	0.4979206	6.36E-06	.
UNC119	.	.	.	.	.	.	.	.	0.4743731	1.96E-05	.	.	.
UNC5B	.	.	0.5724877	9.86E-08	.	.	.	.	.	.	.	.	.
UNC80	.	.	.	.	.	.	.	.	.	.	0.4593682	3.84E-05	.
UPF1	.	.	.	.	.	.	.	.	.	.	.	.	yes
UPF3A	.	.	0.48368	1.27E-05	.	.	.	.	.	.	.	.	.
UPK3B	.	.	0.4549082	4.66E-05	.	.	.	.	.	.	.	.	.
UQCR11	.	.	0.3503672	0.0022112	.	.	.	.	0.5521099	3.41E-07	.	.	.

Gene	Atrial Expression	p-value Atrial	AV-Canal Expression	p-value AV-Canal	Branchial Arches Expression	p-value Branchial Arches	Heart Tube Expression	p-value Heart Tube	Outflow Tract Expression	p-value Outflow Tract	Ventricle Expression	p-value Ventricle	DMP Expression
UQCRB	.	.	0.3749798	0.0009956	.	.	0.3019637	0.0089304	0.368371	0.0012411	.	.	.
UQCRC1	.	.	.	.	.	.	.	.	0.4161624	0.0002262	.	.	yes
UQCRC2	.	.	0.5422774	6.03E-07	.	.	.	.	.	.	.	.	yes
UQCRFS1	.	.	0.5079487	3.84E-06	.	.	.	.	0.4932728	8.00E-06	.	.	.
UQCRH	.	.	0.5352948	8.93E-07	.	.	.	.	0.5974366	1.92E-08	.	.	.
URB1	.	.	.	.	.	.	.	.	0.4549195	4.66E-05	.	.	.
USP10	.	.	.	.	.	.	.	.	.	.	.	.	yes
USP12	.	.	.	.	.	.	.	.	0.4452162	7.05E-05	.	.	.
USP13	.	.	0.6002377	1.58E-08	.	.	.	.	.	.	.	.	.
USP19	.	.	.	.	.	.	.	.	.	.	.	.	yes
USP22	.	.	0.3889645	0.0006149	.	.	.	.	0.3693222	0.0012027	.	.	yes
USP30	.	.	.	.	.	.	.	.	0.5811967	5.66E-08	.	.	.
USP36	.	.	.	.	.	.	.	.	0.552477	3.34E-07	.	.	.
USP39	.	.	0.4797535	1.52E-05	.	.	.	.	0.5474114	4.49E-07	.	.	.
USP40	.	.	.	.	.	.	.	.	0.8881744	5.09E-26	.	.	.
USP47	.	.	0.3021422	0.008888	.	.	.	.	.	.	.	.	yes
USP49	.	.	.	.	.	.	.	.	0.6743032	4.52E-11	.	.	.
USP5	.	.	.	.	.	.	.	.	0.3932483	0.0005282	.	.	yes
USP7	.	.	.	.	.	.	.	.	.	.	.	.	yes
USP9X	.	.	.	.	.	.	.	.	.	.	.	.	yes
UTP18	.	.	0.3998904	0.0004157	.	.	.	.	0.4946777	7.47E-06	.	.	.
UTP20	.	.	0.441353	8.28E-05	.	.	.	.	.	.	.	.	.
UTP23	.	.	0.3882659	0.0006302	.	.	.	.	.	.	.	.	.
UTP3	.	.	0.5006403	5.56E-06	.	.	.	.	0.3520856	0.0020956	.	.	.
UTP6	.	.	.	.	0.4401191	8.71E-05	.	.	0.410511	0.0002804	.	.	.
UXT	.	.	.	.	.	.	.	.	0.5198651	2.07E-06	.	.	.
VAMP3	.	.	.	.	.	.	.	.	0.5321502	1.06E-06	.	.	.
VANGL1	.	.	0.3984363	0.0004383	.	.	.	.	0.3194254	0.0055355	.	.	.
VAPB	.	.	0.3636726	0.0014476	0.3123011	0.0067508	.	.	0.4787193	1.60E-05	.	.	.
VARS	.	.	.	.	.	.	.	.	0.582165	5.31E-08	.	.	yes
VASH2	.	.	.	.	.	.	.	.	0.3267711	0.0044889	0.4666478	2.78E-05	.
VAT1	.	.	.	.	.	.	.	.	0.7748841	5.44E-16	.	.	.
VAV2	.	.	.	.	.	.	.	.	.	.	0.4519065	5.31E-05	.
VCAM1	.	.	0.3774887	0.0009145	.	.	.	.	0.3925157	0.0005422	.	.	.
VCAN	.	.	0.5126032	3.02E-06	.	.	0.389222	0.0006093	.	.	.	.	yes
VCL	.	.	0.4352481	0.0001064	.	.	.	.	.	.	.	.	yes
VDAC1	.	.	.	.	.	.	.	.	0.7424438	3.71E-14	.	.	yes
VDAC2	.	.	.	.	.	.	.	.	0.7690542	1.22E-15	.	.	.
VEZF1	.	.	.	.	.	.	.	.	0.6793091	2.86E-11	.	.	.
VIM	.	.	0.5228256	1.77E-06	.	.	.	.	0.5471369	4.56E-07	.	.	yes
VIMP	.	.	.	.	.	.	.	.	0.5662537	1.45E-07	.	.	.
VNN1	.	.	.	.	.	.	.	.	0.5000772	5.72E-06	.	.	.
VPREB3	.	.	.	.	.	.	0.4798261	1.52E-05	.	.	.	.	.
VPS13D	.	.	.	.	.	.	.	.	0.5727333	9.71E-08	.	.	.
VPS16	.	.	.	.	.	.	.	.	0.6247964	2.69E-09	.	.	.
VPS28	.	.	0.3901435	0.0005898	.	.	.	.	.	.	.	.	.
VPS35	.	.	.	.	.	.	.	.	.	.	.	.	yes
VPS37B	.	.	.	.	.	.	.	.	0.5376967	7.81E-07	.	.	.
VPS39	.	.	.	.	.	.	.	.	0.4431559	7.68E-05	.	.	.
VRK1	.	.	0.4326285	0.0001183	.	.	.	.	.	.	.	.	.
VRK3	.	.	.	.	.	.	.	.	0.4639517	3.14E-05	.	.	.
VWA7	.	.	.	.	.	.	0.5344607	9.36E-07	.	.	.	.	.
WAPAL	.	.	.	.	.	.	.	.	0.4620814	3.41E-05	.	.	.
WARS	.	.	.	.	.	.	.	.	0.6272814	2.23E-09	.	.	.
WAS	.	.	0.441686	8.17E-05	.	.	.	.	.	.	.	.	.
WASH1	.	.	0.3756122	0.0009746	.	.	.	.	.	.	.	.	.
WASL	.	.	.	.	.	.	.	.	0.4391499	9.07E-05	.	.	.
WBP11	.	.	.	.	0.7177657	6.18E-13	.	.	.	.	.	.	yes
WBP1L	.	.	0.4369813	9.91E-05	.	.	.	.	.	.	.	.	.
WBP5	.	.	.	.	.	.	.	.	.	.	.	.	yes
WBSCR22	.	.	.	.	.	.	.	.	0.6625104	1.28E-10	.	.	.
WBSCR27	.	.	.	.	.	.	.	.	0.6822525	2.18E-11	.	.	.
WDR1	.	.	.	.	.	.	.	.	0.624796	2.69E-09	.	.	yes
WDR11	.	.	.	.	.	.	.	.	0.459343	3.84E-05	.	.	.
WDR26	.	.	.	.	.	.	.	.	.	.	.	.	yes
WDR33	.	.	.	.	.	.	.	.	.	.	0.4547767	4.69E-05	.
WDR37	.	.	.	.	.	.	.	.	.	.	0.4451967	7.05E-05	.
WDR41	.	.	0.42604	0.000154	.	.	.	.	.	.	.	.	.
WDR53	.	.	.	.	.	.	.	.	0.7236968	3.23E-13	.	.	.
WDR6	.	.	.	.	.	.	.	.	.	.	.	.	yes
WDR62	.	.	.	.	.	.	.	.	0.5926925	2.65E-08	.	.	.
WDR82	.	.	0.3923091	0.0005462	.	.	.	.	.	.	.	.	yes
WDR83	.	.	.	.	.	.	.	.	0.7379985	6.30E-14	.	.	.
WDR89	.	.	.	.	.	.	.	.	0.4487857	6.06E-05	.	.	.
WDR90	.	.	.	.	.	.	.	.	.	.	0.4792821	1.56E-05	.

Gene	Atrial Expression	p-value Atrial	AV-Canal Expression	p-value AV-Canal	Branchial Arches Expression	p-value Branchial Arches	Heart Tube Expression	p-value Heart Tube	Outflow Tract Expression	p-value Outflow Tract	Ventricle Expression	p-value Ventricle	DMP Expression
WDR91	.	.	0.3815038	0.0007972	.	.	.	.	.	.	.	.	.
WDR93	.	.	.	.	.	.	.	.	0.8369109	1.59E-20	.	.	.
WDR96	.	.	.	.	.	.	.	.	.	.	0.6054039	1.10E-08	.
WDSUB1	.	.	0.3799482	0.000841	.	.	.	.	.	.	.	.	.
WDTC1	.	.	.	.	0.3024239	0.0088216	0.598516	1.78E-08	.	.	.	.	.
WEE1	.	.	.	.	.	.	.	.	0.5559617	2.71E-07	.	.	.
WEE2	.	.	.	.	.	.	0.5173497	2.36E-06	.	.	.	.	.
WFDC8	.	.	.	.	.	.	.	.	.	.	0.6115539	7.12E-09	.
WFS1	.	.	0.3969893	0.0004618	.	.	.	.	0.3515193	0.0021331	.	.	.
WHSC1	.	.	.	.	0.3849879	0.0007067	.	.	.	.	.	.	yes
WHSC1L1	.	.	0.4548106	4.68E-05	.	.	.	.	.	.	.	.	.
WIBG	0.5664623	1.44E-07	.	.	.	.	.	.	.	.	.	.	.
WNK1	.	.	0.3271556	0.0044394	.	.	.	.	.	.	.	.	yes
WNT9B	.	.	0.5370333	8.10E-07	.	.	.	.	.	.	.	.	.
WRAP53	.	.	.	.	.	.	.	.	0.769643	1.13E-15	.	.	.
WRB	.	.	.	.	.	.	.	.	0.560475	2.07E-07	.	.	.
WSB1	.	.	.	.	.	.	.	.	0.3915675	0.0005608	.	.	yes
WSB2	.	.	0.4123394	0.0002617	.	.	.	.	0.4066219	0.0003244	.	.	.
WT1	.	.	0.4065107	0.0003257	.	.	.	.	.	.	.	.	.
WWC1	.	.	.	.	.	.	.	.	.	.	0.5643608	1.63E-07	.
WWC2	.	.	0.4353187	0.0001061	.	.	.	.	.	.	.	.	.
WWP2	.	.	.	.	.	.	.	.	0.4788704	1.59E-05	.	.	.
XIRP1	.	.	0.5185974	2.21E-06	.	.	.	.	.	.	.	.	.
XK	.	.	0.4871716	1.07E-05	.	.	.	.	.	.	.	.	.
XPNPEP1	.	.	0.3995613	0.0004207	.	.	.	.	0.3742311	0.001021	.	.	.
XPO1	.	.	.	.	.	.	.	.	.	.	.	.	yes
XPO6	.	.	.	.	.	.	.	.	0.4374515	9.73E-05	.	.	.
XPO7	.	.	.	.	.	.	.	.	.	.	.	.	yes
XRCC3	.	.	.	.	.	.	.	.	0.4740661	1.98E-05	.	.	.
XXYLT1	.	.	.	.	.	.	.	.	0.7064808	2.03E-12	.	.	.
XYLT2	.	.	0.3108149	0.0070321	0.4383513	9.37E-05	.	.	.	.	.	.	.
YAF2	.	.	0.5794482	6.33E-08	.	.	.	.	.	.	.	.	.
YAP1	.	.	.	.	.	.	.	.	0.4230831	0.000173	.	.	yes
YBX1	.	.	.	.	.	.	.	.	0.3476761	0.0024038	.	.	yes
YDJC	.	.	.	.	.	.	.	.	0.5509562	3.65E-07	.	.	.
YEATS2	.	.	.	.	.	.	.	.	.	.	.	.	yes
YEATS4	.	.	0.4116676	0.0002684	.	.	.	.	.	.	.	.	.
YIF1A	.	.	0.4176043	0.000214	.	.	.	.	0.4470129	6.53E-05	.	.	.
YIPF6	.	.	.	.	.	.	.	.	0.5641511	1.65E-07	.	.	.
YKT6	.	.	.	.	.	.	.	.	0.7087147	1.61E-12	.	.	.
YME1L1	.	.	.	.	.	.	.	.	.	.	.	.	yes
YPEL2	.	.	0.4686394	2.54E-05	.	.	.	.	0.4706993	2.31E-05	.	.	.
YPEL5	.	.	.	.	.	.	.	.	0.4647348	3.03E-05	.	.	.
YTHDF1	.	.	.	.	.	.	.	.	0.6794537	2.83E-11	.	.	.
YTHDF2	.	.	.	.	.	.	.	.	0.4708099	2.30E-05	.	.	.
YWHAB	.	.	.	.	.	.	.	.	0.4178414	0.0002121	.	.	yes
YWHAE	.	.	.	.	.	.	.	.	.	.	.	.	yes
YWHAG	.	.	.	.	.	.	.	.	0.3197539	0.0054845	.	.	yes
YWHAH	.	.	0.4670933	2.72E-05	.	.	.	.	0.5473673	4.50E-07	.	.	.
YWHAQ	.	.	0.3757	0.0009717	.	.	.	.	0.3650971	0.001382	.	.	.
YWHAZ	.	.	.	.	.	.	.	.	.	.	.	.	yes
YY2	.	.	.	.	.	.	.	.	0.4606162	3.63E-05	.	.	.
ZAN	.	.	.	.	.	.	.	.	.	.	0.5417719	6.20E-07	.
ZBTB1	.	.	0.3771048	0.0009265	.	.	.	.	.	.	.	.	.
ZBTB25	.	.	.	.	.	.	.	.	.	.	0.518748	2.19E-06	.
ZBTB6	.	.	0.460639	3.63E-05	.	.	.	.	.	.	.	.	.
ZBTB9	.	.	0.4188316	0.0002041	.	.	.	.	.	.	.	.	.
ZC2HC1B	.	.	0.4537426	4.90E-05	.	.	.	.	.	.	.	.	.
ZC3H12A	.	.	.	.	.	.	.	.	.	.	0.5320744	1.07E-06	.
ZC3H12D	.	.	0.3169559	0.0059329	.	.	.	.	0.5678431	1.32E-07	.	.	.
ZC3H4	.	.	.	.	.	.	.	.	.	.	.	.	yes
ZC3H7B	.	.	.	.	.	.	.	.	0.6930681	7.77E-12	.	.	yes
ZC3HAV1L	.	.	.	.	.	.	.	.	.	.	.	.	yes
ZC4H2	.	.	0.3789288	0.0008708	.	.	.	.	.	.	.	.	.
ZCCHC11	.	.	.	.	0.4372586	9.80E-05	.	.	.	.	.	.	yes
ZCWPW2	.	.	0.4034234	0.0003652	.	.	.	.	.	.	.	.	.
ZDHHC14	.	.	.	.	0.496443	6.85E-06	.	.	0.3443756	0.0026604	.	.	.
ZDHHC5	.	.	.	.	.	.	.	.	0.6671505	8.56E-11	.	.	.
ZDHHC8	.	.	0.3820146	0.0007833	0.3308838	0.0039831	.	.	.	.	.	.	.
ZDHHC9	.	.	.	.	.	.	.	.	0.6988973	4.38E-12	.	.	.
ZFAND4	.	.	.	.	.	.	.	.	.	.	0.5574623	2.48E-07	.
ZFAT	.	.	.	.	.	.	.	.	.	.	0.7251792	2.74E-13	.
ZFHX2	.	.	.	.	0.4511176	5.49E-05	.	.	.	.	.	.	.
ZFP36L1	.	.	0.3431612	0.0027608	.	.	.	.	.	.	.	.	yes
ZFP62	.	.	.	.	.	.	.	.	0.5369678	8.13E-07	.	.	.

Gene	Atrial Expression	p-value Atrial	AV-Canal Expression	p-value AV-Canal	Branchial Arches Expression	p-value Branchial Arches	Heart Tube Expression	p-value Heart Tube	Outflow Tract Expression	p-value Outflow Tract	Ventricle Expression	p-value Ventricle	DMP Expression
ZFP91	.	.	0.3469756	0.0024563	.	.	.	.	0.6082538	9.02E-09	.	.	.
ZFPM1	.	.	0.3059599	0.008024	.	.	.	.	0.6894491	1.10E-11	.	.	.
ZFR	.	.	.	.	.	.	.	.	0.4946024	7.49E-06	.	.	yes
ZFYVE1	.	.	.	.	.	.	.	.	0.4385337	9.30E-05	.	.	.
ZFYVE21	.	.	.	.	.	.	.	.	0.4934413	7.93E-06	.	.	.
ZHX2	.	.	.	.	.	.	.	.	0.5335501	9.84E-07	.	.	.
ZKSCAN5	.	.	0.3895079	0.0006032	.	.	.	.	.	.	.	.	.
ZMAT5	.	.	0.3791326	0.0008647	.	.	.	.	.	.	.	.	.
ZMIZ1	.	.	.	.	.	.	.	.	.	.	.	.	yes
ZMPSTE24	.	.	0.411074	0.0002745	.	.	.	.	0.5154694	2.61E-06	.	.	.
ZMYM1	.	.	.	.	.	.	.	.	0.4797489	1.53E-05	.	.	.
ZMYM3	.	.	.	.	.	.	.	.	0.32035	0.0053929	0.5057015	4.31E-06	.
ZMYM4	.	.	0.4697356	2.42E-05	.	.	.	.	.	.	.	.	.
ZMYND19	.	.	.	.	0.3256194	0.0046404	.	.	0.7081181	1.71E-12	.	.	.
ZNF106	.	.	.	.	.	.	.	.	.	.	.	.	yes
ZNF143	.	.	.	.	.	.	.	.	0.6732595	4.97E-11	.	.	.
ZNF146	.	.	.	.	.	.	0.4640154	3.13E-05	.	.	.	.	.
ZNF207	.	.	.	.	.	.	.	.	0.541599	6.26E-07	.	.	yes
ZNF219	.	.	.	.	.	.	.	.	.	.	0.5598246	2.15E-07	.
ZNF22	.	.	0.4279163	0.0001429	.	.	.	.	.	.	.	.	.
ZNF259	.	.	.	.	.	.	.	.	0.5021981	5.14E-06	.	.	.
ZNF266	.	.	.	.	.	.	.	.	.	.	.	.	yes
ZNF275	.	.	0.3804557	0.0008265	.	.	.	.	.	.	.	.	.
ZNF280B	.	.	.	.	.	.	.	.	0.5591044	2.25E-07	.	.	.
ZNF281	.	.	.	.	.	.	.	.	0.7414216	4.20E-14	.	.	.
ZNF330	.	.	.	.	0.4393142	9.01E-05	.	.	.	.	.	.	.
ZNF334	.	.	.	.	0.4812147	1.42E-05	.	.	.	.	.	.	.
ZNF335	.	.	.	.	.	.	.	.	0.4904921	9.15E-06	.	.	.
ZNF346	.	.	0.4512939	5.45E-05	.	.	.	.	.	.	.	.	.
ZNF354B	.	.	.	.	0.463105	3.26E-05	.	.	.	.	.	.	.
ZNF384	.	.	.	.	.	.	.	.	0.8129315	1.42E-18	.	.	.
ZNF394	.	.	.	.	.	.	.	.	0.5338454	9.68E-07	.	.	.
ZNF397	.	.	.	.	0.4610856	3.56E-05	.	.	.	.	.	.	.
ZNF423	.	.	.	.	0.4827599	1.32E-05	.	.	.	.	.	.	.
ZNF445	.	.	.	.	.	.	.	.	.	.	.	.	yes
ZNF446	.	.	.	.	0.5056948	4.31E-06	.	.	.	.	.	.	.
ZNF462	.	.	.	.	.	.	.	.	.	.	0.3171232	0.0059052	yes
ZNF496	.	.	.	.	.	.	.	.	.	.	0.4898942	9.42E-06	.
ZNF511	.	.	.	.	.	.	.	.	0.4923552	8.36E-06	.	.	.
ZNF516	.	.	.	.	.	.	.	.	.	.	.	.	yes
ZNF521	.	.	.	.	0.5108059	3.32E-06	.	.	.	.	.	.	.
ZNF563	.	.	0.4599157	3.75E-05	.	.	.	.	.	.	.	.	.
ZNF565	.	.	0.4200559	0.0001947	.	.	.	.	.	.	.	.	.
ZNF580	.	.	0.3754021	0.0009815	.	.	.	.	.	.	.	.	.
ZNF585A	.	.	.	.	.	.	.	.	.	.	0.5111884	3.25E-06	.
ZNF592	.	.	.	.	.	.	.	.	.	.	0.5941428	2.40E-08	.
ZNF593	.	.	.	.	0.3397692	0.0030595	.	.	0.5505386	3.74E-07	.	.	.
ZNF609	.	.	.	.	.	.	.	.	.	.	0.5863987	4.03E-08	.
ZNF624	.	.	.	.	.	.	.	.	.	.	0.6126325	6.59E-09	.
ZNF628	.	.	.	.	.	.	.	.	0.5338819	9.66E-07	.	.	.
ZNF629	.	.	.	.	.	.	.	.	.	.	0.6643822	1.09E-10	.
ZNF664	.	.	.	.	.	.	.	.	.	.	.	.	yes
ZNF668	.	.	0.4189183	0.0002034	.	.	.	.	.	.	.	.	.
ZNF687	.	.	0.434889	0.000108	.	.	.	.	0.3490564	0.0023032	.	.	.
ZNF697	.	.	.	.	.	.	.	.	.	.	0.5490689	4.07E-07	.
ZNF710	.	.	.	.	.	.	.	.	0.5772339	7.30E-08	.	.	.
ZNF800	.	.	.	.	.	.	.	.	.	.	0.6110478	7.39E-09	.
ZNF804B	.	.	0.5524928	3.33E-07	.	.	.	.	0.5566647	2.60E-07	.	.	.
ZNF827	.	.	.	.	.	.	.	.	0.5321013	1.07E-06	.	.	.
ZNF846	.	.	.	.	.	.	.	.	.	.	0.5302543	1.18E-06	.
ZNF850	.	.	.	.	.	.	.	.	0.4599253	3.75E-05	.	.	.
ZNF90	.	.	.	.	.	.	.	.	.	.	0.5457422	4.94E-07	.
ZNHIT6	.	.	.	.	.	.	.	.	0.5669647	1.39E-07	.	.	.
ZNRF1	.	.	.	.	.	.	.	.	0.7391338	5.51E-14	.	.	.
ZRANB2	.	.	.	.	.	.	.	.	.	.	.	.	yes
ZSCAN26	.	.	.	.	.	.	.	.	0.5471307	4.56E-07	.	.	.
ZSCAN29	.	.	.	.	0.4699685	2.39E-05	.	.	.	.	.	.	.
ZSWIM3	.	.	.	.	.	.	.	.	0.7053135	2.29E-12	.	.	.
ZSWIM8	.	.	.	.	.	.	.	.	.	.	.	.	yes
ZWINT	.	.	0.4692535	2.47E-05	.	.	.	.	0.4877247	1.05E-05	.	.	.
ZYX	.	.	0.4327482	0.0001178	.	.	.	.	0.4733904	2.05E-05	.	.	.
