# Supplementary material for: De Novo and Rare Variants at Multiple Loci Support the Oligogenic Origins of Atrioventricular Septal Heart Defects
Source: PLoS Genet. 2016 Apr 8;12(4):e1005963. doi: 10.1371/journal.pgen.1005963 (PMC4825975; doi:10.1371/journal.pgen.1005963)
Supplement: S9 Table — (PDF) [file pgen.1005963.s016.pdf]

Table S9. 13 Well Known Cardiac Developmental Genes are Appropriately Localized to Developmental Compartment by Unsupervised Weighted Gene Co-Expression Network Analysis of SAGE expression data

Gene	Associated Malformation	Developmental Compartment
Jag1	Pulmonary Stenosis/Alagille Syndrome	outflow tract
Ptpn11	Pulmonary Stenosis/Tetralogy of Fallot	outflow tract
Hey2	Ventricular Septal Defect	outflow tract
Tbx5	Chamber Septation/Holt-Oram Syndrome	av-canal
Gata4	ASD/VSD/AVSD	av-canal
Nkx2-5	ASD/VSD/AVSD	av-canal
Hand2	Abnormal endocardial cushions	outflow tract
Myh7	Ebstein's Anomaly/ASD/VSD/BaV	outflow tract
MesP1	Key Mesodermal Progenitor	av-canal
Creld1	AVSD	av-canal
Kmt2d	VSD/Kabuki Syndrome	outflow tract
Myh6	ASD	atria
Kdm5b	LV-outflow tract abnormalities	ventricle
