# Supplementary material for: De Novo and Rare Variants at Multiple Loci Support the Oligogenic Origins of Atrioventricular Septal Heart Defects
Source: PLoS Genet. 2016 Apr 8;12(4):e1005963. doi: 10.1371/journal.pgen.1005963 (PMC4825975; doi:10.1371/journal.pgen.1005963)
Supplement: S10 Table — (PDF) [file pgen.1005963.s017.pdf]

**Table S10. Ocular Malformation Genes**

*AGAP1*
*AGT*
*ASPA*
*CDKN1C*
*COMP*
*CPOX*
*CRX*
*CYBA*
*EDNRB*
*EMD*
*ERCC8*
*FANCA*
*FAS*
*GALK1*
*GJB1*
*GLI3*
*GPR143*
*GSN*
*HPS1*
*IDS*
*INS*
*KRT12*
*LEP*
*LYST*
*MYO7A*
*NAGLU*
*NDP*
*NF1*
*NPC1*
*NPHP1*
*NRL*
*OAT*
*OTX2*
*PAH*
*PAX6*
*PDE6B*
*PEX7*
*RAX*
*SGSH*
*SIX3*
*SIX6*
*VAX1*
*VSX2*
